# Supplementary material for: Comparative Evaluation of Breast Ductal Carcinoma Grading: A Deep-Learning Model and General Pathologists’ Assessment Approach
Source: Diagnostics (Basel). 2023 Jul 10;13(14):2326. doi: 10.3390/diagnostics13142326 (PMC10377791; doi:10.3390/diagnostics13142326)
Supplement: Supplementary file 1 [file diagnostics-13-02326-s001.zip › S1 - Ambassador WSI list and ID from TCGA-BRCA.pdf]

Case UUID	Name
92b020c8-1e0d-4f20-8f34-b9c66b2757b1	TCGA-BH-A0B7-01Z-00-DX1.6950CDDF-8A81-4B10-BFFF-BE0E33A2C6CC.svs
9af833c0-30df-4772-91b2-31e4e312f058	TCGA-A2-A0CQ-01Z-00-DX1.4E5FB4E5-A08C-4C87-A3BE-0640A95AE649.svs
2be609ad-4469-412b-a872-c0047698736d	TCGA-AN-A0FZ-01Z-00-DX1.9555AF11-3A0D-4FE3-AE91-09DA77B175CA.svs
619bee8c-e250-49a2-86c0-766112714bb6	TCGA-D8-A27L-01Z-00-DX1.6572593C-B015-4F9A-8C9B-B634CAA0D3B4.svs
b56643ef-970e-48cb-bf38-60bfa688f1e5	TCGA-BH-A0H3-01Z-00-DX1.C6D4DFB9-A4FA-40B2-90EF-75294CBC4523.svs
64750d75-af48-416f-802f-78302509dcb8	TCGA-D8-A1XW-01Z-00-DX2.9849E503-BE3E-417C-ABE8-93A39583DDE0.svs
71d04401-9aaf-4f80-8948-6b0a67c4c965	TCGA-D8-A1JU-01Z-00-DX1.355D93B4-E69E-417C-B3D1-3E1AAF1E02FE.svs
32732b3d-a99a-4653-b8ef-763a15828349	TCGA-D8-A1X9-01Z-00-DX1.28CE7849-EEC5-4ABB-A319-A977A1FD3CD1.svs
64cf590d-97ba-46a6-a9b0-b79a415de9d3	TCGA-EW-A2FS-01Z-00-DX1.A01C9183-2AC8-456A-B5A6-85C5BB0361D8.svs
f071614d-2ddf-42c8-92a1-ef24cbee8bfc	TCGA-C8-A12N-01Z-00-DX1.8E50110E-A6C0-496F-B44E-7190096C113E.svs
