# Supplementary material for: Comparative Evaluation of Breast Ductal Carcinoma Grading: A Deep-Learning Model and General Pathologists’ Assessment Approach
Source: Diagnostics (Basel). 2023 Jul 10;13(14):2326. doi: 10.3390/diagnostics13142326 (PMC10377791; doi:10.3390/diagnostics13142326)
Supplement: Supplementary file 1 [file diagnostics-13-02326-s001.zip › S2 - Pathology reports of the 100 WSI from TCGA-BRCA.pdf]

UUID:265E5A9A-64FD-4B86-89BC-5E89F253C118
TCGA-3C-AALJ-01A-PR Redacted

Name:	[redacted]	Path No.:	[redacted]
MRN:		Date Obtained:	
D.O.B.	(Age:)	Date Received:	
Sex:	F	Physician:	
Location:			

SURGICAL PATHOLOGY

ICD-O-3
Carcinoma, infiltrating duct NOS 8500/3
Site (R) Breast C50.9
path (R) Breast, midline C50.8
8/11/14

SPECIMEN:
Breast, right, skin sparing modified radical mastectomy

DIAGNOSIS(ES):
Breast, right, skin sparing modified radical mastectomy:

Invasive ductal carcinoma, poorly differentiated (Modified Scarff Bloom Richardson Score 3+3+2=8/9), multifocal, with lymphatic invasion.
Metastatic carcinoma in 1/23 axillary lymph nodes (See microscopic description)
pT2N1

CLINICAL INFORMATION: Right breast cancer.

GROSS DESCRIPTION:
The specimen is received unfixed in a container labeled with the patient's name and "Right breast mastectomy". It consists of a right modified mastectomy specimen measuring 24.0 x 20.0 x 5.2 cm. A suture is noted indicating the axillary tail. The overlying skin measures 14.5 x 8.0 cm. The nipple is mobile and everted. An underlying mass is palpable in the 6 o'clock position. The deep (fascial) margin is inked black and the remaining margins are inked yellow. The axillary tissue measures 11.0 x 6.5 x 2.0 cm. Multiple lymph nodes are palpable within it measuring from 0.5 cm to 1.7 cm in greatest dimension. The lymph nodes are dissected, proceeding from the breast toward the axilla.

The specimen is serially sectioned at closely spaced intervals revealing a firm, poorly circumscribed, white and focally hemorrhagic mass measuring 3.5 x 3.0 x 2.0 cm located in the inferior aspect of the specimen (6 o'clock position). The mass comes to within 0.8 cm of the skin and 3.0 cm of the deep margin. A second firm, well circumscribed mass measuring 0.7 x 0.5 x 0.5 is also identified located located 0.8 cm from the skin, 2.4 cm from the deep margin and 2.0 cm from the previously described mass, superiorly. The parenchyma superior and lateral to both masses is firm, lobulated and gritty over an area measuring 4.0 x 3.5 x 2.0 cm. The remaining tissue is composed of a moderate amount of breast tissue and a moderate amount of yellow fatty tissue. Representative sections are submitted in 37 cassettes labeled A1-A37. Please note: The specimen was placed in formalin at

LEGEND:
A1-A3 = Nipple
A4 = Deep margin closest to main mass
A5 = Main mass with closest skin
A6-A8 = Main mass
A9-A10 = Smaller mass
A11 = Smaller mass with closest skin
A12 = Deep margin closest to smaller mass

A13-A18 = Gritty area surrounding masses
A19-A20 = Upper inner quadrant
A21-A22 = Lower inner quadrant
A23-A24 = Lower outer quadrant
A25-A26 = Upper outer quadrant
A27 = Bisected lymph node closest to breast
A28 =3 intact nodes
A29 = 3 intact nodes
A30 = 1 bisected node
A31 = 1 bisected node
A32 = 1 bisected node
A33 = 2 intact nodes
A34 = 4 intact nodes
A35 = 3 intact nodes
A36 = 2 intact nodes
A37 = 1 bisected node at high point.

MICROSCOPIC DESCRIPTION:

I. TYPE OF SPECIMEN: Right modified radical mastectomy

II. LOCATION OF THE TUMOR: Central (6 o'clock), extending into lower inner and outer quadrants.

III. TYPE OF NEOPLASM: Carcinoma, invasive, ductal, with central scar and micropapillary features.

Poorly Differentiated, Total score 8
(Tubule Score 3, Nuclear Grade Score 3, Mitotic Score 2)

Ductal carcinoma in situ, nuclear grade 3, multifocal 30 %

Intraductal papillary subtype
Intraductal micropapillary subtype
Intraductal solid subtype
Intraductal comedo subtype

Necrosis is present within the intraductal carcinoma

Lobular neoplasia is not present

IV. GROSS/MICRO FINAL INVASIVE TUMOR SIZE INTERPRETATION: 3.5 x 3.0 x 2.0 cm. (In addition, separate foci of invasive carcinoma are seen superior to and lateral to the main mass these have a similar histology; the largest such focus measures 1.5cm on the slide.

VI. VASCULAR SPACE INVASION: Present in lymphatics

VII. CALCIFICATION: Present in malignant areas

VIII. NIPPLE: Present, uninvolved by cancer

IX. SKIN: Present, uninvolved by cancer

X. ADJACENT BREAST TISSUE: Cystic disease, proliferative with atypia

XI. MARGINS: Negative

XII. AXILLARY LYMPH NODES:

XII. AXILLARY LYMPH NODES:

[redacted] [redacted]

TOTAL: 23

HIGH POINT: 1

XIII. POSITIVE LYMPH NODES:

XIII. POSITIVE LYMPH NODES:
TOTAL: 1
LEVEL I: 1 (adjacent to breast)

EXTRANODAL EXTENSION: Present

XIV. PECTORAL MUSCLE: No pectoral muscle identified

XV. PATHOLOGIC STAGING (pTNM) *AJCC 7th Edition*: ***Reflects staging only of the current specimen. Ultimate staging responsibility rests with the primary physician.***

pT2: Tumor more than 2.0 cm but not more than 5.0 cm in greatest dimension

pN1a: Metastasis in 1 to 3 axillary lymph nodes (at least 1 tumor deposit greater than 2.0 mm)

This report has been reviewed electronically and signed on by

Interpreted by: Attending:

The diagnosis was rendered by the attending pathologist.

Criteria lw 1/21/14	Yes	No
Diagnosis Discrepancy		✓
Primary Tumor Site Discrepancy		✓
HIPAA Discrepancy		✓
Prior Malignancy History		✓
Dual/Synchronous Primary Noted		✓
Case is (circle): QUALIFIED / DISQUALIFIED		
Reviewer Initials [illegible] Date Reviewed: 1/ /2014		

Note: Immunochemistry testing performed at was developed and its performance characteristics determined by the These tests were interpreted in conjunction with external positive and internal negative controls, unless otherwise noted. It has not been cleared or approved by the US FDA. This test is used for clinical purposes only. It should not be regarded as investigational or for research.

Criteria	Yes	No
Diagnosis Discrepancy		✓
Primary Tumor Site Discrepancy		✓
HIPAA Discrepancy		✓
Prior Malignancy History		✓
Dual/Synchronous Primary Noted		✓
Case is (circle): QUALIFIED / DISQUALIFIED		
Reviewer Initials RB Date Reviewed: 9/29/11		

lw 10/21/11

UUID:1617B36B-4EFB-445E-95BC-E63B129AAAE7
TCGA-A1-A0SF-01A-PR Redacted

ICD-O-3
Carcinoma, infiltrating duct, NOS 8500/3
Site: breast, NOS C50.9 lw 10/21/11

Final Pathologic Diagnosis:

A. Sentinel lymph node #1, left axilla, biopsy: No carcinoma identified in one lymph node (0/1).

B. Sentinel lymph node #2, left axilla, biopsy: No carcinoma identified in one lymph node (0/1).

C. Left breast, mastectomy;
1. Infiltrating ductal carcinoma, SBR grade 2, 2.4 cm; see comment.
2. Intermediate to high grade ductal carcinoma in-situ, cribriform and comedo types.
3. Non-proliferative fibrocystic changes.
4. Duct ectasia.

D. Right breast, mastectomy:
1. Atypical lobular hyperplasia.
2. Non-proliferative fibrocystic changes.
3. Duct ectasia.
4. Microcalcifications associated with benign ducts and stroma.
5. No carcinoma identified.

E. Soft tissue, right breast, excision: Fibroadipose tissue with skeletal muscle; no breast parenchyma or carcinoma identified.

F. Left ovary and fallopian tube, salpingooophorectomy:
- Ovary: No significant pathologic abnormality.
- Fallopian tube: Benign paratubal cyst.

G. Right ovary and fallopian tube, salpingooophorectomy:
- Ovary: No significant pathologic abnormality.
- Fallopian tube: Benign paratubal cyst.

H. Omentum, biopsy: Benign fibrous nodule.

I. Soft tissue, left upper peritoneum, biopsy: Benign fibroadipose tissue.

J. Omentum, omentectomy: No significant pathologic abnormality.

Note: *Breast Tumor Synoptic Comment*
- Laterality: Left.
- Invasive tumor type: Infiltrating ductal.
- Invasive tumor size: 2.4 cm maximum diameter.
- Invasive tumor grade (modified Bloom-Richardson):
  - Nuclear grade: 3, 3 points.
  - Mitotic count: 7 mitotic figures/10 HPF, 1 point.
  - Tubule/papilla formation: >10% but <75%, 2 points.
  - Total points and SBR grade = 6 points, grade 2.
- Lymphatic-vascular invasion: None identified.
- Perineural invasion: None identified.
- Invasive tumor necrosis: None identified.
- Mononuclear cell reaction: None/minimal.
- Resection margins for invasive tumor: Widely clear.
  - Deep margin: Widely clear; closest distance of tumor 2 cm.
  - Medial margin: Widely clear; closest distance of tumor > 6 cm.
  - Lateral margin: Widely clear; closest distance of tumor 2.8 cm.
  - Anterior/superior margin: Widely clear; closest distance of tumor 5.5 cm.
  - Anterior/inferior margin: Widely clear; closest distance of tumor >6 cm.

- Ductal carcinoma in situ (DCIS) type: Cribriform and comedo.
- Ductal carcinoma in situ size: ~2.0 cm in maximum diameter.
- Ductal carcinoma in situ nuclear grade: Intermediate to high grade.
- Necrosis in ductal carcinoma in situ: Present.
- Microcalcifications: Not identified.

- Resection margins for ductal carcinoma in situ: Widely clear (see above for invasive tumor.

- Lobular carcinoma in situ (LCIS): Not identified.
- Number of lobules involved: N/A.
- Nuclear type/size: N/A.
- Resection margins for pleomorphic lobular carcinoma in situ: N/A.

- Lymph node status: Negative.
    - Number of positive lymph nodes: 0.
    - Total number sampled: 2.

- AJCC/UICC stage: pT2N0Mx.

- Nontumorous breast tissue: Non-proliferative fibrocystic change; duct ectasia.
- Nipple: Unremarkable.
- Skin/dermis: Unremarkable.

Pagetoid spread of carcinoma is seen in benign ducts adjacent to the tumor. No such spread is seen away from the tumor.

Atypical lobular hyperplasia is present in the contralateral (right) breast.

An immunohistochemical test for estrogen and progesterone receptors as well as for HER2 was performed on block C3.

The test for estrogen receptors is positve. There is moderate nuclear staining in >95% of tumor cells. Internal positive control is positive.

The test for progesterone receptors is positive. There is moderate to strong nuclear staining in 90% of tumor cells. Internal positive control is positive.

Result of HER2/neu test: This carcinoma is negative for HER2/neu oncoprotein over-expression.

An immunohistochemical assay was performed using the CB11 monoclonal antibody to HER2/neu oncoprotein. The staining intensity of this carcinoma was 1 on a scale of 0-3 (HER2 test interpreted by Dr.

Carcinomas with staining intensity scores of *0 or 1* are considered *negative* for over-expression of HER2/neu oncoprotein.

Those with a staining intensity score of *2* are considered *borderline*. We and others have observed that many carcinomas with staining intensity scores of 2 do not show gene amplification. All carcinomas with staining intensity scores of 2 are therefore submitted for FISH testing. The results of the FISH test are issued directly from the molecular cytogenetics laboratory.

Carcinomas with staining intensity scores of *3* are considered *positive* for over-expression of HER2/neu oncoprotein. Tumors in this category show an excellent correlation between the results of immunohistochemical and FISH testing, and almost always show gene amplification.

Intraoperative Consult Diagnosis

FS1 (A) Sentinel lymph node #1, left axilla, biopsy: No carcinoma.

FS2 (B) Sentinel lymph node #2, left axilla, biopsy: No carcinoma.

FS3 (G) Right adnexa, salpingo-oophorectomy: Paratubal cyst with hemorrhage.

FS4 (H) Omentum, biopsy: Dense fibrous connective tissue, no carcinoma.

FS5 (I) Left upper quadrant, peritoneum, biopsy: No carcinoma.

F. Left adnexa, salpingo-oophorectomy: Paratubal cyst (gross diagnosis only).

Clinical History

The patient is a -year-old woman with left breast cancer. She undergoes bilateral mastectomies.

Gross Description

The specimen is received in ten parts, each labeled with the patient's name and unit number. Parts A through I are received fresh, and Part J is received in formalin.

Part A, additionally labeled "1 consists of one pink, unoriented, fibroadipose tissue fragment measuring 1.5 x 0.8 x 0.8 cm. The entire specimen is frozen for frozen section diagnosis 1, and subsequently submitted in cassette A1.

Part B, additionally labeled consists of one pink-red, unoriented, fibroadipose tissue fragment measuring 1.3 x 0.8 x 0.7 cm. The entire specimen is frozen for frozen section diagnosis 2, and subsequently submitted in cassette B1.

Part C is additionally labeled " It consists of a mastectomy specimen, measuring 15.8 cm from superior to inferior, 15.6 cm from medial to lateral, and 8.2 cm from anterior to posterior. The specimen weighs 442.5 gm. In the lateral aspect of the specimen is a spiculated, firm, tan mass, measuring 2.4 x 1.2 x 1.2 cm. This mass is 2.0 cm from the deep margin, 5.5 cm from the superior margin, >6 cm from the inferior margin, 2.8 cm from the lateral margin, and >6 cm from the medial margin. In the anterior-inferior portion of the breast, there are dilated ducts, filled with cheesy material. This area is 0.2 cm from the deep margin. The remainder of the breast parenchyma is unremarkable. Two pieces of tumor are banked, one for tissue banking and one for the epithelial cell study. Representative sections are submitted as follows:

Cassette C1:	Nipple.
Cassettes C2-C3:	Spiculated mass.
Cassette C4:	Deep margin beneath tumor.
Cassette C5:	Lateral margin.
Cassette C6:	Lower outer quadrant.
Cassette C7:	Lower inner quadrant.
Cassette C8:	Medial margin.
Cassette C9:	Upper inner quadrant.
Cassette C10:	Anterior-inferior area with duct ectasia.

Part D is additionally labeled It consists of a mastectomy specimen, measuring 18.2 cm from superior to inferior, 16.2 cm from medial to lateral, and 4.8 cm from anterior to posterior. The specimen weighs 688 gm. A short stitch is designated by the surgeon as superior, and a long stitch is designated by the surgeon as lateral. In the inferior-medial portion of the specimen is a fragment of skin, measuring 7.5 x 4.9 cm. It is white and unremarkable. There is a nipple, measuring 1.3 x 1.5 x 1.6 cm. The specimen is notable for dilated ducts with a viscous, yellow-tan material within them. This material extends into one of the nipple ducts. No masses are noted, nor are any areas suspicious for DCIS appreciated. The anterior-superior portion of the specimen is inked blue, the anterior-inferior portion of the specimen is inked green, and the posterior aspect of the specimen is inked black. Multiple sections of unremarkable breast parenchyma are taken for two studies, including a high-risk study and an epithelial cell study. Representative sections are submitted as follows:

Cassettes D1-D2:	Nipple and surrounding skin.
Cassette D3:	Representative section of anterior-inferior breast with dilated duct.
Cassette D4:	Representative section of lower inner breast with dilated ducts.
Cassette D5:	Representative section of upper inner breast.
Cassette D6:	Representative section of medial breast.
Cassette D7:	Representative section of upper outer breast.
Cassette D8:	Representative section of upper inner breast.
Cassette D9:	Representative section of lateral breast.

Part E is additionally labeled "

It consists of a single irregular fragment of soft, yellow tissue, measuring 5.4 x 3.8 x 1.5 cm. A suture has been placed on one aspect of the specimen and is designated the new margin. That aspect of the specimen is inked black, and the specimen is serially sectioned and entirely submitted in cassettes E1 through E5.

Part F is received fresh labeled, It consists of an

ovary, measuring 4.3 x 2 x 0.8 cm, with attached fallopian tube, measuring 0.8 x 0.5 x 6 cm. There is a simple paratubal cyst, measuring 1.5 x 1.5 x 0.5 cm. This is received undisrupted, with a thin 0.1 cm translucent wall, and contains 20 cc of clear fluid. In addition, there is a second small paratubal cyst, measuring 0.5 x 0.5 x 0.5 cm. The ovary is serially sectioned, and no abnormalities are detected, other than a 0.5 x 0.5 x 0.5 cm small white firm area. The fallopian tube is serially sectioned, and no abnormalities are detected. Cassettes are submitted as follows:

Cassettes F1-F9: Ovary, entirely submitted (firm, white area in cassette F7).
Cassettes F10-F12: Fallopian tube, entirely submitted.
Cassette F13: Representative section of the paratubal cyst and smaller, 0.5-cm paratubal cyst.
Cassette F14: Representative section of broad ligament.

Part G is additionally labeled It consists of an ovary with attached fallopian tube, weighing 15.9 gm. There is a disrupted hemorrhagic, cystic mass in the broad ligament, measuring 2.3 x 1.6 x 1.5 cm, no capsule is visualized. A representative section of the cystic mass is submitted for frozen section diagnosis as FS3. The ovary and tube do not appear involved by the lesion. The ovary is inked black, it contains a simple corpus luteal cyst measuring 1.5 x 1.6 x 0.8 cm. The ovary measures 3.5 x 3 x 1 cm, and the tube measures 6.5 x 0.8 cm. The ovary and fallopian tube are serially sectioned, and no abnormalities are detected. Cassettes are submitted as follows:

Cassettes G1-G6: Ovary, entirely submitted.
Cassettes G7-G10: Fallopian tube, entirely submitted.
Cassettes G11-G12: Lesion in broad ligament, entirely submitted.
Cassette G13: Frozen section remnant.

Part H is additionally labeled " It consists of an unoriented piece of yellow, adipose tissue, measuring 5 x 1.3 x 0.4 cm, with a white-tan, soft nodule, measuring 0.3 x 0.3 x 0.3 cm, attached to one end. This is entirely submitted for frozen section diagnosis as FS4, with the frozen section remnant submitted in cassette H1. The remaining omentum is entirely submitted in cassette H2.

Part I is additionally labeled It consists of multiple unoriented fragments of tissue, measuring 1 x 0.3 x 0.1 cm in aggregate. The specimen is entirely submitted for frozen section diagnosis as FS5, with the frozen section remnant submitted in cassette I1.

Part J is additionally labeled It consists of an aggregate of fatty tissue, measuring 4 x 2 x 0.5 cm. No abnormalities are detected. 1ne specimen is entirely submitted in cassettes J1 through J3.

/Pathology Resident

Signed: ˜

Fee Codes:

Other Specimens

Specimen Class: Status: Signed Out Accessioned:
Signed Out:

Specimen(s) Received: Right Breast, Fine Needle Aspiration

Final Diagnosis

Right Breast, Fine Needle Aspiration: **Benign scar tissue,** see comment.

Specimen Class: Status: Signed Out Accessioned: Signed Out

Specimen(s) Received: Vaginal/Cervical/Endocervical, Thin Prep Imaged

Final Diagnosis

Vaginal/Cervical/Endocervical, Thin Prep Imaged

NEGATIVE FOR INTRAEPITHELIAL LESION OR MALIGNANCY.
Reactive cellular changes.
Atrophic changes

SPECIMEN ADEQUACY:
Satisfactory for evaluation.
Transformation zone components are present.

_Specimen Class: Status: Signed Out Accessioned. Signed Out:

Specimen(s) Received: Right Breast, implant capsule

Final Diagnosis

Right breast, implant capsule, capsulectomy: Peri-prosthetic capsule.

_Specimen Class: Status: Signed Out Accessioned Signed Out:

Specimen(s) Received: Cervical/Endocervical, Thin Prep Imaged

Final Diagnosis

Cervical/Endocervical, Thin Prep Imaged

NEGATIVE FOR INTRAEPITHELIAL LESION OR MALIGNANCY.
Atrophic changes

SPECIMEN ADEQUACY:
Satisfactory for evaluation.
No transformation zone components are identified.

__Specimen Class: Status: Signed Out Accessioned: Signed Out:

Specimen(s) Received: Endometrium, biopsy

Final Diagnosis

Endometrium, biopsy: Fragments of benign endocervical tissue and no definitive endometrium, see comment.

__Specimen Class: Status: Signed Out Accessioned:
Signed Out:

Specimen(s) Received: Cervical/Endocervical, Thin Prep Imaged

Final Diagnosis

Cervical/Endocervical, Thin Prep Imaged

NEGATIVE FOR INTRAEPITHELIAL LESION OR MALIGNANCY.
Atrophic changes

SPECIMEN ADEQUACY:
Satisfactory for evaluation.
Transformation zone components are present.

_ _________Specimen Class: Status: Signed Out Accessione
Signed Out:

Specimen(s) Received: A: Left breast capsule, B: Right breast capsule

Final Diagnosis

A. Breast capsule, left, excision: Dense fibrous tissue with chronic inflammation.

B. Breast capsule, right, excision: Dense fibrous tissue with chronic inflammation.

__ ___Specimen Class: Status: Signed Out Accessioned:
Signed Out:

Specimen(s) Received: Cervical/Endocervical, Thin Prep Imaged

Final Diagnosis

Cervical/Endocervical, Thin Prep Imaged

NEGATIVE FOR INTRAEPITHELIAL LESION OR MALIGNANCY.
Atrophic changes

SPECIMEN ADEQUACY:
Satisfactory for evaluation.
Transformation zone components are present.

_Specimen Class: Status: N/A Accessioned:
Signed Out:

Specimen(s) Received: A: Skin, biopsy, shave, right scalp, B: Skin, biopsy, shave, left scalp, C: Skin, biopsy, punch, left upper arm

Final Diagnosis **{Final Report Not Signed Out}**

{Not Entered}

_Specimen Class: Status: Signed Out Accessione
Signed Out

Specimen(s) Received: A: Pelvic Washing, B: Diaphragm Washing

Final Diagnosis

A: Pelvic Washing
BENIGN.
Reactive mesothelial cells.

B: Diaphragm Washing
BENIGN.
Reactive mesothelial cells.

Specimen Class: **Status:** Signed Out **Accessioned:**
Signed Out:

Specimen(s) Received: Left Breast, Fine Needle Aspiration

Final Diagnosis

Left Breast, Fine Needle Aspiration: **Adenocarcinoma, morphologically consistent with a primary breast carcinoma**.

Specimen Class: **Status:** Signed Out **Accessioned**
Signed Out:

Specimen(s) Received: Cervical/Endocervical, Thin Prep Imaged

Final Diagnosis

Cervical/Endocervical, Thin Prep Imaged

NEGATIVE FOR INTRAEPITHELIAL LESION OR MALIGNANCY.
Reactive cellular changes.

SPECIMEN ADEQUACY:
Satisfactory for evaluation.
Transformation zone components are present.

Specimen Class: **Status:** Signed Out **Accessioned:**
Signed Out:

Specimen(s) Received: Right foot foreign body

Final Diagnosis

Foot, right, excision: Fragment of hyperkeratotic squamous epithelium.

Specimen Class: **Status:** Signed Out **Accessioned**
Signed Out:

Specimen(s) Received: Cytology, GYN, Site Not Specified, Thin Prep

Final Diagnosis

Cytology, GYN, Site Not Specified, Thin Prep

NEGATIVE FOR INTRAEPITHELIAL LESION OR MALIGNANCY.
Reactive cellular changes.

SPECIMEN ADEQUACY:
Satisfactory for evaluation.
Transformation zone components are present.

Specimen Class: Status: Signed Out Accessioned
Signed Out:

Specimen(s) Received: Cervical/Endocervical, Thin Prep

Final Diagnosis

Cervical/Endocervical, Thin Prep

NEGATIVE FOR INTRAEPITHELIAL LESION OR MALIGNANCY.

SPECIMEN ADEQUACY:
Satisfactory for evaluation.
Transformation zone components are present.

Specimen Class: **Status:** Signed Out **Accessioned**
Signed Out:

Specimen(s) Received: Vaginal/Cervical/Endocervical, Direct

Final Diagnosis

Vaginal/Cervical/Endocervical, Direct
CELLULAR CHANGES WITHIN NORMAL LIMITS.
Endocervical cells present.

SPECIMEN ADEQUACY:
Satisfactory for evaluation but limited by obscuring white blood cells.

Specimen Class: **Status:** N/A **Accessioned:**
Signed Out:

Specimen(s) Received: Skin, biopsy, left chin

Final Diagnosis **{Final Report Not Signed Out}**

{Not Entered}

Specimen Class: **Status:** Signed Out **Accessioned**
Signed Out:

Specimen(s) Received: Cervical, Direct

Final Diagnosis

Cervical, Direct

CELLULAR CHANGES WITHIN NORMAL LIMITS.
Inflammation.

SPECIMEN ADEQUACY:
Satisfactory for evaluation. Endocervical cells present.

Specimen Class: **Status:** Signed Out **Accessioned:**
Signed Out:

Specimen(s) Received: Endometrium, biopsy
Final Diagnosis

Endometrium, biopsy:
Benign dyssynchronous secretory endometrium; no hyperplasia or carcinoma identified.

Specimen Class: **Status:** Signed Out **Accessioned:**
Signed Out: (

Specimen(s) Received: Cervical/Endocervical, Direct
Final Diagnosis

Cervical/Endocervical, Direct
CELLULAR CHANGES WITHIN NORMAL LIMITS.
Inflammation.
Endocervical cells present.

SPECIMEN ADEQUACY:
Satisfactory for evaluation but limited by obscuring white blood cells.

Specimen Class: **Status:** Signed Out **Accessioned**
Signed Out:

Specimen(s) Received: Cervical/Endocervical, Direct
Final Diagnosis

Cervical/Endocervical, Direct
CELLULAR CHANGES WITHIN NORMAL LIMITS.

SPECIMEN ADEQUACY:
Satisfactory for evaluation. Endocervical cells present.

Specimen Class: **Status:** Signed Out **Accessioned**
Signed Out:

Specimen(s) Received: Bladder Washing
Final Diagnosis

Bladder Washing
BENIGN.

Specimen Class: Status: Signed Out Accessioned:
Signed Out:

Specimen(s) Received: Urine, catheterized

Final Diagnosis

BENIGN.

See Below.

Specimen Class: Status: Signed Out Accessioned
{HighRisk Specimen} Signed Out:

Specimen(s) Received: Vaginal/Cervical/Endocervical

Final Diagnosis

CELLULAR CHANGES WITHIN NORMAL LIMITS.

Squamous metaplasia.

SPECIMEN ADEQUACY:

Satisfactory for evaluation. Endocervical cells present.

Specimen Class: Status: Signed Out Accessioned
Signed Out:

Specimen(s) Received: BLADDER WASH

Final Diagnosis

BENIGN

Conversion

Specimen Class: Status: Signed Out Accessioned:
Signed Out:

Specimen(s) Received: BLADDER WASH

Final Diagnosis

BENIGN

Conversion

Specimen Class: Status: Signed Out Accessioned:
Signed Out:

Specimen(s) Received: BLADDER WASH

Final Diagnosis

BENIGN

Conversion

Specimen Class: Status: Signed Out Accessioned:
Signed Out:

Specimen(s) Received: VAGINAL/CERVICAL/ENDOCERVICAL
Final Diagnosis
BETHESDA: CELLULAR CHANGES WITHIN NORMAL LIMITS
BENIGN
Inflammation

Conversion

Specimen Class: Status: Signed Out Accessioned:
Signed Out:

Specimen(s) Received: URINE, CATHETERIZED
Final Diagnosis
BENIGN

Conversion

Specimen Class: Status: Signed Out Accessioned:
Signed Out:

Specimen(s) Received: VAGINAL/CERVICAL/ENDOCERVICAL
Final Diagnosis
DESCRIPTIVE DIAGNOSIS
Diagnosis Deferred

Conversion

Specimen Class: Status: Signed Out Accessioned:
Signed Out:

Specimen(s) Received: SITE NOT SPECIFIED
Final Diagnosis
DESCRIPTIVE DIAGNOSIS
Diagnosis Deferred
Inflammation

Conversion

Specimen Class: Status: Signed Out Accessioned
Signed Out:

Specimen(s) Received: VAGINAL/CERVICAL/ENDOCERVICAL
Final Diagnosis
BENIGN

Conversion

END OF REPORT

Criteria	Yes	No
Diagnosis Discrepancy		✓
Primary Tumor Site Discrepancy		✓
HIPAA Discrepancy		✓
Prior Malignancy History		✓
Dual/Synchronous Primary Noted		✓
Case is (circle): QUALIFIED / DISQUALIFIED		
Reviewer Initials RB Date Reviewed: 9/29/11		

lw 10/21/11

UUID:4F047F5B-EF3A-4590-8E7A-D90A52860324
TCGA-A1-A0SM-01A-PR Redacted

ICD-O-3
carcinoma, infiltrating duct, NOS 8500/3
Site: breast, NOS C50.9 lw 10/21/11

Final Pathologic Diagnosis:

A. Left axillary sentinel lymph node #1, biopsy: No tumor in one lymph node (0/1).

B. Left axillary sentinel lymph node #2, biopsy: No tumor in one lymph node (0/1).

C. Left axillary minor sentinel lymph node #3, biopsy: No tumor in one lymph node (0/1).

D. Left breast, mastectomy:
1. Invasive ductal carcinoma, 3.5 cm, grade 2, associated with microcalcifications; see comment.
2. Ductal carcinoma in situ, cribriform, intermediate grade.
3. Gynecomastia.

Note:
Breast Tumor Synoptic Comment
- Laterality: Left.
- Invasive tumor type: Invasive ductal carcinoma.
- Invasive tumor size: 3.5 cm maximum diameter.
- Invasive tumor grade (modified Bloom-Richardson): Grade 2.
  Nuclear grade: 3 = 3 points.
  Mitotic count: <10 mitotic figures/10 HPF = 1 point.
  Tubule/papilla formation: Definite tubule formation in <10% = 3 points.
  Total points and overall grade: 7 points = grade 2.
- Lymphatic-vascular invasion: None.
- Perineural invasion: None.
- Resection margins for invasive tumor:
  - Deep margin: Negative; (tumor is 0.3 cm away, on slide D4).

- Medial margin: Negative; (tumor is >1 cm away).
- Lateral margin: Negative; (tumor is >1 cm away).
- Anterior/superior margin: Negative; (tumor is >1 cm away).
- Anterior/inferior margin: Negative; (tumor is 0.8 cm away, on slide D3).

- Ductal carcinoma in situ (DCIS) type: Cribriform.
- Ductal carcinoma in situ size: 0.3 cm.
- Ductal carcinoma in situ nuclear grade: Intermediate nuclear grade.
- Necrosis in DCIS: None.
- Microcalcifications: Present in invasive carcinoma.
- Resection margins for ductal carcinoma in situ:
  - Deep margin: Negative; (tumor is >1 cm away).
  - Medial margin: Negative; (tumor is >1 cm away).
  - Lateral margin: Negative; (tumor is >1 cm away).
  - Anterior/superior margin: Negative; (tumor is >1 cm away).
  - Anterior/inferior margin: Negative; (tumor is >1 cm away).

- Lymph node status: Negative (0/3).

- AJCC/UICC stage: pT2N0MX.

- Nontumorous breast tissue: Gynecomastia.
- Nipple: No tumor.
- Skin/dermis: No tumor.

- Additional comments: Each sentinel lymph node was examined with level sections. No metastatic carcinoma was identified.

[redacted] has reviewed selected slides and concurs with the findings.

Intraoperative Consult Diagnosis

FS1 (A) Sentinel lymph node #1, left axilla, biopsy: No carcinoma. Cytologic preparations and frozen section. ([redacted],

FS2 (B) Sentinel lymph node #2, left axilla, biopsy: No carcinoma. Cytologic preparations and frozen section. ([redacted]

Clinical History

The patient is a -year-old with invasive ductal carcinoma of the left breast. This diagnosis was established by core biopsy at another institution. That biopsy showed that the carcinoma is ER, PR, and HER-2-positive.

Gross Description

The specimen is received fresh in four parts, each labeled with the patient's name and unit number.

Part A, labeled " consists of a single soft irregular red-yellow candidate lymph node measuring 2.7 x 1.8 x 1.3 cm. Extraneous fatty tissue is removed. The candidate lymph node is bisected. Touch and scrape preparations are made. The remaining lymph node is submitted for frozen section, and subsequently submitted in cassette A1. The unused fatty tissue is entirely submitted in cassette A2.

Part B, labeled " nsists of a single soft irregular red-yellow candidate lymph node measuring 1.7 x 0.8 x 0.7 cm. Extraneous fatty tissue is removed. The lymph node is bisected. Touch and scrape preparations are made. The remaining lymph node is submitted for frozen section diagnosis 2, and subsequently submitted in cassette B1. The unused fatty tissue is entirely submitted in cassette B2.

Part C, additionally labeled " ," consists of a single fragment of yellow fibrofatty tissue measuring 2 x 1.5 x 0.5 cm. One candidate lymph node is palpated within the fatty tissue. The

---

specimen is entirely submitted in cassette C1.

Part D, additionally labeled consists of a mastectomy specimen oriented with a short suture superior and a long suture lateral. The specimen measures 14.2 cm from superior to inferior, 14.5 cm from medial to lateral and 3 cm from anterior to posterior. On the anterior surface, there is a skin ellipse measuring 12.4 x 4.6 cm. Within the skin ellipse is a nipple/areola measuring 2 cm in diameter. A firm mass is palpated deep and medial to the nipple in the inner upper and lower quadrants. The specimen is inked for microscopic evaluation, with the anterior superior inked in blue, the anterior inferior inked in green and the posterior inked in black. The specimen is then sectioned into fifteen slices, numbered from medial to lateral. The nipple-areolar complex appears in slices 5-7. Sectioning shows an irregular, lobulated, pink-tan mass measuring 3.5 cm; this is located in the inner lower quadrant deep to the nipple, in slices 5-7. The cyst mass extends to within 0.7 cm of the deep margin, 1.7 cm from the anterior superior margin, and 1.9 cm from the anterior inferior margin. The remainder of the breast parenchyma consists of yellow fatty tissue and is unremarkable. Cassettes are submitted as follows:

Cassettes D1-D2:	Nipple.
Cassettes D3-D4:	Mass, slice 5.
Cassettes D5-D7:	Mass, slice 6 (widest cross-section superior to inferior).
Cassette D8:	Representative medial margin.
Cassette D9:	Representative lateral margin.
Cassette D10:	Representative unremarkable inner upper quadrant, slice 2.
Cassette D11:	Representative unremarkable inner upper quadrant, slice 4.
Cassette D12:	Representative unremarkable inner lower quadrant, slice 3.
Cassette D13:	Representative unremarkable inner lower quadrant, slice 4.
Cassette D14:	Representative unremarkable outer upper quadrant, slice 9.
Cassette D15:	Representative unremarkable outer upper quadrant, slice 12.
Cassette D16:	Representative unremarkable outer lower quadrant, slice 10.
Cassette D17:	Representative unremarkable outer lower quadrant, slice 13.

[redacted]/Pathology Resident

[redacted]/Pathologist
Signed:

Fee Codes:

Addenda

Addendum.

Date Ordered:
Date Complete:
Date Reported:

Status: Signed Out
By:

Addendum Comment

An immunohistochemical test for estrogen and progesterone receptors as well as for HER2 was performed on block D6.

The test for estrogen receptors is positive. There is variable nuclear staining (ranging from weak to strong) in ~20% of tumor cells.

The test for progesterone receptors is negative. There is no nuclear staining in any of tumor cells. Internal positive control is present.

Result of HER2/neu test: This carcinoma is positive for HER2/neu oncoprotein over-expression.

An immunohistochemical assay was performed using the CB11 monoclonal antibody to HER2/neu oncoprotein. The staining intensity of this carcinoma was 3 on a scale of 0-3.

Carcinomas with staining intensity scores of *0 or 1* are considered *negative* for over-expression of HER2/neu oncoprotein.

Those with a staining intensity score of *2* are considered *indeterminate*. We and others have observed that many carcinomas with staining intensity scores of 2 do not show gene amplification. All carcinomas with staining intensity scores of 2 are therefore submitted for FISH testing. The results of the FISH test are issued directly from the molecular cytogenetics laboratory.

Carcinomas with staining intensity scores of *3* are considered *positive* for over-expression of HER2/neu oncoprotein. Tumors in this category show an excellent correlation between the results of immunohistochemical and FISH testing, and almost always show gene amplification.

The immunoperoxidase stain(s) reported above were developed and their performance characteristics determined by the They have not been cleared or approved by the U. S. Food and Drug Administration. The FDA has determined that such clearance or approval is not necessary. These tests are used for clinical purposes. They should not be regarded as investigational or for research. This laboratory is certified under the Clinical Laboratory Improvement Amendments of 1988 ("CLIA") as qualified to perform high-complexity clinical testing.

[redacted]/Pathologist

Electronically signed out on

[redacted]

Specimen Class: Status: Signed Out Accessione

END OF REPORT

Criteria	Yes	No
Diagnosis Discrepancy		✓
Primary Tumor Site Discrepancy		✓
HIPAA Discrepancy		✓
Prior Malignancy History		✓
Dual/Synchronous Primary Noted		✓
Case is (circle):	QUALIFIED	DISQUALIFIED
Reviewer Initials RB	Date Reviewed: 9/29/11	

10/21/11

UUID:D0269758-EFAE-4EBA-8CCF-4A6CF4D4B35A
TCGA-A1-A0SN-01A-PR Redacted

ICD-0-3
carcinoma, infiltrating duct, NOS 8500/3
Site: breast, NOS C50.9 lw 10/21/11

Final Pathologic Diagnosis:

A. Lymph node, left axillary sentinel node #1, excision: No metastatic carcinoma in one lymph node (0/1).

B. Lymph node, left axillary sentinel node #2, excision: Metastatic carcinoma in one lymph node with extension into peri-nodal fat (1/1).

C. Lymph node, left axillary sentinel node #3, excision: Metastatic carcinoma in one lymph node (1/1).

D. Lymph node, left axillary non-sentinel node #1, excision: Metastatic carcinoma in one lymph node (1/1).

E. Lymph node, left axillary non-sentinel node #2, excision: Metastatic carcinoma in one lymph node (1/1).

F. Breast, left, partial mastectomy:
1. Infiltrative ductal carcinoma, SBR grade 2, 1.1 cm in maximum dimension, margins negative; see comment.
2. Ductal carcinoma in situ, high-grade, solid and comedo types, 1.1 cm in maximum dimension, intermixed with invasive ductal carcinoma, margins negative.

G. Breast, left lateral anterior, excision: Benign breast tissue, no carcinoma identified.

H. Breast, medial superior anterior, excision: Benign breast tissue, no carcinoma identified.

I. Lymph node, left axillary contents, excision: No metastatic carcinoma in eight lymph nodes (0/8).

Note: *Breast Tumor Synoptic Comment*
- Laterality: Left.
- Invasive tumor type: Invasive ductal carcinoma.
- Invasive tumor size: 1.1 cm maximum diameter.
- Invasive tumor grade (modified Bloom-Richardson): 2.
    Nuclear grade: 3, 3 points.
    Mitotic count: <10 mitotic figures/10 HPF, 1 point.
    Tubule/papilla formation: <10%, 3 points.
    Total points and overall grade = 7 points = grade 2.
- Lymphatic-vascular invasion: Extensive lymphatic-vascular invasion is noted with intra-lymphatic metastasis noted in lymphatics at least 1 cm from the main tumor.
- Perineural invasion: Not present.
- Resection margins for invasive tumor:
    - Deep margin: Negative; (tumor is 0.2 cm away, on slide F6).
    - Medial margin: Negative; (tumor is greater than 1 cm).
    - Lateral margin: Negative; (tumor is greater than 1 cm).
    - Anterior/superior margin: Negative; (tumor is 0.2 cm away, on slide F6).
    - Anterior/inferior margin: Negative; (tumor is 0.3 cm away, on slide F4).

- Ductal carcinoma in situ (DCIS) type: Comedo and solid.
- Ductal carcinoma in situ size: 1.1 cm, intermixed with invasive ductal carcinoma.
- Ductal carcinoma in situ nuclear grade: High-grade.
- Necrosis in DCIS: Comedonecrosis.
- Microcalcifications: Not identified.
- Resection margins for ductal carcinoma in situ:
    - Deep margin: Negative; (tumor is 0.6 cm away, on slide F6).
    - Medial margin: Negative; (tumor is greater than 1 cm).
    - Lateral margin: Negative; (tumor is greater than 1 cm).
    - Anterior/superior margin: Negative; (tumor is 0.25 cm away, on slide F6).
    - Anterior/inferior margin: Negative; (tumor is 0.25 cm away, on slide F6).

- Lobular carcinoma in situ (LCIS): None.

- Lymph node status: Positive.
    - Number of positive lymph nodes: 4.
    - Total number sampled: 13.
- Diameter of largest metastasis: 0.8 cm.

- Extranodal extension: Present.
- AJCC/UICC stage: pT1cN1MX.

An immunohistochemical test for estrogen and progesterone receptors was performed by manual morphometry on block #.

The test for estrogen receptors is positive. There isstrong nuclear staining in 90% of tumor cells. Internal positive control is positive.

The test for progesterone receptors is positive. There is moderate nuclear staining in 60% of tumor cells. Internal positive control is positive.

Result of HER2/neu test: This carcinoma is positive for HER2/neu oncoprotein over-expression.

An immunohistochemical assay was performed by manual morphometry on block F8 using the CB11 monoclonal antibody to HER2/neu oncoprotein. The staining intensity of this carcinoma was 3 on a scale of 0-3 (HER2 test interpreted by Dr.

Carcinomas with staining intensity scores of *0 or 1* are considered *negative* for over-expression of HER2/neu oncoprotein.

Those with a staining intensity score of *2* are considered *indeterminate*. We and others have observed that many carcinomas with staining intensity scores of 2 do not show gene amplification. All carcinomas with staining intensity scores of 2 are therefore submitted for FISH testing. The results of the FISH test are issued directly from the molecular cytogenetics laboratory.

Carcinomas with staining intensity scores of *3* are considered *positive* for over-expression of HER2/neu oncoprotein. Tumors in this category show an excellent correlation between the results of immunohistochemical and FISH testing, and almost always show gene amplification.

Intraoperative Consult Diagnosis

FS1 (A) Left axillary sentinel lymph node #1, biopsy: No tumor seen. (Tissue section and cytopreparation) (Dr.

FS2 (B) Left axillary sentinel lymph node #2, biopsy: Positive for metastatic carcinoma. (Tissue section and cytopreparation) (Dr.

FS3 (C) Left axillary sentinel lymph node #3, biopsy: Positive for metastatic carcinoma. (Tissue section and cytopreparation) (Dr.

FS4 (D) Left axillary non-sentinel lymph node #1, biopsy: Positive for metastatic carcinoma. (Tissue section and cytopreparation) (Dr.

Clinical History

The patient is a year-old woman with left breast invasive ductal carcinoma. She undergoes partial mastectomy.

Gross Description

The specimen is received fresh in nine parts, each labeled with the patient's name and medical record number.

Part A is additionally labeled It consists of a single irregular piece of soft-firm, pink-red tissue, measuring 0.5 x 0.3 x 0.2 cm. The specimen is bisected, and cytologic touch and scrape preparations are prepared. The specimen is then entirely submitted for frozen section diagnosis as FS1, with the frozen section remnant submitted in cassette A1.

Part B is additionally labeled It consists of a single oval piece of soft-to-firm, red-pink tissue, measuring 0.9 x 0.6 x 0.6 cm. The specimen is bisected, and cytologic touch and scrape preparations are made. The specimen is then entirely submitted for frozen section diagnosis as FS2, with the frozen section remnant submitted in cassette B1.

Part C is additionally labeled It consists of a single ovoid piece of soft-to-firm,

pink-red tissue, measuring 0.8 x 0.5 x 0.4 cm. The specimen is bisected, and cytologic touch and scrape preparations are prepared. The specimen is then entirely submitted for frozen section diagnosis as FS3, with the frozen section remnant submitted in cassette C1.

Part D is additionally labeled It consists of a single ovoid piece of soft-to-firm, pink-red tissue, measuring 1.3 x 0.7 x 0.5 cm. The specimen is bisected, and cytologic touch and scrape preparations are prepared. The specimen is then entirely submitted for frozen section diagnosis as FS4, with the frozen section remnant submitted in cassette D1.

Part E is additionally labeled It consists of a single ovoid piece of firm, off-white/tan tissue, measuring 1.5 x 0.7 x 0.4 cm. The specimen is bisected and entirely submitted in cassette E1.

Part F is additionally labeled t consists of an oriented portion of fat, measuring 10.3 (anterior- posterior) x 7.4 (medial-lateral) x 6.4 (superior-inferior) cm and weighing 13 gm. There is a 1.1 cm in diameter white-yellow hard mass with a white homogeneous interior located within the specimen (slices 3-6). This mass abuts the posterior margin, is 0.6 cm from the anterior-inferior and anterior-superior margins, 1 cm from the lateral margin, and 7 cm from the medial margin. The specimen is inked so as the anterior-superior surface is blue, the anterior- inferior surface is green, and the posterior surface is black. A representative section is taken for tissue banking. The specimen is serially sectioned, from lateral to medial into thirteen 0.5 cm slices. Representative sections are submitted as follows:

Cassette F1:	Lateral margin, slice 1, perpendicular.
Cassettes F2-F3:	Slice 2, two pieces.
Cassettes F4-F5:	Slice 3, two pieces, mass.
Cassettes F6-F7:	Slice 4, two pieces, mass.
Cassette F8:	Representative section of slice 5, mass.
Cassette F9:	Representative section of slice 6, mass.
Cassette F10:	Slice 12.
Cassette F11:	Medial margin, slice 13, perpendicular.

Part G is additionally labeled ." It consists of a small fragment of fat, measuring 0.6 x 0.7 x 0.7 cm. A stitch marks a portion of the specimen indicating the true margin. The specimen is inked so as this true margin is blue, and the rest of the specimen is black. The specimen is entirely submitted in cassette G1.

Part H is additionally labeled It consists of a fragment of yellow-white, soft fat, measuring 1 x 0.6 x 0.7 cm. A stitch marks the true margin of the specimen. The specimen is inked so as this true margin is blue, and the remaining portion of the specimen is black. The specimen is entirely submitted in cassette H1.

Part I is additionally labeled It consists of a portion of fat measuring 4.2 x 3.8 x 0.9 cm. Multiple pink-tan lymph nodes are noted within the specimen ranging in size from 0.6-1.3 cm in diameter. Many of these nodes are previously bisected or trisected. Representative sections of the specimen are taken and submitted as follows:

Cassette I1:	Six lymph nodes attached.
Cassette I2:	Two lymph nodes, one bisected and one trisected.

'Pathology Resident

›athologist

Signed:

Fee Codes:

Other Specimens

Specimen Class: | **Status:** N/A | **Accessioned:**
Signed Out:

Specimen(s) Received: Skin, biopsy, punch bx, right posterior calf

Final Diagnosis {Final Report Not Signed Out}

{Not Entered}

Specimen Class: **Status:** Signed Out **Accessioned:**
Signed Out:

Specimen(s) Received: SP Consult

Final Diagnosis

Review from

Breast, left, 1 o'clock, core needle biopsy:

1. Invasive carcinoma; see comment.
2. Microscopic focus of ductal carcinoma in situ, high nuclear grade; see comment.

END OF REPORT

ICD-O-3 Carcinoma, infiltrating ductal, NOS 8500/3
Site: breast, NOS C50.9 1/27/11

Name: [redacted]		Age/Sex:	/F	Location: [redacted]	
Acct#: [redacted]	Unit#: [redacted]	Status:	DIS IN	Room/Bed: [redacted]	
Reg:	Disch:	Att Dr:			

This report contains corrections, additions or deletions.
Any previous versions are stored internally and are available if necessary.

**Specimen:**	**Received:**	**Status:**	**Req#:** [redacted]
	**Spec Type:** SURGICAL P	**Subm Dr:**	

PREOPERATIVE DIAGNOSIS

LEFT BREAST CANCER INVASIVE, BREAST ASYMMETRY

OPERATION PERFORMED

DATE:
DOCTOR(S):
PROCEDURE: LEFT SIMPLE MASTECTOMY, SENTINEL NODE BX, REDUCTION MAMMOPLASTY
PROCEDURE(CONT): BREAST RECONSTRUCTIN WITH TISSUE EXPANDER

TISSUE REMOVED

A. LEFT BREAST MASTECTOMY
B. LT BREAST 1ST SENTINEL NODE
C. LT BREAST 2ND SENTINEL NODE
D. LT BREAST 3RD SENTINEL NODE
E. LEFT BREAST TISSUE
F. RIGHT BREAST TISSUE

UUID:BC75790C-FBC7-4897-B228-762245C48740
TCGA-A2-A0SU-01A-PR Redacted

GROSS DESCRIPTION

PART A RECEIVED FRESH LABELED [redacted] LEFT BREAST MASTECTOMY STITCH AT SUPERIOR MEDIAL BORDER, IS A LEFT SIMPLE MASTECTOMY SPECIMEN AS ORIENTED BY A SUTURE MEASURING 21 X 20 X 4.5 CM. A SKIN ELLIPSE MEASURES 11.2 X 6.5 CM, WITH A GROSSLY UNREMARKABLE NIPPLE. THE SUPERFICIAL ASPECT IS MARKED IN BLUE INK, THE DEEP ASPECT IN BLACK INK. IN THE MIDPORTION, THERE IS A 0.9 X 0.7 X 0.9-CM LESION 2.5 CM FROM THE DEEP MARGIN. A PORTION OF THIS IS SUBMITTED PER PROTOCOL, THE MIRROR IMAGE IS SUBMITTED IN A1, AND THE CORRESPONDING DEEP MARGIN IN A2. A SECOND LARGER LESION IS FOUND LATERAL TO THIS MEASURING 2.2 X 1.6 X 1.5 CM. THIS IS 2 CM FROM THE DEEP MARGIN. THIS DEEP MARGIN IS SUBMITTED IN A3. THESE TWO AREAS ARE 1.5 CM APART. MEDIAL TO ALL OF THESE IS A SMALL SUPERFICIAL LESION, 0.5 CM IN GREATEST DIMENSION, 2.5 CM FROM THE FIRST DESCRIBED LESION. THIS LESION IS SUBMITTED IN A6. A5 REPRESENTS A SECOND SECTION OF THE LARGEST MOST LATERAL LESION. THE SMALLEST LESION IS 4 CM FROM THE DEEP MARGIN, WHICH IS SUBMITTED IN A7. THE REMAINING BREAST TISSUE CONSISTS OF BLAND YELLOW FATTY TISSUE WITH FINE FIBROUS BANDS. ADDITIONAL SECTIONS OF THE BREAST TISSUE ARE SUBMITTED AS FOLLOWS: A8--UPPER OUTER QUADRANT, A9--UPPER INNER QUADRANT, A10--LOWER INNER QUADRANT, A11--LOWER OUTER QUADRANT, A12--NIPPLE. THE LARGEST LESION IS SUPERIOR TO THE NIPPLE, AND

[illegible]

Patient: [redacted] [redacted] (Continued)

Specimen: **Received:** **Status:** **Req#** [redacted]

Spec Type: SURGICAL P **Subm Dr:**

GROSS DESCRIPTION (Continued)

THESE LESIONS PROGRESS THEREFORE IN THE UPPER INNER QUADRANT OF THE BREAST.

PART B RECEIVED FRESH LABELED [redacted] LEFT BREAST FIRST SENTINEL NODE HOT, IS A PORTION OF YELLOW FATTY TISSUE MEASURING 2.2 X 1.6 X 0.1 CM. SECTIONING REVEALS A 0.7-CM LYMPH NODE. ONE-HALF IS SUBMITTED PER PROTOCOL; THE REMAINDER IS SUBMITTED LABELED B.

PART C RECEIVED FRESH LABELED [redacted] LEFT BREAST SECOND SENTINEL NODE HOT, IS YELLOW FATTY TISSUE MEASURING 3.8 X 1.1 X 0.8 CM. EXAMINATION REVEALS A 2.2 CM IN LENGTH GROSSLY UNREMARKABLE LYMPH NODE, A PORTION OF WHICH IS SUBMITTED PER PROTOCOL, AND THE REMAINDER IS SUBMITTED LABELED C.

PART D RECEIVED FRESH LABELED [redacted] THIRD SENTINEL NODE, IS AN OVOID PORTION OF YELLOW FATTY TISSUE MEASURING 2.5 X 1.2 X 1.0 CM. SECTIONING REVEALS FIRM TISSUE BUT NO GROSS NODAL TISSUE. A PORTION IS SUBMITTED PER PROTOCOL, AND THE REMAINDER IS SUBMITTED LABELED D.

PART E RECEIVED LABELED [redacted] SECOND BREAST TISSUE SUPERFICIALLY OVER SUPERIOR MEDIAL ASPECT OVER HARD NODULE STITCH AT NEW MARGIN, IS AN IRREGULAR PORTION OF YELLOW FATTY TISSUE MEASURING 2.9 X 2.0 X 0.5 CM. A SUTURE DENOTES A NEW MARGIN, AND THIS SIDE IS MARKED WITH INK. THIS IS SECTIONED AND SUBMITTED LABELED E.

PART F RECEIVED LABELED [redacted] RIGHT BREAST TISSUE, IS 256 GRAMS OF FIBROFATTY BREAST TISSUE AND SKIN. SECTIONING REVEALS BLAND YELLOW FATTY TISSUE WITH FINE FIBROUS BANDS. THERE ARE NO AREAS OF BROAD FIBROSIS OR MASSES IDENTIFIED. REPRESENTATIVE TISSUE IS SUBMITTED LABELED F1 THROUGH 5.

PATH PROCEDURES

PROCEDURES:

88305, 88307/5, IMMUNOPEROXIDAS/3, A1 BLK, A10 BLK, A11 BLK, A12 BLK, A2 BLK, A3 BLK, A4 BLK, A5 BLK, A6 BLK, A7 BLK, A8 BLK, A9 BLK, BBX X6, CBX X6, DBX X6, E1 BLK, F1 BLK, F2 BLK, F3 BLK, F4 BLK, F5 BLK

FINAL DIAGNOSIS

PART A LEFT BREAST, SIMPLE MASTECTOMY:

1. MULTICENTRIC MODERATELY DIFFERENTIATED INTRADUCTAL AND INFILTRATING DUCT CARCINOMAS (3), NUCLEAR GRADE 2 WITH MODERATE MITOTIC INDEX WITH AN INSITU COMPONENT OF 5-10% OF SOLID AND CRIBRIFORM TYPE

Patient: [redacted] [redacted] (Continued)

Specimen: | Received: | Status: | Req#: [redacted]
Spec Type: SURGICAL P | Subm Dr:

FINAL DIAGNOSIS (Continued)

2. THREE TUMORS MEASURING 2.2, 1.0 AND 1.0 CM ARE PRESENT WITH ONE TUMOR INVOLVING THE SUPERFICIAL MARGIN.

3. DUCTAL CARCINOMA IN SITU IS PRESENT IN THE DEEP DUCTS OF THE NIPPLE SKIN. THE NIPPLE SKIN AND DEEP MARGINS OF EXCISION ARE FREE OF NEOPLASM.

4. FOCI SUSPICIOUS FOR LYMPHATIC INVASION ARE PRESENT.

PART B LEFT BREAST, FIRST SENTINEL NODE, BIOPSY: LYMPH NODE (1) WITH AN ISOLATED FOCUS OF TUMOR CELLS, MEASURING 0.01 MM, INDENTIFIED ON THE CYTOKERATIN STAIN AND ONLY THE FIRST OF THREE STEP-SECTIONS OF THE H&E STAINS.

PART C LEFT BREAST, SECOND SENTINEL NODE, BIOPSY: LYMPH NODE (1), NEGATIVE FOR TUMOR BY ROUTINE AND CYTOKERATIN STAIN.

PART D LEFT BREAST, THIRD SENTINEL NODE, BIOPSY: SMALL LYMPH NODES (5), NEGATIVE FOR TUMOR BY ROUTINE AND CYTOKERATIN STAIN.

PART E LEFT BREAST SUPERFICIAL TISSUE RE-EXCSION: FIBROADIPOSE TISSUE, NEGATIVE FOR TUMOR WITH THE NEW INKED MARGIN FREE OF NEOPLASM.

PART F RIGHT BREAST, REDUCTION MAMMOPLASTY: SKIN, ADIPOSE AND BREAST TISSUE WITH FIBROCYSTIC CHANGE AND A FOCUS OF ATYPICAL LOBULAR HYPERPLASIA.

CONSULTATION DIAGNOSIS

ONCOTYPE

RESULTS: = 14

CLINICAL EXPERIENCE: PATIENTS WITH A RECURRENCE SCORE OF 14 IN THE CLINICAL VALIDATIONS STUDY HAD AN AVERAGE RATE OF DISTANT RECURRENCE AT 10 YEARS OF 9%(95% CI: 6%-12%).

Signed ______(signature on file)_______

Criteria	Yes	No
Diagnosis Discrepancy		✓
Primary Tumor Site Discrepancy		✓
HIPAA Discrepancy		✓
Prior Malignancy History		✓
Dual/Synchronous Primary Noted		✓
Case is (circle): QUALIFIED / DISQUALIFIED		
Reviewer Initials [illegible] Date Reviewed: [illegible]		

[redacted]

Specimen: **Received:** **Status:** [redacted]
Spec Type: SURGICAL P **Subm Dr:**

[redacted]

BREAST CANCER

ICD-0-3
Carcinoma, infiltrating duct, NOS 8500/3
Site: breast, NOS C50.9 [illegible] 1/27/11

[redacted]

DATE:
DOCTOR(S):

[redacted]

UUID:7B1E6EEB-3B61-4CE8-B695-ED11C2EF169F
TCGA-A2-A0T5-01A-PR Redacted

A. LEFT BREAST SUPERFICIAL MARGIN RE-EXCISION
B. RIGHT BREAST
C. LEFT BREAST

[redacted]

PART A RECEIVED LABELED [redacted], LEFT BREAST UPPER OUTER QUADRANT SUPERFICIAL MARGIN REEXCISION STITCH NEW MARGIN, IS AN IRREGULAR PORTION OF YELLOW-PINK FATTY TISSUE MEASURING 12.5 X 7.5 X 1.0 CM IN GREATEST DIMENSIONS. A SUTURE DENOTES THE NEW MARGIN. THIS SIDE IS MARKED WITH BLUE INK WITH A PERIMETER OF BLACK INK. REPRESENTATIVE SECTIONS TO INCLUDE APPROXIMATELY 40% OF THE SPECIMEN ARE SUBMITTED LABELED A1 THROUGH 10.

PART B RECEIVED LABELED [redacted] RIGHT BREAST STITCH AT 12 O'CLOCK, IS A SIMPLE MASTECTOMY SPECIMEN MEASURING 24 X 18.5 X 5.5 CM. THE NIPPLE IS GROSSLY UNREMARKABLE WITHIN A 7 X 3.5-CM SKIN ELLIPSE. A SUTURE DENOTES 12 O'CLOCK. THE SUPERFICIAL ASPECT IS MARKED WITH BLUE INK, THE DEEP WITH BLACK. SECTIONING REVEALS DENSE GRAY-TAN FIBROUS TISSUE INTERMIXED WITH YELLOW FATTY TISSUE. THE FIBROUS TISSUE IS PRIMARILY IN THE MID TO LOWER OUTER QUADRANT AREAS. THIS IS SURROUNDED BY BLAND YELLOW FATTY TISSUE. THE FIBROUS TISSUE COMPRISES UP TO 40% OF THE SPECIMEN. SECTIONS ARE SUBMITTED AS FOLLOWS: B1--NIPPLE, B2--CENTRAL DEEP MARGIN, B3 AND 4--UPPER INNER QUADRANT, B5 AND 6--UPPER OUTER QUADRANT, B7 AND 8--LOWER OUTER QUADRANT, B9 AND 10--LOWER INNER QUADRANT.

PART C RECEIVED FRESH LABELED [redacted] LEFT BREAST STITCH AT 12 O'CLOCK, IS A 26 X 18 X 6-CM SIMPLE MASTECTOMY SPECIMEN. THE NIPPLE IS UNREMARKABLE WITHIN AN 8 X 4.2-CM SKIN ELLIPSE. A SUTURE DENOTES 12 O'CLOCK. THE SUPERFICIAL ASPECT IS MARKED WITH BLUE INK, THE DEEP WITH BLACK. SECTIONING REVEALS A PINK-TAN FIRM GRITTY MASS AT 3 O'CLOCK LATERAL BREAST. THIS AREA MEASURES 4.5 X 2.4 X 4.0 CM IN GREATEST DIMENSIONS. THE TISSUE BETWEEN THIS LESION AND THE DEEP MARGIN CONSISTS OF YELLOW FATTY TISSUE AND PINK-TAN FIBROUS TISSUE. THE MARGIN GROSSLY IS 0.8 CM AWAY. THIS IS 0.8 CM FROM THE PROXIMAL MARGIN. THERE IS A SECOND PALPABLE AREA OF FIRMNESS IN THE UPPER OUTER QUADRANT AT THE LATERAL EDGE OF THE SPECIMEN WHICH WHEN SECTIONED REVEALS NO GROSS LESION. BY PALPATION, THIS AREA IS 2 CM IN GREATEST DIMENSION. THIS AREA DOES DEMONSTRATE YELLOW-WHITE COLORATION SUGGESTIVE OF GROSS FAT

9 ↓ 5cm
2nd UOQ

Patient: [redacted] [redacted] (Continued)

Specimen **Received:** **Status:** [redacted]
Spec Type: SURGICAL P **Subm Dr:**

[redacted] (Continued)

NECROSIS. THIS AREA IS 5 CM FROM THE LESION. CENTRALLY, THE BREAST DOES DEMONSTRATE GRAY-TAN FIBROUS TISSUE. THE PERIMETER OF THE SPECIMEN IS BLAND YELLOW FATTY TISSUE. THE FIBROUS TISSUE COMPRISES NO MORE THAN 25% OF THE SPECIMEN. SECTIONS ARE SUBMITTED AS FOLLOWS: C1--NIPPLE AND SKIN (MIRROR IMAGE TO PROTOCOL), C2 AND 3--FULL CROSS-SECTION OF LESION (MIRROR IMAGE TO TISSUE TAKEN PER PROTOCOL), C4--LESION AND DEEP MARGIN, C5 AND 6--LESION AND SUPERFICIAL MARGIN, C7 AND 8--FULL CROSS-SECTION OF LESION (MIRROR IMAGE TO TISSUE TAKEN PER PROTOCOL), C9 AND 10--TISSUE EXTREME UPPER OUTER QUADRANT WITH INDURATION, C11--CENTRAL BREAST TISSUE WITH FIBROSIS, C12--UPPER OUTER QUADRANT, C13--UPPER INNER QUADRANT, C14--LOWER INNER QUADRANT, C15--LOWER OUTER QUADRANT.
(

[redacted]

PROCEDURES:
88307/3, A BLK/10, B BLK/10, C BLK/15

[redacted]

PART A LEFT BREAST UPPER OUTER QUADRANT SUPERFICIAL MARGIN REEXCISION: FIBROADIPOSE TISSUE WITH NO EVIDENCE OF NEOPLASM. THE NEW INKED MARGIN IS FREE OF NEOPLASM. FOCUS OF PREVIOUS BIOPSY SITE CHANGE WITH ORGANIZING FAT NECROSIS WITH REACTIVE FIBROSIS.

PART B RIGHT BREAST, SIMPLE MASTECTOMY: FIBROCYSTIC DISEASE WITH PATCHY FIBROSIS, APOCRINE METAPLASIA, CYST FORMATION AND A SMALL FIBROADENOMA. NO EVIDENCE OF ATYPIA OR MALIGNANCY. UNREMARKABLE NIPPLE SKIN. (R) FCC AM cyst FA

PART C LEFT BREAST, SIMPLE MASTECTOMY:

1. MODERATELY DIFFERENTIATED INTRADUCTAL AND INFILTRATING DUCT CARCINOMA, NUCLEAR GRADE II WITH LOW MITOTIC INDEX, WITH AN IN SITU COMPONENT OF APPROXIMATELY 10% OF SOLID AND CRIBRIFORM TYPE WITH FOCI OF INTRADUCTAL NECROSIS. (L) DCIS 10% solid/cribriform nec(+) IDC 2/2/1

2. THE TUMOR GROSSLY MEASURES 4.5 CM IN GREATEST DIMENSION. THE NIPPLE, DEEP AND SUPERFICIAL MARGINS ARE FREE OF NEOPLASM. 4.5cm skin/nipple(-)

3. FOCI SUSPICIOUS FOR LYMPHATIC INVASION ARE PRESENT. +LVI

4. BIOPSY SITE CHANGE WITH REACTIVE FIBROSIS AND ORGANIZING FAT NECROSIS INCLUDING AN AREA FROM THE UPPER OUTER QUADRANT WHICH MAY REPRESENT A PREVIOUS SENTINEL NODE BIOPSY SITE.

5. FIBROCYSTIC CHANGE WITH PATCHY DENSE FIBROSIS AND CYST FORMATION. FCC cyst

Patient: [redacted] [redacted] **(Continued)**

**Specimen:**	**Received:**	**Status:** [redacted]
	**Spec Type:** SURGICAL P	**Subm Dr:**

[redacted]

1

Signed ________________________________ (prelim.)

________(signature on file)________

Criteria	Yes	No
Diagnosis Discrepancy		✓
Primary Tumor Site Discrepancy		✓
HIPAA Discrepancy		✓
Prior Malignancy History		✓
Dual/Synchronous Primary Noted		✓
Case is (circle): QUALIFIED / DISQUALIFIED		
Reviewer Initials	Date Reviewed:	

Chief of Pathology

Phone
Fax 1

[redacted] [redacted]

Specimen: **Received:** **Status:** **Req#:** [redacted]
Spec Type: SURGICAL P **Subm Dr:**

PREOPERATIVE DIAGNOSIS

LEFT BREAST CANCER INVASIVE

ICD-0-3
Carcinoma, infiltrating duct, NOS 8500/3
Site: breast, NOS C50.9 1/27/11

OPERATION PERFORMED

DATE:
DOCTOR(S):
PROCEDURE: MASTECTOMY MODIFIED RADICAL

TISSUE REMOVED

UUID:90F13DE2-7C8F-4FA2-B9BF-D68EEC37D514
TCGA-A2-A0YJ-01A-PR Redacted

A. LEFT MODIFIED RADICAL MASTECTOMY
B. LT DEEP MARGIN BENEATH TUMOR

GROSS DESCRIPTION

PART A RECEIVED LABELED [redacted], LEFT MODIFIED RADICAL MASTECTOMY STITCH AT 12 O'CLOCK, IS A LEFT MODIFIED RADICAL MASTECTOMY WITH A SKIN ELLIPSE MEASURING 20 X 8.5 CM WITH A GROSSLY UNREMARKABLE NIPPLE. THE SPECIMEN ITSELF MEASURES 26.5 X 14 X 3.5 CM. THE AXILLARY CONTENTS ARE DISSECTED OFF THE SPECIMEN AND THIS AREA IS MARKED WITH RED INK DENOTING THAT THIS IS NOT A TRUE MARGIN. THE AXILLARY TAIL IS EXAMINED FOR LYMPH NODES. IN THE LOWER AXILLARY TAIL THERE IS A 3.7-CM IN GREATEST DIMENSION GROSSLY POSITIVE LYMPH NODE. ADDITIONAL NODES ARE ALSO IDENTIFIED. TWO ADDITIONAL GROSSLY POSITIVE NODES ARE SUBMITTED, ONE SECTION EACH, IN A1 AND A2 WITH MIRROR IMAGES PER PROTOCOL. A3 IS A GROSSLY UNREMARKABLE LYMPH NODE WITH MIRROR IMAGE IN PROTOCOL. A4--ONE SECTION OF A GROSSLY POSITIVE LYMPH NODE WITH MIRROR IMAGE PROTOCOL, A5--LARGEST LYMPH NODE, A6--TWO NODES, EACH BISECTED, A7--TWO NODES, EACH BISECTED, A8--NINE NODES. EIGHTEEN LYMPH NODES ARE IDENTIFIED IN ALL. THE SUPERFICIAL ASPECT OF THE BREAST IS THEN MARKED WITH BLUE INK, THE DEEP WITH BLACK INK. LATERAL TO THE NIPPLE THERE IS A PINK-TAN FIRM GRITTY MASS MEASURING 7 X 3 X 4.5 CM. THIS GROSSLY EXTENDS TO WITHIN 0.2 CM OF THE DEEP MARGIN. CENTRALLY IN THE BREAST THE TISSUE IS TAN AND FIBROUS. THE PERIMETER OF THE SPECIMEN IS FATTY WITH FINE FIBROUS BANDS. SECTIONS OF THE BREAST ARE SUBMITTED AS FOLLOWS: A9--NIPPLE AND SKIN (MIRROR IMAGE TO PROTOCOL), A10--SECTION OF TUMOR TO INCLUDE DEEP MARGIN (MIRROR IMAGE TO PROTOCOL), A11 THROUGH A16--A FULL CROSS SECTION FROM SUPERIOR TO INFERIOR OF THE LESION TO INCLUDE THE DEEP MARGIN IN A11, 12, 14, AND 16 WITH A13 AND A15 REPRESENTING THE SUPERFICIAL SECTIONS TO A12 AND A14, RESPECTIVELY, A17--TUMOR AND DEEP MARGIN, A18--THE MOST LATERAL ASPECT OF THE LESION, A19--THE MEDIAL ASPECT OF THE LESION (4.5 CM FROM PREVIOUS SECTION), A20--LOWER OUTER QUADRANT, A21--UPPER OUTER QUADRANT, A22--UPPER INNER QUADRANT 5 CM FROM TUMOR, A23-LOWER INNER QUADRANT 5 CM FROM TUMOR, A22 AND A23--MIRROR IMAGES TO PROTOCOL TISSUE.

PART B RECEIVED LABELED [redacted] DEEP MARGIN BENEATH TUMOR STITCH NEW MARGIN, IS AN IRREGULAR PORTION OF RED-TAN MUSCULAR TISSUE WITH CAUTERY ARTIFACT ON THE SIDE WITH THE SUTURE DENOTING THE NEW MARGIN. THIS MEASURES 2.7 X 2.5 X 0.4 CM IN GREATEST DIMENSIONS. THE

Chief of Pathology

Phone

Fax (

Patient: [redacted] [redacted] (Continued)

Specimen: **Received:** **Status:** **Req#:** [redacted]

Spec Type: SURGICAL P **Subm Dr:**

GROSS DESCRIPTION

(Continued)

SIDE DESIGNATED AS THE NEW MARGIN IS MARKED WITH BLUE INK WITH A PERIMETER OF BLACK INK. THE SPECIMEN IS SECTIONED AND ENTIRELY SUBMITTED LABELED B1 THROUGH 3.

PATH PROCEDURES

PROCEDURES:

88307, 88309, A BLK/23, B BLK/3

FINAL DIAGNOSIS

PART A LEFT MODIFIED RADICAL MASTECTOMY: IN SITU AND POORLY DIFFERENTIATED INFILTRATING DUCT CARCINOMA, NUCLEAR GRADE 3 OF 3 WITH A HIGH MITOTIC INDEX. GROSSLY THE TUMOR SPANNED A DISTANCE OF 70 MM. LYMPHATIC SPACE INVASION IS PRESENT. A HIGH-GRADE IN SITU COMPONENT OF THE COMEDOCARCINOMA TYPE IS PRESENT. 70 TO 80% OF THE TUMOR IS INVASIVE. INVASIVE CARCINOMA IS LOCATED IN THE LYMPHATIC SPACES AT THE DEEP MARGIN AND WITHIN THE STROMA OF THE BREAST 1.1 MM FROM THE DEEP MARGIN. TUMOR IS PRESENT IN A LYMPHATIC SPACE IN A SECTION FROM THE LOWER OUTER QUADRANT OF THE BREAST AWAY FROM THE GROSSLY IDENTIFIABLE PRIMARY LESION. METASTATIC CARCINOMA IS PRESENT IN 5 OF 18 AXILLARY LYMPH NODES.

PART B DEEP MARGIN BENEATH TUMOR, REEXCISION: SKELETAL MUSCLE WITH NO TUMOR IDENTIFIED.

CODE

1

Signed ______________________________ , M.D. (prelim.)

_________(signature on file)_________

Criteria	Yes	No
Diagnosis Discrepancy		✓
Primary Tumor Site Discrepancy		✓
HIPAA Discrepancy		✓
Prior Malignancy History		✓
Dual/Synchronous Primary Noted		✓
Case is (circle): QUALIFIED / UNQUALIFIED		
Reviewer Initials [illegible] Date Reviewed: [illegible]		

UUID:EC1BB6E8-6897-4E70-80A6-5A4E4B9C4E1F
TCGA-A2-A3XY-01A-PR
Redacted

SURGICAL PATHOLOGY REPORT

Patient: [redacted]
FMP/SSN: [redacted]
DOB/Age/Sex: (Age: Race:
Location:
Physician(s):

Specimen #:
Taken:
Received:
Reported:

ICD-0-3
Carcinoma, infiltrating duct, NOS 8500/3
Site: breast, NOS C50.9
Path: breast, upper outer quadrant C50.4
5-23-12 PD

AMENDED

SPECIMEN: LEFT BREAST AXILLARY & CONTENTS

FINAL DIAGNOSIS:

BREAST, LEFT, MASTECTOMY WITH AXILLARY DISSECTION:

- TUMOR TYPE: INVASIVE DUCTAL CARCINOMA (NO SPECIAL TYPE).
- TUMOR FOCALITY: UNIFOCAL.
- HISTOLOGIC GRADE: GRADE 3.
  - NOTTINGHAM SCORE: 9/9 (Tubules= 3, Nuclei= 3, Mitoses= 3; mitotic count 24 PER 10 high power fields).
- TUMOR SIZE (GREATEST DIMENSION): 2.5 CM (GROSS MEASUREMENT).
- TUMOR SIDE (QUADRANT): LEFT, UPPER OUTER QUADRANT.
- TUMOR NECROSIS: PRESENT IN INVASIVE AND IN SITU CARCINOMA.
- MICROCALCIFICATIONS: PRESENT IN DUCTAL CARCINOMA IN SITU AND BENIGN BREAST TISSUE.
- VENOUS/LYMPHATIC INVASION: NONE DEFINITELY IDENTIFIED.
- INTRADUCTAL COMPONENT: DUCTAL CARCINOMA IN SITU, NUCLEAR GRADE 3, SOLID TYPE WITH CENTRAL EXPANSIVE "COMEDO" NECROSIS.
- MARGINS:
  - DISTANCE OF INVASIVE CARCINOMA FROM CLOSEST MARGIN: 1.2 MM (SUPERFICIAL MARGIN).
  - DISTANCE OF IN SITU CARCINOMA FROM CLOSEST MARGIN: 1.0 MM (SUPERFICIAL MARGIN) AND 1.2 MM (DEEP MARGIN).
- LYMPH NODES: 3 OF 17 NODES POSITIVE FOR METASTATIC CARCINOMA.
  - LARGEST METASTATIC FOCUS: 1.2 CM (MICROSCOPIC MEASUREMENT).
  - EXTRACAPSULAR EXTENSION: ABSENT.
- NIPPLE INVOLVEMENT: ABSENT.
- SKIN INVOLVEMENT: ABSENT.
- PATHOLOGIC STAGE (AJCC EDITION): pT2 N1a.
- ANCILLARY STUDIES:
  - ESTROGEN RECEPTORS: NEGATIVE (0% NUCLEAR STAINING).
  - PROGESTERONE RECEPTORS: NEGATIVE (0% NUCLEAR STAINING).
  - HER2 BY FISH: NEGATIVE (HER2/CEP17 RATIO: 1.0; PER , PERFORMED ON PRIOR SPECIMEN FROM ',
- ADDITIONAL PATHOLOGIC CHANGES:
  - PRIOR BIOPSY SITE CHANGES.

[redacted]

Continued on Next Page
FOR OFFICIAL USE ONLY - PERSONAL DATA - PRIVACY ACT OF

SURGICAL PATHOLOGY REPORT

Patient: [redacted] Specimen #:

FINAL DIAGNOSIS (continued):

- FIBROADENOMA (1.2 CM; MICROSCOPIC MEASUREMENT).
- USUAL DUCTAL HYPERPLASIA, MODERATE.
- SMALL PERIPHERAL PAPILLOMA.
- FIBROCYSTIC CHANGES.

Comment: This case is amended to add the results of immunohistochemical stains for estrogen and progesterone receptors, as noted above. No other changes are made.

Although ER and PR stains were previously performed on the patient's core biopsy , they were repeated due the greater number of neoplastic cells in the current specimen and due to the focal staining for progesterone receptors (reportedly 1%) in the prior biopsy.

Estrogen and progesterone receptors were evaluated by immunohistochemical methods (estrogen receptor antibody 1D5, progesterone receptor antibody PgR636). A positive test is defined as easily discernable nuclear staining in 1% or more of the tumor cells.

** Report Electronically Signed Out **

==

CLINICAL DIAGNOSIS AND HISTORY:
-year0old with left breast mass in Diagnostic mammography and ultrasound on confirmed presence of a 2.2 x 1.4 cm left upper outer quadrant breast mass.

PRE-OPERATIVE DIAGNOSIS:
Left breast mass; breast cancer.

POST-OPERATIVE DIAGNOSIS:
None provided.

GROSS DESCRIPTION:
Received fresh, labeled with the patient's name [redacted] and designated, "Left Breast Axillary and Contents" and consists of a left mastectomy oriented with a short stitch superior; two short stitches posterior; one long lateral; and two long on an axillary lymph node. The specimen weighs 381 grams and measures 17.0 cm medially to laterally; 15.0

 Continued on Next Page

[redacted]

SURGICAL PATHOLOGY REPORT

Patient: [redacted] Specimen #:

GROSS DESCRIPTION (continued):

cm superiorly to inferiorly; and 2.5 cm anterior to posterior. The darkly pigmented superficial skin ellipse measures 15.5 x 5.5 cm and displays a 1.5 x 1.0 x 1.0 cm centrally located, everted nipple, free of discharge. The deep margin is inked black and the superficial margin is inked blue. Serial sections reveal a fairly well defined, firm pink-tan mass in the upper outer quadrant. The mass measures 2.5 x 1.4 x 0.4 cm, comes to within 0.4 cm of the deep margin and abuts the superficial margin. Immediately adjacent to, and continuous with the mass, is a 0.4 cm centrally located, red-brown probable biopsy cavity. A 1.2 x 1.2 x 0.5 cm well-circumscribed, rubbery fibrous nodule is noted in the lower mid breast which is consistent with a fibroadenoma. The remainder of the specimen is composed of markedly dense tan-white fibrous tissue with minimal admixed adipose tissue. No additional lesions are identified.

The attached axillary tail measures 8.0 x 6.5 x 2.0 cm. Sectioning reveals 11 lymph nodes ranging in size from 0.3 cm in greatest dimension to 2.5 x 2.0 x 1.7 cm. On sectioning, the two largest lymph nodes, one of which is tagged with a suture, are tan-white and moderately firm, consistent with involvement by metastatic lesion.

Representative sections are submitted as follows: 1- nipple; 2- mass with deep margin; 3- mass with superficial margin; 4- mass with deep margin; 5- mass with superficial margin; 6-7- mass with deep margin; 8-9- mass with superficial margin; 10- mass; 11- biopsy cavity; 12- biopsy cavity; 13- lower mid nodule; 14- UIQ; 15- LIQ; 16- LOQ; 17; central (adjacent to mass); 18- one lymph node (marked with suture); 19- five lymph nodes; 20- one lymph node; 21- one lymph node, bisected; 22- one lymph node; 23- one lymph node; 24-25- one lymph node, sectioned.

Matched sections of 1,5,8-10, 12-18, 20 and 22-24 are submitted in OCT/Paraffin per Time in formalin: >80 hours.

[redacted]

End of Report

Criteria	Yes	No
Diagnosis Discrepancy		✓
Primary Tumor Site Discrepancy		✓
HIPAA Discrepancy		✓
Prior Malignancy History		✓
Dual/Synchronous Primary Noted		✓
Case is (circle): QUALIFIED / DISQUALIFIED		
Reviewer Initials [illegible] Date Reviewed: [illegible]		

[illegible] 3/17/12

UUID:1D4CA1BA-B534-4CEA-ABA5-EF9B31694204
TCGA-A2-A25C-01A-PR Redacted

[redacted] /F [redacted]

Specimen: **Received:** **Status:** **Req#:** [redacted]
Spec Type: SURGICAL P **Subm Dr:**

ICD-0-3
Carcinoma, infiltrating duct, NOS 8500/3
Site: breast, NOS C50.9 [illegible]
4/27/11

PREOPERATIVE DIAGNOSIS

LEFT BREAST CANCER INVASIVE

OPERATION PERFORMED

DATE:
DOCTOR(S):
PROCEDURE: SENTINEL NODE BX/SIMPLE MASTECTOMY

TISSUE RECEIVED

A. LT BREAST SIMPLE MASTECTOMY
B. LT SENTINEL NODE #1
C. LT SENTINEL NODE #2

GROSS DESCRIPTION

RECEIVED IN 3 PARTS.

PART A RECEIVED LABELED [redacted] SIMPLE LEFT BREAST MASTECTOMY STITCH AT 12 O'CLOCK, IS A SIMPLE MASTECTOMY SPECIMEN MEASURING 17.5 X 16 X 2.8 CM. THE NIPPLE IS UNREMARKABLE WITHIN A 9 X 2.5-CM SKIN ELLIPSE. ADDITIONALLY WITHIN THE CONTAINER ARE 2 IRREGULAR FRAGMENTS OF FATTY TISSUE WITH NO PALPABLE MASSES TOGETHER MEASURING 4.5 X 3 X 0.5 CM. THE SUPERFICIAL ASPECT IS MARKED WITH BLUE INK, THE DEEP WITH BLACK. SECTIONING REVEALS 2 DISCRETE GRAY FIBROUS NODULES; THE MOST LATERAL IS AT 3 O'CLOCK MEASURING 1.5 X 1.1 X 1.5 CM. ADJACENT TO THIS SEPARATED BY 1.5 CM IN THE 6:30 AREA IS A MASS WITH A COIL CLIP MEASURING 2.2 X 1.8 X 2.1 CM. THESE ARE BOTH 0.5 CM FROM THE DEEP MARGIN. CENTRALLY, THE BREAST HAS DENSE TAN FIBROUS TISSUE, BUT THE PERIMETER OF THE SPECIMEN AND THE TISSUE ADJACENT TO THE DEEP MARGIN IS YELLOW FATTY TISSUE. THE 3 O'CLOCK LESION IS 0.7 CM FROM THE SUPERFICIAL MARGIN. THE 6 O'CLOCK LESION EXTENDS TO WITHIN 0.6 CM OF THE SUPERFICIAL MARGIN. SECTIONS ARE SUBMITTED AS FOLLOWS: A1--NIPPLE; A2--TISSUE BETWEEN THE TWO LESIONS; A3--3 O'CLOCK LESION TO INCLUDE DEEP MARGIN; A4--3 O'CLOCK LESION TO INCLUDE SUPERFICIAL MARGIN; A5 AND 6--FULL CROSS-SECTION OF 6 O'CLOCK LESION TO INCLUDE THE DEEP AND SUPERFICIAL MARGIN, RESPECTIVELY; A7--6 O'CLOCK LESION; A8--UPPER OUTER QUADRANT; A9--UPPER INNER QUADRANT; A10--LOWER INNER QUADRANT; A11--LOWER OUTER QUADRANT. TISSUE FROM EACH LESION IS SUBMITTED PER PROTOCOL.

PART B RECEIVED LABELED [redacted] LEFT SENTINEL NODE #1 HOT AND BLUE, IS AN OVOID FRAGMENT OF YELLOW-RED SOFT TISSUE MEASURING 1.7 X 1.0 X 0.9 CM. SECTIONING REVEALS A GROSSLY UNREMARKABLE 1.3-CM LYMPH NODE WITH CENTRAL FAT REPLACEMENT AND BLUE COLORATION. THIS IS SUBMITTED LABELED B, BISECTED.

PART C RECEIVED LABELED [redacted] LEFT SENTINEL NODE #2 HOT

Patient: [redacted] [redacted] (Continued)

Specimen: **Received:** **Status:** **Req#**[redacted]

Spec Type: SURGICAL P **Subm Dr:**

GROSS DESCRIPTION (Continued)

AND BLUE, IS A 1.5 X 0.6 X 0.4-CM OVOID PORTION OF RED-TAN TISSUE. SECTIONING REVEALS THIS TO BE A GROSSLY UNREMARKABLE LYMPH NODE WITH BLUE COLORATION SUBMITTED LABELED C, BISECTED.

PATH PROCEDURES

PROCEDURES:

88307/3, IMMUNOPEROXIDAS/2, A BLK/11, BBX X6, CBX X6

FINAL DIAGNOSIS

PART A LEFT BREAST, SIMPLE MASTECTOMY:

1. MODERATELY DIFFERENTIATED INFILTRATING DUCT CARCINOMA, NUCLEAR GRADE II/III, MODERATE MITOTIC INDEX AND TUBULE FORMATION 2, WITH A TOTAL NOTTINGHAM SCORE OF 6. RARE FOCI OF DCIS OF THE CRIBRIFORM AND SOLID TYPES, NUCLEAR GRADE II WITH LOW MITOTIC INDEX, ARE SEEN AT THE PERIPHERY OF THE TUMOR. *IDC 2/2/2 rare DCIS g2 crib solid*

2. A 1.5-CM TUMOR IS IDENTIFIED IN THE 3 O'CLOCK LOCATION AND A 2.2-CM LESION IS IDENTIFIED IN THE 6 O'CLOCK PORTION WHICH ARE SEPARATED BY AT LEAST 1.5 CM OF BENIGN BREAST TISSUE. *multifocal 1.5cm apart*

3. THE MARGINS OF EXCISION, NIPPLE SKIN AND RANDOM SECTIONS OF REMAINING QUADRANTS ARE FREE OF TUMOR. *2.2cm & 1.5cm*

4. LYMPHOVASCULAR INVASION IS IDENTIFIED. *+LVI*

5. BIOPSY CHANGES ARE PRESENT. *Bx Δ*

PART B LEFT AXILLA, SENTINEL LYMPH NODE BIOPSY #1: METASTATIC BREAST CARCINOMA IS MULTIFOCALLY IDENTIFIED ON H&E STEP SECTIONS AND CYTOKERATIN STAINS IN 1 LYMPH NODE EXAMINED, THE LARGEST FOCUS OF WHICH MEASURES 3 MM IN GREATEST MICROSCOPIC DIMENSION. NO EVIDENCE OF EXTRANODAL EXTENSION. *1/1 SN*

PART C LEFT AXILLA, SENTINEL LYMPH NODE BIOPSY #2: MULTIFOCAL METASTATIC BREAST CARCINOMA IS IDENTIFIED ON H&E STEP SECTIONS AND CYTOKERATIN STAINS IN 1 LYMPH NODE EXAMINED, THE LARGEST FOCUS OF WHICH MEASURES 2 MM IN GREATEST MICROSCOPIC DIMENSION. EXTRANODAL EXTENSION IS NOT PRESENT. *1/1 SN*

Patient: [redacted] [redacted] **(Continued)**

Specimen: **Received:** **Status:** **Req#:** [redacted]

Spec Type: SURGICAL P **Subm Dr:**

DIAGNOSTIC/THERAPEUTIC MARKERS

Her-2/neu: NEGATIVE
Score: 1+

Reference Range: Negative 0 - 1+
Equivocal 2+
Positive 3+

Electronically signed by: M.D.
See full scanned report for details.
Test performed at:

SEND OUT

1. **SENT TO:**
2. **REQUESTED BY:** DR
3. **DATE SENT:**
4. **METHOD OF TRANSPORTATION:** COURIER
5. **NO. OF BLOCKS:** 1 BLOCK A3
7. **MATERIAL(S) TO BE RETURNED:** Y
14. **SPECIMEN SENT(Y/N):** Y

Signed ______________________ M.D. (prelim.)

_____Electronically signed by:_____ M.D.

Criteria	Yes	No
Diagnosis Discrepancy		✓
Primary Tumor Site Discrepancy		✓
HIPAA Discrepancy		✓
Prior Malignancy History		✓
Dual/Synchronous Primary Noted		✓
Case is (circle): QUALIFIED / DISQUALIFIED		
Reviewer Initials [illegible] Date Reviewed: [illegible]		

UUID:8A853D7F-979F-4112-9659-1FDE84EF5102
TCGA-A7-A0CE-01A-PR Redacted

ICD-0-3
Carcinoma, infiltrating duct, NOS 8500/3
Site: breast, NOS C50.9 fw 9/3/11

SPECIMEN
A. Left axillary sentinel node
B. Left breast tissue

CLINICAL NOTES
PRE-OP DIAGNOSIS: Left breast cancer

FROZEN SECTION DIAGNOSIS
A) Lymph node, left axillary sentinel node, excision - One lymph node negative for metastatic carcinoma (0/1).

GROSS DESCRIPTION
A. Received fresh for frozen section labeled "left axillary sentinel node" are irregularly-shaped fragments of fibroadipose tissue that measure 2.5 x 2.2 x 1.2 cm. in dimension. Within the tissue a lymph node is identified that measures 1.6 cm. in maximum dimension. The lymph node is bisected and frozen as AFS1 and AFS2.
B. Received fresh in a container labeled "left breast tissue" is an 8 x 7 x 5 cm. portion of soft tan-yellow breast tissue partially covered with a 4.5 x 2 cm. tan-pink wrinkled skin ellipse. There are orienting sutures present. The specimen is inked as follows based upon the orienting sutures: superior - orange; anterior - blue; posterior - black; inferior - green. The specimen is sectioned from medial to lateral. On cut surface, there is a 2.8 x 2.8 x 2.5 cm. tan white-pink indurated mass, which is 1.1 cm. from the nearest margin, which is the inferior posterior margin.
The specimen away the mass consists of yellow adipose tissue with scant interspersed tan tissue. RS-10.

BLOCK SUMMARY: B1 - medial margin; B2 - lateral margin; B3,B4 - tumor to nearest margin (inferior/posterior); B5,6 - tumor to posterior/deep margin; 7 - superior margin; 8 - skin to tumor; 9,10 - specimen away from mass lesion.

GROSS DESCRIPTION

MICROSCOPIC DESCRIPTION

A. Serial H&E stained sections and immunostains with antibodies to keratin (AE1-AE3) are examined, and the sentinel lymph node is negative for malignancy.

B. The following template summarizes the findings in this part:

Invasive Carcinoma: Present
  Histologic type: Invasive ductal carcinoma.
  Histologic grade:
    Overall grade: 3
      Architectural score: 3
      Nuclear score: 3
      Mitotic score: 3

  Greatest dimension (pT): 2.8 cm. (pT2)
  Specimen margins: Negative
  Vessel invasion: Not definitively identified
  Calcification: There is necrosis within invasive carcinoma which has focal calcification.

Ductal carcinoma in situ: Present focally
  Histologic pattern: Solid
  Nuclear grade: 3
  Central necrosis: Absent
  Extensive intraductal component: Absent
  Specimen margins: Negative

MICROSCOPIC DESCRIPTION

Description of non-tumorous breast: Changes compatible with reaction
to prior biopsy. Fibrocystic changes.

Prognostic markers: Previously performed (see

4x2,14,15,20x2

[A few of the antibodies used in our laboratory may be classified as
analyte specific reagents. These antibodies are monitored and controlled in our laboratory and their performance for in vitro diagnosis is well described in the medical literature. They have not been cleared or approved by the FDA.]

DIAGNOSIS

A. Lymph node, left axially sentinel, excision -
Negative for malignancy.

B. Breast, left, biopsy -
Invasive ductal carcinoma, margins negative for malignancy.

(Electronic Signature)

--- End Of Report ---

Criteria	Yes	No
Diagnosis Discrepancy		✓
Primary Tumor Site Discrepancy		✓
HIPAA Discrepancy		✓
Prior Malignancy History		✓
Dual/Synchronous Primary Noted		✓
Case is (circle): QUALIFIED / DISQUALIFIED		
Reviewer Initials [illegible] Date Reviewed: 9/3/11		

UUID:6F3784B2-4A75-447C-9EAE-F146461C737E
TCGA-A7-A13D-01A-PR Redacted

ICD-0-3
Carcinoma - Infiltrating Duct, NOS
8500/3
Site: breast, NOS C50.9
11/14/10

SPECIMEN

A. Sentinel node #1 (84) hot and blue right axilla
B. Sentinel node #2 (237) hot and blue right axilla
C. Sentinel node #3 (277) hot and blue right axilla
D. Segmental mastectomy right breast single suture lateral, double long anterior
E. Superior wall segmental wall right breast; stitch at new superior margin
F. Posterior wall segmental mastectomy right breast; stitch at new posterior margin
G. Retroareolar mass right breast
H. Sentinel node #4 (300) hot and blue right axilla

CLINICAL NOTES

Right breast cancer

FROZEN SECTION DIAGNOSIS

A. Sentinel node #1, right axilla:
One benign lymph node (0/1).
B. Sentinel node #2, right axilla:
One negative lymph node (0/1).
C. Sentinel node #3, right axilla:
One negative lymph node (0/1).
H. Sentinel node #4, right axilla: Benign (0/1).

GROSS DESCRIPTION

A. Received unfixed for frozen section, labeled "sentinel node #1 hot and blue 84 right axilla" is a 1.4 x 1.1 x 0.6 cm lymph node, bisected and entirely submitted in two blocks.

B. Received unfixed for frozen section, labeled "sentinel node #2 (237) hot and blue right axilla", is a 1 x 0.8 x 0.6 cm lymph node, bisected and entirely submitted in one block.

C. Received unfixed for frozen section, labeled "sentinel

node #3 (277) hot and blue right axilla," is a lymph node that is 3.5 x 3 x 2 cm quadrisected and entirely submitted in four blocks.

D. Received unfixed for tissue procurement, labeled "segmental mastectomy right breast", is a portion of fibroadipose tissue that is oriented with sutures as previously stated, and 8 cm. from medial to lateral, 5.4 cm. from anterior to posterior, and 4.0 cm. from superior to inferior. A mass lesion is identified, 3 x 2.5 x 2.4 cm., a portion of which is submitted for tissue procurement. Margins are inked: superior black, anterior green, inferior yellow, posterior blue. The mass lesion is 2 mm. from the black superior inked margin, and possible less than 1 mm. from the yellow inferior margin. Representative sections are submitted sequentially from lateral to medial, with D1 being the lateral margin, D2-D4 being representative sections between lateral margin and beginning of mass lesion, including representative deep

margin and debris of prior biopsy site, with D15 being medial shaved margins. A total 15 cassettes. RS15.

E. Received fresh subsequently fixed in formalin labeled "superior wall right breast" is a 4.5 x 3.0 x 1.2 cm. yellow lobular fatty tissue fragment which has a suture designating a new superior margin. The surface is inked blue. The specimen is sectioned to show no other discrete gross lesions identified and the yellow lobular fatty cut surface. The specimen is entirely submitted in 6 cassettes. AS-6

F. Received fresh subsequently fixed in formalin labeled "posterior wall right breast" is a 1.7 x 1.6 x 1.0 cm. pink yellow rubbery tissue fragment which has a suture designated to the new posterior margin. This is located on skeletal muscle. The surface is inked blue. The opposite surface is yellow, pink and fatty. No residual tumor is grossly identified. The specimen is sectioned and entirely submitted in one cassette. AS-1

G. Received fresh subsequently fixed in formalin labeled

GROSS DESCRIPTION

"retroareolar" is a white pink rubbery nodule which shows gross evidence of bluing staining present. The specimen is 2.5 x 2.2 x 1.7 cm., having a single suture designating lateral and double suture designating anterior. The specimen is inked as follows: Superior orange, anterior blue, posterior black, inferior green. The specimen is sectioned from medial to lateral to show a white tan whorled cut surface, shows white rice like pellets located in the inferior aspect of the specimen. The specimen is entirely submitted from medial to lateral labeled "1-5". AS-5.

MICROSCOPIC DESCRIPTION

Invasive Carcinoma:

- Histologic type: Ductal
- Histologic grade: III
  - Overall grade: 9/9
    - Architectural score: 3
    - Nuclear score: 3
    - Mitotic score: 3
- Greatest dimension (pT2): 3.0 cm
- Specimen margins: invasive carcinoma is close to the margins in the main resection specimen (part D): 1.5 mm from inferior margin, 1 mm from posterior margin, and 1 mm from anterior margin. Additional margin specimens (superior wall, posterior wall) are negative for carcinoma.

Vessel invasion: Not identified
Calcification: present
Necrosis: present

Ductal carcinoma in situ:
Histologic pattern: Solid
Nuclear grade: 3

MICROSCOPIC DESCRIPTION

Central necrosis: Present
% DCIS of total tumor (if mixed): <1%
Extensive intraductal component: absent
Specimen margins: Negative
Calcification: Present

Description of non-tumorous breast: Fibroadenoma (part G, retroareolar)
Comments: Changes of prior biopsy identified (parts D, G).
Prognostic markers: See core biopsy report,

4x7, 3, 14x4, 15x4

DIAGNOSIS

A. Sentinel lymph node #1, right axilla, biopsy:
One negative lymph node (0/1).
B. Sentinel lymph node #2, right axilla, biopsy:
One negative lymph node (0/1).
C. Sentinel lymph node #3, right axilla, biopsy:
One negative lymph node (0/1).
D. Breast, right, segmental resection:
Invasive ductal carcinoma, grade III, close to excisional margins.
E. Breast, superior wall, new margin:
Negative for carcinoma.
F. Breast and muscle tissue, posterior wall, new margin:
Negative for carcinoma.
G. Breast, right retroareolar, biopsy:
Fibroadenoma.
H. Sentinel lymph node #4, right axilla, biopsy:
One negative lymph node (0/1).

DIAGNOSIS

(Electronic Signature)

riteria	Yes	No
iagnosis Discrepancy		✓
rimary Tumor Site Discrepancy		✓
IIPAA Discrepancy		✓
rior Malignancy History		✓
ual/Synchronous Primary Noted		✓

ase is (circle): QUALIFIED / DISQUALIFIED
eviewer Initials: [illegible] Date Reviewed: 11/16/10

Collection Date:
Hospital of Origin:
Copy to.

QC Pathologist:

FINAL PATHOLOGIC DIAGNOSIS:
A. Right axillary sentinel lymph node:
One lymph node, total replacement by metastatic adenocarcinoma.
Confirms frozen section diagnosis.
Size of involved node: 2 cm.
B. Right breast mastectomy:
Invasive ductal carcinoma.
Architectural score: 1 of 3.
Nuclear score: 2 of 3.
Mitotic score: 2 of 3.
Total score: 5 of 9 = Grade I.
Size: 3.8 cm.
No evidence of skin or nipple involvement.
Deep margin of excision is free of carcinoma.
C. Right axillary sentinel lymph node #2:
One lymph node, no evidence of metastatic carcinoma.
pTNM classification: pT2 pN1 Mx.

COMMENTS:

ICD O 3
carcinoma, infiltrating duct, NOS 8500/3
Site: breast, NOS C50.9
[illegible] 10/27/11

CLINICAL HISTORY:
Preoperative Diagnosis: Right breast cancer ER positive 32%, PR 84%
Postoperative Diagnosis:
Symptoms/Radiologic Findings:
SPECIMENS:
A. Right axillary sentinel node at 900 with frozen section
B. Right breast
C. Right axillary sentinel node #2

CODES:

Criteria	Yes	No
Diagnosis Discrepancy		X
Primary Tumor Site Discrepancy		X
HIPAA Discrepancy		X
Prior Malignancy History		X
Dual/Synchronous Primary Noted		X
Case is (circle): QUALIFIED / DISQUALIFIED		
Reviewer Initials: KMH [illegible] 10/27/11	Date Reviewed: 10/27/11	

PROCEDURAL DEMOGRAPHICS:
Date of Procedure:

UUID:C976ACED-7FF8-4287-9D68-C6FD17895FC0
TCGA-AC-A23E-01A-PR Redacted

Accession Date/Time:

GROSS DESCRIPTION:
The specimen is received in three containers labeled with the patient's name
A. Container A is additionally labeled 'right axillary sentinel node' and contains a 1.5 cm yellow-tan firm fatty nodule consistent with possible lymph node. The nodule is bisected and entirely submitted for frozen section with the residual entirely resubmitted for permanent section in cassette A labeled
B. Container B is additionally labeled 'right breast' and contains a 1050.0 g, 22.0 x 18.5 x 6.5 cm simple mastectomy specimen partially surfaced by a 22.5 x 12.5 cm portion of pink-tan skin bearing a central 1.5 x 1.3 x 0.7 cm everted nipple. Orientation is not offered or possible. Located 5.0 cm from the nipple is a 2.3 cm partially healed pink-tan linear incision possibly consistent with previous biopsy site. Additionally, three tan-brown granular lesions are identified. These lesions range from 0.4 up to 1.0 cm in greatest dimension and reside 3.0 to 6.0 from the nipple. The deep margin is inked and the specimen is serially sectioned to reveal a 3.8 x 3.5 x 2.6 cm gray-white firm, gritty mass surrounded by fibrosis. This mass resides 2.0 cm below the skin's surface and approaches to within 2.3 cm of the inked deep margin. The remainder of the cut surface is comprised of predominantly yellow-tan adipose tissue admixed with moderate amounts of interspersed gray-white cystic fibrous tissue. No additional lesions are identified. Representative sections are submitted in cassettes B1 - 11 labeled designated as follows: B1, nipple; B2, skin lesions; B3, inked deep margin, perpendicular; B4 - B8, mass; B9 - B11, uninvolved tissue from each of the three uninvolved quadrants. Additionally, a yellow and green cassette are submitted for genomics research each labeled

C. Container C is additionally labeled 'right axillary sentinel node #2' and contains a 0.9 cm yellow-tan firm fatty nodule consistent with possible lymph node. The specimen is bisected and entirely submitted in cassette C labeled

INTRA-PROCEDURE CONSULTATION:
A. FROZEN SECTION DIAGNOSIS: Positive for tumor per Dr.

Results were communicated to after confirming patient identity.

....

Clinical Diagnosis & History:
r/o female with left breast IDC

Specimens Submitted:
1: SP: Level three left axillary lymph nodes
2: SP: Level two left axillary lymph nodes
3: SP: Lateral left breast skin
4: SP: Right upper abdominal skin tag
5: SP: Left breast and level 1 axillary lymph nodes

UUID:6C202D3E-5670-4B63-82A2-9EE7AC385089
TCGA-AO-A0JB-01A-PR Redacted

DIAGNOSIS:

1) SOFT TISSUE, LEVEL III LEFT AXILLA; EXCISION:
- BENIGN FIBROADIPOSE TISSUE.
- NO LYMPH NODE IDENTIFIED.

2) LYMPH NODES, LEVEL II LEFT AXILLA; EXCISION:
- THREE BENIGN LYMPH NODES (0/3).

3) SOFT TISSUE, LATERAL LEFT BREAST; EXCISION:
- ONE BENIGN LYMPH NODE (0/1).
- BENIGN FIBROADIPOSE TISSUE.

4) SKIN, RIGHT UPPER ABDOMINAL TAG; EXCISION:
- IRRITATED SEBORRHEIC KERATOSIS.

5) BREAST AND LEVEL I AXILLARY LYMPH NODES, LEFT; MASTECTOMY:
- INVASIVE DUCTAL CARCINOMA, HISTOLOGIC GRADE III/III (SLIGHT OR NO TUBULE FORMATION), NUCLEAR GRADE II/III (MODERATE VARIATION IN SIZE AND SHAPE), WITH AREAS OF LOBULAR GROWTH, MEASURING 11.0 CM IN LARGEST DIMENSION GROSSLY.
- DUCTAL CARCINOMA IN SITU (DCIS) IS ALSO IDENTIFIED, SOLID TYPE, WITH MODERATE HIGH NUCLEAR GRADE AND EXTENSIVE NECROSIS.
- LOBULAR INVOLVEMENT BY DCIS IS PRESENT.
- THE DCIS CONSTITUTES <= 25% OF THE TOTAL TUMOR MASS, AND IS PRESENT ADMIXED WITH AND AWAY FROM THE INVASIVE COMPONENT.
- THE INVASIVE CARCINOMA IS LOCATED IN THE UPPER OUTER QUADRANT, UPPER INNER QUADRANT AND LOWER OUTER QUADRANT.
- THE DCIS IS LOCATED IN THE UPPER OUTER QUADRANT, UPPER INNER QUADRANT AND LOWER OUTER QUADRANT.

** Continued on next page **

ICD-O-3
carcinoma, infiltrating duct, NOS 8500/3
Site: breast, NOS C50.9 lw 10/24/11

Criteria	Yes	No
Diagnosis Discrepancy		X
Primary Tumor Site Discrepancy		X
HIPAA Discrepancy		X
Prior Malignancy History		X
Dual/Synchronous Primary Noted		X
Case is (circle): QUALIFIED / DISQUALIFIED		
Reviewer Initials [illegible] Date Reviewed: [illegible]		

lw 10/24/11

- NO INVOLVEMENT OF THE NIPPLE BY EITHER IN SITU OR INVASIVE CARCINOMA IS IDENTIFIED.
- CALCIFICATIONS ARE PRESENT IN THE IN SITU AND INVASIVE CARCINOMA, AND IN BENIGN BREAST PARENCHYMA.
- EXTENSIVE VASCULAR INVASION IS PRESENT.
- NO INVOLVEMENT OF THE SURGICAL MARGINS BY EITHER INVASIVE OR IN SITU CARCINOMA IS IDENTIFIED.
- NO SKIN INVOLVEMENT BY CARCINOMA IS IDENTIFIED.
- THE ATTACHED SKELETAL MUSCLE IS UNINVOLVED BY CARCINOMA.
- THE NON-NEOPLASTIC BREAST TISSUE SHOWS BIOPSY SITE CHANGES AND PROLIFERATIVE FIBROCYSTIC CHANGES.
- THE LYMPH NODE STATUS IS AS FOLLOWS (EXPRESSED AS THE NUMBER OF POSITIVE LYMPH NODES IN RELATION TO THE TOTAL NUMBER OF LYMPH NODES EXAMINED): LEVEL I: 1/10.
- THERE IS EXTRANODAL EXTENSION OF CARCINOMA, >2 MM.

IMMUNOHISTOCHEMICAL STAINS WERE PERFORMED ON FORMALIN-FIXED TISSUE WITH THE FOLLOWING RESULTS FOR INVASIVE CARCINOMA (BLOCK 5T5):

ESTROGEN RECEPTOR (6F11, 90% NUCLEAR STAINING WITH STRONG INTENSITY

PROGESTERONE RECEPTOR (1E2; 60% NUCLEAR STAINING WITH MODERATE INTENSITY

HER2 (HERCEPTEST; DAKO): NEGATIVE (STAINING INTENSITY OF 0)

2

CONTROLS ARE SATISFACTORY.

COMMENT: HERCEPTESTTM (DAKO) IS AN FDA-APPROVED METHOD FOR ASSESSMENT OF HER2 PROTEIN OVEREXPRESSION IN BREAST CANCER TISSUE ROUTINELY PROCESSED FOR HISTOLOGICAL EVALUATION. THE HER2 TEST RESULTS ARE REPORTED IN ACCORDANCE WITH THE ASCO/CAP GUIDELINE RECOMMENDATIONS FOR HER2 TESTING IN BREAST CANCER (J CLIN ONCOL 2007; 25(1):1-28).

I ATTEST THAT THE ABOVE DIAGNOSIS IS BASED UPON MY PERSONAL EXAMINATION OF THE SLIDES (AND/OR OTHER MATERIAL), AND THAT I HAVE REVIEWED AND APPROVED THIS REPORT.

*** Report Electronically Signed Out ***

Special Studies:

Result	Special Stain	Comment
	ER-C	
	PR-C	
	HER2-C	
	IMM RECUT	
	NEG CONT	
	NEG-HER2	

Gross Description:

** Continued on next page **

MD

D.

1.) The specimen is received in formalin, labeled "level 3 left axillary lymph nodes" and consists of two irregularly shaped fragments of yellow lobulated adipose tissue measuring 1.0 x 1.0 x 0.2 cm in aggregate. No lymph nodes are identified grossly. The specimen is entirely submitted.

Summary of sections:
U-undesignated

2.) The specimen is received in formalin, labeled "level 2 left axillary lymph nodes" and consists of one irregularly shaped fragment of yellow lobulated adipose tissue measuring 3.5 x 2.5 x 0.3 cm. Three possible lymph nodes are identified, ranging from 0.3 cm to 0.5 cm in greatest dimension. The lymph nodes are entirely submitted.

Summary of sections:
U -- undesignated

4.D.

3.) The specimen is received in formalin, labeled "lateral left breast skin" and consists of one irregularly shaped fragment of yellow to tan fibroadipose tissue measuring 10.3 x 2.9 x 1.4 cm. No skin is identified grossly. Serial sectioning reveals a grossly unremarkable fibroadipose tissue with a single possible lymph node measuring 0.4 cm in greatest dimension. Representative sections are submitted.

3

Summary of sections:
U-undesignated

.D.

4.) The specimen is received in formalin, labeled "right upper abdominal skin tag" and consists of one polypoid shaped fragment of brown skin measuring 0.3 x 0.2 x 0.2 cm. The specimen is entirely submitted.

Summary of sections:
U-undesignated

.D.

5.) The specimen is received fresh, labeled "left breast and level 1 axillary lymph nodes, stitch marks level 1 axillary lymph nodes" and consists of a breast with attached axillary tail. The breast measures 22.2 x 16.0 x 3.5 cm with overlying skin ellipse measuring 16.6 x 7.9 cm. Situated eccentrically on the skin surface is an everted nipple measuring 1.2 x 1.1 x 0.3 cm and areola measuring 4.9 x 4.1 cm. The skin shows no scars. A suture demarcates the axillary tail which measures 9.8 x 5.5 x 1.5 cm. The posterior surface of the breast is inked black and the specimen is serially sectioned to reveal an irregularly shaped white-tan firm mass

** Continued on next page **

measuring 11.0 x 6.5 x 4.8 cm, involving upper outer, lower outer and upper inner quadrants, located 0.6 cm away from the deep resection margin. The remaining breast tissue shows predominantly yellow lobulated adipose tissue admixed with scant white-tan fibrous soft tissue with no other grossly identifiable lesions. The axillary tissue is dissected to reveal multiple lymph nodes ranging in size from 0.5 cm to 2.5 cm. The specimen is submitted for lymph node dissection. Representative sections of the mastectomy specimen and all identified axillary lymph nodes are submitted. Tissue submitted for TPS.

Summary of sections:
N - nipple
NB - nipple base
S - skin
D - deep margin
T - tumor
UIQ - upper inner quadrant
LIQ - lower inner quadrant
UOQ - upper outer quadrant
LOQ - lower outer quadrant
LNS -- lymph nodes

4

Summary of Sections:
Part 1: SP: Level three left axillary lymph nodes

Block	Sect. Site	PCs
1	U	1

Part 2: SP: Level two left axillary lymph nodes

Block	Sect. Site	PCs
1	U	1

Part 3: SP: Lateral left breast skin (sr)

Block	Sect. Site	PCs
1	U	1

Part 4: SP: Right upper abdominal skin tag

Block	Sect. Site	PCs
1	U	1

Part 5: SP: Left breast and level 1 axillary lymph nodes

Block	Sect. Site	PCs
1	D	1
2	LIQ	2
6	LN	11
2	LOQ	2

** Continued on next page **

1	N	1
1	NB	1
1	S	1
5	T	5
2	UIQ	2
2	UOQ	2

5

** End of Report **

Clinical Diagnosis & History:
year-old female with right breast mass s/p core biopsy showing invasive ductal carcinoma, poorly differentiated. Now for TM SLN with IR.

Specimens Submitted:
1: SP: Right breast and silicone implant
2: SP: Sentinel node #1 level one right axilla
3: SP: Sentinel node #2 level one right axilla
4: SP: Sentinel node #3 level one right axilla
5: SP: Sentinel node #4 level one right axilla
6: SP: Non-sentinel tissue right axilla
7: SP: Sentinel node #5 level one right axilla
8: SP: Additional superior right mastectomy flap tissue

DIAGNOSIS:

1) BREAST, RIGHT AND SILICONE IMPLANT; MASTECTOMY:
- INVASIVE DUCTAL CARCINOMA, HISTOLOGIC GRADE III/III (SLIGHT OR NO TUBULE FORMATION), NUCLEAR GRADE III/III (MARKED VARIATION IN SIZE AND SHAPE), MEASURING 3.1 CM IN LARGEST DIMENSION GROSSLY.
- DUCTAL CARCINOMA IN SITU (DCIS) IS ALSO IDENTIFIED, SOLID AND CRIBRIFORM TYPES WITH INTERMEDIATE TO HIGH NUCLEAR GRADE AND MINIMAL NECROSIS. THE DCIS CONSTITUTES <= 25% OF THE TOTAL TUMOR MASS, AND IS PRESENT ADMIXED WITH THE INVASIVE COMPONENT.
- THE INVASIVE CARCINOMA IS LOCATED IN THE UPPER OUTER QUADRANT. THE DCIS IS LOCATED IN THE UPPER OUTER QUADRANT.
- NO INVOLVEMENT OF THE NIPPLE BY EITHER IN SITU OR INVASIVE CARCINOMA IS IDENTIFIED.

- NO CALCIFICATIONS ARE IDENTIFIED IN EITHER THE INVASIVE OR IN SITU CARCINOMA.
- NO VASCULAR INVASION IS NOTED.
- NO INVOLVEMENT OF THE SURGICAL MARGINS BY EITHER INVASIVE OR IN SITU CARCINOMA IS IDENTIFIED.
- INVASIVE CARCINOMA IS 0.2 CM FROM THE NEAREST (ANTERIOR AND DEEP) MARGINS.
- NO SKIN INVOLVEMENT BY CARCINOMA IS IDENTIFIED.
- THE NON-NEOPLASTIC BREAST TISSUE SHOWS BIOPSY SITE CHANGES AND FIBROCYSTIC CHANGES WITH APOCRINE METAPLASIA, MICROCALCIFICATIONS, AND COLUMNAR CELL ALTERATION WITHOUT ATYPIA.

PATH REPORT

** Continued on next page **

ICD-0-3
carcinoma, infiltrating duct, NOS 8500/3
Site breast, NOS C50.9 lw 10/22/11

UUID:A54E82A4-D7C2-4A5C-A69C-C66976F0282F
TCGA-AO-A12A-01A-PR Redacted

Criteria	Yes	No
Diagnosis Discrepancy		
Primary Tumor Site Discrepancy		
HIPAA Discrepancy		
Prior Malignancy History		
Dual/Synchronous Primary Noted		
Case is (circle): QUALIFIED / DISQUALIFIED		
Reviewer Initials [illegible] Date Reviewed: [illegible]		

lw 10/22/11

- RESULTS OF SPECIAL STAINS (ER, PR, HER2-NEU) WILL BE REPORTED AS AN ADDENDUM.

2

2) LYMPH NODE, SENTINEL #1, LEVEL I, RIGHT AXILLA; EXCISION:
- ONE BENIGN LYMPH NODE (0/1). DEEPER LEVEL RECUTS AND SPECIAL STAINS HAVE BEEN ORDERED. THE RESULTS WILL BE REPORTED IN AN ADDENDUM.

3) LYMPH NODE, SENTINEL #2, LEVEL I, RIGHT AXILLA; EXCISION:
- ONE BENIGN LYMPH NODE (0/1). DEEPER LEVEL RECUTS AND SPECIAL STAINS HAVE BEEN ORDERED. THE RESULTS WILL BE REPORTED IN AN ADDENDUM.

4) LYMPH NODE, SENTINEL #3, LEVEL I, RIGHT AXILLA; EXCISION:
- ONE BENIGN LYMPH NODE (0/1). DEEPER LEVEL RECUTS AND SPECIAL STAINS HAVE BEEN ORDERED. THE RESULTS WILL BE REPORTED IN AN ADDENDUM.

5) LYMPH NODE, SENTINEL #4, LEVEL I, RIGHT AXILLA; EXCISION:
- ONE BENIGN LYMPH NODE (0/1). DEEPER LEVEL RECUTS AND SPECIAL STAINS HAVE BEEN ORDERED. THE RESULTS WILL BE REPORTED IN AN ADDENDUM.

6) NON-SENTINEL TISSUE, RIGHT AXILLA; EXCISION:
- TWO BENIGN LYMPH NODES (0/2).

7) LYMPH NODES, SENTINEL #5, LEVEL I, RIGHT AXILLA; EXCISION:
- TWO BENIGN LYMPH NODES (0/2). DEEPER LEVEL RECUTS AND SPECIAL STAINS HAVE BEEN ORDERED. THE RESULTS WILL BE REPORTED IN AN ADDENDUM.

8) BREAST, RIGHT, ADDITIONAL SUPERIOR MASTECTOMY FLAP TISSUE; EXCISION:
- BENIGN FIBROADIPOSE TISSUE AND SKELETAL MUSCLE.
- NO RESIDUAL CARCINOMA SEEN.

I ATTEST THAT THE ABOVE DIAGNOSIS IS BASED UPON MY PERSONAL EXAMINATION OF THE SLIDES (AND/OR OTHER MATERIAL), AND THAT I HAVE REVIEWED AND APPROVED THIS REPORT.

MD/
*** Report Electronically Signed Out ***

Special Studies:

Result	Special Stain	Comment
positive	ER-C	
positive	PR-C	
negative	HER2-C	intensity of 1+
	NEG CONT	
	NEG-HER2	
	IMM RECUT	
negative	AE1:AE3	
	NEG CONT	
	IMM RECUT	
positive	AE1:AE3	
	NEG CONT	
	IMM RECUT	
negative	AE1:AE3	
	NEG CONT	

** Continued on next page **

negative IMM RECUT
AE1:AE3
NEG CONT
negative IMM RECUT
AE1:AE3
NEG CONT
IMM RECUT

3

Gross Description:

1) The specimen is received fresh, labeled, "Right breast and silicone implant". It consists of product of right mastectomy which measures 14.0 cm from superior to inferior, 14.0 cm from medial to lateral, 5.0 cm from anterior to posterior. There is a skin ellipse with nipple areolar complex which measures 10.0 x 3.5 cm. The nipple is erect. The posterior aspect is inked in black, superior-anterior tissue edge is inked in green, and the inferior-anterior tissue edge is inked in blue. The specimen is serially sectioned from the lateral to medial revealing a stellate vasculated mass in the upper outer quadrant which measures 3.1 x 1.4 x 1.7 cm. It is present 0.2 cm from anterior tissue edge, 0.2 cm from the deep surgical margin. The deep margin is easily movable over the tumor mass. A silicone implant is identified which measures 7.0 x 7.0 x 0.3 cm. It is intact. It is surrounded by fibrous partially calcified capsule.

Summary of Sections:
N nipple
T tumor
UOQ - upper outer quadrant
LOQ - lower outer quadrant
UIQ - upper inner quadrant
LIQ - lower inner quadrant

)

2) The specimen is received fresh for frozen section, labeled "Sentinel node #1 level one right axilla". It consists of one firm brown-tan node measuring 1.0 x 0.5 x 0.4 cm. Bisected and entirely frozen.

Summary of Sections:
FSC - frozen section control

3) The specimen is received fresh for frozen section, labeled "Sentinel node #2 level one right axilla". It consists of a 1.6 x 1.2 x 0.6 cm brown-tan lymph node bisected and entirely frozen.

Summary of Sections:
FSC - frozen section control

** Continued on next page **

4) The specimen is received fresh for frozen section, labeled "Sentinel node #3 level one right axilla". It consists of a 1.7 x 0.6 x 0.6 cm brown-tan node bisected and entirely frozen.

Summary of Sections:
FSC - frozen section control

5) The specimen is received in formalin, labeled "Sentinel node #4 level one right axilla". It consists of a portion of adipose tissue which measures 1.9 x 1.5 x 0.6 cm. Examination reveals a pink-tan lymph node which measures 1.5 x 1.0 x 0.4 cm. The lymph node is bisected and submitted entirely.

Summary of Sections:
SN sentinel node

6) The specimen is received in formalin, labeled "Non-sentinel tissue right axilla". It consists of three irregular shaped portions of yellow adipose which measures 0.6 x 2.5 cm in greatest dimension. The specimen is submitted entirely in one cassette.

Summary of Sections:
NS non-sentinel tissue

7) The specimen is received in formalin, labeled "Sentinel node #5 level one right axilla". It consists of pink-tan portion of soft tissue which measures 0.7 x 0.4 x 0.3 cm. The specimen is submitted entirely.

Summary of Sections:
SN sentinel node

8) The specimen is received in formalin, labeled "Additional superior right mastectomy flap tissue". It consists of two unoriented flap portions of fibrofatty tissue which measure 5.5 x 2.0 x 0.6 cm and 4.5 x3.0 x 0.9 cm. Each piece has a cauterized aspect which is inked in red. The opposing aspect is inked in green. The specimen is serially sectioned and submitted entirely.

Summary of Sections:
U - undesignated

** Continued on next page **

5

Summary of Sections:

Part 1: SP: Right breast and silicone implant

Block	Sect. Site	PCs
1	C	3
1	LIQ	1
1	LOQ	1
1	N	1
5	T	5
1	UIQ	1

Part 2: SP: Sentinel node #1 level one right axilla

Block	Sect. Site	PCs
1	FSC	1

Part 3: SP: Sentinel node #2 level one right axilla

Block	Sect. Site	PCs
1	FSC	1

Part 4: SP: Sentinel node #3 level one right axilla

Block	Sect. Site	PCs
1	FSC	1

Part 5: SP: Sentinel node #4 level one right axilla

Block	Sect. Site	PCs
1	SN	1

Part 6: SP: Non-sentinel tissue right axilla

Block	Sect. Site	PCs
1	NS	1

Part 7: SP: Sentinel node #5 level one right axilla

Block	Sect. Site	PCs
1	SN	1

Part 8: SP: Additional superior right mastectomy flap tissue

Block	Sect. Site	PCs
8	U	8

Procedures/Addenda:
Addendum
Date Ordered:
Date Complete: Status: Signed Out
By:

** Continued on next page **

6

Addendum Diagnosis
ADDENDUM

SITE: SENTINEL LYMPH NODE, RIGHT AXILLA
PART #3.

METASTATIC CARCINOMA IN THE FORM OF MICROSCOPIC CLUSTERS, MEASURING LESS THAN 2MM (MICROMETASTASIS), IS IDENTIFIED ON ADDITIONAL H&E STAINED SECTIONS AND CYTOKERATIN IMMUNOHISTOCHEMICAL STAINS.

ADDITIONAL H&E STAINED SECTIONS AND IMMUNOHISTOCHEMICAL STAINS FOR CYTOKERATINS (AE1:AE3 AND CAM 5.2) ON THE OTHER SENTINEL LYMPH NODE FROM PART #2,4,5,7 SHOW NO EVIDENCE OF METASTATIC TUMOR.

Addendum
Date Ordered:
Date Complete:
Date Reported:

Status: Signed Out
By:

Addendum Diagnosis
ADDENDUM

SITE: RIGHT BREAST
PART #1.

ER: 90% OF NUCLEAR STAINING WITH MODERATE INTENSITY.
PR: 90% OF NUCLEAR STAINING WITH STRONG INTENSITY.
HER2/NEU (HERCEPTEST): NEGATIVE (STAINING INTENSITY OF 1+).

Intraoperative Consultation:

Note: The diagnoses given in this section pertain only to the tissue sample examined at the time of the intraoperative consultation.

2) FROZEN SECTION DIAGNOSIS: BENIGN LYMPH NODES.

PATH
REPORT

** Continued on next page **

PERMANENT DIAGNOSIS: SAME.

3) FROZEN SECTION DIAGNOSIS: BENIGN LYMPH NODES.
PERMANENT DIAGNOSIS: SAME.

4) FROZEN SECTION DIAGNOSIS: BENIGN LYMPH NODES.
PERMANENT DIAGNOSIS: SAME.

71

** End of Report **

PATH
REPORT

ICD-0-3
Carcinoma, infiltrating ductal, NOS 8500/3
Site: breast, NOS C50.9 2/18/11 [illegible]

Case Number :

Collection Date :

Surgical Pathology Report

Diagnosis:
A: Sentinel lymph node #1, right axillary, excision
- Positive for metastatic adenocarcinoma in one of four lymph nodes (1/4)
- Size of metastasis: 0.6 cm
- Extracapsular extension: Present, extensive
- Intraoperative diagnosis confirmed

B: Sentinel lymph node #2, right axillary, excision
- Positive for metastatic adenocarcinoma in one lymph node (1/1) (metastatic focus present on frozen slide only)
- Size of metastasis: 0.1 cm
- Extracapsular extension: Not identified
- Intraoperative diagnosis confirmed

C: Non-sentinel lymph node, right axillary, excision
- Positive for metastatic adenocarcinoma in one lymph node (1/1)
- Size of metastasis: 0.6
- Extracapsular extension: Present, extensive
- Intraoperative diagnosis confirmed

D: Sentinel lymph node #3, right axillary, excision
- Positive for metastatic adenocarcinoma in one lymph node (1/1)
- Size of metastasis: 0.2
- Extracapsular extension: Not identified
- Intraoperative diagnosis confirmed

UUID:5BC01981-366E-44EC-A986-087DB7904E5C
TCGA-AQ-A0Y5-01A-PR Redacted

E: Breast, right, mastectomy

Tumor type: Invasive ductal carcinoma

Nottingham combined histologic grade: 3
Tubule formation score: 3
Nuclear pleomorphism score: 3
Mitotic count score: 3

Focality of tumor: Unifocal

Tumor size (greatest dimension): 2.4 cm (gross measurement)

Tumor location: Outer half (at interface between upper outer and lower outer quadrants)

Lymphovascular invasion: Not identified

Perineural invasion. Present

DQ = ITN

Criteria	Yes	No
Diagnosis Discrepancy		✓
Primary Tumor Site Discrepancy		✓
HIPAA Discrepancy		✓
Prior Malignancy History		✓
Dual/Synchronous Primary Noted		✓
Case is (circle):	QUALIFIED / DISQUALIFIED	
Reviewer Initials KWL for	Date Reviewed: 10/01/10	

In Situ Component: Present, ductal carcinoma in situ

In Situ Component type/Architecture pattern: Ductal carcinoma in situ, solid and comedo types

In Situ Component nuclear grade: 2-3

In Situ Component necrosis: Present

In Situ Component extent/size: DCIS present admixed with invasive ductal carcinoma, comprising approximately 5% of the total tumor volume

Extensive intraductal component: Absent

Nipple/skin involvement: Not identified

Margin status:
Invasive component: Negative; closest posterior margin 0.4 cm; all other margins more than 1.5 cm away
In Situ component: Negative; more than 1.0 cm away from all margins

Right axillary lymph nodes (none present in this specimen, but the total number includes specimens A-D and F):
Total number with metastasis: 5
Total number examined: 17 (5/17)
Size of largest metastasis: 0.6 cm
Extracapsular extension: Present, extensive

Microcalcifications: Present, associated with invasive ductal carcinoma

Other findings: Fibrocystic changes

AJCC PATHOLOGIC TNM STAGE: pT2 pN2a
Note: This pathologic stage assessment is based on information available at the time of this report, and is subject to change pending clinical review and additional information.

F: Lymph nodes, right axillary, lymphadenectomy
- Positive for metastatic adenocarcinoma in 1 of 10 lymph nodes (1/10)
- Size of metastasis: 0.1 cm
- Extracapsular extension: Not identified

Comment:
For the results of immunohistochemical stains for ER, PR and Her2/neu, please refer to the addendum report of the prior specimen

Intraoperative Consult Diagnosis:
Frozen section was requested

FSA1/FSA2: Right axillary sentinel lymph node #1
- Metastatic carcinoma in one lymph node (1/2); Dr agrees.

FSB1: Right axillary sentinel lymph node #2
- Metastatic carcinoma in one lymph node (1/1); Dr agrees.

FSC1: Right axillary non-sentinel lymph node
- Metastatic carcinoma in one lymph node (1/1)

FSD1: Right axillary sentinel lymph node #3
- At least micrometastatic carcinoma in one lymph node

Frozen Section Pathologist:

Clinical History:
invasive ductal Ca, right breast, Grade 2.

Gross Description:
Received are six appropriately labeled containers.

Container A: Received fresh for frozen section is a 3 x 3 x 2 cm aggregate of multiple tan lymph node candidates and adipose tissue. Two larger lymph nodes were isolated, sectioned and frozen as FSA1 and FSA2.

Further examination of the adipose tissue finds four additional lymph node candidates from 5 x 5 x 3 mm to 7 x 4 x 4 mm. All lymph node candidates are submitted as is in block A1. Fibrofatty tissue is retained in formalin.

Container B: Received fresh for frozen section is a 1.5 x 1.0 x 0.5 cm fragment of yellow fibrofatty tissue within which one lymph node candidate was identified at the time of frozen section. The lymph node was serially sectioned and was frozen as FSB1. Fibrofatty tissue is retained in formalin.

Container C: Received fresh for frozen section is a 1.0 x 0.5 x 0.5 cm fragment of pink/yellow fibrofatty lymphoid tissue. The fragment was frozen as FSC1, NTR.

Container D: Received fresh for frozen section is a 1 x 1 x 0.5 cm fragment of yellow lobulated fibrofatty lymphoid tissue. The fragment was serially sectioned, was frozen as FSD1, NTR.

Container E:

Specimen fixation: formalin

Time in fixative: approximately 8 hours, 45 mins

Type of mastectomy: unspecified

Size of specimen: 880 grams; 22 cm medial to lateral x 25.5 cm superior to inferior x 4 cm anterior to posterior

Orientation of specimen: Per the specimen requisition, a short stitch indicates superior and a long stitch indicates lateral.

At the time of Tissue Procurement triage, the specimen is inked as follows: anterior=blue, posterior=black, and lateral=yellow.

Skin ellipse dimensions: 18.0 x 13.5 cm; The lightly pigmented skin ellipse is remarkable for some blue sentinel dye discoloration lateral and slightly inferior to the nipple.

Nipple/areola: Nipple: 1.0 cm
Areola: 2.7 cm
The structures are unremarkable.

Axillary tail: There is no well delineated axillary tail. The lateral most breast tissue is sectioned, however, no lymph node candidates are identified.

Biopsy site: absent

Residual tumor: present, A 2.4 x 2.0 x 1.9 cm firm white mass with a somewhat gritty cut surface and stellate borders is identified. This mass has no hemorrhagic areas or necrotic areas readily identified.

Location of tumor: roughly located in the midline between the upper outer quadrant and the lower outer quadrant

Distance of mass/biopsy site from surgical margin: The mass is focally 6 mm to the black inked posterior margin, 1.5 cm to the skin (anterior margin), 9 cm to the inferior margin, 11 cm to the superior margin, at least 4.5 cm to the lateral margin and distant (at least 14 cm) to the medial margin.

Gross involvement of skin or fascia/muscle by tumor: absent

Description of remainder of breast: The remainder of the mastectomy specimen consists of yellow lobulated fibroadipose tissue separated by thin fibrous septae. Much of the breast consists of dense white fibrous breast tissue. The breast tissue medial to the mass is intensely stained with a blue dye. There are some areas within this firm breast tissue which are firmer than others.

Other remarkable features: On the superior edge of the tumor a small metal radiographic marker is identified.

Tissue submitted for special investigations: Tumor and normal was taken by Tissue Procurement.

Digital photograph taken: no

Block Summary:
(Inking: anterior=blue, posterior=black, lateral=yellow)

E1 - nipple
E2 - mass and closest black inked deep margin, perpendicular sections
E3 - perpendicular sections to closest skin margin (no tumor represented)
E4-E8 - additional tumor (E7,E8 one slice, bisected)
E9-E10 - breast tissue immediately surrounding mass
E11 - inner upper quadrant
E12 - inner lower quadrant
E13 - outer upper quadrant
E14 - outer lower quadrant
E15-E16 - lateral breast tissue

Container F is additionally labeled "right axillary contents." It holds a 12 x 9.5 x 3.0 cm aggregate of multiple fragments of yellow lobulated fibroadipose tissue which are palpated for lymph node candidates. Multiple lymph node candidates from 4 x 4 x 3 mm to 1.4 x 1.0 x 0.6 cm are identified.

Block summary;

F1 - five lymph node candidates
F2 - two lymph node candidates
F3 - one lymph node candidate, serially sectioned
F4 - one lymph node candidate, sectioned
F5 - one lymph node candidate, sectioned
F6-F7 - one lymph node candidate, sectioned
F8 - one lymph node candidate, sectioned
F9 - one lymph node candidate, sectioned

Light Microscopy:
Light microscopic examination is performed.

Signature
I have personally conducted the evaluation of the above specimens and have rendered the above diagnosis(es).

UUID:6061FCA5-A113-4985-905D-E17020F6933E
TCGA-AR-A0TQ-01A-PR Redacted

ICD-0-3
carcinoma, infiltrating duct, NOS 8500/3
Site: breast, NOS C50.9

Breast, right, simple mastectomy: Infiltrating ductal carcinoma, Nottingham grade III (of III) [tubules 3/3, nuclei 3/3, mitoses 2/3; Nottingham score 8/9], forming a single mass (7.2 x 4.5 x 3.4 cm) located in the upper inner and upper central quadrant of the breast [AJCC pT2]. Angiolymphatic invasion is absent. The non-neoplastic breast parenchyma shows nonproliferative fibrocystic changes. Calcifications are present in malignant ducts. Biopsy site changes are present. All surgical resection margins, including the deep margin, are negative for tumor (minimum tumor free margin, 7.0 cm, deep margin).

Breast, right, supranumerary nipple excision: Intradermal nevus (0.6 cm in greatest dimension).

Lymph nodes, right axillary, dissection: Multiple (2 of 9) right axillary lymph nodes are positive for metastatic carcinoma. The largest lymph node measures 1.4 cm. No extranodal extension identified.

Lymph nodes, highest right axillary, dissection: Multiple (6) highest right axillary lymph nodes are negative for tumor.

Breast, left, simple mastectomy: Nonproliferative fibrocystic changes.

Her-2/NEU has been ordered on paraffin-embedded tissue.

Criteria	Yes	No
Diagnosis Discrepancy		X
Primary Tumor Site Discrepancy		X
HIPAA Discrepancy		X
Prior Malignancy History		X
Dual/Synchronous Primary Noted		X
Case is (circle): QUALIFIED / DISQUALIFIED		
Reviewer Initials [illegible] Date Reviewed: 9/1/10		

UUID:48F86416-8304-479E-B477-7C37A2F951A2
TCGA-AR-A0TZ-01A-PR Redacted

ICD-0-3
Carcinoma, infiltrating ductal, NOS 8500/3
Site: breast, NOS C50.9

Breast, left, modified radical mastectomy: Invasive ductal carcinoma, Nottingham grade II, is identified forming multiple (5) masses in the left breast. The largest mass is situated in the upper outer quadrant and measures 3.2 x 2.7 x 2.3 cm (AJCC pT2). Additional nodules are situated inferior and just superior to the main mass and range in size from 0.5 cm to 0.8 cm in diameter. There is approximately 10% component of ductal carcinoma in situ, cribriform type, intermediate nuclear grade. Skin, nipple, and deep margin are free of neoplasm. Multiple (6 of 30) left axillary lymph nodes are positive for metastatic carcinoma, including multiple (2) matted lymph nodes (AJCC pN2).

HER2/neu has been ordered on paraffin embedded tissues.

Criteria	Yes	No
Diagnosis Discrepancy		X
Primary Tumor Site Discrepancy		X
HIPAA Discrepancy		X
Prior Malignancy History		X
Dual/Synchronous Primary Noted		X
Case is (circle): QUALIFIED / DISQUALIFIED		
Reviewer Initials [illegible] Date Reviewed: 9/1/10		

UUID:007C609A-7874-4E39-BC4D-1EA81F4722EA
TCGA-AR-A0U1-01A-PR Redacted

ICD-O-3
Carcinoma, infiltrating ductal, NOS 8500/3
Site: breast, NOS C50.9

Breast, left, wide local excision: Invasive ductal carcinoma, Nottingham grade 3 (of 3), forming a 2.3 x 2.2 x 2.0 cm mass (AJCCpT2). All surgical margins, including separately submitted inferior margin, are negative for tumor.

Lymph node, left breast intramammary, excision: Metastatic adenocarcinoma. no extranodal extension is identified.

Lymph nodes, left axillary sentinel, excisions: Multiple (5) left axillary sentinel lymph nodes are negative for tumor (blue dye identified in left axillary lymph node No. 1 only).

Lymph nodes, left axillary, dissection: Multiple left highest (2) and left axillary (23) lymph nodes are negative for tumor.

Her-2/NEU has been ordered on paraffin embedded tissue.

Criteria	Yes	No
Diagnosis Discrepancy		X
Primary Tumor Site Discrepancy		X
HIPAA Discrepancy		X
Prior Malignancy History		X
Dual/Synchronous Primary Noted		X
Case is (circle): QUALIFIED / DISQUALIFIED		
Reviewer Initials [illegible] Date Reviewed: 9/1/10		

UUID:51F69645-9564-4257-A86E-3A574C27034F
TCGA-AR-A0U2-01A-PR **Redacted**

ICD-O-3
carcinoma, infiltrating ductal, NOS 8500/3
Site: breast, NOS C50.9

Breast, left, radical mastectomy: Infiltrating ductal carcinoma, Nottingham grade III (of III) forming a 3.2 x 2.2 x 1.6 cm mass (AJCC pT2) adjacent to the biopsy cavity in the central breast. Two separate microscopic foci (>2.0 mm) of invasive carcinoma are present in subareolar tissue in association with high grade (nuclear grade 3) ductal carcinoma in situ. Angiolymphatic invasion is identified. The surgical resection margins are negative for tumor.

Lymph nodes, left axillary, lymphadenectomy: Metastatic adenocarcinoma, consistent with breast origin, is identified involving multiple (4 of 16) axillary lymph nodes. The largest metastatically involved node measures 3.0 cm in maximum dissension and shows extracapsular tumor extension (AJCC pN2).

Fallopian tubes and ovaries, right and left, salpingo-oophorectomy: Fallopian tubes and ovaries showing no diagnostic abnormalities.

Estrogen and progesterone receptor analysis and her2-Neu have been ordered on paraffin embedded tissue.

Criteria	Yes	No
Diagnosis Discrepancy		X
Primary Tumor Site Discrepancy		X
HIPAA Discrepancy		X
Prior Malignancy History		X
Dual/Synchronous Primary Noted		
Case is (circle): QUALIFIED / DISQUALIFIED		
Reviewer Initials: [illegible] Date Reviewed: 9/1/10		

UUID:AD07F611-0EEA-4890-A02C-6DA3F5F57C45
TCGA-AR-A24Z-01A-PR Redacted

Criteria	Yes	No
Diagnosis Discrepancy		✓
Primary Tumor Site Discrepancy		✓
HIPAA Discrepancy		✓
Prior Malignancy History		✓
Dual/Synchronous Primary Noted		✓
Case is (circle): (QUALIFIED) / DISQUALIFIED		
Reviewer Initials RB Date Reviewed: 4/20/11		
lw	4/25/11	

Final Diagnosis

Breast, left, total mastectomy: Infiltrating ductal carcinoma, Nottingham grade II (of III), [tubules 2/3, nuclei 2/3, mitoses 3/3; Nottingham score 7/9], forming a 4.2 x 4.0 x 3.0 cm mass located in the upper outer quadrant of the breast [AJCC pT2]. Focal ductal carcinoma in situ, intermediate nuclear grade, comprising approximately 5% of tumor volume. The non-neoplastic breast parenchyma shows nonproliferative fibrocystic changes. Biopsy site changes present. The tumor does not involve the nipple, overlying skin, or underlying chest wall. All surgical resection margins, including deep margin, are negative for tumor (minimum tumor free margin, 1.5 cm, anterior margin).

Lymph nodes, left axillary sentinel, excision: Multiple (8) left axillary sentinel lymph nodes are negative for tumor (AJCCpN0(i-)(sn)). Blue dye was identified in lymph node Nos. 2A, 2B, and 2C. No blue dye was identified in lymph node Nos. 1, 3, 4, 5, and 6. Immunohistochemical cytokeratin stain was performed on the paraffin embedded sentinel lymph node tissue and confirms the H&E impression.

Estrogen and progesterone receptor analysis and HER2/neu have been ordered on paraffin embedded tissues.

ICD-0-3
carcinoma, infiltrating duct, NOS 8500/3
Site: breast, NOS C50.9 lw
4/25/11

Criteria	Yes	No
Diagnosis Discrepancy		✓
Primary Tumor Site Discrepancy		✓
HIPAA Discrepancy		✓
Prior Malignancy History		✓
Dual/Synchronous Primary Noted		✓
Case is (circle): QUALIFIED / DISQUALIFIED		
Reviewer Initials RB	Date Reviewed: 4/21/11	

fw 4/25/11

UUID:0FDC2801-71F8-4D45-BFF1-89445E621A41
TCGA-AR-A256-01A-PR
Redacted

Final Diagnosis

Breast, right, mastectomy: Infiltrating ductal carcinoma, Nottingham grade III (of III), forming a 2.5 x 2.5 x 2 cm mass. Ductal carcinoma in-situ is not identified (AJCC pT2). Surgical margins and nipple are negative for tumor.

Lymph nodes, right sentinel axillary (No. 1 and No. 2) and right axillary, excision: Multiple (3) right sentinel axillary lymph nodes and (2) right axillary lymph nodes are negative for tumor.

Breast, right, biopsy: Small fragment of skin and adipose tissue, negative for tumor.

Breast, left, mastectomy: Proliferative fibrocystic changes characterized by fibrosis, sclerosing adenosis and cyst formation. A single 0.5 cm fibroadenoma with myxoid degeneration is identified.

Immunohistochemical studies with antibodies against cytokeratin show the sentinel lymph nodes #1 and #2 to be negative for metastatic carcinoma. Immunohistochemical stains for estrogen and progesterone receptors are negative in neoplastic cells.

ICD-0-3
carcinoma, infiltrating duct, NOS 8500/3
Site: breast, NOS C50.9
fw
4/25/11

Carcinoma, infiltrating ductal, NOS - 8500/3
Path: Site Code: Breast, upper outer quadrant C50.4

Patient: 1/17/11

CQCF: breast, NOS C50.9

Surgica[redacted]

Surg Path

UUID:F2B21F52-74F4-44C5-9991-60EBCC4E43A6
TCGA-B6-A0RI-01A-PR Redacted

CLINICAL HISTORY:
Not provided.

GROSS EXAMINATION:
Estrogen receptor tissue sent for estrogen and progesterone receptor.
A. "Tru-cut breast biopsy.": Received fresh. The specimen is a 1 cm x 0.1 cm tan piece of breast tissue. This frozen as frozen section AF1. The frozen section remnant consists of two pieces of tan tissue measuring 0.8 x 0.1 x 0.1 and 0.7 x 0.1 x 0.1 cm. They are submitted in toto in Block A1.

B. "Right breast.": Received fresh. The specimen consists of breast with an overlying ellipse of skin. The overall dimensions of the specimen are 15 x 11 x 2 cm with an attached axillary tail which in addition measures 7.8 x 3.5 cm. The skin ellipse measures 11.5 x 4 cm in greatest dimensions. And contains a nipple at its center. The deep surgical margin is inked blue and the lateral surgical margins are inked black. Several sections have been made through the deep aspect of of the specimen revealing a 1.5 x 2.8 x 1.0 cm firm mass which radially retracts the adjacent breast tissue and which is located approximately 0.2 cm from the deep surgical margin at one point. The mass appears located in the upper outer quadrant of the breast. Further sectioning through the specimen reveals unremarkable breast parenchyma in areas away from the tumor. Material submitted for ER/PR.

Block Summary:
B1 and B2-Closest sections of tumor to the deep surgical margin inked blue.
B3 and B4-Further sections of tumor to the deep surgical margin.
B5-Section through the nipple.
B6-Representative sampling from the upper inner quadrant.
B7-Representative sampling of the lower inner quadrant.
B8-Representative sampling of the upper outer quadrant.
B9-Representative sampling of the lower outer quadrant.
B10-Further sections through the lower outer quadrant.

The axillary tail is dissected. Tumor margin has been marked with surgical suture. These are present in zone 2 at the juncture with zone 1 and are submitted separately in Block B11.

B12-Sections of fat from zone 1 appear that no definite lymph nodes are identified in zone 1.
B13-Candidate lymph nodes from zone 2.
B14, 15 and B16-Candidate lymph nodes from zone 3.

INTRA OPERATIVE CONSULTATION:
AF1 "Tru-cut breast biopsy": infiltrating ductal carcinoma.

Criteria	Yes	No
Diagnosis Discrepancy		X
Primary Tumor Site Discrepancy		X
HIPAA Discrepancy		X
Prior Malignancy History		X
Dual/Synchronous Primary Noted		X
Case is (circle): QUALIFIED / DISQUALIFIED		
Reviewer Initials: KM Date Reviewed: 1/14/11		

DIAGNOSIS:
A. "TRU CUT BREAST BIOPSY":

INFILTRATING DUCTAL CARCINOMA.

B. "RIGHT BREAST":

1. INFILTRATING DUCTAL CARCINOMA, NSABP HISTOLOGIC GRADE 3, NUCLEAR GRADE MODERATELY DIFFERENTIATED. THE TUMOR COMES WITHIN 0.2 CM OF THE DEEP MARGIN GROSSLY. THE TUMOR SIZE IS 1.5 X 2.8 X 1.0 CM. VASCULAR INVASION IS PRESENT. THE TUMOR IS LOCATED IN THE UPPER OUTER QUADRANT ONLY.

2. SIX OF FIFTEEN LYMPH NODES CONTAIN METASTATIC CARCINOMA.

Verified by:

Carcinoma, infiltrating ductal, NOS 8500/3
Site Code: breast, NOS C50.9
1/7/11

Patien

Surgical F

UUID:EFA0354A-641D-4EC2-A1BE-4ACC7CE421DF
TCGA-B6-A0RO-01A-PR Redacted

Surg Path

CLINICAL HISTORY:
Right breast Ca, rule out deep margin positive.

GROSS EXAMINATION:
A. "#1 right modified radical mastectomy", in formalin. Received in the container is a portion of breast with an attached skin ellipse containing the nipple and an axillary dissection. The specimen measures 18.5 x 14 x 3.9 cm. The attached skin ellipse measures 14.7 x 6.3 cm. The nipple appears grossly unremarkable. There is a small 0.5 cm hole 1.7 cm inferior to the nipple. The deep surface of the specimen is inked in blue and the specimen is serially sectioned revealing a 3.5 x 2 x 1.5 cm firm white mass in the medial portion of the specimen. The mass is fairly well-circumscribed but very firm to palpation. Grossly it appears to approach within 0.2 cm of the deep surgical margin. There is no definite biopsy cavity; however, there is a small area of hemorrhage just adjacent to the tumor mass. Tissue from the tumor mass has been sent for estrogen and progesterone receptors.

Block Summary:
A1- section through nipple.
A2- section through hole in skin ellipse.
A3-A5- sections of tumor with adjacent deep margin.
A6-A9- representative sections of tumor.
A10- representative sections of upper lateral quadrant.
A11- representative section of lower lateral quadrant.
A12- representative section of upper medial quadrant.
A13- five lymph node candidates from level three.
A14-A15- one large lymph node bisected, level three.
A16- one large lymph node bisected and one small lymph node candidate, level three.
A17- one lymph node candidate bisected, level three.
A18- one lymph node candidate bisected, level three.
A19- four lymph node candidates from level II.
A20- one lymph node candidate bisected from level II.
A21- two lymph node candidates from level II.
A22- five lymph node candidates from level I.
A23- two lymph node candidates from level I.
A24- two lymph node candidates from level I.
A25- two lymph node candidates from level I.

Criteria	Yes	No
Diagnosis Discrepancy		X
Primary Tumor Site Discrepancy		X
HIPAA Discrepancy		X
Prior Malignancy History		X
Dual/Synchronous Primary Noted		X
Case is (circle): QUALIFIED / DISQUALIFIED		
Reviewer Initials [illegible] Date Reviewed: 1/3/11		

MICROSCOPIC EXAMINATION:
Most of the tumor consists of tubules and cribriform glands composed of atypical cells with a moderate amount of eosinophilic cytoplasm and oval nuclei with distinct relatively small nucleoli. There is a sparse inflammatory response. A minor non-comedo intraductal component is present, within or near the tumor mass. Away from the tumor, no intraductal carcinoma or epithelial hyperplasia is seen.

DIAGNOSIS:
A. "RIGHT" BREAST, MODIFIED RADICAL MASTECTOMY:

INFILTRATING DUCTAL CARCINOMA (2.5 CM), NSABP HISTOLOGIC GRADE II/III, NUCLEAR GRADE MODERATELY DIFFERENTIATED.

A NON-COMEDO INTRADUCTAL CARCINOMA COMPONENT IS IDENTIFIED, COMPRISING LESS THAN 10% OF THE CARCINOMA.

NO TUMOR IS SEEN IN THE DEEP SURGICAL MARGIN.

NO VASCULAR INVASION IS IDENTIFIED.

TWO OF SEVENTEEN (2/17) LYMPH NODES ARE POSITIVE FOR METASTATIC ADENOCARCINOMA.

BREAST TISSUE WITH APOCRINE METAPLASIA AND MICROCALCIFICATIONS IN NON-NEOPLASTIC LOBULES.

Verified by

ICD-O-3
Carcinoma, infiltrating ductal, NOS 8500/3
Path: Site: breast, upper inner quadrant C50.2
[redacted] CQF: breast, NOS C50.9
1/25/11 lw

urgical Pathology: Add [redacted]

Surg Path

UUID:F05F5886-DC5D-4685-B2BF-57A68A0BB7B9
TCGA-B6-A0WW-01A-PR
Redacted

CLINICAL HISTORY:
Cervical cancer and breast cancer.

GROSS EXAMINATION:
A. "True cut breast biopsy", AF1. Frozen tissue remnant labeled AF1 submitted in toto in block A1.

B. "Cervical tumor", tissue fragment labeled BF1 in submitted in toto in block B1. A 4.2 x 3 x 2.3 cm aggregate of tan tissue containing multiple edematous papillary structures. Representative sections are submitted in blocks B2 and B3. A representative sample of the specimen is placed in a tea bag for gross photography.

C. "Right breast and axillary nodes", received unfixed and placed in formalin. A sample of fresh tissue has been sent for ER/PR. A 580 gm, 28.4 x 15.3 x 3.9 cm modified radical mastectomy specimen containing a 22.0 x 15.3 x 3.9 cm breast with an 8.5 x 5.8 x 2 cm axillary tail, and a 2 cm nipple within a 5.5 cm areola. The specimen is remarkable for a 4.1 x 2.6 x 4 cm white hard mass located within the upper inner portion of the breast 1.1 cm from the inked deep margin and 1.7 cm from the skin surface. The axillary tail contains multiple firm matted lymph nodes within the proximal mid and distal portions, the largest measuring 3.5 x 1.4 x 1 cm. The breast also contains a soft pale pink 5 x 1.2 x 1.5 cm. The axillary tail is removed and dissected for lymph nodes.

BLOCK SUMMARY:
C1-C2 - tumor with soft tissue margins.
C3-C4 - random soft tissue margins
C5-C7 - tumor
C8-C9 - unremarkable breast tissue
C10-C11 - representative sections of skin
C12 - section of areola
C13 - section of nipple
C14 - one bisected lymph node candidate from the proximal axillary lymph nodes
C15 - three lymph node candidates from the proximal axillary lymph nodes
C16 - six lymph node candidates from the mid axillary lymph nodes
C17 - one bisected lymph node candidate from mid axillary lymph nodes
C18 - two lymph node candidates from the distal axillary lymph nodes
C19 - one bisected lymph node candidate from the distal portion of axillary lymph nodes

Dr. /Dr. /Slides to Dr.

INTRA OPERATIVE CONSULTATION:
A. "True cut breast", AF1: invasive carcinoma (Dr.

B. "Cervical tumor", BF1: papillary squamous cell carcinoma in situ at least (Dr.

DIAGNOSIS:
A. "TRUE CUT BIOPSY":

INFILTRATING DUCTAL CARCINOMA.
N.S.A.B.P. NUCLEAR GRADE 2 OF 3.
N.S.A.B.P. HISTOLOGIC GRADE 3 OF 3.
LYMPHATIC/VASCULAR INVASION PRESENT.

Criteria	Yes	No
Diagnosis Discrepancy		X
Primary Tumor Site Discrepancy		X
HIPAA Discrepancy		X
Prior Malignancy History		X
Dual/Synchronous Primary Noted		X
Case is (circle): QUALIFIED / DISQUALIFIED	✓	
Reviewer Initials [illegible] KMT lw	Date Reviewed: [illegible]	

https:/

B. "CERVICAL TUMOR":

POLYPOID PAPILLARY SQUAMOUS CELL CARCINOMA IN-SITU WITH EQUIVOCAL EARLY STROMAL INVASION. (SEE COMMENT).

C. "RIGHT BREAST AND AXILLARY NODES" (MODIFIED RADICAL MASTECTOMY):

RESIDUAL INFILTRATING DUCTAL CARCINOMA.
N.S.A.B.P. NUCLEAR GRADE 2 OF 3.
N.S.A.B.P. HISTOLOGIC GRADE 3 OF 3.
GROSS TUMOR SIZE 4.1 X 2.6 X 4.0 CM (GROSSLY).
SIZE OF INVASIVE COMPONENT 4.1 CM.
LOCATION OF THE TUMOR, UPPER INNER QUADRANT.
LYMPHATIC/VASCULAR INVASION PRESENT.
MULTIFOCAL TUMOR NO.

IN SITU CARCINOMA PRESENT, OCCUPYING APPROXIMATELY 5% OF TUMOR.
TYPE OF IN-SITU CARCINOMA CRIBRIFORM WITH NECROSIS AND SOLID TYPES (SLIDES C4, C6, C7)
EXTENSIVE INTRADUCTAL COMPONENT, NO.

NIPPLE STATUS, FREE OF TUMOR.
SKIN STATUS, FREE OF TUMOR.
MUSCLE STATUS, NOT SAMPLED.
STATUS OF NON-NEOPLASTIC BREAST TISSUE: FIBROSIS.
SURGICAL MARGIN STATUS: NEGATIVE.

LYMPH NODE STATUS: METASTATIC CARCINOMA IN EIGHT OF 15 RIGHT AXILLARY LYMPH NODES.
SIZE OF LARGEST LYMPH NODE METASTASIS 1.5 CM (SLIDE C14).
EXTRANODAL INVASION PRESENT (SLIDE C14).

ESTROGEN/PROGESTERONE RECEPTOR AND CELL CYCLE ANALYSIS PENDING.
METHODOLOGY: IMMUNOHISTOCHEMISTRY, PARAFFIN BLOCK (C6).

COMMENT: The lesion displays in-situ form of cancer that architecturally resembles the papillary transitional cell cancer, grade 1, commonly seen in the bladder. In multiple areas, the base of the epithelium is cut on a bais, so that it cannot be determined whether the few cells seen in the superficial stroma are artifactual or the earliest form of microinvasion. While such a lesion, if seen in the bladder, would be called low grade, there is no recorded experience with such a neoplasm in the cervix.
Dr. has reviewed the slides of the cervical tumor and concurs with the diagnosis.

I certify that I personally conducted the diagnostic evaluation of the above specimen(s) and have rendered the above diagnosis(es).

, M.D. Page#

Electronically signed:

ADDENDUM 2:

Breast tissue was sent to the _ , for assay of the estrogen and progesterone receptors. The estrogen receptor activity was judged to be POSITIVE with an estimated FMOL value of 99. The progesterone receptor activity was judged as POSITIVE with an estimated FMOL value of 15. Please refer to for a complete report.

I certify that I personally conducted the diagnostic evaluation of the above specimen(s) and have rendered the above diagnosis(es).

http

FINAL DIAGNOSIS:

PART 1: LYMPH NODE, LEFT BREAST, #1 SENTINEL, EXCISION –
NO EVIDENCE OF METASTATIC MAMMARY CARCINOMA IN ONE LYMPH NODE (0/1).

PART 2: LYMPH NODE, LEFT BREAST, #2 SENTINEL, EXCISION –
NO EVIDENCE OF METASTATIC MAMMARY CARCINOMA IN ONE LYMPH NODE (0/1).

PART 3: BREAST, LEFT, SEGMENTAL MASTECTOMY –

A. INVASIVE DUCTAL CARCINOMA.
B. NOTTINGHAM GRADE I (TUBULE FORMATION: 2, NUCLEAR PLEOMORPHISM: 2, MITOTIC ACTIVITY: 1; TOTAL SCORE 5/9).
C. INVASIVE TUMOR MEASURES 1.8 CM IN LARGEST DIMENSION.
D. NO LYMPHOVASCULAR SPACE INVASION IS IDENTIFIED.
E. INVASIVE CARCINOMA IS 0.8 CM FROM THE NEAREST ANTERIOR MARGIN.

SYNOPTIC - PRIMARY INVASIVE CARCINOMA OF BREAST

LATERALITY: Left
PROCEDURE: Segmental
LOCATION: Not specified
SIZE OF TUMOR: Maximum dimension invasive component: 1.8 cm
MULTICENTRICITY/MULTIFOCALITY OF INVASIVE FOCI:
No
TUMOR TYPE (invasive component):
Ductal adenocarcinoma, NOS
NOTTINGHAM SCORE: Nuclear grade: 2
Tubule formation: 2
Mitotic activity score: 1
Total Nottingham score: 5
Nottingham grade (1, 2, 3): 1

ICD-0-3
Carcinoma, infiltrating ductal, NOS 8500/3
Site: breast, NOS C50.9 3/13/11

ANGIOLYMPHATIC INVASION: No
DERMAL LYMPHATIC INVASION: No
CALCIFICATION: No
SURGICAL MARGINS INVOLVED BY INVASIVE COMPONENT:
No
Distance of invasive tumor to closest margin: 8 mm
PAGET'S DISEASE OF NIPPLE: No
LYMPH NODES POSITIVE: 0
LYMPH NODES EXAMINED: 2
METHOD(S) OF LYMPH NODE EXAMINATION:
H/E stain
SENTINEL NODE METASTASIS: No
NON-NEOPLASTIC BREAST TISSUE: FCD
T STAGE, PATHOLOGIC: pT1c
N STAGE, PATHOLOGIC: pN0
M STAGE, PATHOLOGIC: pMX
ESTROGEN RECEPTORS: positive
PROGESTERONE RECEPTORS: positive
HER2/NEU: zero or 1+

UUID:EB09D890-879C-4874-AE3F-47E41C2C66FE
TCGA-BH-A0B0-01A-PR Redacted

Criteria	Yes	No
Diagnosis Discrepancy		✓
Primary Tumor Site Discrepancy		✓
HIPAA Discrepancy		✓
Prior Malignancy History		✓
Dual/Synchronous Primary Noted		
Case is (circle): QUALIFIED / DISQUALIFIED		
Reviewer Initials [illegible] Date Reviewed: 3/13/11		

[illegible] 3/13/11

FINAL DIAGNOSIS:

PART 1: LEFT BREAST, SEGMENTAL MASTECTOMY AT 3 O'CLOCK –

A. TWO FOCI OF INFILTRATING DUCTAL CARCINOMA, 0.7 AND 1.5 CM, NOTTHINGHAM SCORE 6/9 (TUBULES 3, NUCLEAR 2, MITOSES 1).
B. TUMOR AGGREGATE 2.2 CM.
C. DUCTAL CARCINOMA IN SITU WITH COMEDO NECROSIS ASSOCIATED WITH MICROCALCIFICATION PRESENT IN BOTH FOCI OF TUMOR AND REPRESENTING 10% OF THE TUMOR VOLUME.
D. LYMPHOVASCULAR SPACE INVOLVEMENT IS NOT PROMINENT.
E. 1ND INVASIVE TUMOR IS 0.2 CM FROM THE ANTERIOR MARGIN AND DCIS IS 0.1 CM FROM ANTERIOR MARGIN.
F. 2ND INVASIVE TUMOR IS 0.1 CM FROM THE POSTERIOR MARGIN.
G. MARGINS FREE OF TUMOR.
H. FIBROCYSTIC CHANGES WITH ATYPICAL DUCTAL EPITHELIAL HYPERPLASIA, SCLEROSING ADENOSIS ASSOCIATED WITH MICROCALCIFICATION.
I. MICROSCOPIC RADIAL SCAR, CONFIRMED BY POSITIVE P63 AND SMOOTH MUSCLE MYOSIN HEAVY CHAIN IMMUNOSTAIN.

ICD-O-3
Carcinoma, infiltrating duct, NOS 8500/3
Site: breast, NOS C50.9 3/13/11

PART 2: LEFT AXILLA, SENTINEL LYMPH NODE #1, BIOPSY – TWO LYMPH NODES NEGATIVE FOR TUMOR.

PART 3: LEFT AXILLA, SENTINEL LYMPH NODE #2, BIOPSY – ONE LYMPH NODE NEGATIVE FOR TUMOR.

CASE SYNOPSIS:

SYNOPTIC - PRIMARY INVASIVE CARCINOMA OF BREAST

LATERALITY:	Left
PROCEDURE:	Segmental
LOCATION:	Upper outer quadrant
SIZE OF TUMOR:	Maximum dimension invasive component: 1.5 cm
MULTICENTRICITY/MULTIFOCALITY OF INVASIVE FOCI:	Yes
TUMOR AGGREGATE SIZE:	Sum of the sizes of multiple invasive tumors: 2.2 cm
TUMOR TYPE (invasive component):	Ductal adenocarcinoma, NOS
NOTTINGHAM SCORE:	Nuclear grade: 2<br>Tubule formation: 3<br>Mitotic activity score: 1<br>Total Nottingham score: 6<br>Nottingham grade (1, 2, 3): 2
ANGIOLYMPHATIC INVASION:	No
DERMAL LYMPHATIC INVASION:	Not applicable
CALCIFICATION:	Yes, benign zones<br>Yes, malignant zones
TUMOR TYPE, IN SITU:	Solid<br>Comedo<br>DCIS admixed with invasive carcinoma<br>Percent of tumor occupied by in situ component: 10 %
SURGICAL MARGINS INVOLVED BY INVASIVE COMPONENT:	No<br>Distance of invasive tumor to closest margin: 1 mm
SURG MARGINS INVOLVED BY IN SITU COMPONENT:	No<br>Distance of in situ disease to closest margin: 1 mm
LYMPH NODES POSITIVE:	0
LYMPH NODES EXAMINED:	3
METHOD(S) OF LYMPH NODE EXAMINATION:	H/E stain
SENTINEL NODE METASTASIS:	No
NON-NEOPLASTIC BREAST TISSUE:	ADH, Radical scar, FCD
T STAGE, PATHOLOGIC:	pT1c
N STAGE, PATHOLOGIC:	pN0
M STAGE, PATHOLOGIC:	pMX
ESTROGEN RECEPTORS:	previously performed
PROGESTERONE RECEPTORS:	previously performed
HER2/NEU:	zero or 1+

UUID:2444E1FE-E0AB-4AA0-9081-967426B90EE6
TCGA-BH-A0BP-01A-PR Redacted

[redacted]

Criteria	Yes	No
Diagnosis Discrepancy		✓
Primary Tumor Site Discrepancy		✓
HIPAA Discrepancy		✓
Prior Malignancy History		✓
Dual/Synchronous Primary Noted		✓
Case is (circle): QUALIFIED / DISQUALIFIED		
Reviewer Initials [illegible] Date Reviewed: 3/13/11		

lw 3/13/11

[redacted]

FINAL DIAGNOSIS:

PART 1: LYMPH NODE, RIGHT SENTINEL #1, EXCISION –
ONE (1) LYMPH NODE, NEGATIVE FOR METASTATIC ADENOCARCINOMA (0/1).

PART 2: LYMPH NODE, RIGHT SENTINEL #2, EXCISION –
A. ONE (1) LYMPH NODE, POSITIVE FOR MICROMETASTASIS TWO (2) FOCI MEASURING LESS THAN 1.0 MM, AND ONE (1) ALSO LESS THA 1.0 MM (1/1) (see comment).
B. NO EXTRACAPSULAR SPREAD IS SEEN.

PART 3: LYMPH NODE, RIGHT SENTINEL #3, EXCISION –
ONE (1) LYMPH NODE, NEGATIVE FOR METASTATIC ADENOCARCINOMA (0/1).

ICD-0-3
Carcinoma, infiltrating ductal, NOS 8500/3
Site: breast, NOS C50.9 3/13/11 [illegible]

PART 4: LYMPH NODE, RIGHT NON-SENTINEL, EXCISION –
ONE (1) LYMPH NODE, NEGATIVE FOR METASTATIC ADENOCARCINOMA (0/1).

PART 5: BREAST, RIGHT, SEGMENTAL MASTECTOMY –
A. INFILTRATING DUCTAL CARCINOMA NOS
B. NOTTINGHAM GRADE 1, SCORE OF 5/9 (TUBULES = 2, NUCLEI = 2, MITOSIS = 1).
C. TUMOR MEASURES 2.0 CM ON GROSS EXAMINATION.
D. DUCTAL CARCINOMA IN-SITU, CRIBRIFORM TYPE AND WITH COMEDO NECROSIS, NUCLEAR GRADE 2, ADMIXED WITH INVASIVE COMPONENT, COMPRISING APPROXIMATELY LESS THAN 5% OF THE TOTAL TUMOR VOLUME.
E. SURGICAL MARGINS OF RESECTION ARE FREE OF INVASIVE TUMOR.
F. CLOSEST POSTERIOR AND SUPERIOR MARGINS OF RESECTION ARE AT 0.6 CM.
G. MARGINS OF RESECTION ARE FREE OF DUCTAL CARCINOMA IN-SITU, CLOSEST POSTERIOR MARGIN AT 1.2 CM.
H. ANGIOLYMPHATIC INVASION IS IDENTIFIED.
I. ESTROGEN RECEPTOR POSITIVE, PROGESTERONE RECEPTOR POSITIVE, HER-2/NEU NEGATIVE (SCORE 0), PER PREVIOUS REPORT
J. SKIN WITH NO SIGNIFICANT PATHOLOGIC ABNORMALITY.
K. NON-NEOPLASTIC BREAST WITH FIBROCYSTIC CHANGES, DUCTAL EPITHELIAL HYPERPLASIA, AND FIBROADENOMATOID NODULES.

PART 6: BREAST, RIGHT MEDIAL NEW MARGIN, EXCISION –
BREAST TISSUE, NEGATIVE FOR TUMOR.

PART 7: BREAST, RIGHT INFERIOR NEW MARGIN, EXCISION –
A. FIBROADIPOSE TISSUE NEGATIVE FOR TUMOR.
B. FRAGMENTS OF SKELETAL MUSCLE.

CASE SYNOPSIS:

SYNOPTIC - PRIMARY INVASIVE CARCINOMA OF BREAST

LATERALITY:	Right
PROCEDURE:	Segmental
LOCATION:	Not specified
SIZE OF TUMOR:	Maximum dimension invasive component: 2.0 cm
MULTICENTRICITY/MULTIFOCALITY OF INVASIVE FOCI:	No
TUMOR TYPE (invasive component):	Ductal adenocarcinoma, NOS
NOTTINGHAM SCORE:	Nuclear grade: 2<br>Tubule formation: 2<br>Mitotic activity score: 1<br>Total Nottingham score: 5<br>Nottingham grade (1, 2, 3): 1
ANGIOLYMPHATIC INVASION:	Yes
DERMAL LYMPHATIC INVASION:	No
CALCIFICATION:	No
TUMOR TYPE, IN SITU:	Cribriform<br>DCIS admixed with invasive carcinoma<br>Percent of tumor occupied by in situ component: 5 %
SURGICAL MARGINS INVOLVED BY INVASIVE COMPONENT:	No<br>Distance of invasive tumor to closest margin: 6 mm
SURG MARGINS INVOLVED BY IN SITU COMPONENT:	No<br>Distance of in situ disease to closest margin: 12 mm
LYMPH NODES POSITIVE:	1
LYMPH NODES EXAMINED:	4
METHOD(S) OF LYMPH NODE EXAMINATION:	H/E stain
SENTINEL NODE METASTASIS:	Yes
ONLY KERATIN POSITIVE CELLS ARE PRESENT:	No
SIZE OF NODAL METASTASES:	Diameter of largest lymph node metastasis: 1 mm
LYMPH NODE METASTASIS(-ES) WITH EXTRACAPSULAR EXTENSION:	No
SKIN INVOLVED (ULCERATION):	No
NON-NEOPLASTIC BREAST TISSUE:	FCD
T STAGE, PATHOLOGIC:	pT1c
N STAGE, PATHOLOGIC:	pN1mi
M STAGE, PATHOLOGIC:	pMX
ESTROGEN RECEPTORS:	positive
PROGESTERONE RECEPTORS:	positive
HER2/NEU:	zero or 1+

UUID:18E4043D-34BF-4CD1-9E2D-F906C7C7B019
TCGA-BH-A0BT-01A-PR
Redacted

Criteria	Yes	No
Diagnosis Discrepancy		X
Primary Tumor Site Discrepancy		X
HIPAA Discrepancy		X
Prior Malignancy History		X
Dual/Synchronous Primary Noted		
Case is (circle): QUALIFIED / DISQUALIFIED		
Reviewer Initials [illegible]	Date Reviewed: 3/13/11	

[illegible] 3/13/11

[redacted]

[illegible] DIAGNOSIS:

PART 1: LYMPH NODE, RIGHT SENTINEL NUMBER 1, BIOPSY –
NO EVIDENCE OF METASTATIC MAMMARY CARCINOMA IN ONE LYMPH NODE (0/1).

PART 2: LYMPH NODE, RIGHT SENTINEL NUMBER 2, BIOPSY
NO EVIDENCE OF METASTATIC MAMMARY CARCINOMA IN ONE LYMPH NODE (0/1).

PART 3: BREAST, RIGHT, TOTAL MASTECTOMY –
- A. INVASIVE DUCTAL CARCINOMA.
- B. NOTTINGHAM GRADE I (TUBULE FORMATION 2, NUCLEAR PLEOMORPHISM 2, MITOTIC ACTIVITY 1; TOTAL SCORE 5/9).
- C. INVASIVE TUMOR MEASURES 1.2 CM IN MAXIMUM DIMENSION (SLIDE 3F).
- D. DUCTAL CARCINOMA IN SITU (DCIS), NUCLEAR GRADE 1, CRIBRIFORM PATTERN.
- E. DCIS CONSTITUTES 10% OF THE TOTAL TUMOR MASS AND IS PRESENT ADMIXED WITH AND AWAY FROM THE INVASIVE COMPONENT.
- F. LOBULAR CARCINOMA IN SITU (LCIS) IS ALSO IDENTIFIED.
- G. NO LYMPHOVASCULAR SPACE INVASION IS IDENTIFIED.
- H. INVASIVE CARCINOMA EXTENDS FOCALLY TO THE ANTERIOR MARGIN (SLIDE 3E).
- I. NIPPLE IS NEGATIVE FOR TUMOR.
- J. ATYPICAL DUCTAL HYPERPLASIA.
- K. ATYPICAL LOBULAR HYPERPLASIA.
- L. THE NON-NEOPLASTIC BREAST SHOWS FIBROADENOMA FORMATION.
- M. PREVIOUS BIOPSY SITE CHANGES.

PART 4: LYMPH NODE, RIGHT SENTINEL NODE NUMBER 3, BIOPSY –
METASTATIC INFILTRATING DUCT CARCINOMA; 4.0 MM MAXIMUM METASTASIS DIMENSION WITHOUT PERICAPSULAR INVASION, INVOLVING ONE LYMPH NODE (1/1).

PART 5: LYMPH NODE, RIGHT SENTINEL NODE NUMBER 4, BIOPSY –
NO EVIDENCE OF METASTATIC MAMMARY CARCINOMA IN ONE LYMPH NODE (0/1).

PART 6: AXILLARY CONTENTS, RIGHT, DISSECTION –
NO EVIDENCE OF METASTATIC MAMMARY CARCINOMA IN FOURTEEN LYMPH NODES (0/14).

CASE SYNOPSIS:

SYNOPTIC - PRIMARY INVASIVE CARCINOMA OF BREAST

LATERALITY:	Right
PROCEDURE:	Simple mastectomy
LOCATION:	Not specified
SIZE OF TUMOR:	Maximum dimension invasive component: 1.2 cm
MULTICENTRICITY/MULTIFOCALITY OF INVASIVE FOCI:	No
TUMOR TYPE (invasive component):	Ductal adenocarcinoma, NOS
NOTTINGHAM SCORE:	Nuclear grade: 2<br>Tubule formation: 2<br>Mitotic activity score: 1<br>Total Nottingham score: 5<br>Nottingham grade (1, 2, 3): 1
ANGIOLYMPHATIC INVASION:	No
DERMAL LYMPHATIC INVASION:	No
CALCIFICATION:	No
TUMOR TYPE, IN SITU:	Cribriform<br>LCIS
SURGICAL MARGINS INVOLVED BY INVASIVE COMPONENT:	Yes, focal
SURG MARGINS INVOLVED BY IN SITU COMPONENT:	No
PAGET'S DISEASE OF NIPPLE:	No
LYMPH NODES POSITIVE:	1
LYMPH NODES EXAMINED:	18
METHOD(S) OF LYMPH NODE EXAMINATION:	H/E stain
SENTINEL NODE METASTASIS:	No
SIZE OF NODAL METASTASES:	Diameter of largest lymph node metastasis: 4.0 mm
LYMPH NODE METASTASIS(-ES) WITH EXTRACAPSULAR EXTENSION:	No
SKIN INVOLVED (ULCERATION):	No
NON-NEOPLASTIC BREAST TISSUE:	ADH, ALH, Fibroadenoma
T STAGE, PATHOLOGIC:	pT1c
N STAGE, PATHOLOGIC:	pN1
M STAGE, PATHOLOGIC:	pMX
ESTROGEN RECEPTORS:	positive
PROGESTERONE RECEPTORS:	positive
HER2/NEU:	zero or 1+

[redacted]

ICD-O-3
carcinoma, infiltrating ductal, NOS 8500/3
Site: breast, NOS C50.9 3/13/11

UUID:C0AFC806-9F96-484F-BF40-89B26ABF8714
TCGA-BH-A0DT-01A-PR Redacted

Criteria	Yes	No
Diagnosis Discrepancy		✓
Primary Tumor Site Discrepancy		✓
HIPAA Discrepancy		✓
Prior Malignancy History		✓
Dual/Synchronous Primary Noted		✓

Case is (circle): QUALIFIED / DISQUALIFIED
Reviewer Initials [illegible] Date Reviewed: 3/15/11
[illegible] 3/13/11

[redacted]

FINAL DIAGNOSIS:

PART 1: LEFT SENTINEL LYMPH NODE #1, BIOPSY –
ONE (1) LYMPH NODE, NEGATIVE FOR TUMOR (0/1).

PART 2: LEFT SENTINEL LYMPH NODE #2, BIOPSY –
ELEVEN LYMPH NODES, NEGATIVE FOR TUMOR (0/11).

PART 3: LEFT BREAST, SEGMENTAL MASTECTOMY –

A. INFILTRATING DUCT CARCINOMA, 0.8 X 0.7 X 0.6 CM.
B. NOTTINGHAM SCORE: 7/9 (TUBULES – 2, NUCLEI – 2, MITOSES – 3).
C. NO LYMPHOVASCULAR INVASION IDENTIFIED.
D. DUCTAL CARCINOMA IN SITU, CRIBRIFORM TYPE, NUCLEAR GRADE 2, PRESENT IN THE SURROUNDING BREAST TISSUE AND ASSOCIATED WITH MICROCALCIFICATIONS.
E. SURGICAL MARGINS NEGATIVE FOR INFILTRATING DUCT CARCINOMA AND DUCTAL CARCINOMA IN SITU.
F. CHANGES CONSISTENT WITH PREVIOUS CORE BIOPSY SITE.
G. TUMOR IS ESTROGEN RECEPTOR POSITIVE, PROGESTERONE RECEPTOR FOCALLY AND WEAKLY POSITIVE AND HER-2/neu IS NEGATIVE, SCORE: 0.
H. FIBROCYSTIC CHANGES.
I. THERMAL EFFECT.

PART 4: LEFT BREAST, NEW POSTERIOR MARGIN, EXCISION –

A. NEGATIVE FOR TUMOR.
B. FIBROCYSTIC CHANGES.

ICD-0-3
Carcinoma, infiltrating ductal, NOS 8500/3
Site: breast, NOS C50.9 3/13/11 [illegible]

CASE SYNOPSIS:

SYNOPTIC - PRIMARY INVASIVE CARCINOMA OF BREAST

LATERALITY:	Left
PROCEDURE:	Segmental
LOCATION:	Not specified
SIZE OF TUMOR:	Maximum dimension invasive component: 0.8 cm
MULTICENTRICITY/MULTIFOCALITY OF INVASIVE FOCI:	No
TUMOR TYPE (invasive component):	Ductal adenocarcinoma, NOS
NOTTINGHAM SCORE:	Nuclear grade: 2<br>Tubule formation: 2<br>Mitotic activity score: 3<br>Total Nottingham score: 7<br>Nottingham grade (1, 2, 3): 2
ANGIOLYMPHATIC INVASION:	No
DERMAL LYMPHATIC INVASION:	Not applicable
CALCIFICATION:	Yes, malignant zones
TUMOR TYPE, IN SITU:	Cribriform
SURGICAL MARGINS INVOLVED BY INVASIVE COMPONENT:	No
SURG MARGINS INVOLVED BY IN SITU COMPONENT:	No
PAGET'S DISEASE OF NIPPLE:	No
LYMPH NODES POSITIVE:	0
LYMPH NODES EXAMINED:	11
METHOD(S) OF LYMPH NODE EXAMINATION:	H/E stain
SENTINEL NODE METASTASIS:	No
NON-NEOPLASTIC BREAST TISSUE:	FCD
T STAGE, PATHOLOGIC:	pT1b
N STAGE, PATHOLOGIC:	pN0
M STAGE, PATHOLOGIC:	pMX
ESTROGEN RECEPTORS:	positive
PROGESTERONE RECEPTORS:	positive
HER2/NEU:	zero or 1+

Criteria	Yes	No
Diagnosis Discrepancy		X
Primary Tumor Site Discrepancy		X
HIPAA Discrepancy		X
Prior Malignancy History		X
Dual/Synchronous Primary Noted		X
Case is (circle): QUALIFIED / DISQUALIFIED		
Reviewer Initials [illegible]	Date Reviewed: 3/15/11	

[illegible] 3/13/11

UUID:EBAB36DE-A97F-4F8B-806C-EF182519933A
TCGA-BH-A0DX-01A-PR Redacted

[redacted]

FINAL DIAGNOSIS:

PART 1: BREAST, RIGHT, NEEDLE LOCALIZED SEGMENTAL MASTECTOMY –

A. INVASIVE DUCTAL CARCINOMA, 2.0 CM (GROSS), NOTTINGHAM GRADE 1 (COMBINED NOTTINGHAM SCORE 4/9: TUBULE FORMATION 1/3, NUCLEAR PLEOMORPHISM 2/3, MITOTIC ACTIVITY 1/3).
B. DUCTAL CARCINOMA IN SITU (DCIS), NUCLEAR GRADE 2, CRIBRIFORM TYPE, CONSTITUTES APPROXIMATELY 5% OF THE TUMOR VOLUME AND IS ADMIXED WITH THE INVASIVE COMPONENT.
C. LYMPHOVASCULAR SPACE INVASION IS NOT IDENTIFIED.
D. MARGINS FREE, CLOSEST ANTERIOR/SUPERIOR WITHIN 0.6 CM.
E. DUCTAL EPITHELIAL HYPERPLASIA.
F. FIBROCYSTIC CHANGES.
G. SCLEROSING ADENOSIS.
H. COLUMNAR CELL CHANGE.
I. FIBROADENOMATOID NODULES.
J. SKIN IS NEGATIVE FOR TUMOR.
K. BIOPSY SITE CHANGES.

PART 2: LYMPH NODES, RIGHT, SENTINEL #1, [illegible]

A. METASTATIC CARCINOMA (0.4 CM.) INVOLVES ONE OF TWO LYMPH NODES (1/2) (see comment).
B. EXTRACAPSULAR EXTENSION IS IDENTIFIED, 0.1 CM X 0.07 CM.

PART 3: LYMPH NODE, RIGHT, SENTINEL #2, BIOPSY –

A. METASTATIC CARCINOMA INVOLVES ONE LYMPH NODE (1/1) (see comment).
B. EXTRACAPSULAR EXTENSION IS NOT IDENTIFIED.

PART 4: LYMPH NODE, RIGHT, SENTINEL #3, BIOPSY –

ONE LYMPH NODE, NEGATIVE FOR METASTATIC CARCINOMA (0/1) (see comment).

PART 5: BREAST, RIGHT, NEW LATERAL MARGIN, EXCISION –

A. RADIAL SCAR.
B. FIBROCYSTIC CHANGES.
C. SCLEROSING ADENOSIS.
D. DUCTAL EPITHELIAL HYPERPLASIA.
E. COLUMNAR CELL CHANGE WITH ASSOCIATED CALCIFICATIONS.
F. FAT NECROSIS.

[illegible] SYNOPSIS:

SYNOPTIC - PRIMARY INVASIVE CARCINOMA OF BREAST

LATERALITY:	Right
PROCEDURE:	Segmental
LOCATION:	Not specified
SIZE OF TUMOR:	Maximum dimension invasive component: 2 cm
MULTICENTRICITY/MULTIFOCALITY OF INVASIVE FOCI:	Yes
TUMOR AGGREGATE SIZE:	Sum of the sizes of multiple invasive tumors: 2.5 cm
TUMOR TYPE (invasive component):	Ductal adenocarcinoma, NOS
NOTTINGHAM SCORE:	Nuclear grade: 2<br>Tubule formation: 1<br>Mitotic activity score: 1<br>Total Nottingham score: 4<br>Nottingham grade (1, 2, 3): 1
ANGIOLYMPHATIC INVASION:	No
DERMAL LYMPHATIC INVASION:	No
CALCIFICATION:	Yes, benign zones<br>Yes, malignant zones
TUMOR TYPE, IN SITU:	Cribriform<br>DCIS admixed with invasive carcinoma<br>Percent of tumor occupied by in situ component: 5 %
SURGICAL MARGINS INVOLVED BY INVASIVE COMPONENT:	No
SURG MARGINS INVOLVED BY IN SITU COMPONENT:	No
LYMPH NODES POSITIVE:	2
LYMPH NODES EXAMINED:	4
METHOD(S) OF LYMPH NODE EXAMINATION:	H/E stain, Keratin stain
SENTINEL NODE METASTASIS:	Yes
SIZE OF NODAL METASTASES:	Diameter of largest lymph node metastasis: 4 mm
LYMPH NODE METASTASIS(-ES) WITH EXTRACAPSULAR EXTENSION:	Yes
T STAGE, PATHOLOGIC:	pT1c
N STAGE, PATHOLOGIC:	pN1
M STAGE, PATHOLOGIC:	pMX
ESTROGEN RECEPTORS:	positive
PROGESTERONE RECEPTORS:	positive
HER2/NEU:	zero or 1+

ICD-O-3
Carcinoma, infiltrating ductal, NOS 8500/3
Site: breast, NOS C50.9 3/13/11 [illegible]

UUID:DFB21194-30C7-430E-A53C-C38345209B1F
TCGA-BH-A0EI-01A-PR Redacted

Criteria	Yes	No
Diagnosis Discrepancy		X
Primary Tumor Site Discrepancy		X
HIPAA Discrepancy		X
Prior Malignancy History		X
Dual/Synchronous Primary Noted		X
Case is (circle): QUALIFIED / DISQUALIFIED		
Reviewer Initials [illegible] Date Reviewed: 3/13/11		

[illegible] 3/13/11

ICD-O-3
Carcinoma, infiltrating ductal, NOS 8500/3
Site Breast, NOS C50.9 1/21/11

Procedure Date:
Procedure Physician:
Attending Physician/Copies To:

UUID:FF6BC87F-029E-4565-A35A-6FB968F8EABA
TCGA-BH-A1EU-01A-PR Redacted

PATIENT HISTORY:

PATH HAS SPECIMEN nnDATE of LMP: *
DATE OF LAST DELIVERY: *
PRE-OP DIAGNOSIS: R BREAST CA
POST-OP DIAGNOSIS: SAME
OPERATIVE PROCEDURE: R SEGM MAST
CLINICAL HISTORY: PATH HAS SPECIMEN
MATERIAL SUBMITTED: RIGHT BREAST BIOPSY/MASS/SEGMENTAL MASTECTOMY, PROCUREMENT BY SURGICAL PROCEDURE
INTRAOPERATIVE CONSULTATION:
CONSULT: Right breast: 8.0 by 7.0 by 2.5 cm, with 1.7 by 1.5 by 1.5 cm tumor. All margins grossly negative.
[redacted]

ADDENDA:

Addendum

FINAL DIAGNOSIS:
[redacted]
ER/PR
IMMUNOPEROXIDASE IDENTIFICATION OF ESTROGEN AND PROGESTERONE RECEPTORS IS CARRIED OUT ON SLIDE [redacted] "A1". DISTINCT INTRANUCLEAR STAINING IS IDENTIFIED FOR ESTROGEN RECEPTOR (95%) AND PROGESTERONE RECEPTOR (95%). THEREFORE, BOTH ARE INTERPRETED AS POSITIVE.
HER-2/NEU
c-erbB2 (HER-2/NEU) IMMUNOSTAINING IS CARRIED OUT ON MAGEE SURGICAL BLOCK "D2" (BREAST CANCER) USING A 1:300 DILUTION OF DAKO'S POLYCLONAL ANTIBODY A485 (DIRECTED AGAINST THE INTRACELLULAR DOMAIN OF c-erbB2) WITHOUT ANTIGEN RETRIEVAL. NO DISTINCT COMPLETE MEMBRANE STAINING IS IDENTIFIED. THEREFORE, c-erbB2 (HER-2/NEU) IS INTERPRETED AS NEGATIVE (SCORE 0)

FINAL DIAGNOSIS:

FINAL DIAGNOSIS:
RIGHT SEGMENTAL MASTECTOMY:
- INFILTRATING DUCTAL CARCINOMA, INTERMEDIATE HISTOLOGIC AND NUCLEAR GRADE
- NOTTINGHAM SCORE 5
- SIZE OF TUMOR 1.7 CM
- RETROGRADE EXTENSION INTO LOBULES IS SEEN
- LYMPHOCYTIC INFILTRATION SEE
- MICROCALCIFICATIONS
- PROLIFERATIVE FIBROCYSTIC CHANGES AND INTRADUCTAL PAPILLOMA
- SCLEROSING ADENOSIS
- MARGINS ARE FREE

Criteria	Yes	No
Diagnosis Discrepancy		X
Primary Tumor Site Discrepancy		X
HIPAA Discrepancy		X
Prior Malignancy History		X
Dual/Synchronous Primary Noted		X
Case is (circle): QUALIFIED / DISQUALIFIED		
Reviewer Initials [illegible] Date Reviewed: [illegible]		

Collection Date:

ICD-0-3
Carcinoma, infiltrating ductal, NOS 8500/3
Site: Breast, NOS C50.9 1/20/11

Criteria	Yes	No
Diagnosis Discrepancy		X
Primary Tumor Site Discrepancy		X
HIPAA Discrepancy		X
Prior Malignancy History		X
Dual/Synchronous Primary Noted		X
Case is (circle): QUALIFIED / DISQUALIFIED		
Reviewer Initials	Date Reviewed:	

UUID:7E9B0BE7-121E-423F-AA37-445BA922FC13
TCGA-BH-A1F6-01A-PR
Redacted

PATIENT HISTORY:

The patient is a year-old female. Date of last menstrual period: Not given.
PRE OP DIAGNOSIS: Left breast cancer.
POST OP DIAGNOSIS: Same.
PROCEDURE: Left modified radical mastectomy.
[redacted]

FINAL DIAGNOSIS:

PART 1: LEFT BREAST, MODIFIED RADICAL MASTECTOMY –

A. INFILTRATING DUCTAL CARCINOMA OF BREAST, NOTTINGHAM SCORE 8/9 (TUBULES 3, NUCLEI 3, MITOSES 2).
B. NEOPLASM MEASURES 5.5 CM IN MAXIMUM DIMENSION .
C. NEOPLASM EXTENDS INTO DERMIS.
D. MARGINS OF RESECTION FREE OF NEOPLASM.
E. EXTENSIVE DERMAL LYMPHOVASCULAR EXTENSION IDENTIFIED (T4d).
F. NO PAGET'S DISEASE OF NIPPLE IDENTIFIED.
G. METASTATIC CARCINOMA IN INTRAMAMMARY LYMPH NODE, 1 OF 1, 0.7 CM IN DIAMETER WITH EXTRACAPSULAR EXTENSION.

PART 2: LEFT AXILLARY CONTENTS, AXILLARY DISSECTION.
METASTATIC CARCINOMA IN LYMPH NODES, 3 OF 3.

PART 3: LEFT AXILLARY CONTENTS, AXILLARY DISSECTION.
[redacted] METASTATIC CARCINOMA IN LYMPH NODES, 2 OF 2.

[redacted]

COMMENT:

Estrogen Progesterone and Her-2/Neu analyses were performed on the two previous specimens (outside block from [redacted] and [redacted] and interpreted on both specimens as ER negative, PR negative and Her-2/Neu negative. If repeat analyses are desired on current specimen, please contact the laboratory.

SYNOPTIC - PRIMARY INVASIVE CARCINOMA OF BREAST

A. Laterality: 2
   1. Right   2. Left
B. Procedure: 3
   1. Segmentectomy   3. Modified radical mastectomy
   2. Simple mastectomy   4. Re-excision
C. Location: 6
   1. Central subareolar   4. LOQ
   2. UOQ   5. LIQ
   3. UIQ   6. Not specified
D. Size of tumor (maximum dimension invasive component by gross or microscopic exam): 5.5 x 3.5 x 2.5 cm
E. Type (invasive component): 1
   1. Ductal, NOS   5. Cribriform   9. Metaplastic
   2. Tubular   6. Papillary   10. Other
   3. Mucinous   7. Lobular
   4. Medullary   8. Carcinoid like
F. If lobular carcinoma, specify type: N/A
   1. Classical   4. Signet ring   7. Pleomorphic
   2. Solid   5. Trabecular
   3. Alveolar   6. Tubulobular
G. Nottingham Score:
   G1. Nuclear grade: 3
   G2. Tubule formation: 3
   G3. Mitotic activity score: 2
   G4. Total Nottingham score: 8
   G5. Nottingham grade (1, 2, 3): #
H. Angiolymphatic invasion: 2
   1. No   2. Yes
I. Dermal lymphatic invasion: 1
   1. Yes   2. No   3. Not applicable
J. Calcification: 1
   1. No   2. Yes – benign zones   3. Yes – malignant zones
K. Type of in situ component: N/A
   1. Cribriform   4. Micropapillary   7. Lobular
   2. Solid   5. Apocrine
   3. Papillary   6. Comedo
L. Percentage of tumor occupied by in situ component: N/A
M. Surgical margins involved by invasive component: 1
   1. No   2. Yes – focal   3. Yes – diffuse
N. Surgical margins involved by in situ component: N/A
   1. No   2. Yes – focal   3. Yes - diffuse
O. Paget's disease of nipple: 2
   1. Yes   2. No
P. Number of positive lymph nodes: 6
Q. Total number of lymph nodes examined: 6
R. Sentinel node metastasis: N/A
   1. Yes   2. No
S. Only micrometastases to lymph nodes (none larger than 0.2 cm): 2
   1. Yes   2. No
T. Metastasis/es to a lymph node 2 cm. or more in greatest dimension: 2
   1. Yes   2. No
U. Lymph node metastasis/es with extracapsular extension: 1
   1. Yes   2. No
V. Metastases to ipsilateral internal mammary lymph node (if applicable): N/A
   1. Yes   2. No
W. Skin Involved (ulceration): 2
   1. Yes   2. No
X. Non-neoplastic breast tissue: 6
   1. ADH   4. Fibroadenoma   7. LCIS
   2. ALH   5. Papilloma   8. Other
   3. Radical scar   6. FCD
Y. Multicentricity/multifocality of invasive foci: 2
   1. Yes   2. No
Z. TNM stage: T 4d pN 2a M X

PATIENT HISTORY:
CHIEF COMPLAINT/ PRE-OP/ POST-OP DIAGNOSIS: Left breast invasive ductal carcinoma.
LMP DATE: Not applicable
PROCEDURE: Left segmental mastectomy with sentinel lymph node biopsy.
SPECIFIC CLINICAL QUESTION: Not listed.
OUTSIDE TISSUE DIAGNOSIS: Not listed.
PRIOR MALIGNANCY: Not listed.
CHEMORADIATION THERAPY: Not listed
OTHER DISEASES: Not listed.

ICD-O-3
Carcinoma, infiltrating ductal, NOS 8500/3
Site: breast, NOS C50.9 [illegible] 3/28/11

FINAL DIAGNOSIS:

PART 1: LYMPH NODE, NON-SENTINEL, BIOPSY -
ONE (1) LYMPH NODE, NEGATIVE FOR METASTATIC CARCINOMA (0/1).

PART 2: LYMPH NODE, LEFT AXILLA, SENTINEL #1, BIOPSY -
ONE (1) LYMPH NODE, NEGATIVE FOR METASTATIC CARCINOMA (0/1).

PART 3: LYMPH NODE, LEFT AXILLA, SENTINEL #2, BIOPSY -
ONE (1) LYMPH NODE, NEGATIVE FOR METASTATIC CARCINOMA (0/1).

PART 4: BREAST, LEFT, SEGMENTAL MASTECTOMY -
- A. INVASIVE DUCTAL CARCINOMA, NO SPECIAL TYPE.
- B. NOTTINGHAM GRADE III (TUBULE FORMATION: 3, NUCLEAR PLEOMORPHISM: 3, MITOTIC ACTIVITY: 3; TOTAL SCORE: 9/9).
- C. THE INVASIVE TUMOR MEASURES 2.6 CM IN LARGEST DIMENSION.
- D. DUCTAL CARCINOMA IN SITU, NUCLEAR GRADE 3, SOLID TYPE WITH COMEDO NECROSIS.
- E. THE DUCTAL CARCINOMA IN SITU CONSTITUTES 40% OF THE TOTAL TUMOR VOLUME AND IS PRESENT ADMIXED WITH THE INVASIVE COMPONENT.
- F. NO LYMPHOVASCULAR SPACE INVASION IS NOTED.
- G. RESECTION MARGINS ARE NEGATIVE FOR CARCINOMA.
- H. INVASIVE CARCINOMA IS 0.4 CM FROM THE NEAREST (ANTERIOR) MARGIN.
- I. DUCTAL CARCINOMA IN SITU IS 0.3 CM FROM THE NEAREST (ANTERIOR) MARGIN.
- J. ATYPICAL DUCTAL HYPERPLASIA.
- K. THE NON-NEOPLASTIC BREAST SHOWS DUCTAL EPITHELIAL HYPERPLASIA, INTRADUCTAL PAPILLOMA, RADIAL SCAR, COLUMNAR CELL CHANGES, AND FIBROCYSTIC CHANGES.
- L. PREVIOUS BIOPSY SITE CHANGES.

PART 5: LYMPH NODE, LEFT "INTRAMAMMARY", EXCISIONAL BIOPSY -
- A. ONE (1) LYMPH NODE, NEGATIVE FOR METASTATIC CARCINOMA (0/1).
- B. BENIGN PERINODAL ADIPOSE TISSUE.
- C. NO DUCTAL BREAST TISSUE SEEN.

[redacted]

COMMENT:
Part 4: The invasive tumor was reported to be positive for Estrogen Receptors (H-score: 250), positive for Progesterone Receptors (H-score: 75) and also positive for HER2, as per previous pathology report ([redacted]).

CASE SYNOPSIS:

SYNOPTIC - PRIMARY INVASIVE CARCINOMA OF BREAST

LATERALITY:	Left
PROCEDURE:	Segmental
LOCATION:	Clock position.12
SIZE OF TUMOR:	Maximum dimension invasive component: 26 mm
MULTICENTRICITY/MULTIFOCALITY OF INVASIVE FOCI:	No
TUMOR TYPE (invasive component):	Ductal adenocarcinoma, NOS
NOTTINGHAM SCORE:	Nuclear grade: 3<br>Tubule formation: 3<br>Mitotic activity score: 3<br>Total Nottingham score: 9<br>Nottingham grade (1, 2, 3): 3
ANGIOLYMPHATIC INVASION:	No
DERMAL LYMPHATIC INVASION:	Not applicable
CALCIFICATION:	No
TUMOR TYPE, IN SITU:	Solid<br>Comedo
SURGICAL MARGINS INVOLVED BY INVASIVE COMPONENT:	No<br>Distance of invasive tumor to closest margin: 4 mm
SURG MARGINS INVOLVED BY IN SITU COMPONENT:	No<br>Distance of in situ disease to closest margin: 3 mm
LYMPH NODES POSITIVE:	0
LYMPH NODES EXAMINED:	4
METHOD(S) OF LYMPH NODE EXAMINATION:	H/E stain
SENTINEL NODE METASTASIS:	No
NON-NEOPLASTIC BREAST TISSUE:	Radial scar, Papilloma, FCD
T STAGE, PATHOLOGIC:	pT2
N STAGE MODIFIER:	(sn)
N STAGE, PATHOLOGIC:	pN0
M STAGE:	Not applicable
ESTROGEN RECEPTORS:	positive, H-score: 250
PROGESTERONE RECEPTORS:	positive, H-score: 75
HER2/NEU:	3+

UUID:57323AE5-3EFE-4492-8522-D9A6DB3F1BE0
TCGA-BH-A202-01A-PR
Redacted

Criteria	Yes	No
Diagnosis Discrepancy		X
Primary Tumor Site Discrepancy		X
HIPAA Discrepancy		X
Prior Malignancy History		X
Dual/Synchronous Primary Noted		X
Case is (circle): QUALIFIED / DISQUALIFIED		
Reviewer Initials [illegible]	Date Reviewed: 3/28/11	

ICD-O-3
Carcinoma, infiltrating ductal, NOS 8500/3
Site: breast, NOS C50.9 1/28/11

ID#:

Pathology Form

Specimen Information

Collected by: ____________ Date ______ Time: ________

Preserved by: ____________ Date ______ Time: ________

SPECIMEN TYPE (# of samples provided)							
Frozen		Paraffin Block		Blood/Serum/Plasma		Slide	
Diseased	Normal	Diseased	Normal	Diseased	Normal	Diseased	Normal
X	X	X	X			X	X
Time to LN2		Time to Formalin		Time to LN2			
10 min		10 min		60 min			

PATHOLOGICAL DESCRIPTION			
**Primary Tumor**			
**Organ**	**Size**	**Extension of Tumor**	**Distance to NAT**
Breast	2.5 x x cm	NO	2 cm

**Lymph Nodes**		
**Location**	**# Examined**	**# Metastasized**
Axilla	8	5

**Distant Metastasis**		
**Organ**	**Detailed Location**	**Size**
NO		

Pathological Staging

pT 2 N 1 M 0 Stage: II B

Notes:

UUID:F04324BB-F0B1-4948-9189-7833646029F1
TCGA-C8-A12P-01A-PR
Redacted

Criteria	Yes	No
Diagnosis Discrepancy		X
Primary Tumor Site Discrepancy		X
HIPAA Discrepancy		X
Prior Malignancy History		X
Dual/Synchronous Primary Noted		X
Case is (circle): QUALIFIED / DISQUALIFIED		
Reviewer Initials: KM	Date Reviewed: 9/20/10	

ID#:

ILSbio

Microscopic Description

Histological Pattern

Cell Distribution	+	-	Structural Pattern	+	-
Diffuse	+		Streaming		
Mosaic			Storiform		
Necrosis			Fibrosis		
Lymphocytic Infiltration			Palisading		
Vascular Invasion			Cystic Degeneration		
Clusterized	+		Bleeding		
Alveolar Formation			Myxoid Change		
Indian File			Psammoma/Calcification		

Cellular Differentiation

Squamous	+	-	Adenomatous	+	-	Sarcomatous	+	-	Lymphomatous	+	-
Squamoid Cell			Glandular cell	+		Round Cell			Large Cell		
Spindle Cell			Cell Stratification			Fibroblast			Small Cell		
Keratin			Secretion			Osteoblast			RS Cell/RS Like		
Desmosome			Intracyt. Vacuole			Lipoblast			Inflam. Cell		
Pearl			Gland formation	+		Myoblast			Plasma Cell		

Cellular Differentiation: Well X Moderate Poor

Nuclear Appearance

Nuclear Atypia:	0	I	II	III
Aniso Nucleosis				+
Hyperchromatism			+	
Nucleolar Prominent			+	
Multinucleated Giant Cell				
Mitotic Activity			+	
**Nuclear Grade:**			+	

IHC Data

Marker	Result	Value	Date
ER	□ Negative □ Positive		
PR	□ Negative □ Positive		
Her-2/neu	□ Negative □ Positive		
B-Cell Marker	□ Negative □ Positive		
T-Cell Marker	□ Negative □ Positive		
Other:	□ Negative □ Positive		
Other:	□ Negative □ Positive		

Final Pathology Report

Histological Diagnosis: Invasive Ductal Carcinoma

Grade: II

Comments:

Principal Investigator

Pathologist

Date

CONSOLIDATED DIAGNOSTIC PATHOLOGY FORM*

Microscopic Appearance:

1. ***Histological pattern:***

CELL DISTRIBUTION	+	-	STRUCTURAL PATTERN	+	-
Diffuse		X	Streaming		
Mosaic	X		Storiform		
Necrosis		X	Fibrosis		
Lymphocytic Infiltration	X		Palisading		
Vascular Invasion		X	Cystic Degeneration		
Clusterized	X		Bleeding		
Alveolar Formation	X		Myxoid Change		
Indian File	X	X	Psammoma/Calcification		

2. ***Cellular features:***

Squamous	+	-	Adenomatous	+	-	Sarcomatous	+	-	Lymphomatous	+	-
Squamoid Cell			Glandular cell	X		Round Cell			Large Cell		
Spindle Cell			Cell Stratification	X		Fibroblast			Small Cell		
Keratin			Secretion	X		Osteoblast			RS Cell/RS Like		
Desmosome			Intracyt. Vacuole	X		Lipoblast			Inflam. Cell		
Pearl			Gland formation		X	Myoblast			Plasma Cell		
Otherwise Specified: D1 40%, D2 40%											

2. ***Cellular Differentiation:***

Well	Moderately	Poor
		X

3. ***Nuclear Atypia:***

Nuclear Appearance	0	I	II	III
Aniso Nucleosis				X
Hyperchromatism				X
Nucleolar Prominent				X
Multinucleated Giant Cell				X
Mitotic Activity				X
Nuclear Grade				/

Histological Diagnosis: Infiltrating ductal carcinoma NOS

Comments: 6-3

________________ Date ________

[illegible]

ICD-0-3
Carcinoma, infiltrating ductal, NOS 8500/3
Site: breast, NOS C50.9 1/28/11 [illegible]

Pathology Form

Specimen Information

Collected by: [illegible] Date: [illegible] Time:

Preserved by: [illegible] Date [illegible] Time:

SPECIMEN TYPE (# of samples provided)							
Frozen		Paraffin Block		Blood/Serum/Plasma		Slide	
Diseased	Normal	Diseased	Normal	Diseased	Normal	Diseased	Normal
X	X	X	X			X	X
Time to LN2		Time to Formalin		Time to LN2			
10 min		10 min		30 min			

PATHOLOGICAL DESCRIPTION			
**Primary Tumor**			
**Organ**	**Size**	**Extension of Tumor**	**Distance to NAT**
Breast	12 x x cm	NO	2 cm

**Lymph Nodes**		
**Location**	**# Examined**	**# Metastasized**
Axilla	7	5

**Distant Metastasis**		
**Organ**	**Detailed Location**	**Size**
NO		

Pathological Staging

pT1 N2 M0 **Stage:** IIIA

Notes:

UUID:90AF32F0-5684-4D5B-B619-2B12DA3823C6
TCGA-C8-A12Q-01A-PR
Redacted

Criteria	Yes	No
Diagnosis Discrepancy		X
Primary Tumor Site Discrepancy		X
HIPAA Discrepancy		X
Prior Malignancy history		X
Dual/Synchronous Primary Noted		X
Case is (circle): QUALIFIED / DISQUALIFIED		
Reviewer Initials: KMH	Date Reviewed: 9/30/10	

Microscopic Description

Histological Pattern

Cell Distribution	+	-	Structural Pattern	+	-
Diffuse	+		Streaming		
Mosaic			Storiform		
Necrosis	+		Fibrosis		
Lymphocytic Infiltration			Palisading		
Vascular Invasion			Cystic Degeneration		
Clusterized			Bleeding		
Alveolar Formation			Myxoid Change		
Indian File			Psammoma/Calcification		

Cellular Differentiation

Squamous	+	-	Adenomatous	+	-	Sarcomatous	+	-	Lymphomatous	+	-
Squamoid Cell			Glandular cell	+		Round Cell			Large Cell		
Spindle Cell			Cell Stratification			Fibroblast			Small Cell		
Keratin			Secretion			Osteoblast			RS Cell/RS Like		
Desmosome			Intracyt. Vacuole			Lipoblast			Inflam. Cell		
Pearl			Gland formation	+		Myoblast			Plasma Cell		

Cellular Differentiation: Well ✓ Moderate Poor

Nuclear Appearance

Nuclear Atypia:	0	I	II	III
			+	
Aniso Nucleosis			+	
Hyperchromatism			+	
Nucleolar Prominent				
Multinucleated Giant Cell			+	
Mitotic Activity				
**Nuclear Grade:**			+	

IHC Data

Marker	Result	Value	Date
ER	□ Negative □ Positive		
PR	□ Negative □ Positive		
Her-2/neu	□ Negative □ Positive		
B-Cell Marker	□ Negative □ Positive		
T-Cell Marker	□ Negative □ Positive		
Other:	□ Negative □ Positive		
Other:	□ Negative □ Positive		

Final Pathology Report

Histological Diagnosis: Invasive Ductal Carcinoma **Grade:** II

Comments:

Principal Investigator

Pathologist

Date

[illegible]

CONSOLIDATED DIAGNOSTIC PATHOLOGY FORM*

Microscopic Appearance:

1. ***Histological pattern:***

CELL DISTRIBUTION	+	-	STRUCTURAL PATTERN	+	-
Diffuse		✓	Streaming		
Mosaic	✓		Storiform		
Necrosis		✓	Fibrosis		
Lymphocytic Infiltration	✓		Palisading		
Vascular Invasion		✓	Cystic Degeneration		
Clusterized	✓		Bleeding		
Alveolar Formation		✓	Myxoid Change		
Indian File		✓	Psammoma/Calcification		

2. ***Cellular features:***

Squamous	+	-	Adenomatous	+	-	Sarcomatous	+	-	Lymphomatous	+	-
Squamoid Cell			Glandular cell	✓		Round Cell			Large Cell		
Spindle Cell			Cell Stratification	✓		Fibroblast			Small Cell		
Keratin			Secretion	✓		Osteoblast			RS Cell/RS Like		
Desmosome			Intracyt. Vacuole	✓		Lipoblast			Inflam. Cell		
Pearl			Gland formation	✓		Myoblast			Plasma Cell		

Otherwise Specified: D1 50% D2 50%

2. ***Cellular Differentiation:***

Well	Moderately	Poor
	✓	

3. ***Nuclear Atypia:***

Nuclear Appearance	0	I	II	III
Aniso Nucleosis			✓	
Hyperchromatism			✓	
Nucleolar Prominent			✓	
Multinucleated Giant Cell			✓	
Mitotic Activity			✓	
Nuclear Grade				

Histological Diagnosis: Infiltrating Ductal carcinoma, NOS, G2

Comments: M/M: carcinoma metastasized to LN.

Date

*(INTEGRATED REPORT OF FINDINGS BY CONTRIBUTOR AND PATHOLOGIST STAFF FOR RESEARCH USE ONLY).

ICD-O-3
Carcinoma, infiltrating ductal, NOS 8500/3
Site: breast, NOS C50.9 1/28/11

Pathology Form

Specimen Information

Collected by: ______________ Date: ______ Time: ________

Preserved by: ______________ Date: ______ Time: ________

SPECIMEN TYPE (# of samples provided)							
Frozen		Paraffin Block		Blood/Serum/Plasma		Slide	
Diseased	Normal	Diseased	Normal	Diseased	Normal	Diseased	Normal
x	x	x	x			x	x
Time to LN2		Time to Formalin		Time to LN2			
40 min		10 min		14 min			

PATHOLOGICAL DESCRIPTION			
**Primary Tumor**			
**Organ**	**Size**	**Extension of Tumor**	**Distance to NAT**
Breast	2.5 x x cm	NO	2 cm

**Lymph Nodes**		
**Location**	**# Examined**	**# Metastasized**
Axilla	9	2

**Distant Metastasis**		
**Organ**	**Detailed Location**	**Size**
NO		

Pathological Staging

pT2 N1 M0 Stage: II B.

Notes:

UUID:28E368DA-56B9-4BB1-99C3-7FF2CEC964CF
TCGA-C8-A12Y-01A-PR Redacted

Criteria	Yes	No
Diagnosis Discrepancy		X
Primary Tumor Site Discrepancy		X
HIPAA Discrepancy		X
Prior Malignancy History		X
Dual/Synchronous Primary Noted		X
Case is (circle): QUALIFIED // DISQUALIFIED		
Reviewer Initials: [illegible] Date Reviewed: 9/30/10		

Microscopic Description

Histological Pattern

Cell Distribution	+	-	Structural Pattern	+	-
Diffuse	+		Streaming		
Mosaic			Storiform		
Necrosis	+		Fibrosis		
Lymphocytic Infiltration			Palisading		
Vascular Invasion			Cystic Degeneration		
Clusterized	+		Bleeding		
Alveolar Formation			Myxoid Change		
Indian File			Psammoma/Calcification		

Cellular Differentiation

Squamous	+	-	Adenomatous	+	-	Sarcomatous	+	-	Lymphomatous	+	-
Squamoid Cell			Glandular cell	+		Round Cell			Large Cell		
Spindle Cell			Cell Stratification			Fibroblast			Small Cell		
Keratin			Secretion			Osteoblast			RS Cell/RS Like		
Desmosome			Intracyt. Vacuole			Lipoblast			Inflam. Cell		
Pearl			Gland formation	+		Myoblast			Plasma Cell		

Cellular Differentiation: x Well Moderate Poor

Nuclear Appearance

Nuclear Atypia:	0	I	II	III
			+	
Aniso Nucleosis				
Hyperchromatism		+		
Nucleolar Prominent		+		
Multinucleated Giant Cell				
Mitotic Activity		+		
**Nuclear Grade:**		+		

IHC Data

Marker	Result	Value	Date
ER	☐ Negative ☐ Positive		
PR	☐ Negative ☐ Positive		
Her-2/neu	☐ Negative ☐ Positive		
B-Cell Marker	☐ Negative ☐ Positive		
T-Cell Marker	☐ Negative ☐ Positive		
Other:	☐ Negative ☐ Positive		
Other:	☐ Negative ☐ Positive		

Final Pathology Report

Histological Diagnosis: Invasive Ductal Carcinoma **Grade:** I

Comments:

Principal Investigator ______ Pathologist ______ Date ______

CONSOLIDATED DIAGNOSTIC PATHOLOGY FORM*

Microscopic Appearance:

1. Histological pattern:

CELL DISTRIBUTION	+	-	STRUCTURAL PATTERN	+	-
Diffuse		X	Streaming		
Mosaic	X		Storiform		
Necrosis		X	Fibrosis		
Lymphocytic Infiltration	X		Palisading		
Vascular Invasion	X		Cystic Degeneration		
Clusterized	X		Bleeding		
Alveolar Formation	X		Myxoid Change		
Indian File		X	Psammoma/Calcification		

2. Cellular features:

Squamous	+	-	Adenomatous	+	-	Sarcomatous	+	-	Lymphomatous	+	-
Squamoid Cell			Glandular cell	X		Round Cell			Large Cell		
Spindle Cell			Cell Stratification	X		Fibroblast			Small Cell		
Keratin			Secretion	X		Osteoblast			RS Cell/RS Like		
Desmosome			Intracyt. Vacuole	X		Lipoblast			Inflam. Cell		
Pearl			Gland formation	X		Myoblast			Plasma Cell		

Otherwise Specified: [illegible] 60% Pr 60%

2. Cellular Differentiation:

Well	Moderately	Poor
	X	

3. Nuclear Atypia:

Nuclear Appearance	0	I	II	III
Aniso Nucleosis			X	
Hyperchromatism		V		
Nucleolar Prominent			X	
Multinucleated Giant Cell		X		
Mitotic Activity			X	
Nuclear Grade			X	

Histological Diagnosis: Infiltrating ductal carcinoma, NOS, G2

Comments: ______________________________

Date

(INTEGRATED REPORT OF FINDINGS BY CONTRIBUTOR AND PATHOLOGIST STAFF FOR RESEARCH USE ONLY).

ICD-0-3
Carcinoma, infiltrating ductal, NOS 8500/3
Site: breast, NOS C50.9 1/28/11 [illegible]

ID#:

Pathology Form

Specimen Information

Collected by: ____ ________________ Date: ________ Time

Preserved by: ________________ Date ________ Time: ____

SPECIMEN TYPE (# of samples provided)							
Frozen		Paraffin Block		Blood/Serum/Plasma		Slide	
Diseased	Normal	Diseased	Normal	Diseased	Normal	Diseased	Normal
4	2	4	2			4	2
Time to LN2		Time to Formalin		Time to LN2			
11 min		12 min		min			

PATHOLOGICAL DESCRIPTION			
**Primary Tumor**			
**Organ**	**Size**	**Extension of Tumor**	**Distance to NAT**
BREAST TUMOR	2.5 x 2 x 1.5 cm		6 cm

**Lymph Nodes**		
**Location**	**# Examined**	**# Metastasized**

**Distant Metastasis**		
**Organ**	**Detailed Location**	**Size**

Pathological Staging

pT 2 **N** 1 **M** 0 **Stage:** II

Notes: BREAST nodes 4 (3 Negative, 1 positive)

UUID:175224E6-3A06-4799-B695-704C254A847C
TCGA-C8-A132-01A-PR
Redacted

Criteria	Yes	No
Diagnosis Discrepancy		X
Primary Tumor Site Discrepancy		X
HIPAA Discrepancy		X
Prior Malignancy History		X
Dual/Synchronous Primary Noted		
Case is (circle): QUALIFIED / DISQUALIFIED		
Reviewer Initials [illegible] Date Reviewed 9/30/10		

ID#:

Microscopic Description

Histological Pattern					
**Cell Distribution**	**+**	**-**	**Structural Pattern**	**+**	**-**
Diffuse	X		Streaming		
Mosaic			Storiform		
Necrosis			Fibrosis		
Lymphocytic Infiltration	X		Palisading		
Vascular Invasion			Cystic Degeneration		
Clusterized			Bleeding		
Alveolar Formation			Myxoid Change		
Indian File			Psammoma/Calcification		

Cellular Differentiation											
**Squamous**	**+**	**-**	**Adenomatous**	**+**	**-**	**Sarcomatous**	**+**	**-**	**Lymphomatous**	**+**	**-**
Squamoid Cell			Glandular cell	X		Round Cell			Large Cell		
Spindle Cell			Cell Stratification			Fibroblast			Small Cell		
Keratin			Secretion			Osteoblast			RS Cell/RS Like		
Desmosome			Intracyt. Vacuole			Lipoblast			Inflam. Cell		
Pearl			Gland formation	X		Myoblast			Plasma Cell		

Cellular Differentiation: Well Moderate X Poor

Nuclear Appearance				
**Nuclear Atypia:**	**0**	**I**	**II**	**III**
Aniso Nucleosis		X		
Hyperchromatism			X	
Nucleolar Prominent			X	
Multinucleated Giant Cell			X	
Mitotic Activity			X	
**Nuclear Grade:**				

IHC Data			
**Marker**	**Result**	**Value**	**Date**
ER	☐ Negative ☐ Positive		
PR	☐ Negative ☐ Positive		
Her-2/neu	☐ Negative ☐ Positive		
B-Cell Marker	☐ Negative ☐ Positive		
T-Cell Marker	☐ Negative ☐ Positive		
Other:	☐ Negative ☐ Positive		
Other:	☐ Negative ☐ Positive		

Final Pathology Report

Histological Diagnosis: Infiltrating ductal carcinoma **Grade:** III

Comments:

Principal Investigator

Pathologist

[illegible]
Date

P

CONSOLIDATED DIAGNOSTIC PATHOLOGY FORM*

Microscopic Appearance:

1. ***Histological pattern:***

CELL DISTRIBUTION	+	-	STRUCTURAL PATTERN	+	-
Diffuse		X	Streaming		
Mosaic	X		Storiform		
Necrosis	X		Fibrosis		
Lymphocytic Infiltration	X		Palisading		
Vascular Invasion	X		Cystic Degeneration		
Clusterized		X	Bleeding		
Alveolar Formation	X		Myxoid Change		
Indian File		X	Psammoma/Calcification		

2. ***Cellular features:***

Squamous	+	-	Adenomatous	+	-	Sarcomatous	+	-	Lymphomatous	+	-
Squamoid Cell			Glandular cell	X		Round Cell			Large Cell		
Spindle Cell			Cell Stratification	X		Fibroblast			Small Cell		
Keratin			Secretion	X		Osteoblast			RS Cell/RS Like		
Desmosome			Intracyt. Vacuole	X		Lipoblast			Inflam. Cell		
Pearl			Gland formation		X	Myoblast			Plasma Cell		

Otherwise Specified: Cancer, D1 35%, D2 20%, D3 70%, D4 35%. Necrosis <5%

2. ***Cellular Differentiation:***

Well	Moderately	Poor
		X

3. ***Nuclear Atypia:***

Nuclear Appearance	0	I	II	III
Aniso Nucleosis				X
Hyperchromatism				X
Nucleolar Prominent				X
Multinucleated Giant Cell				+
Mitotic Activity				+
Nuclear Grade				X

Histological Diagnosis: Infiltrating Ductal Carcinoma, NOS, G3

Comments: M2: Chronic Lymphadenitis: Detected.
M1: Carcinoma Metastasized to LN

Date

(INTEGRATED REPORT OF FINDINGS BY CONTRIBUTOR AND PATHOLOGIST STAFF FOR RESEARCH USE ONLY).

ICD-O-3
Carcinoma, infiltrating ductal, NOS 8500/3
Site: breast, NOS C50.9 3/11/11

Department of Cancer Pathology

copy No.
Date:

Examination: Histopathological examination (cito)

Examination No.:

Patient: [redacted] PESEL [redacted] Age Gender: **F**

Material: **Multiple organ resection – right breast**
Unit in charge

Physician in charge:
Material collected on: Material received on:
Expected time of examination: 5 working days
Clinical diagnosis:

Examination performed on

Macroscopic description:
Right breast, sized 21.3 x 18.6 x 4.8 cm, removed along with axillary tissues sized 10 x 7 x 3.5 cm and a skin flap of 19.2 x 7.1 cm. Weight 923 g.
Tumour sized 3.3 x 1.8 x 3.2 cm on the border of outer quadrants, placed 2.2 cm from the lower edge, 0.3 cm from the base and 2.8 cm from the skin. Lymph nodes of 0.4 cm in length, fat.

Microscopic description:
Carcinoma ductale invasivum - NHG2 (3 + 3 +2:14 mitoses/10 HPF, visual area diameter 0.55 mm). Mamilla sine laesionibus.
Glandular tissue showing mastopathia fibrosa et cystica. (fibrocystic changes)
AXILLARY LYMPH NODES
Metastases carcinomatosae in lymphonodis (No III/XVI).

Examination result:/**Histopathology diagnosis:**
Carcinoma ductale invasivum mammae dextrae. Invasive ductal carcinoma of the right breast
Metastases carcinomatosae in lymphonodis axillae (NO III/XVI). Cancer metastases in axillary lymph nodes (No III/XVI) (NHG2, pT2, pN1a).

Compliance validated by:

Examination performed on

Results of immunohistochemical examination:
Estrogen receptors found in 75% of neoplastic cell nuclei. Progesterone receptors found in 10-75% of neoplastic cell nuclei. HER2 protein stained with HercepTest™ by DAKO. Negative reaction in invasive cancerous cells (Score = 0)

Compliance validated by:

UUID:E8E799F8-158D-4AEC-8F86-D40F132C071C
TCGA-D8-A1J8-01A-PR Redacted

Criteria	Yes	No
Diagnosis Discrepancy		X
Primary Tumor Site Discrepancy		X
HIPAA Discrepancy		X
Prior Malignancy History		X
Dual/Synchronous Primary Noted		X
Case is (circle): QUALIFIED / DISQUALIFIED		
Reviewer Initials	Date Reviewed: 3/11/11	

ICD-O-3
Carcinoma, infiltrating ductal, NOS 8500/3
Site: breast, NOS C50.9 3/11/11

Department of Cancer Pathology

copy No.

Date:

Examination: Histopathological examination

Internal invoice No. Cost of diagnostic procedure **Examination No.:**

Patient: **XXX** PESEL: **XXX** Age: Gender: **F**

Material: **1. Multiple organ resection – left breast and axillary tissue**

Unit in charge:

Physician in charge:

Material collected on: Material collected on:

Expected time of examination: **up to 8 working days**

Clinical diagnosis: **Status after intraoperative examination – ca invasivum.**

Examination performed on:

Macroscopic description:

Left breast sized 21 x 10 x 5 cm removed along with axillary tissues sized 12 x 7 x 15 cm and a 19 x 10 cm skin flap. Weight 820 g. Tumour site sized 11 x 7 x 3 cm on the boundary of outer quadrants, 2 cm from the upper boundary, 0.5 cm from the base and 1 cm from the skin.

A post-operative scar visible in the outer quadrant skin with a seam of 7 cm.

Metastatic lymph nodes of 2.5 cm.

Microscopic description:

Invasio carcinomatosa vasorum.

Infiltratio carcinomatosa mamillae.

In the tumour site, singular foci of carcinoma ductale invasivum and lesions of the type mastopathia fibrosa et cystica, hyperplasia ductalis simplex (UDH)

AXILLARY LYMPH NODES

Metastases carcinomatosae in lymphonodis No IV/XI.

Infiltratio capsulae lymphonodorum.

Histpathological diagnosis:

Carcinoma ductale partim micropapillare invasivum mammae sinistrae. Invasive ductal carcinoma partially micropapillary of the left breast.

Metastases carcinomatosae in lymphonodis axillae (No IV/XI) (NHG2, pT2, pN2a). Cancer metastases in axillary lymph nodes (No IV/XI)

Invasio carcinoma vasorum. Vascular invasion.

Compliance validated by:

UUID:B4DB830F-BFA4-47F5-94C7-D95984315866
TCGA-D8-A1JC-01A-PR Redacted

Criteria	Yes	No
Diagnosis Discrepancy		X
Primary Tumor Site Discrepancy		X
HIPAA Discrepancy		X
Prior Malignancy History		X
Dual/Synchronous Primary Noted		
Case is (circle): QUALIFIED / DISQUALIFIED		
Reviewer Initials:	Date Reviewed: 3/11/11	

ICD-0-3
Carcinoma, infiltrating ductal, NOS 8500/3
Site: breast NOS C50.9 3/11/11 [illegible]

Department of Cancer Pathology

copy No.

Date:

Examination: **Histopathological examination**

Internal invoice No. Cost of diagnostic procedure **xamination No.:**

Patient: **XXX** PESEL: **XXX** Age: Gender: **F**

Material: **1. Total organ resection – left breast and axillary tissue**

Unit in charge:

Physician in charge:

Material collected on: Material received on:

Expected time of examination: **up to 8 working days**

Clinical diagnosis: **Cancer of the left breast - outer upper quad.**

Examination performed on

Results of immunohistochemical examination:
***Estrogen* receptors found in over 75% of neoplastic cell nuclei.**
Progesterone receptors found in 10-75% of neoplastic cell nuclei.
HER2 protein stained with HercepTest™ by DAKO.
Negative reaction in invasive cancerous cells (Score = 1+)

Compliance validated by:

Examination performed on:

Macroscopic description:
Left breast sized 19 x 18 x 5 cm removed along with axillary tissues sized 11 x 4.5 x 1.5 cm and a 19 x 10 cm skin flap. Weight 620 g.
Tumour sized 2.8 x 1.5 x 2.5 cm on the border of upper quadrants, 4.5 cm from the upper boundary, 0.2 cm from the base and 1.0 cm from the skin.
Another tumour sized 0.5 x 0.7 x 0.5 cm found 1.5 cm away from the first one (margins: lower boundary – 10 cm; base – 0.1 cm; skin – 3.5 cm).

UUID:332880ED-FE4E-4EE4-9E96-0A61BFCCA27E
TCGA-D8-A1JD-01A-PR Redacted

Criteria	Yes	No
Diagnosis Discrepancy		X
Primary Tumor Site Discrepancy		X
HIPAA Discrepancy		
Prior Malignancy History		X
Dual/Synchronous Primary Noted		X
Case is (circle): QUALIFIED / DISQUALIFIED		
Reviewer Initials [illegible]	Date Reviewed: 3/11/11	

Microscopic description:
Both tumours showing similar pattern.
Carcinoma ductale invasivum bifocal - NHG2 (2 + 3 +1/7 mitoses/10 HPF, visual area diameter 0.55 mm).
Foci of carcinoma ductale in situ DCIS found within the tumour (solid and cribrate type with high nuclear atypia and comedo necrosis, 5% of the tumour).
Reactio lymphocytaria peritumoralis.
Mamilla sine laesionibus.
Glandular tissue showing mastopathia fibrosa et cystica.
AXILLARY LYMPH NODES:
Metastases carcinomatosae in lymphonodis No III/XIV.
Infiltratio capsulae lymphonodis et telae perinodalis.

Examination No.:

Patient: **XXX** PESEL: **XXX** Gender: **F**

Examination performed on

Histopathological diagnosis:
Carcinoma ductale invasivum bifocale mammae sinistrae. Invasive bifocal ductal carcinoma of the left breast. Metastases carcinomatosae in lymphonodis axillae No. III/XIV. Cancer metastases in axillary lymph nodes No. III/XIV.

(NHG2, pT2, pN1a).

Compliance validated by:

ICD-0-3
Carcinoma, infiltrating ductal, NOS 8500
Site: breast, NOS C50.9 3/1/11

Department of Cancer Pathology

copy No.

Date:

Examination: **Histopathological examination**

Internal invoice No. Cost of diagnostic procedure **Examination No.:**

Patient: **XXX** PESEL: **XXX** Age: Gender: **F**

Material: **Multiple organ resection – the left breast and axillary tissue**

Unit in charge:

Physician in charge:

UUID:746C603E-FB1F-40D7-BEAF-20ACE27D6C21
TCGA-D8-A1JE-01A-PR
Redacted

Material collected on: /aterial received on:

Expected time of examination: **up to 8 working days**

Clinical diagnosis: **Cancer of the left breast.**

Examination performed on

Results of immunohistochemical examination:
Estrogen receptors found in over 75% of neoplastic cell nuclei.
Progesterone receptors found in over 75% of neoplastic cell nuclei.
HER2 protein stained with HercepTest™ by DAKO.
Negative reaction in invasive cancerous cells (Score = 1+)

Compliance validated by:

Examination performed on:

Macroscopic description:
Left breast sized 19 x 20 x 5 cm removed along with axillary tissues sized 12 x 6 x 2 cm and a 16 x 11 cm skin flap. Weight 800 g. Tumour sized 1.5 x 1.5 x 1.5 cm on the border of upper quadrants, 6.5 cm from the upper boundary, 0.7 cm from the base and 0.1 cm from the skin.
Microscopic description:
Carcinoma ductale invasivum - NHG2 (2 + 2 +3/15 mitoses/10 HPF, visual area diameter 0.55 mm).
Glandular, outside the tumour, tissue showing lesions of the type mastopathia fibrosa et cystica, hyperplasia ductalis simplex (UDH).
AXILLARY LYMPH NODES
Metastases carcinomatosae in lymphonodis No III/X.
Infiltratio capsulae lymphonodorum.

Criteria	Yes	No
Diagnosis Discrepancy		X
Primary Tumor Site Discrepancy		X
HIPAA Discrepancy		X
Prior Malignancy History		X
Dual/Synchronous Primary Noted		X
Case is (circle): QUALIFIED / DISQUALIFIED		
Reviewer Initials	Date Reviewed: / /	

3/1/11

Histopathological diagnosis:

Carcinoma ductale invasivum mammae sinistrae. Invasive ductal carcinoma of the left breast. Metastases carcinomatosae in lymphonodis axillae No. III/X. Cancer metastases in axillary lymph nodes No III/X.

(NHG2, pT1c, pN1a).

Compliance validated by:

ICD-O-3
Carcinoma, infiltrating, ductal, NOS 8500/3
Site: breast, NOS C50.9 3/1/11

Department of Cancer Pathology

copy No.

Date:

Examination: Histopathological examination

Internal invoice No. Cost of diagnostic procedure Examination No.:

Patient: XXX PESEL: XXX Age: Gender: **F**

Material: **Multiple organ resection – right breast and axillary tissue**

UUID:49C79E09-2F69-4089-BFB1-53197DA09A46
TCGA-D8-A1JH-01A-PR Redacted

Unit in charge:

Physician in charge:

Material collected on: Material received on:

Expected time of examination: **up to 8 working days**

Clinical diagnosis: **Cancer of the right breast.**

Examination performed on:

Results of immunohistochemical examination:

Estrogen receptors found in over 75% of neoplastic cell nuclei.
Progesterone receptors found in over 75% of neoplastic cell nuclei.
HER2 protein stained with HercepTest™ by DAKO.
Negative reaction in invasive cancerous cells (Score = 1+)

Compliance validated by:

Examination performed on

Macroscopic description:

Right breast sized 14 x 14.5 x 2.5 cm removed along with axillary tissues sized 11 x 4.5 x 2 cm and a 11 x 5.6 cm skin flap. Weight 260 g.

Tumour sized 1,2 x 10 x 1,0 cm found in the outer lower quadrant, 0.9 cm from the lower boundary, 0.5 cm from the base and less than 0.1 cm from the parenchyma.

Lymph nodes 1.8 cm in length.

Microscopic description:

Carcinoma ductale invasivum – NHG1 (2 + 2 +1: 5 mitoses/ 10 HPF, visual area diameter: 0.55 m).

Calcifications within the tumour.

Mamilla sine laesionibus.

Glandular tissue showing lesions of the type mastopathia fibrosa (fibrocystic changes).

Invasive lesions reaching the base.

AXILLARY LYMPH NODES

Lymphonodulitis chronica et sinis histiocytosis No IX.

Histopathology diagnosis:

Carcinoma ductale invasivum mammae dextrae. (NHG1, pT1c, pN0). Invasive ductal carcinoma of the right breast

Criteria	Yes	No
Diagnosis Discrepancy		X
Primary Tumor Site Discrepancy		X
HIPAA Discrepancy		X
Prior Malignancy History		X
Dual/Synchronous Primary Noted		X
Case is (circle): QUALIFIED / DISQUALIFIED		
Reviewer Initials: [illegible]	Date Reviewed: 3/1/11	

Compliance validated by:

ICD-0-3
Carcinoma, infiltrating ductal, NOS 8500/3
Site: breast, NOS C50.9 3/11/11

Department of Cancer Pathology

copy No.

Date:

Examination: **Histopathological examination**

Internal invoice No. Cost of diagnostic procedure **Examination No.:**

Patient: XXX PESEL: XXX Age: Gender: F

Material: **Multiple organ resection – left breast**

Unit in charge:

Physician in charge:

Material collected or Material received on:

Expected time of examination: **8 working days**

Clinical diagnosis:

Examination performed on:

Results of immunohistochemical examination:

No estrogen receptors found in the neoplastic cell nuclei. Progesterone receptors found in less than 10% of the neoplastic cell nuclei. HER2 protein stained with HercepTest™ by DAKO. Negative reaction in invasive cancerous cells (Score = 1+). Estrogen receptor was stained twice.

Compliance validated by:

Examination performed on:

Macroscopic description:

Right breast sized 15 x 9 x 3 cm removed along with axillary tissues sized 7 x 4 x 1 cm and a skin flap of 11 x 7 cm. Weight 650 g. Tumour sized 3.2 x 2.0 x 3.0 cm on the border of the upper quadrants, placed 2.0 cm from the upper edge and 1.5 cm from the base.

Microscopic description:

Carcinoma ductale invasivum NHG3 (3 + 3 + 3: 21 mitoses/10 HPF - visual area of 0.55 mm). Glandular tissue off the tumour showing lesions of the type mastopathia fibrosa et cystica, hyperplasia ductalis simplex (UDH).

Axillary lymph nodes:

Sinus histiocytosis cum microcalcificationes lymphonodorum (No XIII).

Histopathological diagnosis:

Carcinoma ductale invasivum mammae sinistrae. Invasive ductal carcinoma of the left breast. (NHG3, pT2, pN0).

Compliance validated by

CONTACT YOUR DOCTOR WITH THIS REPORT!

UUID:1841FEE0-EC36-4EC4-8B5E-6D340C80735B
TCGA-D8-A1JK-01A-PR Redacted

Criteria	Yes	No
Diagnosis Discrepancy		X
Primary Tumor Site Discrepancy		X
HIPAA Discrepancy		X
Prior Malignancy History		X
Dual/Synchronous Primary Noted		X
Case is (circle): QUALIFIED / DISQUALIFIED		
Reviewer Initials	Date Reviewed: 3/11/11	

ICD-0-3
carcinoma, infiltrating duct, NOS 8500/3
site: breast, NOS C50.9 4/12/11

Department of Cancer Pathology

copy No.

Date:

Examination: Histopathological examination

Examination No.:

Patient: **XXX** PESEL: **XXX** Age: Gender: **F**

Material: **1. Multiple organ resection – left breast with axillary tissues**

Unit in charge:

Physician in charge:

Material collected on: Material received on:

Expected time of examination: **upto 8 working days**

Clinical diagnosis: **Cancer of the left breast.**

Criteria	Yes	No
Diagnosis Discrepancy		X
Primary Tumor Site Discrepancy		X
HIPAA Discrepancy		X
Prior Malignancy History		X
Dual/Synchronous Primary Noted		X
Case is (circle): QUALIFIED / DISQUALIFIED		
Reviewer Initials	Date Reviewed: 4/12/11	

Examination performed on:

Macroscopic description:

Left breast sized 21 x 18 x 3 cm removed along with axillary tissues sized 10 x 5 x 2 cm and a 15 x 6 cm skin flap. Weight 520 g. Tumour sized 1.9 x 1.6 x 1.7 cm in the middle part, 1.5 cm from the upper boundary, 1.6 cm from the base and 0 cm from the skin.

Microscopic description:

Carcinoma ductale invasivum – NHG2 (2+3+2/10 mitoses/10 HPF - visual area: 0.55mm).

Foci of carcinoma ductale in situ (DCIS) found within the tumour (solid and cribrate type, with medium nuclear atypia, without necrosis and with calcifications of below 5% of the tumour).

Infiltratio carcinomatosa mamillae.

Focus of carcinoma ductale invasivum found in the glandular tissue (inner lower quadrant – diameter 0.1 cm).

Also showing also lesions of the type mastopathia fibrosa et cystica, hyperplasia ductalis simplex (UDH).

AXILLARY LYMPH NODES:

Metastases carcinomatosae in lymphonodis (No VI/XVIII).

Infiltratio capsulae lymphonodi.

Preliminary result:

Final response to be given after whole of the material is analysed.

Compliance validated by:

Examination performed on:.

Results of immunohistochemical examination:

Estrogen receptors found in over 75% of neoplastic cell nuclei. Progesterone receptors found in over 75% of neoplastic cell nuclei. HER2 protein stained with HercepTest™ by DAKO.

Negative reaction in invasive cancerous cells (Score = 1+)

Compliance validated by:

Examination performed on:

UUID:D5614445-4EBF-4050-8FC6-D34D5CAADD82
TCGA-D8-A1XD-01A-PR Redacted

Examination: Histopathological examination

Examination No.:

Patient: XXX PESEL: XXX Gender: F

Examination performed on:

Histopathological diagnosis:

Carcinoma ductale invasivum bifocal et ductale in situ mammae sinistrae.

Invasive ductal bifocal carcinoma and ductal in situ carcinoma of the left breast.

Metastases carcinomatosae in lymphonodis axillae (No VI/XVIII) (NHG2, pT1c, pN2a).

Cancer Metastases in axillary lymph nodes.

Compliance validated by:

CONTACT YOUR DOCTOR WITH THIS REPORT!

Department of Cancer Pathology

conv No.

Date:

Examination: Histopathological examination

Internal invoice No. Cost of diagnostic procedure Examination No.:

Patient: **XXX** PESEL: **XXX** Age Gender: **F**

Material: **1. lesion excision – from th[redacted] outer upper quadrant**
Unit in charge: **Central C**
Physician in charge:
Material collected on: Material received on
Expected time of examination: **5 working days**
Clinical diagnosis: **and left axillary nodes in one block**

ICD-0-3
Carcinoma, infiltrating duct, NOS 8500/3
Site: breast, NOS C50.9 [illegible] 4/12/11

Examination performed on:

Results of immunohistochemical examination:
Estrogen receptors found in over 75% of neoplastic cell nuclei. Progesterone receptors found in over 75% of neoplastic cell nuclei. HER2 protein stained with HercepTest™ by DAKO. Score = 2+, FISH verification recommended

Compliance validated by:

Examination performed on:

Macroscopic description:
Partial resection of breast – outer upper quadrant.
Breast segment sized 8.4 x 7.4 x 2.6 cm with a 2.8 x 6.8 cm skin flap and axillary tissue sized 8 x 9 x 2, no RTG.
Tumour cross section sized 3.0 x 1.8 x 2.9 cm.
Margin: to the base 0.1 cm, to the front skin surface 0.2 cm, to the sternum 3.5 cm, to the axilla 2.2 cm, to the shoulder 1.3 cm, lower 0.8 cm.

Microscopic description:
Carcinoma ductale invasivum NHG3 (3 + 2 + 3/19 mitoses/10 HPF – visual area of 0.55 mm).
Largest dimension of the lesion 3.0 cm.
Minimum margin at the base side < 0.05 cm. The other margins as in macroscopic description.
Lymph nodes: Metastases carcinomatosae in lymphonodis (No II / XIII).
Infiltratio carcinomatosa capsulae lymphonodorum et telae adiposae perinodalis.

Histopathological diagnosis:
Carcinoma ductale invasivum NHG3, pT2, pN1a. Invasive ductal carcinoma of the left breast.

Compliance validated b[illegible]

Examination performed on.

UUID:762963FB-3D8C-4616-8C8C-0CD3E45FD243
TCGA-D8-A1XL-01A-PR Redacted

Criteria	Yes	No
Diagnosis Discrepancy		X
Primary Tumor Site Discrepancy		X
HIPAA Discrepancy		X
Prior Malignancy History		X
Dual/Synchronous Primary Noted		X
Case is (circle): QUALIFIED / DISQUALIFIED		
Reviewer Initials [illegible]	Date Reviewed: 4/13/11	

Examination: Intraoperative examination

Examination No.:

Patient: **XXX** PESEL: **XXX** Gender: **F**

Examination performed on:

Results of immunohistochemical examination:

RESULT OF HER2/neu GENE AMPLIFICATION with the FISH method by Path Vysion HER2 DNA Probe Kit

FINAL RESULT:

HER-2 GENE AMPLIFICATION NOT FOUND

Compliance validated by:

CONTACT YOUR DOCTOR WITH THIS REPORT!

Department of Cancer Pathology

copy No.

Date:

Examination: Histopathological examination

Internal invoice No. Cost of diagnostic procedure Examination No.:

ICD-O-3
carcinoma, infiltrating duct, NOS 8500/3
Site: breast, NOS C50.9 [illegible] 4/12/11

Patient: **XXX** PESEL: **XXX** Age: Gender: **F**

Material: **Multiple organ resection – right breast with axillary tissues**

Unit in charge:

Physician in charge:

Material collected on: Material received on:

Expected time of examination: **up to 8 working days**

Clinical diagnosis: **Cancer of the right breast.** Specimen from a modified radical mastectomy using technique.

Examination performed on:

Macroscopic description:

Right breast sized 20 x 16 x 5 cm removed along with axillary tissues sized 9 x 11 x 3 cm and a skin flap of 20 x 8 cm. Weight 880 g.

Tumour sized 2.2 x 2.0 x 1.5 cm on the border of the outer quadrants, located 3.0 cm from the lower boundary, 1.2 cm from the base and 1.6 cm from the skin. Lymph nodes of 1.0 cm in length.

Microscopic description:

Carcinoma ductale invasivum, basal-like NHG3 (3 + 3 + 2/15 mitoses/10 HPF - visual area 0.55 mm).

Reactio lymphocytaria peritumoralis.

Mamilla sine laesionibus.

Axillary lymph nodes:

Lymphonodulitis reactiva No VII.

UUID:33B6269D-1D59-4184-A786-2BEB73F6D3A8
TCGA-D8-A1XQ-01A-PR Redacted

Histopathological diagnosis:

Invasive ductal carcinoma of the right breast (NHG3, pT2, pNO).

Compliance validated by:

Examination performed on:

Results of immunohistochemical examination:

No estrogen receptors found in neoplastic cell nuclei. Progesterone receptors found in less than 10% of neoplastic cell nuclei. HER2 protein stained with HercepTest™ by DAKO. Negative reaction in invasive cancerous cells (Score = 1+). Tests performed twice.

Compliance val

CONTACT YOUR DOCTOR WITH THIS REPORT!

Criteria	Yes	No
Diagnosis Discrepancy		X
Primary Tumor Site Discrepancy		X
HIPAA Discrepancy		X
Prior Malignancy History		X
Dual/Synchronous Primary Noted		X
Case is (circle): QUALIFIED / DISQUALIFIED		
Reviewer Initials [illegible] Date Reviewed: 4/12/11		

Department of Cancer Pathology

Date:

Examination: Histopathological examination

Internal invoice No. Cost of diagnostic procedure Examination No.:

Patient: **XXX** PESEL: **XXX** Age: Gender: **F**

ICD-0-3
carcinoma, infiltrating duct, nos 8500/3
Site: breast, nos C50.9
4/12/11

Material: **Multiple organ resection – right breast with axillary tissues**
Unit in charge:
Physician in charge:
Material collected on: Material received on:
Expected time of examination: **up to 8 working days**
Clinical diagnosis: **Cancer of the right breast.**

Examination performed on:

Results of immunohistochemical examination:

Estrogen receptors found in over 75% of neoplastic cell nuclei.
Progesterone receptors found in over 75% of neoplastic cell nuclei.
HER2 protein stained with HercepTest™ by DAKO. Negative reaction in invasive cancerous cells (Score = 1+).

Compliance validated by:

Examination performed on:

Macroscopic description:

Right breast sized 26 x 17 x 7 cm removed along with axillary tissues sized 10 x 14 x 6 cm and a skin flap of 16 x 8 cm. Tumour sized 3.2 x 2.5 x 1.9 cm in the upper outer quadrant, located 4.5 cm from the upper boundary, 1.7 cm from the base and 1.2 cm from the skin.
Seven separate pieces with 3 x 2 x 1 cm in total.

Microscopic description:

Carcinoma ductale invasivum NHG2 (2 + 3 + 1/15 mitoses/10 HPF - visual area 0.55 mm). Extensive fibrosis in the central part of the tumour. Numerous signet ring celi. Mamilla sine laesionibus.
Glandular tissue showing lesions of the type mastopathia fibrosa et lipomatosis.
Axillary lymph nodes: Metastases carcinomatosae in lymphonodis (No II/VII).

Histopathological diagnosis:

Invasive ductal carcinoma of the right breast.
Metastases carcinomatosae in lymphonodis axillae (No II/VII). (NHG2; pT2; pNla). Cancer metastases in axillary lymph nodes.

Compliance validat

CONTACT YOUR DOCTOR WITH THIS REPORT!

UUID:D9A0E659-99BC-4704-AC85-E63098BA731D
TCGA-D8-A1XR-01A-PR Redacted

Criteria	Yes	No
Diagnosis Discrepancy		X
Primary Tumor Site Discrepancy		X
HIPAA Discrepancy		X
Prior Malignancy History		X
Dual/Synchronous Primary Noted		X
Case is (circle): QUALIFIED / DISQUALIFIED		
Reviewer Initials	Date Reviewed: 4/12/11	

Department of Cancer Pathology

copy No.

Date:

Examination: Histopathological examination

Internal invoice No. Value of diagnostic procedure **Examination No.**:

Patient: **XXX** PESEL: **XXX** Age: Gender: **F**

ICD-O-3
Carcinoma, infiltrating duct, NOS 8500/3
Site: breast, NOS C50.9 [illegible] 4/12/11

Material: **Multiple organ resection – left breast**

Unit in charge

Physician in charge:

Material collected on: Material received on:

Expected time of examination: **up to 8 working days**

Clinical diagnosis:

Examination performed on:

Results of immunohistochemical examination:

Estrogen receptors found in over 75% of neoplastic cell nuclei. Progesterone receptors found in over 75% of neoplastic cell nuclei. HER2 protein stained with HercepTest™ by DAKO. Positive reaction in invasive cancerous cells (Score = 3+).

Compliance validated by:

Examination performed on:

Macroscopic description:

Left breast sized 30.4 x 22.2 x 8.2 cm removed without axillary tissues and with a skin flap of 32.1 x 12.8 cm. Weight 1760 g. Tumour sized 2.1 x 1.8 x 1.7 cm found in the upper outer quadrant, 5.9 cm from the upper boundary, 2.6 cm from the base and 2.1 cm from the skin.

Microscopic description:

Carcinoma ductale invasivum – NHG2 (2+3+1/0 mitoses/10 HPF - visual area: 0.55mm).

Mamilla sine laesionibus. Glandular tissue showing parenchymal atrophy.

AXILLARY LYMPH NODES:

Lymphonodulitis chronica (NO III).

Histopathological diagnosis:

Carcinoma ductale invasivum mammae sinistrae (NHG2, pT2, pNO). Invasive ductal carcinoma of the left breast.

Compliance validated by:

CONTACT YOUR DOCTOR WITH THIS REPORT!

UUID:9D1EDFE8-FA4E-4F6C-99E9-1FEB12175D6C
TCGA-D8-A1XY-01A-PR Redacted

Criteria	Yes	No
Diagnosis Discrepancy		X
Primary Tumor Site Discrepancy		X
HIPAA Discrepancy		X
Prior Malignancy History		X
Dual/Synchronous Primary Noted		X
Case is (circle): QUALIFIED / DISQUALIFIED		
Reviewer Initials: [illegible] Date Reviewed: 4/12/11		

Department of Cancer Pathology

copy No.

Date:

Examination: Histopathological examination

Internal invoice No.

Cost of diagnostic procedure

Examination No.:

Patient: XXX PESEL: XXX Age: ender: F

ICD-0-3
carcinoma, infiltrating duct, NOS 8500/3
Site: breast, NOS C50.9 [illegible] 4/12/11

Material: **Total organ resection - left breast and axillary tissues**

Unit in charge:

Physician in charge:

Material collected on: Material received on:

Expected time of examination: **up to 8 working days**

Clinical diagnosis:

Examination performed on:

Macroscopic description:
Left breast sized 24 x 19.5 x 5 cm removed with axillary tissues sized 10 x 7 x 2.5 cm and a skin flap of 24 x 12 cm. Tumour sized 1.9 x 1.3 x 1.4 found in the subaurolar part, located 5.2 cm from the upper boundary, 2.8 cm from the base and 0 cm from the skin. Metastatic lymph nodes 2 cm in length.

Microscopic description:
Carcinoma ductale invasivum - NHG2 (3 + 2 + 1 / 6 mitoses/10 HPF - visual area 0.55 mm).
Foci of carcinoma ductale in situ DCIS detected within the tumour (cribrate and solid type with high nuclear atypia with comedo necrosis, 5% of the tumour).
Mamilla sine laesionibus. Glandular tissue showing lesions of the type mastopathia fibrosa.
AXILLARY LYMPH NODES
Metastases carcinomatosae in lymphonodis (No IX/XV). Infiltratio capsulae lymphonodis et telae perinodalis.

Histopathological diagnosis:
Carcinoma ductale invasivum et ductale in situ mammae sinistrae. Invasive ductal and in situ ductal carcinoma of the left breast.
Metastases carcinomatosae in lymphonodis axillae (No IX/XV). Cancer metastases of the axillary lymph nodes (No IX/XV).
(NHG2, pTlc, pN2a).

UUID:53ED4388-8CD2-4925-81A9-9C4792C83566
TCGA-D8-A1Y0-01A-PR Redacted

Criteria	Yes	No
Diagnosis Discrepancy		X
Primary Tumor Site Discrepancy		X
HIPAA Discrepancy		X
Prior Malignancy History		X
Dual/Synchronous Primary Noted		X
Case is (circle): QUALIFIED / DISQUALIFIED		
Reviewer Initials [illegible]	Date Reviewed: 4/12/11	

Compliance validated by:
Examination performed on:

Results of immunohistochemical examination:
Estrogen receptors found in over 75% of neoplastic cell nuclei. Progesterone receptors found in over 75% of neoplastic cell nuclei.
HER2 protein stained with HercepTestTM by DAKO.
Negative reaction in invasive cancerous cells (Score = 1+)

CONTACT YOUR DOCTOR WITH THIS REPORT!

ICD-0-3
Carcinoma, infiltrating duct, NOS
8500/3
Site: breast, NOS
C50.9
11/22/10

UUID:0CF5DE3C-272C-4127-8047-4359D1278181
TCGA-D8-A13Y-01A-PR
Redacted

Department of Cancer Pathology

copy No.
Date:

Examination: Histopathological examination

Internal invoice No. Value of diagnostic procedure Examination No.:

Patient: [redacted] PESEL: Age: Gender: F

Material: **Multiple organ resection – right breast**
Unit in charge:
Physician in charge:
Material collected on: Material received on
Expected time of examination: up to 8 working days
Clinical diagnosis: **Cancer of the right breast.**

Examination performed on:
Results of immunohistochemical examination:
Estrogen receptors found in 10-75% of neoplastic cell nuclei. Progesterone receptors found in 10-75% of neoplastic cell nuclei. HER2 protein stained with HercepTest™ by DAKO. Negative reaction in invasive cancerous cells (Score = 1+)

Compliance validated by

Examination performed on:
Macroscopic description:
Right breast, sized 16.6 x 12.4 x 4.8 cm, removed along with axillary tissues sized 8 x 6 x 2 cm and a skin flap of 10,7 x 8,4 cm. Weight 513 g.
Tumour sized 1,5 x 1,9 x 1,0 cm in the upper outer quadrant, placed 3.3 cm from the upper edge, 0.1 cm from the base and 0.8 cm from the skin.

Microscopic description:
Carcinoma ductale invasivum - NHG3 (3 + 3 +3: 52 mitoses/ 10 HPF, visual area diameter: 0.55 m). Tumour of "basal - like carcinoma" morphology ? Ma mi 11 a sine laesionibus.
Glandular tissue showing lesions of the type mastopathia fibrosa et cystica.
Additional parenchyma atrophy.
AXILLARY LYMPH NODES
Lymphonodulitis chronica et sinus histiocytosis lymphonodorum (No VI).

Examination result:
Carcinoma ductale invasivum mammae dextrae. (NHG3, pTlc, pNO).
Tumour of "basal - like carcinoma" morphology.

DUCTAL INVASIVE CARCINOMA OF THE RIGHT BREAST.

Compliance validated by:

Criteria	Yes	No
Diagnosis Discrepancy		X
Primary Tumor Site Discrepancy		X
HIPAA Discrepancy		X
Prior Malignancy History		X
Dual/Synchronous Primary Noted		X
Case is (circle): QUALIFIED / DISQUALIFIED		
Reviewer Initials	Date Reviewed: / /	

ICD-0-3
Carcinoma, infiltrating duct, NOS 8500/3
Site: breast, NOS C50.9 5/19/11

UUID:5E997487-9A03-4723-B1E6-FA0B557D8FDF
TCGA-D8-A27R-01A-PR Redacted

original

Examination: Histopathological examination

Internal invoice No. Value of diagnostic procedure **Examination No.**

Patient: XXX PESEL: XXX Age: Gender: F

Material: **Total organ resection – right breast with axillary tissues**

Unit in charge:

Physician in charge:

Material collected on: Material received on:

Expected time of examination: **up to 8 working days**

Clinical diagnosis: **Cancer of the right breast**

Criteria	Yes	No
Diagnosis Discrepancy		
Primary Tumor Site Discrepancy		X
HIPAA Discrepancy		X
Prior Malignancy History		X
Dual/Synchronous Primary Noted		X
Case is (circle):	QUALIFIED	DISQUALIFIED
Reviewer Initials: KM	Date Reviewed: 5/19/11	

Examination performed on

Macroscopic description:

Right breast sized 14.4 x 11.2 x 4.2 cm removed along with axillary tissues sized 8 x 5 x 2 cm and a skin flap of 12,2 x 8.8 cm. Weight 330 g.

Tumour sized 3.2 x 2.3 x 2.6 cm found on the boundary of upper quadrants, located 1.6 cm from the upper boundary, 0.5 cm from the base and 1.2 cm from the skin.

Microscopic description:

Carcinoma ductale invasivum - NHG3 (3 + 3 + 3 / 2 0 mitoses/10 HPF - visual area: 0.55mm).

Numerous foci of carcinoma ductale in situ DCIS found within the tumour (solid type with high nuclear atypia and comedo necrosis with calcinations, 20% of the tumour volume).

Invasio carcinomatosa vasovum massiva.

Emboliae carcinomatosae mamillae.

Glandular texture showing parenchyma of normal structure.

In situ lesions removed by 0.1 cm from the base

AXILLARY LYMPH NODES:

Metastases carcinomatosae in lymphonodis (No XI/XI).

Infitratio telae perinodalis.

Emboliae carcinomatosae vasorum.

Test result:

Incl. Examination

Carcinoma ductale invasivum mammae dextrae. Invasive ductal carcinoma of the right breast

Metastases carcinomatosae in lymphonodis axillae. Cancer metastases in axillary lymph nodes. (No XI/XI) (NHG3, pT2, pN3a).

Invasio carcinomatosa vasorum massiva. Massive vascular invasion.

Compliance validated by:

Examination performed on

Examination: Histopathological examination

Examination No.:

Patient: **XXX** PESEL: **XXX** Gender: **F**

Examination performed on

Results of immunohistochemical examination:

Estrogen receptors found in over 75% of neoplastic cell nuclei.

Progesterone receptors found in 10-75% neoplastic cell nuclei.

HER2 protein stained with Ventana's Pathway HER-2/neu (4B5) Rabbit Monoclonal Antibody. Score=2+, FISH verification recommended.

dr

Compliance validated by

ICD O-3
Infiltrating duct Carcinoma, NOS
8500/3
Site: breast, NOS
C50.9
11/22/10

Department of Cancer Pathology

copy No.
Date:

Examination: Histopathological examination

Examination No.:

Patier PESEL: Age: Gender: **F**

Material: **Partial organ resection – lesion from the left breast – lower outer quadrant**
Unit in charge
Physician in charge:
Material collected on: Material received on:
Expected time of examination:
Clinical diagnosis: **Cancer of the right breast, typical quadrant marking**

Examination performed on:

Macroscopic description:
Part of the breast sized 8.8 x 8.3 x 2.4 cm with a skin flap of 5.8 x 2.3 cm, marked typically, with an X-ray image. Tumour cross section sized 1.7 x 1.5 x 2.3 cm. Margins: to the base 0.1 cm; do the skin surface 0.6 cm; to the sternum 2.2 cm, to the axilla 3.6 cm, to the shoulder 2.3 cm, lower 4.3 cm.

Microscopic description:
Carcinoma ductale invasivum: NHG3 (3+3+3: 18 mitoses /10 HPF - diam. 0.55mm), pT2. Solitary DCIS focuses found beneath the tumour (solid type with medium atypia, no necrosis) placed 0.5 cm from the incision line.

Histopathological diagnosis:
Resectio partalis mammae dextrae:
Carcinoma ductale invasivum et ductale in situ (NHG3, pT2).
INVASIVE DUCTAL CARCINOMA AND IN SITU DUCTAL CARCINOMA OF THE RIGHT BREAST

Compliance validated by:

Examination performed on:

Results of immunohistochemical examination:
Estrogen receptors in neoplastic cell nuclei not found.
Progesterone receptors in neoplastic cell nuclei not found.
HER2 protein stained with HercepTest™ by DAKO.
Negative reaction in invasive cancerous cells (Score = 0)

Compliance validated by:

UUID:380639A2-E89C-4495-8168-82120617129F
TCGA-D8-A147-01A-PR
Redacted

Criteria	Yes	No
Diagnosis Discrepancy		X
Primary Tumor Site Discrepancy		X
HIPAA Discrepancy		X
Prior Malignancy History		X
Dual/Synchronous Primary Noted		X
Case is (circle): QUALIFIED / DISQUALIFIED		
Reviewer Initials:	Date Reviewed: 5/10/11	

TSS ID

ICD-0-3
Carcinoma, infiltrating ductal, NOS 8500/3
Site: breast, NOS C50.9 2/8/11 [illegible]

UUID:CC0B1145-B242-45BD-A946-B14BA5A9E923
TCGA-E2-A1IN-01A-PR Redacted

SPECIMENS:

A. WLE RIGHT BREAST NEEDLE LOCALIZATION
B. SENTINEL NODE #1
C. SENTINEL NODE #2
D. ADDITIONAL LATERAL POSTERIOR MARGIN

SPECIMEN(S):

A. WLE RIGHT BREAST NEEDLE LOCALIZATION
B. SENTINEL NODE #1
C. SENTINEL NODE #2
D. ADDITIONAL LATERAL POSTERIOR MARGIN

INTRAOPERATIVE CONSULTATION DIAGNOSIS:

A-WLE right breast: Gross examination only, tumor is 0.8 cm from the nearest superior margin.
TPB/TPC-SLN #1, #2: Touch imprints only, negative for tumor cells.
Diagnoses called by Dr. to Dr. at (A, B, C).

GROSS DESCRIPTION:

A. WLE RIGHT BREAST NEEDLE LOCALIZATION
Received fresh labeled with the patient's identification and "WLE right breast needle localization" is a previously inked, oriented (single suture-anterior, double suture-lateral) 76 g, 3.7 x 3.6 x 2.9 cm needle localized lumpectomy with radiograph. Ink code: Anterior-yellow, posterior-black, medial-green, lateral-red, inferior-blue, inferior-orange. The specimen is serially sectioned from lateral to medial into 7 slices revealing a 1.8 x 1.3 x 1.1 cm tan, stellate nodule that is closest to the superior margin at 0.8 cm. Tissue is procured. Representatively submitted:
A1-A2: lateral margin, perpendicular sections
A3: slice 2, anterior superior
A4: slice 2, posterior superior
A5: slice 2, posterior superior
A6: slice 2, mid posterior
A7: slice 3, anterior superior (mass)
A8: slice 3, posterior superior (mass)
A9: slice 3, mid anterior (mass)
A10: slice 3, mid posterior (mass)
A11: slice 3, inferior
A12: slice 4, mid anterior (mass)
A13: slice 4, mid posterior (mass)
A14: slice 4, mid inferior
A15: slice 4, posterior
A16: slice 5, anterior
A17: slice 5, inferior
A18: slice 5, posterior
A19: slice 6, superior
A20-A21: slice 6, anterior
A22: slice 6, inferior
A23: slice 6, posterior
A25-A25: medial margin, perpendicular sections
B. SENTINEL LYMPH NODE #1 RIGHT AXILLA
Received fresh labeled with the patient's identification and "SLN #1" is a 1.5 x 0.7 x 0.4 cm lymph node. It is sectioned, a touch prep is performed, lymph node is submitted entirely in cassette B1.
C. SENTINEL LYMPH NODE #2 RIGHT AXILLA
Received fresh labeled with the patient's identification and "SLN #2" it is a 1.7 x 0.3 x 0.1 cm lymph node. It is sectioned, a touch prep is performed, submitted entirely in cassette C1.
D. ADDITIONAL LATERAL POSTERIOR MARGIN
Received fresh labeled with the patient's identification and "additional lateral-posterior margin" is an oriented (suture at final margin) 9 g, 5 x 3 x 1.2 cm fibrofatty tissue. Final margin is inked blue. Serial sectioning reveals no discrete lesions. Entirely submitted in cassettes B1-B8.

DIAGNOSIS:

A. BREAST, RIGHT, WIDE LOCAL EXCISION:
- INVASIVE DUCTAL CARCINOMA, SBR GRADE 3, MEASURING 1.8-CM

TSS ID

- HIGH NUCLEAR GRADE, DUCTAL CARCINOMA IN SITU, SOLID AND CRIBRIFORM TYPES WITH MICROCALCIFICATIONS
- INVASIVE TUMOR PRESENT WITHIN 1-MM FROM INFERIOR SURGICAL RESECTION MARGIN
- LOBULAR CARCINOMA IN SITU
- SEE SYNOPTIC REPORT.

B. LYMPH NODE, SENTINEL #1, RIGHT AXILLA, EXCISION:
- ONE LYMPH NODE, NEGATIVE FOR METASTASES (0/1).

C. LYMPH NODE, SENTINEL #2, RIGHT AXILLA, EXCISION:
- ONE LYMPH NODE, NEGATIVE FOR METASTASES (0/1).

D. BREAST, ADDITIONAL LATERAL POSTERIOR MARGIN, EXCISION:
- BREAST TISSUE, NO TUMOR SEEN.

SYNOPTIC REPORT - BREAST
Specimen Type: Excision
Needle Localization: Yes - For mass
Laterality: Right
Invasive Tumor: Present
Multifocality: No
WHO CLASSIFICATION
Invasive ductal carcinoma, NOS 8500/3
Tumor size: 1.8cm
Margins: Negative
Distance from closest margin: Less than 0.1cm
inferior
Tubular Score: 3
Nuclear Grade: 2
Mitotic Score: 3
Modified Scarff Bloom Richardson Grade: 3
Necrosis: Absent
Vascular/Lymphatic Invasion: None identified
Lobular neoplasia: LCIS
Lymph nodes: Sentinel lymph node only
Lymph node status: Negative 0 / 2

DCIS present
Margins uninvolved by DCIS
DCIS Quantity: Estimate 10%
DCIS Type: Solid
Cribriform
DCIS Location: Associated with invasive tumor
Nuclear grade: High
Necrosis: Absent
Location of CA++: DCIS
Benign epithelium

ER/PR/HER2 Results
ER: Positive
PR: Positive
HER2: Negative by IHC
Performed on Case:

Pathological staging (pTN): pT 1c N 0
Pathological staging is based on the AJCC Cancer Staging Manual, 7th Edition
Distant Metastases (M): MX: Cannot be assessed

CLINICAL HISTORY:
None given

TSS ID

PRE-OPERATIVE DIAGNOSIS:
None given

ADDENDUM:
ONCOTYPE DX BREAST CANCER ASSAY

RESULTS: Recurrence Score: 14
CLINICAL EXPERIENCE: Patients with a recurrence score of: 14 in the clinical validation study had an average rate of Distant Recurrence at 10 years of 9%

ER Score: 11.3 Positive
PR Score: 9.1 Positive
Her2 Score: 9.1 Negative

Interpretation:

ER	Negative < 6.5	Positive >= 6.5	
PR	Negative < 5.5	Positive >= 5.5	
Her2	Negative <10.7	Positive >=11.5	Equivocal = 10.7 - 11.4

Microscopic/Diagnostic Dictation:, ,
Final Review:, M.D., Pathologist,
Final: M.D., Pathologist, (
Addendum: M.D., Pathologist, (
Addendum Final: M.D., Pathologist,

Criteria	Yes	No
Diagnosis Discrepancy		✓
Primary Tumor Site Discrepancy		✓
HIPAA Discrepancy		✓
Prior Malignancy History		✓
Dual/Synchronous Primary Noted		✓
Case is (circle): QUALIFIED / DISQUALIFIED		
Reviewer Initials [illegible] Date Reviewed: [illegible]		

ICD-0-3
Carcinoma, infiltrating ductal, NOS 8500/3
Path Site: breast, lower outer quadrant C50.5
CQCF Site: breast, NOS C50.9 2/15/11

TSS: -

SPECIMENS:
A. SENTINEL LYMPH NODE 1 LEFT AXILLA
B. SENTINEL LYMPH NODE 2 LEFT AXILLA
C. SENTINEL LYMPH NODE 3 LEFT AXILLA
D. LEFT BREAST

DIAGNOSIS:
A. SENTINEL LYMPH NODE #1 LEFT AXILLA, EXCISION:
- METASTATIC ADENOCARCINOMA, IDENTIFIED IN ONE LYMPH NODE (MICROMETASTASES, 1.8 MM IN GREATEST DIAMETER).

B., C. SENTINEL LYMPH NODES #2-3, LEFT AXILLA, EXCISION:
- TWO LYMPH NODES, NEGATIVE FOR MALIGNANCY (0/2).

D. LEFT BREAST, MASTECTOMY:
- WELL DIFFERENTIATED INFILTRATING DUCTAL CARCINOMA, (1.5 CM IN GREATEST DIAMETER, SBR GRADE I).
- MULTIFOCAL INTERMEDIATE GRADE DUCTAL CARCINOMA IN SITU (CRIBRIFORM, MICROPAPILLARY, AND PAPILLARY PATTERNS).
- PROLIFERATIVE TYPE FIBROCYSTIC CHANGES.
- ATYPICAL DUCTAL HYPERPLASIA.
- ATYPICAL LOBULAR HYPERPLASIA.
- CHANGES CONSISTENT WITH PREVIOUS BIOPSY SITE, UPPER INNER QUADRANT, WITH ORGANIZING HEMATOMA.
- THREE ADDITIONAL LYMPH NODES, NEGATIVE FOR TUMOR (0/3). SEE COMMENT.

SPECIMEN(S):
A. SENTINEL LYMPH NODE 1 LEFT AXILLA
B. SENTINEL LYMPH NODE 2 LEFT AXILLA
C. SENTINEL LYMPH NODE 3 LEFT AXILLA
D. LEFT BREAST

UUID:F23523C7-B6E9-4205-BD36-D76E9EE9C453
TCGA-E2-A1L6-01A-PR
Redacted

CLINICAL HISTORY:
year old with left breast ca

GROSS DESCRIPTION:
A. SENTINEL LYMPH NODE 1 LEFT AXILLA
Received fresh for touch prep evaluation labeled with the patient name designated "A - sentinel lymph node #1 left axilla" is a beige-tan lymph node measuring 1.6 x 1.2 x 0.7 cm. The specimen is bisected, touch preps are performed. The entire specimen is submitted in a cassette labeled A1.
B. SENTINEL LYMPH NODE 2 LEFT AXILLA
Received fresh for touch prep evaluation labeled with the patient name designated "B – sentinel lymph node #2 left axilla" is a fragment of yellow beige soft tissue measuring 2.2 x 1.8 x 0.5 cm. A tan lymph node is identified and measures 0.6 x 0.5 x 0.4 cm. The lymph node is bisected, touch preps are performed. The entire specimen is submitted in cassette labeled B1.

C. SENTINEL LYMPH NODE 3 LEFT AXILLA
Received fresh for touch prep evaluation labeled with the patient name designated "C – sentinel lymph node #3 left axilla" is a tan lymph node measuring 1.3 x 0.9 x 0.5 cm. The specimen is bisected, touch preps are performed. The entire specimen is submitted in a cassette labeled C1.
D. LEFT BREAST
Received fresh for tissue procurement labeled with the patient name designated "D – left breast" is a mastectomy specimen weighing 228 grams measuring 15.3 x 15.0 x 3.0 cm. The axilla measures 5.0 x 3.5 x 1.0 cm. The specimen is received with orientation, a black suture indicating the axillary tail. The specimen is inked as follows: posterior deep margin, black; anterior, blue. The overlying ellipse of beige-tan skin measures 8.0 x 2.5 cm. The light beige areola measures 2.0 cm in diameter. The everted nipple measures 0.9 cm in diameter. The specimen is serially sectioned from medial to lateral.

TSS:

Cut section shows a firm, granular beige-tan mass in the lower outer quadrant approaching the deep margin at a distance of 0.2 cm. The mass measures 1.5 x 1.3 x 1.2 cm and is located 4.5 cm from the axilla. Extending from the area of the mass through the central portion and to the medial, beige-tan fibrous parenchyma is demonstrated. In the upper inner quadrant there is a hemorrhagic well-circumscribed area measuring 2.4 x 1.6 x 1.0 cm. This area approaches the deep margin at a distance of 0.2 cm and is located 4.8 cm from the lesion. A portion of the specimen is submitted for tissue procurement. Representative sections are submitted as follows:

- D1-D4: sections of the lesion and overlying posterior margin lower outer quadrant
- D5-D9: representative sections of central fibrous tissue
- D10-D17: the entire hemorrhagic area in the upper inner quadrant
- D18-D20: representative sections from the upper outer quadrant
- D21-D23: representative sections from the lower inner quadrant
- D24-D25: sections of nipple
- D26: representative section of skin
- D27-D30: possible axillary lymph nodes

COMMENT:

Re-examination of the original touch-prep examined at the time of the intra-operative consultation again was interpreted as no evidence of malignant cells on this slide. The permanent sections from the lymph node however, show a micrometastases, (1.8 mm). Gross examination of the breast reveals an ill defined mass in the lower outer quadrant measuring 1.5 x 1.3 x 1.0 cm in greatest extent. In addition, in the upper inner quadrant there is a hemorrhagic area measuring 2.4 x 1.6 x 1 cm.

BREAST CANCER TEMPLATE:

Specimen type:	Mastectomy
Needle localization:	No
Laterality:	Left
INVASIVE TUMOR:	Present
Multifocal:	No
Histologic type:	Ductal
Tumor Size (cm):	1.5 x 1.3 x 1.2 cm
Tumor site:	Lower outer quadrant
Grade, Tubular:	2
Grade, Nuclear:	2
Grade, Mitotic:	1
Modified Scarff Bloom Richardson grade:	I
Necrosis:	Absent
Invasion Vasc/Lymphatic:	None identified
DCIS COMPONENT:	
DCIS Quantity:	Estimated 20%
DCIS Type:	*Cribriform, micropapillary and papillary*
DCIS Location:	DCIS is associated with the invasive tumor with separate foci seen away from the invasive tumor in the central region of the breast
Nuclear grade:	Intermediate
Necrosis:	None identified
Location of Ca++:	DCIS and benign epithelium
Margins:	Negative.
Distance from closest margin:	DCIS extends to within 2 mm of *carcinoma extends to within 3 mm* of the deep margin

TSS

Specimens Involved
Specimens: D: LEFT BREAST

HER2 Status Results, Immunohistochemistry Evaluation
SPECIMEN
Surgical Excision
Block Number: Block
D4
TEST RESULTS
Interpretation: Negative
Intensity: 1+
% Tumor Staining: 40%
FISH ORDERED
No
METHODOLOGY
Methodology: Fixation Type and Length: Tissue was fixed in 10% neutral buffered formalin) for no less than 8 and no longer than 24 hours. Antibody and Assay Methodology: Rabbit anti-human HER2, HerceptestTM (FDA-approved test kit), Control Slides Examined: External kit-slides provided by manufacturer (cell lines with high, low and negative HER2 protein expression), and in-house known HER2 amplified control tissue were evaluated along with the test tissue. These control slides run along side of this patient's sample showed appropriate staining. Adequacy of Specimen: Adequate, well preserved, clear-cut invasive carcinoma identified for HER2 evaluation.
Scoring Criterion and Scoring System:
IHC Level of Expression(Score) /Tumor Cell Membrane Staining Pattern
Negative (0)/Absence of Staining
Negative (1+)/Faint Incomplete membrane Staining, >10% of Cells
Equivocal (2+)/Weak complete membrane Staining, >10% of Cells
Positive (3+)/Strong complete membrane Staining, >10% of Cells
Equivocal Category for HER2 IHC results: A HER2, 2+ staining result that is interpreted as equivocal may not indicate gene amplification. A FISH test for HER2 gene amplification will be ordered for all HER2 IHC 2+ results.
COMMENT
HER2 analysis was performed on this case by immunohistochemistry utilizing the FDA approved HercepTest (TM) test kit following the manufacturer's instructions listed in the package insert. This assay was not modified, and adherence to all instruction and guidelines were strictly followed. Interpretation of the HER2 immunohistochemical staining characteristics is guided by published results in the medical literature (4), information provided by the reagent manufacturer and by internal review of staining performance within Pathology Department.
HER2 TEST VALIDATION
This HER2 immunohistochemical assay has been validated according to the recently revised recommendations and guidelines from the NCCN HER2 testing in Breast Cancer Task Force, and the jointly issued recommendations and guidelines from ASCO and the CAP (5). 80 randomly selected breast cancer samples were tested for HER2 by IHC as outline above and interpreted as, negative (score 0/1+) equivocal (score 2+) and positive (score 3+) without knowledge of the previous reported results.

These cases were also blindly read using two different FISH assay as amplified or non-amplified and the HER2/CEP17 ratios were recorded. After analyzing these results, there was 100% concordance between the IHC and FISH results for cases that were interpreted as either positive or negative by IHC. 9 of the 80 cases were interpreted as equivocal by IHC and of these 3/9 (33%) were non-amplified by FISH and 6/9 (66%) were found to be amplified.

The Pathology Department Immunohistochemistry laboratory takes full responsibility for this tests performance and has programs in place to regularly monitor the proficiency and the interpretation of HER2 assays. The laboratory also participates in external quality assurance HER2 programs including the CAP proficiency testing program.
REFERENCE
1. Carlson RW, Anderson BO, Burstein HJ, et al., NCCN breast cancer clinical practice guidelines in oncology. J Natl Compr Canc Netw. 2005;3:238-289.

TSS

Lobular Neoplasia: Atypical lobular hyperplasia

Lymph nodes: Sentinel lymph nodes and axillary lymph nodes one positive, (1/6). Micrometastases (1.8 mm in greatest diameter, negative for extranodal extension)

Non-neoplastic areas: Atypical ductal hyperplasia, atypical lobular hyperplasia, columnar cell change with foci of

BREAST TUMOR BIOMARKERS TEMPLATE

Immunohistochemistry for ER/PR and Her-2 have been ordered on block D4, and the results will be issued as an addendum.

Pathologic Stage : pT1c pNimi

This assay can be used to select invasive breast cancer patients for hormone therapy (1).
ER and PR analysis was performed on this case by immunohistochemistry utilizing the ER (ER 1D5, 1:100) and PR (PGR 136, 1:100) antibody provided by following the manufacturer's instructions listed in the package insert. This assay was not modified, and adherence to all instruction and guidelines were strictly followed. Interpretation of the ER/PR immunohistochemical staining characteristics is guided by published results in the medical literature (1), information provided by the reagent manufacturer and by internal review of staining performance within the Pathology Department.

1. Harvey JM, et al. Estrogen receptor status by immunohistochemistry is superior to the ligand-binding assay for predicting response to adjuvant endocrine therapy in breast cancer. J Clin Oncol. 17:1474-1481, 1999

ADDENDUM:

BREAST ER/PR -1
Specimens Involved
Specimens: D: LEFT BREAST

SPECIMEN
Type: Surgical Excision
Block Number: D4
HORMONE RECEPTOR STATUS
Laboratory:
Estrogen Receptor: Positive
Allred Score: 8 = Proportion score 5 + Intensity score 3
Progesterone Receptor: Positive
Allred Score: 8 = Proportion Score 5 + Intensity Score 3
The Allred score for estrogen and progesterone receptors is calculated by adding the sum of the proportion score (0 = no staining, 1 = <1% of cells staining, 2 = 1 - 10% of cells staining, 3 = 11-30% of cells staining, 4 = 31-60% of cells staining, 5 = >60% of cells staining) to the intensity score (1 = weak intensity of staining, 2 = intermediate intensity of staining, 3 = strong intensity of staining), with a scoring range from 0 to 8.
ER/PR positive is defined as an Allred score of >2 and ER/PR negative is defined as an Allred score of less than or equal to 2.
Methodology: Fixation Type and Length: Tissue was fixed in 10% neutral buffered formalin for no less than 8 and no longer than 24 hours. Antibody and Assay Methodology: Mouse anti-human ER and PR,

TSS

2. Carlson RW, Brown E, Burstein HJ, et al., NCCN Task Force Report: adjuvant therapy for breast cancer. J Natl Compr Canc Netw. 2006;4:S1-S26.
3. Romond EH, Perez EA, Bryant J, et al. Trastuzumab plus adjuvant chemotherapy for operable HER2-positive breast cancer. N Eng J Med 2005;353(16):1673-84
4. Leong ASY, Formby M, Haffajee Z, et al. Refinement of immunohistologic parameters for Her2/neu scoring validation by FISH and CISH. Appl Immunohistochem Mol Morphol. 2006;14:384-389.
5. Wolff AC, Hammond EH, Schwartz JN, et al., American Society of Clinical Oncology/College of American Pathologists Guideline Recommendations for Human Epidermal Growth Factor Recepto 2 Testing in Breast Cancer. Arch of Path and Lab Med 2007; 131:18-43.

Gross Dictation:
Microscopic/Diagnostic Dictation: PATHOLOGIST,
Microscopic/Diagnostic Dictation: PATHOLOGIST
Final Review: PATHOLOGIST,
Final: PATHOLOGIST,
Addendum:,
Addendum Review: PATHOLOGIST
Addendum Final: PATHOLOGIST
Addendum: PATHOLOGIST, 0
Addendum Review: PATHOLOGIST
Addendum Final: PATHOLOGIST,

Criteria	Yes	No
Diagnosis Discrepancy		/
Primary Tumor Site Discrepancy		/
HIPAA Discrepancy		/
Prior Malignancy History		/
Dual/Synchronous Primary Noted		/
Case is (circle): QUALIFIED / DISQUALIFIED		
Reviewer Initials [illegible] Date Reviewed: 2/19/11		

TSS

ICD-0-3
Carcinoma, infiltrating ductal, NOS 8500/3
Site: breast, NOS C50.9
2/15/11

UUID:97B6C43B-4C7D-49FC-BA49-00D2DF41827D
TCGA-E2-A1L9-01A-PR
Redacted

SPECIMENS:

A. WLE RIGHT BREAST NEEDLE LOCALIZATION
B. SENTINEL NODE #1 RIGHT AXILLA
C. SENTINEL NODE #2

SPECIMEN(S):

A. WLE RIGHT BREAST NEEDLE LOCALIZATION
B. SENTINEL NODE #1 RIGHT AXILLA
C. SENTINEL NODE #2

INTRAOPERATIVE CONSULTATION DIAGNOSIS:

A-WLE right breast: Mass (1.4 x 1.2 x 1 cm) is 0.8 cm from the closest inferior margin.
TPB/TPC-sentinel lymph nodes #1 & #2 right axilla: Negative for tumor by touch prep.
Diagnoses called by Dr. to Dr. at '

GROSS DESCRIPTION:

A. WLE RIGHT BREAST NEEDLE LOCALIZATION
Received fresh labeled with the patient's identification and "WLE left breast needle localization" is a previously inked, oriented (single-anterior, double-lateral, triple-superior) 53-g, 4 x 3.4 x 2.5 cm needle localized lumpectomy with radiograph. Ink code: Anterior-yellow, posterior-black, medial-green, lateral-red, superior-blue, inferior-orange. Specimen is serially sectioned from lateral to medial into 7 slices revealing a 1.4 x 1.2 x 1 cm firm, tan, stellate mass that is closest to the inferior margin at 0.8 cm. Tissue is procured; Representatively submitted:
A1-A3: lateral margin, perpendicular sections
A4: slice 2, anterior inferior
A5: slice 3, anterior inferior
A6: slice 3, mid inferior (mass)
A7: slice 3, posterior inferior
A8: slice 4, anterior inferior (mass)
A9: slice 4, posterior inferior (mass)
A10: slice 5, anterior superior
A11: slice 5, posterior superior
A12: slice 5, anterior inferior (mass)
A13: slice 5, posterior inferior (mass)
A14: slice 6, anterior inferior (mass)
A15: slice 6, posterior inferior (mass)
A16-A18: medial margin, perpendicular sections (muscle infiltrating the tissue)
B. SENTINEL LYMPH NODE #1 RIGHT AXILLA
Received fresh labeled with the patient's identification and "sentinel lymph node #1 right axilla" is a 4 x 2.3 x 1.5 cm lymph node; sectioned, it has a yellow-tan cut surface. A touch prep is performed; submitted entirely in cassettes B1-B2.
C. SENTINEL LYMPH NODE #2
Received fresh labeled with the patient's identification and "sentinel lymph node #2" is a 3.4 x 3 x 0.4 cm lymph node; sectioned, it has a yellow-tan cut surface. Touch prep is performed; submitted entirely in cassettes C1-C3.

DIAGNOSIS:

A. BREAST, RIGHT, WIDE LOCAL EXCISION:
- INVASIVE DUCTAL CARCINOMA, SBR GRADE 2 WITH FOCAL NECROSIS, MEASURING 1.5-CM
- INTERMEDIATE NUCLEAR GRADE, DUCTAL CARCINOMA IN SITU, SOLID AND CRIBRIFORM TYPES WITH CENTRAL NECROSIS
- SURGICAL RESECTION MARGINS NEGATIVE FOR TUMOR
- BIOPSY SITE CHANGES WITH FIBROSIS AND GRANULATION TISSUE
- SEE SYNOPTIC REPORT.

B. LYMPH NODE, SENTINEL #1, RIGHT AXILLA, EXCISION:
- METASTATIC CARCINOMA TO ONE OF ONE LYMPH NODE (1/1), MEASURING 1.0-MM (MICROMETASTASES) WITH NO EXTRANODAL EXTENSION, SEE NOTE.

C. LYMPH NODE, SENTINEL #2, RIGHT AXILLA, EXCISION:
- ONE LYMPH NODE, NEGATIVE FOR METASTASES (0/1).

TSS:

NOTE: The touch preparation was reviewed, no tumor cells are identified. Therefore, the false negativity is due to sampling error.

SYNOPTIC REPORT - BREAST
Specimen Type: Excision
Needle Localization: Yes - For mass
Laterality: Right
Invasive Tumor: Present
Multifocality: No
WHO CLASSIFICATION
Invasive ductal carcinoma, NOS 8500/3
Tumor size: 1.5cm
Tumor Site: Not specified
Margins: Negative
Distance from closest margin: 0.7cm
inferior
Tubular Score: 2
Nuclear Grade: 2
Mitotic Score: 2
Modified Scarff Bloom Richardson Grade: 2
Necrosis: Present
Vascular/Lymphatic Invasion: Present
Extent: focal
Lobular neoplasia: None
Lymph nodes: Sentinel lymph node only
Lymph node status: Positive 1 / 2
Micrometastases: Yes

DCIS present
Margins uninvolved by DCIS
DCIS Quantity: Estimate 2%
DCIS Type: Solid
Cribriform
DCIS Location: Associated with invasive tumor
Nuclear grade: Intermediate
Necrosis: Present

ER/PR/HER2 Results
ER: Positive
PR: Positive
HER2: Negative by IHC
Performed on Case:

Pathological staging (pTN): pT 1c N 1mi

CLINICAL HISTORY:
None given

PRE-OPERATIVE DIAGNOSIS:
Cancer right breast

Final ReviewPathologist,
Final:., Pathologist, ‘

Criteria	Yes	No
Diagnosis Discrepancy		✓
Primary Tumor Site Discrepancy		✓
HIPAA Discrepancy		✓
Prior Malignancy History		✓
Dual/Synchronous Primary Noted		✓
Case is (circle): QUALIFIED / DISQUALIFIED		
Reviewer Initials [illegible] Date Reviewed: [illegible]		

TSS:

ICD-0-3
Carcinoma, infiltrating ductal, NOS 8500/3

Path Site: breast, upper inner quadrant C50.2 2/15/11

CQCF Site: breast, NOS C50.9

SPECIMENS:
A. NON-SENTINEL NODES RIGHT AXILLA
B. RIGHT BREAST

SPECIMEN(S):
A. NON-SENTINEL NODES RIGHT AXILLA
B. RIGHT BREAST

INTRAOPERATIVE CONSULTATION DIAGNOSIS:
TPA: Lymph nodes, right axillary non-sentinel, biopsy: Two lymph nodes positive for carcinoma on tough prep.
By Dr. called to Dr.

GROSS DESCRIPTION:
A. NON-SENTINEL NODES RIGHT AXILLA
Received fresh and labeled with the patient name designated "A – non-sentinel nodes right axilla", are 2 portions of fibroadipose tissue demonstrating 2 presumptive palpable lymph nodes; one lymph node measures 3.5 x 1.2 x 0.8 cm, the second measures 2.0 x 1.2 x 0.7 cm. Both nodes are bisected. Touch prep performed. One lymph node is submitted in cassette A1 and the second is submitted in cassette A2.

B. RIGHT BREAST
Received fresh and labeled with the patient name designated "B – right breast", is a resected mastectomy specimen weighing 1,383 grams and measuring 29.5 x 22.0 x 4.0 cm. The attached axillary tail measures 13.0 x 7.2 x 1.5 cm. A suture indicates the axillary region. The posterior margin is inked black. The white-beige ellipse of overlying skin measures 21.8 x 11.0. The light beige areola measures 3.5 cm in diameter. The inverted nipple measures 1.0 cm in diameter. The surface of the skin is dense and wrinkled. The specimen is serially sectioned from medial to lateral. Cut section shows two firm beige distinct lesions; the larger lesion is located in the lower inner quadrant in the subareolar region and measures 3.6 x 2.2 x 1.5 cm located 4.4 cm from the smaller lesion. The smaller lesion is firm present in the upper inner quadrant measuring 1.0 x 0.9 x 0.8 cm. The larger lesion is 5.1 cm from the deep margin. The smaller lesion approaches the deep margin at a distance of 2.0 cm. The remainder of the specimen shows dark yellow lobulated adipose tissue. Many firm lymph nodes are demonstrated in the axillary tail ranging in size from 0.5 x 0.5 x 0.4 cm up to 2.5 x 2.0 x 1.0 cm. A portion of the specimen is submitted for tissue procurement. Representative sections are submitted as follows:

B1-B2: The smaller lesion submitted entirely with overlying deep margin
B3-B9: Sections from the larger lesion
B10: Margin overlying the larger lesion
B11-B13: Sections of nipple
B14: Section of skin adjacent to nipple
B15: Additional section of skin
B16-B17: Representative sections upper outer quadrant
B18-B19: Representative sections lower outer quadrant
B20: Additional section upper inner quadrant
B21-B22: One bisected lymph node
B23: One-half of one bisected lymph node
B24: One bisected lymph node
B25: Three possible lymph nodes
B26: Three possible lymph nodes
B27: Four possible lymph nodes
B28: Four possible lymph nodes
B29: Four possible lymph nodes
B30: One lymph node
B31: One lymph node
B32: One lymph node

UUID:42E3E8C1-D18A-4457-B2F0-FE49F4C027D7
TCGA-E2-A1LE-01A-PR Redacted

DIAGNOSIS:
A. LYMPH NODE, NON-SENTINEL, RIGHT AXILLA, BIOPSY:
- METASTATIC CARCINOMA TO TWO OF TWO LYMPH NODES (2/2), MEASURING 2-CM, WITH EXTRANODAL EXTENSION.

TSS:

B. BREAST, RIGHT, MASTECTOMY AND AXILLARY NODE DISSECTION:
- MULTIFOCAL, INVASIVE, DUCTAL CARCINOMA, SBR GRADE 3, LARGEST MEASURING 3.6-CM, PRESENT IN THE LOWER INNER AND UPPER INNER QUADRANTS AND INVOLVES NIPPLE AND ADJACENT SKIN
- SURGICAL RESECTION MARGINS NEGATIVE FOR TUMOR
- EXTENSIVE LYMPHOVASCULAR INVASION IDENTIFIED
- METASTATIC CARCINOMA TO TWENTY FIVE OF TWENTY SIX LYMPH NODES (25/26), LARGEST MEASURING 2.1-CM, WITH EXTENSIVE EXTRANODAL EXTENSION
- SEE SYNOPTIC REPORT AND SEE NOTE.

NOTE: Breast biomarkers have been ordered and addendum report to follow.

SYNOPTIC REPORT - BREAST
Specimen Type: Mastectomy
Needle Localization: No
Laterality: Right
Invasive Tumor: Present
Multifocality: Yes
Tumor size: 3.6cm
Tumor Site: Upper inner quadrant
Lower inner quadrant and nipple and adjacent skin
Margins: Negative
Tubular Score: 3
Nuclear Grade: 3
Mitotic Score: 3
Modified Scarff Bloom Richardson Grade: 3
Necrosis: Absent
Vascular/Lymphatic Invasion: Present
Extent: extensive
Lobular neoplasia: None
Lymph nodes: Axillary dissection
Lymph node status: Positive 27 / 28 Extranodal extension

DCIS not present

ER/PR/HER2 Results
ER: Pending
PR: Pending
HER2: Pending

Pathological staging (pTN): pT 2 N 3a

CLINICAL HISTORY:

A -year-old Caucasian female post menopausal abnormal mammogram. Biopsy showed an adenocarcinoma, ER/PR-, Her2+, 2 masses at right breast seen. One at 12 o'clock position subareolar, 2nd mass at 1 o'clock posterior to 1st mass (3.0 cm).

PRE-OPERATIVE DIAGNOSIS:

Infiltrating adenocarcinoma

ADDENDUM:

SYNOPTIC REPORT - BREAST, ER/PR RESULTS
Specimen: Surgical Excision
Block Number: B4

ER: Negative Allred Score: 0 = Proportion Score 0 + Intensity Score 0
PR: Negative Allred Score: 0 = Proportion Score 0 + Intensity Score 0

TSS:

COMMENT:

The Allred score for estrogen and progesterone receptors is calculated by adding the sum of the proportion score (0 = no staining, 1 = <1% of cells staining, 2 = 1 - 10% of cells staining, 3 = 11-30% of cells staining, 4 = 31-60% of cells staining, 5 = >60% of cells staining) to the intensity score (1 = weak intensity of staining, 2 = intermediate intensity of staining, 3 = strong intensity of staining), with a scoring range from 0 to 8.

ER/PR positive is defined as an Allred score of >2 and ER/PR negative is defined as an Allred score of less than or equal to 2.

METHODOLOGY:

Tissue was fixed in 10% neutral buffered formalin for no less than 8 and no longer than 24 hours. Immunohistochemistry was performed using the mouse anti-human ER (ER 1D5, 1:100) and PR (PGR 136, 1:100) provided by following the manufacturer s instructions. This assay was not modified. Interpretation of the ER/PR immunohistochemical stain is guided by published results in the medical literature, information provided by the reagent manufacturer and by internal review of staining performance.

SYNOPTIC REPORT - BREAST HER-2 RESULTS

Specimen: Surgical Excision

Block Number: B4

Interpretation: POSITIVE

Intensity: 3+

% Tumor Staining: 90%

Fish Ordered: No

METHODOLOGY:

Tissue was fixed in 10% neutral buffered formalin for no less than 8 and no longer than 24 hours. Her2 analysis was performed using the FDA approved HercepTest (TM) test kit using rabbit anti-human HER2. This assay was not modified. External kit-slides provided by the manufacturer (cell lines with high, low and negative HER2 protein expression) and in-house known HER2 amplified control tissue were evaluated along with the test tissue. Adequate, well preserved, clear-cut invasive carcinoma was identified for HER2 evaluation. Interpretation of the HER2 immunohistochemical stain is guided by published results in the medical literature, information provided by the reagent manufacturer and by internal review of staining performance.

This assay has been validated according to the 2007 joint recommendations and guidelines from ASCO and CAP and from the NCCN HER2 testing in Breast Cancer Task Force. Pathology Department takes full responsibility for this test's performance.

Gross Dictation: Pathologist,
Microscopic/Diagnostic Dictation: Patholoaist.
Final Review: Patholoaist.
Final: Pathologist,
Addendum: Pathologist,
Addendum Final: Pathologis'

Criteria	Yes	No
Diagnosis Discrepancy		
Primary Tumor Site Discrepancy		
HIPAA Discrepancy		
Prior Malignancy History		
Dual/Synchronous Primary Noted		
Case is (circle): QUALIFIED / DISQUALIFIED		
Reviewer Initials ___ Date Reviewed: ___		

TSS: [illegible]

ICD-O-3
Carcinoma, infiltrating ductal, NOS 8500/3
Site: breast, NOS C50.9 2/15/11 [illegible]

SPECIMENS:

A. SENTINEL LYMPH NODE BX #1 RIGHT AXILLA
B. SENTINEL LYMPH NODE BX #2 RIGHT AXILLA
C. WIDE EXCISION RIGHT BREAST
D. RIGHT BREAST CYST
E. RE-EXCISION INFERIOR LATERAL MARGIN-RIGHT BREAST

DIAGNOSIS:

A. LYMPH NODE, SENTINEL #1, RIGHT AXILLA, EXCISION:
ONE LYMPH NODE, NEGATIVE FOR TUMOR (0/1).
B. LYMPH NODE, SENTINEL #2, RIGHT AXILLA, EXCISION:
ONE LYMPH NODE, NEGATIVE FOR TUMOR (0/1).
C. BREAST, RIGHT, WIDE EXCISION:
-INVASIVE POORLY DIFFERENTIATED DUCTAL CARCINOMA WITH LOBULAR EXTENSION OF RIGHT BREAST (SBR GRADE 3)
-SIZE OF TUMOR: 1.5x1.5x1.2cm
-MARGINS OF RESECTION: FREE OF TUMOR
-BLUNT DUCT ADENOSIS AND FOCAL SCLEROSING ADENOSIS
-FOCAL DUCT ECTASIA
D. RIGHT BREAST CYST, EXCISION:
-CONISITENT WITH RUPTURED APOCRINE RETENTION CYST WITH REACTIVE ATYPIA; AND PERIDUCTAL FIBROSIS WITH GRANULATION TISSUE
-CYSTIC AND PAPILLARY APOCRINE CHANGE
-FOCAL BLUNT DUCT ADENOSIS
-FOCAL PERIDUCTAL DUCT ECTASIA (NEGATIVE FOR TUMOR)
E. RIGHT BREAST, RE-EXCISION-INFERIOR LATERAL MARGIN:
-DUCTAL CARCINOMA IN SITU WITH LOBULAR EXTENSION (SOLID PATTERN) ,HIGH NUCLEAR GRADE .see note
- STROMAL FIBROSIS ,FOCAL PERIDUCTAL MASTITIS
-CYSTIC APOCRINE CHANGE WITH MICRO AND COARSE CALCIFIACTION

Note: Slide#E1-represents section from new margin that shows extension of DCIS involving 3 lobular acini. There is no stromal invasion.
Slide#E6-focus of DCIS measures 8x5 mm, and in#E4-6x4 mm.

Invasive Breast Cancer Template

UUID:DAB174BB-862D-49BE-9BA4-9C11C4489305
TCGA-E2-A1LH-01A-PR Redacted

INVASIVE TUMOR:
Histologic type: ductal
Tumor Size (cm): 1.5x1.5x1.2cm
Size of Invasive Focus: 1.5x1.5x1.2cm
Grade, Histologic: 3
Grade, Nuclear: 3
Mitoses (Olympus 40x): 3
Scarff Bloom Richardson grade: III
Necrosis: absent
Invasion Vasc/Lymphatic: absent
DCIS component
DCIS Quantity: <25%
DCIS Type: solid
DCIS Location: inside and outside main mass
Nuclear grade: high
Necrosis: present
Margins: see note

Lymph nodes: negative (0/2) sentinel lymph nodes

Stage, Pathology : pT1c

TSS:

Non-neoplastic areas:

Hormone receptor status (by IHC): ER: pending
PR: pending

HERCEPTEST (by IHC): pending

Pathologist
Electronically signed

ADDENDUM

The ER/PR/HER2 status of the invasive breast carcinoma was determined by immunohistochemistry and quantitated via ACIS (image analysis). Results are as follows:

ER 0%

PR 0%

HER2 0.0%

A separate ACIS report has been generated.

NOTE: FISH analysis for HER2 gene amplification has not been ordered.

SPECIMEN(S):
A. SENTINEL LYMPH NODE BX #1 RIGHT AXILLA B. SENTINEL LYMPH NODE BX #2 RIGHT AXILLA C. WIDE EXCISION RIGHT BREAST D. RIGHT BREAST CYST E. RE-EXCISION INFERIOR LATERAL MARGIN-RIGHT BREAST

CLINICAL HISTORY:
Right breast ca.

FROZEN SECTION DIAGNOSIS:
A. SENTINEL LYMPH NODE #1 RIGHT AXILLA
Touch prep: No tumor seen on touch prep
B. SENTINEL LYMPH NODE BIOPSY #2 RIGHT AXILLA
Touch prep: No tumor seen on touch prep, reported to Dr. by Dr. a
C. WIDE EXCISION RIGHT BREAST
Gross only: Tumor about 0.8cm. from anterior margin, reported to Dr. by Dr.

GROSS DESCRIPTION:
A. SENTINEL LYMPH NODE BIOPSY #1 RIGHT AXILLA
Received fresh in a single container labelled and designated "sentinel lymph node bx #1 right axilla" and consists of a single 1.5x1.1x0.4cm. lymph node with tan cut surfaces and associated unremarkable adipose tissue. A touch preparation is made from the lymph node. The entire specimen is submitted in a single cassette labelled A1.
B. SENTINEL LYMPH NODE BIOPSY #2 RIGHT AXILLA
Received fresh in a single container labelled and designated "sentinel lymph node bx #2 right axilla" and consists of a single 1.3x1.3x0.4cm. lymph node with tan cut surfaces and associated unremarkable adipose tissue. Touch preparations are made from the lymph node. The entire specimen is submitted in a single cassette labelled B1.
C. WIDE EXCISION RIGHT BREAST
Received fresh in a single container labelled and designated "wide excision right breast cancer with needle localization" and consists of a single 8.5x7.5x3.0cm. resected portion of breast tissue. A single stitch of suture indicates the anterior aspect and a double stitch indicates the lateral aspect. A

localization wire is present within the specimen. A radiograph is also received with the specimen and shows a radiographic density in the region of the tip of the wire. The margins of resection is inked as follows: inferior orange, superior red, lateral yellow, anterior blue, medial green, posterior black. The specimen is serially sectioned from superior to inferior and a 1.5x1.5x1.2cm. tan grey well circumscribed tumor is identified. The tumor does not approach any of the margins grossly. However, it is within approximately 0.8cm. of the anterior (blue) margin. The tumor is at least 1.5cm. away from all the other margins. A small portion of tumor is submitted for tissue procurement as well as a portion of uninvolved breast parenchyma. The remainder of the cut surfaces are remarkable only for multiple small cystic nodules all 0.3cm. in diameter or less. Multiple sections including approximately 95% of the tumor are submitted and labelled as follows:.

Code of sections:

C1-C2: tumor approaching anterior margin
C3: tumor
C4-C5: tissue adjacent to medial margin
C6-C9: breast with cystic nodules

D. RIGHT BREAST CYST,excision

Received in formalin in a single container labelled and designated "right breast cyst" and consists of a single portion of firm tan yellow fibrofatty tissue measuring 3.7x2.7x2.2cm. No orientation is given. The resection margin is inked. The specimen is serially sectioned and located centrally is a 1.2cm. diameter cyst. The inner lining is smooth and tan. The remainder of the cut surfaces are composed of unremarkable adipose tissue with streaks of breast parenchyma. All of the cyst is submitted in cassettes D1 through D6.

E. RE-EXCISION INFERIOR LATERAL MARGIN RIGHT BREAST

Received in formalin in a single container labelled and designated "re-excision inferior lateral margin right breast" and consists of a portion of tissue measuring 2.0x1.7x1.5cm. A short stitch of suture indicates the inferior aspect of the specimen and a long stitch indicates the lateral aspect which is the new margin. The new margin submitted in a single cassette labelled E1 en face. The remainder of the specimen is serially sectioned and the cut surfaces show unremarkable adipose tissue and breast parenchyma.Remainder of specimen submitted and labelled E2-E9.

Gross Dictation: Pathology Fellow
Microscopic/Diagnostic Dictation: Pathologist, 1
Final Review: Pathologis
Final: Pathologist,
Addendum: Pathologist,
Addendum Review: Pathologist,
Addendum Final: Pathologis

Criteria	Yes	No
Diagnosis Discrepancy		✓
Primary Tumor Site Discrepancy		✓
HIPAA Discrepancy		✓
Prior Malignancy History		✓
Dual/Synchronous Primary Noted		✓
Case is (circle): QUALIFIED / DISQUALIFIED		
Reviewer Initials [illegible] Date Reviewed: [illegible]		

TSS

ICD-O-3
Carcinoma, infiltrating ductal, NOS 8500/3
Site: breast, NOS C50.9 2/15/11

SPECIMENS:
A. SENTINEL LYMPH NODE LEFT AXILLA
B. SENTINEL LYMPH NODE #2 LEFT AXILLA
C. WIDE LOCAL EXCISION LEFT BREAST

DIAGNOSIS:
A. SENTINEL LYMPH NODE #1, LEFT AXILLA:
-ONE LYMPH NODE, NO TUMOR SEEN (0/1).
B. SENTINEL LYMPH NODE #2, LEFT AXILLA:
-ONE LYMPH NODE, NO TUMOR SEEN (0/1).
C. WIDE LOCAL EXCISION, LEFT BREAST:
-INVASIVE AND IN-SITU DUCTAL CARCINOMA, SBR GRADE III/III WITH EXTENSIVE NECROSIS AND CANCERIZATION OF LOBULES, SEE TEMPLATE

Invasive Breast Cancer Template

UUID:B3A319BE-2230-43E2-91E5-A3B3C76B85BA
TCGA-E2-A1LI-01A-PR Redacted

INVASIVE TUMOR:
Histologic type: ductal
Tumor Size (cm): 2.5 cm
Size of Invasive Focus: 2.5 cm
Grade, Histologic: 3
Grade, Nuclear: 3
Mitoses 3
Scarff Bloom Richardson grade: III
Necrosis: present
Invasion Vasc/Lymphatic: absent

DCIS component
DCIS Quantity: <25%
DCIS Type: Clining
DCIS Location: outside main mass: diffuse
Nuclear grade: high
Necrosis: absent

Margins: DCIS present within 1 hpf of lateral and 1 mm of deep and medial margins

Lymph nodes: Negative (0/2)

Stage, Pathology : T2N1

Hormone receptor status (by IHC): ER & PR: PENDING
HERCEPTEST (by IHC): Pending

Pathologist
Electronically signed

ADDENDUM

Case #
Patient:

The ER/PR/HER2 status of the invasive breast carcinoma was determined by immunohistochemistry and quantitated via ACIS (image analysis). Results are as follows:

ER 0%

TSS:

PR 0%

HER2 2.3 (borderline)

A separate ACIS report has been generated.

NOTE: FISH analysis for HER2 gene amplification has been ordered.

Pathologist
Electronically signed

Addendum

Fluorescence in situ hybridization (FISH) for Her-2 gene amplification.
Her-2/neu gene: Not amplified
Ratio of Her-2/neu to CEP17: 1.21

The assay is performed on formalin fixed, paraffin embedded section using Her-2 DNA probe kit from Vysis. The quality of the fluorescence signals and tissue section is adequate for analysis. The signals of both Her-2 and CEP17 are counted in 60 invasive tumor nuclei. The Her-2 gene is considered amplified when the ratio of Her-2 to CEP17 signal is >2.0.

SPECIMEN(S):
A. SENTINEL LYMPH NODE LEFT AXILLA B. SENTINEL LYMPH NODE #2 LEFT AXILLA C. WIDE LOCAL EXCISION LEFT BREAST

CLINICAL HISTORY:
None

FROZEN SECTION DIAGNOSIS:
A. SENTINEL LYMPH NODE LEFT AXILLA
Touch prep: No tumor seen by Dr.
B. SENTINEL LYMPH NODE #2 LEFT AXILLA
Touch prep: No tumor seen by Dr.

GROSS DESCRIPTION:
A. SENTINEL LYMPH NODE LEFT AXILLA
Received fresh is a 1x0.8x0.5cm. lymph node. It is bisected and touch prep done. Specimen entirely submitted for permanent section.
B. SENTINEL LYMPH NODE #2 LEFT AXILLA
Received fresh is sentinel lymph node left axilla measuring 0.7x0.5x0.4cm. It is bisected and touch prep done. Submitted in toto in cassette B for permanent section.
C. WIDE LOCAL EXCISION LEFT BREAST
Received fresh is an oriented portion of breast tissue measuring 6x5.5x2.7cm. Sectioning shows a well circumscribed tan nodular tumor mass measuring 2.5x1.8x1cm. It closely approaches the superior posterior medial margin. Specimen is inked as follows: superior red, inferior orange, medial green, lateral yellow, posterior black. Tumor tissue and normal tissue given for tissue procurement and tumor sent for flow cytometry. Representative sections submitted in seventeen cassettes.

Gross Dictation: Pathology Fellow,
Microscopic/Diagnostic Dictation: Pathologist.
Final Review: Pathologist.
Final: Pathologist
Addendum: Pathologist
Addendum Review: Pathologist
Addendum Final: Pathologist

TSS:

Addendum: Pathologist, [illegible]
Addendum Review: Pathologist, [illegible]
Addendum Final: Pathologis[illegible]

Criteria	Yes	No
Diagnosis Discrepancy		✓
Primary Tumor Site Discrepancy		✓
HIPAA Discrepancy		✓
Prior Malignancy History		✓
Dual/Synchronous Primary Noted		✓
Case is (circle): QUALIFIED / DISQUALIFIED		
Reviewer Initials [illegible] Date Reviewed: [illegible]		

TSS: -

ICD-0-3
Carcinoma, infiltrating ductal, NOS 8500/3
Path Site: breast, upper outer quadrant C50.4
CQCF Site: breast, NOS C50.9
2/15/11

SPECIMENS:
A. LEFT MODIFIED RADICAL MASTECTOMY
B. LEFT AXILLARY CONTENTS LEVELS 1 AND 2

SPECIMEN(S):
A. LEFT MODIFIED RADICAL MASTECTOMY
B. LEFT AXILLARY CONTENTS LEVELS 1 AND 2

INTRAOPERATIVE CONSULTATION DIAGNOSIS:
Gross Exam A Left breast, modified radical mastectomy: Tumor is grossly 0.1 cm for posterior margin and >0.5 cm from other margins.
By Dr., called to Dr. at

GROSS DESCRIPTION:
A. LEFT MODIFIED RADICAL MASTECTOMY
Received fresh labeled with the patient name designated "left modified radical mastectomy", is a resected mastectomy specimen weighing 203 grams and measuring 19.6 x 7.5 x 5.8 cm. Specimen is received with orientation, a suture indicating the axillary tail. The ellipse of beige-tan skin measures 15.8 x 7.2 cm. The light brown areola measures 2.5 cm in diameter. The everted nipple measures 1.3 cm in diameter. The surface of the skin shows two firm masses, the larger measuring 2.5 x 2, the smaller measuring 0.6 x 0.6 cm. A large mass is palpated beneath the two lesions. The deep posterior margin is inked black, the anterior is inked orange. The specimen is serially sectioned from lateral to medial. Cut section shows a beige-tan firm, slightly necrotic mass which demonstrates a thick amber colored mucoid fluid. The mass lies just beneath the deep margin at a distance of 0.1 cm. The mass occupies the upper outer quadrant into the axillary tail. The mass measures 6.8 x 6.5 x 3.5 cm. The remainder of the specimen shows dark yellow lobulated adipose tissue with focal areas of white firm, fibrous parenchyma. A portion of the specimen is submitted for tissue procurement. Gross photographs are taken. Representative sections are submitted as follows:
A1-A2: sections of lesion and closest margin, posterior, and skin
A3-A4: one full thickness section from skin to deep margin with lesion
A5-A11: sections of lesion from upper-outer quadrant through mid portion
A12-A13: sections of lower-outer quadrant
A14-A15: representative sections lower-inner quadrant
A16-A17: representative sections upper-inner quadrant
A18-A19: sections of nipple
A20-A24: axillary lymph nodes, 2 sections from each

B. LEFT AXILLARY CONTENTS LEVELS 1&2
Received fresh are multiple tan-pink fragments of fibrofatty tissue aggregating to 6.0 x 3.0 x 2.0 cm. Dissection reveals 13 possible lymph nodes ranging from 0.1 x 0.1 x 0.1 cm to 3.5 x 2.5 x 2.0 cm.
Section code:
B1: Three possible lymph nodes
B2: Four possible lymph nodes
B3: Two possible lymph nodes
B4: Two possible lymph nodes
B5: One lymph node serially sectioned
B6-B10: Largest lymph node serially sectioned

UUID:6CC4E6EF-6664-448E-933E-FFA9117C7ABF
TCGA-E2-A1LK-01A-PR Redacted

DIAGNOSIS:
A. BREAST, LEFT, MODIFIED RADICAL MASTECTOMY:
- MULTIFOCAL, INVASIVE, DUCTAL CARCINOMA, SBR GRADE 3 WITH NECROSIS, LARGEST FOCUS MEASURES 6.8-CM, INVOLVING LATERAL HALF OF THE BREAST WITH EXTENSION TO SUPERFICIAL DERMIS
- SURGICAL RESECTION MARGINS ARE NEGATIVE FOR CARCINOMA
- LYMPHOVASCULAR AND PERINEURAL INVASIONS IDENTIFIED
- METASTATIC CARCINOMA TO TWO OF THREE LYMPH NODES (2/3), WITH EXTRANODAL EXTENSION, LARGEST MEASURING 2.8-CM
- SEE SYNOPTIC REPORT.

B. LYMPH NODES, LEFT AXILLARY CONTENTS, LEVELS 1 AND 2, RESECTION:
- METASTATIC CARCINOMA TO NINE OF TWELVE LYMPH NODES (9/12), WITH EXTRANODAL EXTENSION, LARGEST MEASURING 3.5-CM.

TSS:

SYNOPTIC REPORT - BREAST
Specimen Type: Mastectomy
Needle Localization: No
Laterality: Left
Invasive tumor: Present
Multifocality: Yes
WHO CLASSIFICATION
Invasive ductal carcinoma, NOS 8500/3
Tumor size: 6.8cm
Tumor site: Upper outer quadrant
Lower outer quadrant
Margins: Negative
Distance from closest margin: 0.4cm
deep
Tubular score: 3
Nuclear grade: 3
Mitotic score: 3
Modified Scarff Bloom Richardson Grade: 3
Necrosis: Present
Vascular/Lymphatic Invasion: Present
Extent: focal
Lobular neoplasia: None
Lymph nodes: Axillary dissection
Lymph node status: Positive 11 / 15 Extranodal extension
Non-neoplastic areas: fibroadenoma

DCIS not present

ER/PR/HER2 Results
Performed on Case:
ER: Negative
PR: Negative
HER2: Negative by FISH

Pathological staging (pTN): pT 3 N 3a

CLINICAL HISTORY:
year old female with large left breast IDC/ulcerating – now for modified radical mastectomy (left).

PRE-OPERATIVE DIAGNOSIS:
Left breast carcinoma.

Gross Dictation: Pathologist,
Microscopic/Diagnostic Dictation: Pathologist,
Final Review: Pathologist
Microscopic/Diagnostic Dictation: Pathologist,
Final Review:., Pathologist,
Final: Pathologist,

Criteria	Yes	No
Diagnosis Discrepancy		✓
Primary Tumor Site Discrepancy		✓
HIPAA Discrepancy		✓
Prior Malignancy History		✓
Dual/Synchronous Primary Noted		✓
Case is (circle): QUALIFIED / DISQUALIFIED		
Reviewer Initials [illegible] Date Reviewed: [illegible]		

ICD-O-3
Carcinoma, infiltrating duct 8500/3
Site (R) Breast NOS C50.9
3/10/14

TSS
Surg Date

SPECIMENS:
A. RIGHT AXILLARY CONTENTS
B. RIGHT BREAST WIDE EXCISION
C. RIGHT POSTERIOR MARGIN

UUID:E28607CD-0847-46F4-8E69-2D1DC80DE56F
TCGA-E2-A9RU-01A-PR Redacted

SPECIMEN(S):
A. RIGHT AXILLARY CONTENTS
B. RIGHT BREAST WIDE EXCISION
C. RIGHT POSTERIOR MARGIN

INTRAOPERATIVE CONSULTATION DIAGNOSIS:
A.-excision right breast: Mass/lesion identified 1 cm to the superior margin.
Diagnosis called by Dr. to Dr. at

DIAGNOSIS:
A.RIGHT AXILLARY CONTENTS:
-12 OF 22 LYMPH NODES ARE POSITIVE FOR METASTATIC CARCINOMA (12/22).
-FOCAL EXTRANODAL INVOLVEMENT IS IDENTIFIED.

B. RIGHT BREAST WIDE EXCISION:
- INVASIVE DUCTAL CARCINOMA (IDC), SBR GRADE 3, MEASURING 3.5 CM
- DUCTAL CARCINOMA IN -SITU, INTERMEDIATE NUCLEAR GRADE, SOLID AND CRIBRIFORM TYPES (MINOR COMPONENT).
- SURGICAL RESECTION MARGINS ARE NEGATIVE FOR TUMOR
- BIOPSY SITE CHANGES WITH FIBROSIS, GRANULATION TISSUE, FOREIGN BODY GIANT CELL REACTION AND FAT NECROSIS
- SEE SYNOPTIC REPORT AND SEE NOTE.

C. RIGHT POSTERIOR MARGIN:
- MAINLY ADIPOSE TISSUE WITH CALCIFICATION OF VESSEL WALL (MONCKEBERGS CALCIFIC SCLEROSIS).
- NO TUMOR IS IDENTIFIED.

SYNOPTIC REPORT - BREAST
Specimens Involved
Specimens: A: RIGHT AXILLARY CONTENTS
B: RIGHT BREAST WIDE EXCISION

Specimen Type: Excision
Localization: Needle
Laterality: Right
Multifocality: No
WHO CLASSIFICATION
Invasive carcinoma of no special type (NST) 8500/3
Tumor size: 35mm
Additional dimensions: 30mm x 30mm
Tumor Site: Not specified
Margins: Negative
distance from (in mm):anterior margin: 6
Tubular Score: 3
Nuclear Grade: 3
Mitotic Score: 3
Modified Scarff Bloom Richardson Grade: 3
Necrosis: Present
Vascular/Lymphatic Invasion: Present
Lymph nodes: Non-sentinel lymph node
Lymph node status: Positive 12 / 22
Size of largest metastasis: 20mm

TSS
Surg Date

Micrometasteses: Yes
Extranodal extension: Yes

DCIS PRESENT
Yes
Margins: Margins uninvolved by DCIS Specify: 1.5 mm to superior resection margin
DCIS Quantity: Estimate % 2
DCIS Type: Solid
Cribriform
DCIS Location: Separate from invasive tumor mass
Nuclear Grade: Intermediate
Necrosis: Absent
ER/PR/HER2 Results
Estrogen Receptor: Positive
Allred Score: 6
Progesterone Receptor: Negative
Allred Score: 0
Her2: Interpretation: Negative
Methodology: FISH
Performed on Case: (outside slides0

Pathological staging (pTN): pT 2 N 3a
Pathological staging is based on the AJCC Cancer Staging Manual, 7th Edition

GROSS DESCRIPTION:
A. RIGHT AXILLARY CONTENTS
Received fresh labeled with the patient's identification and "right axillary contents " are piece of yellow-tan adipose tissue, 14 x 10 x 2 cm, containing lymph nodes ranging from 0.2 cm to 2 cm; lymph nodes are sectioned and submitted entirely:
A1: 6 possible lymph nodes
A2: 2 lymph nodes, bisected (one inked green)
A3-A5: 1 lymph node, bisected, each block
A6: 1 lymph node
A7: 2 lymph nodes, bisected (one inked green)
A8: 1 lymph node, bisected
A9:2 lymph nodes
A10: 1 lymph node
A11: 3 lymph nodes
A12-13:1 lymph node
A14-A17: 1 lymph node
A18-A19: 1 lymph node

B. RIGHT BREAST EXCISION
Received fresh labeled with the patient's identification and "right breast excision" is an previously inked, 177 g, 11.5 cm medial to lateral x 7.5 cm superior to inferior x 3.5 cm anterior to posterior, excision. The overlying skin measuring 9.5x 7 cm in diameter. There are multiple brown papules on the skin. Ink code: anterior-yellow, posterior-black, medial-green, lateral-red, superior-blue, inferior-orange. The specimen is serially sectioned from medial to lateral into 7 slices revealing a 3.5 x 3 x 3 cm, white, firm mass that is closest to the superior and anterior margins at 1 cm. The clip was retrieved in slice 4. Representatively submitted as per the attached diagram:
B1: medial margin
B2: slice 2, anterior margin with tumor
B3: slice 2, superior margin with tumor
B4: slice 3, skin with tumor
B5-B6: slice 3, anterior margin with tumor
B7: slice 3, superior margin with tumor
B8: slice 3, posterior margin with tumor
B9: slice 3, inferior margin

TSS
Surg Date

B10: slice 4, tumor around clip
B11: slice 4, skin with tumor
B12: Slice 5, superior and anterior margins
B13: slice 7, lateral margin

C. RIGHT POSTERIOR MARGIN
Received fresh labeled with the patient's identification and "right posterior margin" is a yellow-tan adipose tissue, 10 x 2 x 1.3 cm. One surface has been marked with clips indicating new margin. This surface is inked black. Serial sectioning does not reveal any abnormality. Representative sections submitted in 4 cassettes.

CLINICAL HISTORY:
None Given

PRE-OPERATIVE DIAGNOSIS:
None Given

Gross Dictation:
Final Review:
Final: M.D.,

Criteria 1/7/14	Yes	No
Diagnosis Discrepancy		
Primary Tumor Site Discrepancy		
HIPAA Discrepancy		
Prior Malignancy History		
Dual/Synchronous Primary Noted		
Case is (circle): QUALIFIED / DISQUALIFIED		
Reviewer Initials Date Reviewed: / /		

ICD-0-3
Carcinoma, infiltrating duct, NOS 8500/3
Path Site Code: breast, upper outer quadrant C50.4
CQCF Site: breast, NOS C50.9
12/19/10

TSS Pt Id:

UUID:AA1BACC4-0B3D-4E2E-865E-24B570DA4E74
TCGA-E2-A10A-01A-PR Redacted

SPECIMENS:
A. SENTINEL LYMPH NODE #1 LEFT AXILLA
B. SENTINEL LYMPH NODE #2 LEFT AXILLA
C. SENTINEL LYMPH NODE #3 LEFT AXILLA
D. LEFT BREAST
E. RIGHT BREAST SKIN

SPECIMEN(S):
A. SENTINEL LYMPH NODE #1 LEFT AXILLA
B. SENTINEL LYMPH NODE #2 LEFT AXILLA
C. SENTINEL LYMPH NODE #3 LEFT AXILLA
D. LEFT BREAST
E. RIGHT BREAST SKIN

INTRAOPERATIVE CONSULTATION DIAGNOSIS:
TP A-C: Negative for tumor. By Dr., called to Dr.

GROSS DESCRIPTION:
A. SENTINEL LYMPH NODE #1 LEFT AXILLA
Received fresh labeled with patient name designated "A – sentinel lymph node #1 left axilla" is a fragment of beige-tan possible lymphoid tissue measuring 1.1 x 0.9 x 0.3 cm. The specimen is serially sectioned. Touch preps were performed. The entire specimen is submitted in cassette A1.
B. SENTINEL LYMPH NODE #2 LEFT AXILLA
Received fresh labeled with patient name designated "B – sentinel lymph node #2" is a fragment of yellow-red fibroadipose tissue measuring 3.2 x 1.5 x 0.7 cm. One possible lymph node is identified measuring 1.2 x 0.6 x 0.5 cm. The specimen is bisected. Touch preps were performed. The entire lymph node is submitted in cassette B1.
C. SENTINEL LYMPH NODE#3 LEFT AXILLA
Received fresh labeled with patient name designated "C – sentinel lymph node #3 left axilla" is a fragment of beige-tan possible lymphoid tissue measuring 1.2 x 0.6 x 0.4 cm. The specimen is serially sectioned, touch preps were performed. The entire specimen is submitted in cassettes C1.
D. LEFT BREAST
Received fresh labeled with patient name designated "D – left breast" is a resected mastectomy specimen weighing 676 grams and measuring 22.5 x 19 x 3 cm. The specimen is received with orientation. A suture designates the axillary end of breast. The deep margin is inked black. The overlying beige-tan ellipse of skin measures 14.5 x 4.2 cm. The light brown areola measures 3.5 cm in diameter. The everted nipple measures 1.1 cm in diameter. The specimen is serially sectioned from medial to lateral. Cut section shows a firm beige-tan mass in the upper outer quadrant approaching the deep surgical margin at closest distance 1.3 cm and is located 5 cm from the axillary tail. The lesion measures 3.6 x 3 x 2.2 cm. This lesion extends to the lower outer quadrant for about 2.5 x 1.5 cm. A second possible lesion is noted in the upper outer quadrant superior to the first main mass approaching the deep surgical margin at a distance of 2.5 cm. This area is located 5.2 cm from the first lesion and measures 0.5 x 0.5 x 0.4 cm. A third possible subareolar is noted located 4.5 cm from the first main mass in the upper outer quadrant. This third lesion measures 0.6 x 0.5 x 0.5 cm. A fourth possible mass is located approximately 2.5 cm from the main mass in the upper outer quadrant and approaches the deep margin at a distance of 2.2 cm. The fourth lesion measures 0.5 x 0.3 x 0.3 cm. The remainder of the breast parenchyma shows multiple patchy fibrous firm tissue. A portion of the specimen is submitted for tissue procurement. Representative sections are submitted as follows:
D1-D3: the main mass in the upper outer quadrant with overlying deep margin
D4-D5: remainder of the main mass in the upper outer quadrant
D6: lesion #2 upper outer quadrant
D7: lesion #3 subareolar
D8: lesion #4 upper outer quadrant
D9-D10: additional firm fibrous tissue adjacent to main mass of lower outer quadrant
D11-D14: additional section lower outer quadrant
D15-D16: fibrous tissue central subareolar
D17-D18: representative sections upper inner quadrant
D19-D21: representative sections lower inner quadrant
D22: section of nipple
D23: representative sections of skin

HX

D24-D28: multiple possible axillary lymph nodes

E. RIGHT BREAST TISSUE SKIN
Received in formalin in a container labeled with the patient name designated "e. right breast skin" is an irregular fragment of beige-tan skin measuring 11.4 x 4.5 x 0.3 cm. The surface of the specimen is unremarkable. A section shows unremarkable skin tissue. Representative sections are submitted in cassettes E1-E3.

DIAGNOSIS:

A. SENTINEL LYMPH NODE #1, LEFT AXILLA, EXCISION:
- ONE LYMPH NODE, NEGATIVE FOR TUMOR (0/1).

B. SENTINEL LYMPH NODE #2, LEFT AXILLA, EXCISION:
- ONE LYMPH NODE, NEGATIVE FOR TUMOR (0/1).

C. SENTINEL LYMPH NODE #3, LEFT AXILLA, EXCISION:
- ONE LYMPH NODE, NEGATIVE FOR TUMOR (0/1).

D. LEFT BREAST, MASTECTOMY:
- INVASIVE DUCTAL CARCINOMA, MULTIFOCI, SBR GRADE II.
- SIZE OF LARGEST TUMOR FOCUS MEASURING 6.1 X 4.5 CM.
- DUCTAL CARCINOMA IN-SITU, CRIBRIFORM AND MICROPAPILLARY TYPES.
- ATTACHED SKIN AND NIPPLE, NEGATIVE FOR TUMOR.
- SURGICAL RESECTION MARGINS, NEGATIVE FOR TUMOR.
- SEE TEMPLATE.

SYNOPTIC REPORT - BREAST
Specimens Involved
Specimens: D: LEFT BREAST

Specimen Type: Mastectomy
Needle Localization: No
Laterality: Left
Invasive tumor: Present
Multifocality: Yes
WHO CLASSIFICATION
Invasive ductal carcinoma, NOS 8500/3
Specimen size: Size of Invasive focus 6.1cm
Additional dimensions: 4.5cm x 2.2cm
Tumor Site: Upper outer quadrant
Lower outer quadrant
Margins: Negative
Distance from closest margin: 1.3cm
Margin: deep
Tubular score: 2 (10-75% tubule)
Nuclear grade: 2
Mitotic score (Olympus 40x): 2 (7-13/10)
Modified Scarff Bloom Richardson Grade: II (6-7 points)
Necrosis: Present
Vascular/Lymphatic Invasion: Indeterminate
Lobular neoplasia: None
Lymph nodes: Sentinel lymph node only
Lymph node status: Negative 0 / 3
Non-neoplastic areas: Fibrocystic disease, pseudoangiomatous stromal hyperplasia
DCIS present
DCIS Quantity: Estimate % 10
DCIS type: Cribriform
Micropapillary
DCIS location: Associated with invasive tumor
Nuclear grade: Intermediate
Necrosis: Absent
Location of CA++: Benign epithelium
Pathological staging (pTN): pT 3 N 0
Comment(s): See breast biomarker template

SYNOPTIC REPORT - BREAST, ER/PR RESULTS
Specimens Involved
Specimens: D: LEFT BREAST

SPECIMEN:
Other
mastectomy
Block Number: D4
ER: Positive - Allred Score: 7 = Proportion score: 4 + Intensity Score 3
PR: Positive - Allred Score: 8 = Proportion Score 5 + Intensity Score 3
COMMENT:
The Allred score for estrogen and progesterone receptors is calculated by adding the sum of the proportion score (0 = no staining, 1 = <1% of cells staining, 2 = 1 - 10% of cells staining, 3 = 11-30% of cells staining, 4 = 31-60% of cells staining, 5 = >60% of cells staining) to the intensity score (1 = weak intensity of staining, 2 = intermediate intensity of staining, 3 = strong intensity of staining), with a scoring range from 0 to 8.
ER/PR positive is defined as an Allred score of >2 and ER/PR negative is defined as an Allred score of less than or equal to 2.
Methodology: Fixation Type and Length: Tissue was fixed in 10% neutral buffered formalin) for no less than 8 and no longer than 24 hours. Antibody and Assay Methodology: Mouse anti-human ER and PR,
Comment: This assay can be used to select invasive breast cancer patients for hormone therapy (1). ER and PR analysis was performed on this case by immunohistochemistry utilizing the ER (ER 1D5, 1:100) and PR (PGR 136, 1:100) antibody provided by following the manufacturer's instructions listed in the package insert. This assay was not modified, and adherence to all instruction and guidelines were strictly followed. Interpretation of the ER/PR immunohistochemical staining characteristics is guided by published results in the medical literature (1), information provided by the reagent manufacturer and by internal review of staining performance within the Pathology Department.
1. Harvey JM, et al. Estrogen receptor status by immunohistochemistry is superior to the ligand-binding assay for predicting response to adjuvant endocrine therapy in breast cancer. J Clin Oncol. 17:1474-1481, 1999

CLINICAL HISTORY:
None given

PRE-OPERATIVE DIAGNOSIS:
Left breast ca

ADDENDUM:
E. RIGHT BREAST SKIN, EXCISION:
- FRAGMENT OF UNREMARKABLE SKIN, NEGATIVE FOR TUMOR.

PathVysion HER-2 DNA Probe Kit
Case No
Analytical Interpretation of Results: HER-2 NOT AMPLIFIED
Clinical Interpretation of results
Amplification of the HER-2 gene was evaluated with interphase fluorescence in-situ hybridization (FISH) on formalin-fixed paraffin embedded tissue sections using a chromosome 17 centromeric probe and a HER-2 probe that spans the entire HER-2 gene in the i. A majority of tumors cells displayed 2 chromosome 17 signals and 2 HER-2 signals, with a HER-2/CEP 17 Ratio </=2.0, consistent with no amplification of the HER2/neu gene.
Block used D4 Source of case: RPCI
Tissue fixation formalin-fixed tissue Outside Case No: NA
Tissue source breast Results interpreted: yes
HER2/CEP17 ratio: 1.21
This ratio is derived by dividing the total number of LSI HER-2/neu signals by the total number of CEP17 signals in at least 20 interphase nuclei with nonoverlapping nuclei in the neoplastic mammary epithelial cells. Cells with no signals or with signals of only one color are disregarded.

Method of ratio enumeration: manual count

Limitations

The Vysis PathVysion Kit is not intended for use to screen for or diagnose breast cancer. It is intended to be used as an adjunct to other prognostic factors currently used to predict disease-free and overall survival in stage II, node-positive breast cancer patients. In making decisions regarding adjuvant CAF treatment, all other available clinical information should also be taken into consideration, such as tumor size, number of involved lymph nodes, and steroid receptor status.

No

treatment decision for stage II, node-positive breast cancer patients should be based on HER-2/neu

gene amplification status alone.

Overview of this test

FDA APPROVED REAGENT

PathVysion HER-2 DNA Probe Kit is FDA approved for selection of patients for whom Herceptin® therapy is being considered. These tests were performed in the under the direction

of The results of these studies should always be interpreted in the context of the clinical, morphological, and immunophenotypic diagnosis.

Gross Dictation:,
Microscopic/Diagnostic Dictation:., Pathologist,
Microscopic/Diagnostic Dictation: Pathologist,
Microscopic/Diagnostic Dictation: Pathologist
Final Review: Pathologist,
Final: Pathologist,
Addendum: Pathologist,
Addendum Final: Pathologist,
Addendum: Pathologist,
Addendum Review: Pathologist,
Addendum Final:., Pathologist,
Addendum: Pathologist,
Addendum Final:., Pathologist,

Criteria	Yes	No
Diagnosis Discrepancy		✓
Primary Tumor Site Discrepancy		✓
HIPAA Discrepancy		✓
Prior Malignancy History		✓
Dual/Synchronous Primary Noted		✓
Case is (circle): QUALIFIED / DISQUALIFIED		
Reviewer Initials [illegible] Date Reviewed: [illegible]		

ICD-O-3
Carcinoma, infiltrating duct, NOS 8500/3
Path Site Code: breast, central C50.1
CQCF Site: breast, NOS C50.9
12/19/10

TSS Pt ID

UUID:CFB8EE09-41D3-4A39-A4AE-4CDEC84BB9BC
TCGA-E2-A10E-01A-PR Redacted

SPECIMENS:
A. WLE VULVA
B. SENTINEL LYMPH NODE #1
C. SENTINEL LYMPH NODE #2
D. SENTINEL LYMPH NODE #3
E. SENTINEL LYMPH NODE #4
F. SENTINEL LYMPH NODE #5
G. LEFT BREAST
H. LEFT AXILLARY CONTENTS

SPECIMEN(S):
A. WLE VULVA
B. SENTINEL LYMPH NODE #1
C. SENTINEL LYMPH NODE #2
D. SENTINEL LYMPH NODE #3
E. SENTINEL LYMPH NODE #4
F. SENTINEL LYMPH NODE #5
G. LEFT BREAST
H. LEFT AXILLARY CONTENTS

GROSS DESCRIPTION:
A. WLE VULVA
Received is a vulvectomy specimen measuring 4 x 1.7 x 1 cm. The surface of the specimen is tan-gray and unremarkable. The single right stitch is arbitrarily designated 12 o'clock. The specimen is inked as follows: 12 o'clock, 3 o'clock, 6 o'clock-blue, 6 o'clock, 9 o'clock, 12 o'clock-orange. The specimen is serially sectioned from right to left and submitted in toto as follows:
A1: 12 o'clock right margin
A2-A4: full thickness sections from right to left
A5: 6 o'clock left margin
B. SLN #1: Received fresh is a tan pink lymph nodes 1.0 x 0.9 x 0.5cm. The specimen is bisected, touch preps are taken and the specimen is submitted in toto in FSB.
C. SLN #2: Received fresh is a tan pink lymph nodes 0.5 x 0.3 x 0.3cm. The specimen is bisected, touch preps are taken and the specimen is submitted in toto in C1.
D. SLN #3: Received fresh is a tan pink lymph nodes 1.0 x 0.5 x 0.5cm. The specimen is bisected, touch preps are taken and the specimen is submitted in toto in D1.
E. SLN#4: Received fresh is a tan pink lymph nodes 1.5 x 1.3 x 0.5cm. The specimen is bisected, touch preps are taken and the specimen is submitted in toto in E1.
F. SLN#5: Received fresh is a tan pink lymph nodes 1.0 x 0.9 x 0.5cm. The specimen is bisected, touch preps are taken and the specimen is submitted in toto in F1.
G. LEFT BREAST:
Received fresh is a 474 gram simple mastectomy specimen measuring 21 x 19 x 3.5 cm. The specimen is partially surfaced with a tan-pink ellipse of skin measuring 16 x 16 cm. The skin surface is remarkable for a centrally located partially raised nipple 1 cm. The areola rim measures 1.3 cm. The specimen is inked as follows: superior anterior-blue, anterior inferior-orange, posterior-black. The specimen is serially sectioned from lateral to medial into 10 slices; slice serially sectioned from medial to lateral in 10 slices; slice 1 the most medial, slice 10 the most lateral. The nipple is located in slice 5 and 6. The cut surface reveals a gray-white firm well circumscribed mass measuring 2.5 x 1.5 x 1 cm, located in slice 5 and 6 and measuring 0.7 cm. from the closest deep margin. The mass is retroareolar measuring 2 cm. deep from the nipple. A second satellite nodule is identified in slice 8 measuring 1 x 0.9 x 0.8 cm, 0.4 cm. from the deep margin and 5.5 cm. from nodule #1. A third possible satellite nodule is grossly identified in slice 7 measuring 0.4 cm. in greatest dimension, greater than 0.1 cm. from the deep margin and 2.0 cm. from nodule #2 and 4 cm. from nodule #1. Nodule 2 and 3 are both located in the lower outer quadrant. Remaining cut surfaces reveal predominantly yellow lobulated adipose tissue inrterdispersed with gray-white fibrous tissue. A portion of the specimen is submitted for tissue procurement. Representative sections are submitted as follows:
G1: nipple serially sectioned slice 5
G2: nipple serially sectioned slice 6
G3: upper inner quadrant slice 3
G4: upper inner quadrant with deep margin slice 4
G5: lower inner quadrant slice 3

G6: lower inner quadrant slice 4
G7: area immediately adjacent to mass #1
G8; slice 4
G9: slice 5
G10: upper central slice 5
G11: lower central slice 5
G12: skin adjacent no nodule #1
G13-G14: nodule #1 with closest deep margin slice 6
G15: area above nodule #1 slice 6
G16: immediately adjacent to nodule #1 with deep margin slice 7
G17: nodule #3 with deep margin slice 7
G18: lower outer quadrant slice 7
G19: area immediately adjacent to nodule #2 with deep margin slice 7
G20: upper outer quadrant with deep margin slice 8
G21: inferior margin lower outer quadrant adjacent to nodule #2 slice 8
G22: nodule #2 with deep margin slice 8 lower outer quadrant
G23: area immediately adjacent to nodule #2 with inferior and deep margin slice 9

H. LEFT AXILLARY CONTENTS:
Received in formalin are multiple tan-pink fragments of fibrofatty tissue aggregating to 7 x 6 x 3 cm. Dissection reveals 15 possible lymph nodes ranging from 0.1 x 0.1 x 0.1 cm to 2 x 1.5 x 1 cm. Section code:
H1: Five possible lymph nodes
H2: Five possible lymph nodes
H3: Four possible lymph nodes
H4: One lymph node serially sectioned.

DIAGNOSIS:

A. VULVA, WIDE LOCAL EXCISION:
- MODERATE TO SEVERE SQUAMOUS DYSPLASIA (VIN II-III)
- MILD SQUAMOUS DYSPLASIA PRESENT AT 12 O'CLOCK TO 6 O'CLOCK MARGIN, SEE NOTE 1.

B. LYMPH NODE, SENTINEL #1, LEFT AXILLA, BIOPSY:
- METASTATIC CARCINOMA TO ONE OF ONE LYMPH NODE (1/1), MEASURING 3.5 MM WITH EXTRANODAL EXTENSION.

C. LYMPH NODE, SENTINEL #2, LEFT AXILLA, BIOPSY:
- ONE LYMPH NODE, NEGATIVE FOR METASTASES (0/1).

D. LYMPH NODE, SENTINEL #3, LEFT AXILLA, BIOPSY:
- ONE LYMPH NODE, NEGATIVE FOR METASTASES (0/1).

E. LYMPH NODE, SENTINEL #4, LEFT AXILLA, BIOPSY:
- METASTATIC CARCINOMA TO ONE OF ONE LYMPH NODE (1/1), MEASURING 1.2-CM WITH NO EXTRANODAL EXTENSION.

F. LYMPH NODE, SENTINEL #5, LEFT AXILLA, BIOPSY:
- ONE LYMPH NODE, NEGATIVE FOR METASTASES (0/1).

G. BREAST, LEFT, SIMPLE MASTECTOMY:
- THREE FOCI OF INVASIVE, DUCTAL CARCINOMA, SBR GRADE 1, LARGEST MEASURING 1.8-CM
- SURGICAL RESECTION MARGINS NEGATIVE FOR TUMOR
- TUMOR (LARGEST FOCUS) IS 2.5-MM FROM THE DEEP SURGICAL RESECTION MARGIN
- LOW NUCLEAR GRADE, DUCTAL CARCINOMA IN SITU, MICROPAPILLARY, CRIBRIFORM AND PAPILLARY TYPES
- PERINEURAL INVASION IDENTIFIED
- SEE SYNOPTIC REPORT AND SEE NOTE 2.

H. LYMPH NODES, LEFT AXILLARY CONTENTS, DISSECTION:

- SIXTEEN LYMPH NODES, NEGATIVE FOR METASTASES (0/16).

NOTE 1: Mild squamous dysplasia is present at the 12 o'clock-3 o'clock-6 o'clock margin in multiple levels. Focally, this margin has cautery artifact which precludes the assessment of degree of dysplasia.

NOTE 2: Three foci of invasive ductal carcinoma are identified; largest measuring 1.8-cm is located centrally. The other two foci measure 1.0-cm and 0.4-cm respectively. All surgical resection margins are free of tumor. The closest margin is posterior (2.5 mm from the largest tumor mass).

SYNOPTIC REPORT - BREAST
Specimen Type: Mastectomy
Needle Localization: No
Laterality: Left
Invasive Tumor: Present
Multifocality: Yes
WHO CLASSIFICATION
Invasive ductal carcinoma, NOS 8500/3
Tumor size: 1.8cm
Tumor Site: Central
Margins: Negative
Distance from closest margin: 0.25cm
deep
Tubular Score: 2
Nuclear Grade: 2
Mitotic Score: 1
Modified Scarff Bloom Richardson Grade: 1
Necrosis: Absent
Vascular/Lymphatic Invasion: None identified
Lobular neoplasia: None
Lymph nodes: Sentinel lymph node and axillary dissection
Lymph node status: Positive 2 / 21 Extranodal extension
Micrometastases: No

DCIS present
Margins uninvolved by DCIS
DCIS Quantity: Estimate 2%
DCIS Type: Cribriform
Micropapillary
Papillary
DCIS Location: Associated with invasive tumor
Nuclear grade: Low
Necrosis: Absent
Location of CA++: DCIS
Benign epithelium

ER/PR/HER2 Results
ER: Positive
PR: Positive
HER2: Negative by FISH
Performed on Case:

Pathological staging (pTN): pT 1c N 1a

CLINICAL HISTORY:
None given

PRE-OPERATIVE DIAGNOSIS:
Multifocal invasive ca. left breast

INTRAOPERATIVE CONSULTATION:
FSB/TPB: Positive for tumor cells. Diagnosis called to Dr. at by Dr
TPC/TPD/TPF: Negative for tumor cells. Diagnosis called to Dr at , by Dr.
TPE: Positive for tumor cells. Diagnosis called to Dr. at by Dr.

Gross Dictation:., Pathologist
Gross Dictation:
Microscopic/Diagnostic Dictation: Pathologist
Microscopic/Diagnostic Dictation: Pathologist,
Microscopic/Diagnostic Dictation:.. Pathologist
Final Review:., Pathologist,
Final: Pathologist,

Criteria	Yes	No
Diagnosis Discrepancy		✓
Primary Tumor Site Discrepancy		✓
HIPAA Discrepancy		✓
Prior Malignancy History		✓
Dual/Synchronous Primary Noted		✓
Case is (circle): QUALIFIED / DISQUALIFIED		
Reviewer Initials [illegible] Date Reviewed: [illegible]		

[illegible]

Carcinoma, infiltrating duct, NOS 8500/3

Path Site Code: breast, upper inner quadrant C50.2 12/29/10

CQCF Site: breast, NOS C50.9

UUID:C972EC08-E1AE-4FCB-B8A1-15000306CDE7
TCGA-E2-A14N-01A-PR Redacted

TSS:

SPECIMENS:
A. SENTINEL NODE #1 RIGHT AXILLA
B. RIGHT BREAST
C. AXILLARY CONTENT
D. LEFT BREAST REDUCTION

SPECIMEN(S):
A. SENTINEL NODE #1 RIGHT AXILLA
B. RIGHT BREAST
C. AXILLARY CONTENT
D. LEFT BREAST REDUCTION

INTRAOPERATIVE CONSULTATION DIAGNOSIS:
TPA, FSA: Sentinel lymph node #1 right axilla: Smears (touch imprint)-Negative for tumor cells, (frozen section)-Positive for carcinoma.
By Dr, called to Dr. at

GROSS DESCRIPTION:
A. SENTINEL LYMPH NODE #1, RIGHT AXILLA
Received fresh and labeled "sentinel node #1 right axilla" is a 2.8x1.0x1.2cm lymph node. There is blue dye staining present. The specimen is sectioned and a touch prep is performed. A portion of the lymph node is submitted for frozen section. The lymph node is submitted in toto as follows:
FSA1: frozen section of portion of lymph node
A2-A4: remainder of lymph node
B. RIGHT BREAST,MASTECTOMY:
Received is a 1,321gm right mastectomy specimen measuring 23x23x5.8cm. Margin of specimen oriented with a stich indicating the lateral margin . In the medial portion of the specimen is an ellipse of tan skin measuring 6.7cm in length with a diameter of 3.1cm. Eccentrically located on the skin is a healed scar measuring 5.5cm in length. The areola is present and measures 4.0cm in length with a width of 3.0cm. The nipple is everted and is 1.0cm. The axillary tail is 6.5x5.0x1.5cm. The anterior surface of the specimen is inked blue, the posterior surface is inked black and the specimen is serially sectioned. In the upper inner quadrant is a well circumscribed white-tan mass measuring 3.8x3.2x2.9cm which is located 0.5 cm from the nearest deep margin. The central portion of the mass shows areas of hemorrhage and possible necrosis. 4.6cm lateral from the mass, located approximately 2.0cm from the areola region, is a hemorrhagic white-tan firm mass measuring 2.5x2.0x1.8cm. It is located 2.5cm from the deep margin. The remainder of the specimen consists of primarily adipose tissue. A few possible lymph nodes are found within the axillary tail. Multiple sections are submitted and labelled as follows: follows:
B1-10:sections from the larger tumor (medial)
B11-14: sections from the smaller tumor near nipple area.
B15-16 – sections from upper inner quadrant
B17-18: sections from upper outer quadrant
B19-20: sections from lower outer quadrant.
B21-22: sections from lower inner quadrant
B23- 24 sections from nipple and areolar area.
B25-B26: possible lymph nodes
C. AXILLARY CONTENTS
Received in formalin and labeled "axillary contents levels 1&2" is a piece of adipose tissue, 7.3 x 5.6 x 0.9 cm. Multiple lymph nodes are found, ranging in size from 0.1 to 2.8cm. Lymph nodes are submitted in toto as follows:
C1: 5 possible lymph nodes
C2-C6: 4 possible lymph nodes, each
C7: 2 possible lymph nodes
C8: 3 possible lymph nodes
C9-C10: 1 bisected lymph node, each
C11-C13: 1 lymph node each
D. LEFT BREAST REDUCTION:
Received in formalin and labeled ", Left Breast Reduction mammoplasty". The specimen consists of primarily adipose tissue, little fibrous breast tissue is found. No masses or lesions are seen. Representative sections are submitted as follows: D1 skin and subjacent adipose tissue, D2-D3 fibrous tissue.

DIAGNOSIS:
A. SENTINEL NODE #1, RIGHT AXILLA:
- METASTATIC CARCINOMA TO ONE OUT OF ONE LYMPH NODE, CONSISTENT WITH METASTASIS FROM PRIMARY BREAST CARCINOMA.
(1/1) see note.

B. RIGHT BREAST, MASTECTOMY SPECIMEN:
- INVASIVE DUCTAL CARCINOMA, SBR GRADE II WITH GEOGRAPHIC AREAS OF NECROSIS, MULTICENTRIC.

- SIZE OF TUMOR: MEDIAL ASPECT OF BREAST-3.8 x 3.2 x 2.9 CM.
  - CENTRAL AREA - SIZE OF TUMOR-2.5 x 2.0 x 1.8 CM.
- MARGINS OF RESECTION-NEGATIVE FOR TUMOR.
- FOCAL COLUMNAR CELL CHANGE.
- TWO AXILLARY LYMPH NODES-NEGATIVE FOR TUMOR (0/2).

C. AXILLARY CONTENTS, RESECTION:
- THIRTY-FIVE AXILLARY LYMPH NODES-NEGATIVE FOR TUMOR (0/35).

D. LEFT BREAST REDUCTION:
- BREAST TISSUE WITH INCLUDED SKIN TISSUE-NO SPECIFIC PATHOLOGIC CHANGES-NEGATIVE FOR TUMOR.

NOTE: In specimen A,(A1) size of lymph node measured 2.8x1.0x1.2cm and metastatic tumor only seen on the portion of lymphnode submitted for frozen and permanent section in an area measuring 5.0x2.5mm. The remainder of the lymph node submitted as A2-A4 are negative for metastatic tumor.

SYNOPTIC REPORT - BREAST
Specimens Involved
Specimens: A: SENTINEL NODE #1 RIGHT AXILLA
B: RIGHT BREAST
C: AXILLARY CONTENT
D: LEFT BREAST REDUCTION

Specimen Type: Mastectomy
Needle Localization: No
Laterality: Right
Invasive tumor: Present
Multifocality: Yes
WHO CLASSIFICATION
Invasive ductal carcinoma, NOS 8500/3
Specimen size: Size of Invasive focus 3.8cm
Additional dimensions: 3.2cm x 2.9cm
Tumor Site: Upper inner quadrant
Central
Margins: Negative
Distance from closest margin: 0.5cm
Margin: deep
Tubular score: 3 (<10% tubule)
Nuclear grade: 3
Mitotic score (Olympus 40x): 3 (>13/10 hpf)
Modified Scarff Bloom Richardson Grade: III (8-9 points)
Necrosis: Present
Vascular/Lymphatic Invasion: None identified
Lobular neoplasia: None
Lymph nodes: Sentinel lymph node and axillary dissection
Lymph node status: Positive 1 / 38
Micrometastases: No
DCIS PRESENT?
No
Pathological staging (pTN): pT 2 N 1

CLINICAL HISTORY:
year old with right breast ca

PRE-OPERATIVE DIAGNOSIS:
Right breast ca

ADDENDUM:
BREAST ER/PR -1
SPECIMEN
Type: Other
Mastectomy
Block Number: B9
HORMONE RECEPTOR STATUS
Laboratory:
Estrogen Receptor: Negative
Allred Score: 0 = Proportion Score 0 + Intensity Score 0

Progesterone Receptor: Negative
Allred Score: 0 = Proportion Score 0 + Intensity Score 0
The Allred score for estrogen and progesterone receptors is calculated by adding the sum of the proportion score (0 = no staining, 1 = <1% of cells staining, 2 = 1 - 10% of cells staining, 3 = 11-30% of cells staining, 4 = 31-60% of cells staining, 5 = >60% of cells staining) to the intensity score (1 = weak intensity of staining, 2 = intermediate intensity of staining, 3 = strong intensity of staining), with a scoring range from 0 to 8.
ER/PR positive is defined as an Allred score of >2 and ER/PR negative is defined as an Allred score of less than or equal to 2.
Methodology: Fixation Type and Length: Tissue was fixed in 10% neutral buffered formalin
CT) for no less than 8 and no longer than 24 hours. Antibody and Assay Methodology: Mouse anti-human ER and PR,
Comment: This assay can be used to select invasive breast cancer patients for hormone therapy (1).
ER and PR analysis was performed on this case by immunohistochemistry utilizing the ER (ER 1D5, 1:100) and PR (PGR 136, 1:100) antibody provided by , following the manufacturer's instructions listed in the package insert. This assay was not modified, and adherence to all instruction and guidelines were strictly followed. Interpretation of the ER/PR immunohistochemical staining characteristics is guided by published results in the medical literature (1), information provided by the reagent manufacturer and by internal review of staining performance within Pathology Department.

1. Harvey JM, et al. Estrogen receptor status by immunohistochemistry is superior to the ligand-binding assay for predicting response to adjuvant endocrine therapy in breast cancer. J Clin Oncol. 17:1474-1481, 1999

SYNOPTIC REPORT - BREAST HER-2 RESULTS
Specimens Involved
Specimens: B: RIGHT BREAST

HER2 Status Results, Immunohistochemistry Evaluation
SPECIMEN
Surgical Excision
Block Number: Block
B9
Interpretation: Negative
Intensity: 0
% Tumor Staining: 0%
FISH Ordered NO
METHODOLOGY
Methodology: Fixation Type and Length: Tissue was fixed in 10% neutral buffered formalin
for no less than 8 and no longer than 24 hours. Antibody and Assay Methodology: Rabbit anti-human HER2, HerceptestTM (FDA-approved test kit), (A). Control Slides Examined: External kit-slides provided by manufacturer (cell lines with high, low and negative HER2 protein expression), and in-house known HER2 amplified control tissue were evaluated along with the test tissue. These control slides run along side of this patient's sample showed appropriate staining. Adequacy of Specimen: Adequate, well preserved, clear-cut invasive carcinoma identified for HER2 evaluation.
Scoring Criterion and Scoring System:
IHC Level of Expression(Score) /Tumor Cell Membrane Staining Pattern
Negative (0)/Absence of Staining
Negative (1+)/Faint Incomplete membrane Staining, >10% of Cells
Equivocal (2+)/Weak complete membrane Staining, >10% of Cells
Positive (3+)/Strong complete membrane Staining, >10% of Cells
Equivocal Category for HER2 IHC results: A HER2, 2+ staining result that is interpreted as equivocal may not indicate gene amplification. A FISH test for HER2 gene amplification will be ordered for all HER2 IHC 2+ results.
COMMENT
This assay can be used to select invasive breast cancer patients for Trastuzumab (Hereptin) therapy (1,2). Clinical Trials have shown that Trastuzumab substantially increases the likelihood for an objective response and overall survival for patients with metastatic HER2-positive breast cancer, regardless of whether HER2 tumor status was determined as IHC 3+ or FISH positive. Trastuzumab added to adjuvant chemotherapy substantially increase disease-free survival and decreases the risk of disease recurrence by about 50% for patients with early-stage HER2 protein over-expressed or gene amplified invasive breast cancer (3).
HER2 analysis was performed on this case by immunohistochemistry utilizing the FDA approved Dako HercepTest (TM) test kit following the manufacturer's instructions listed in the package insert. This assay was not modified, and adherence to all instruction and guidelines were strictly followed. Interpretation of the HER2 immunohistochemical staining characteristics is guided by published results in the medical literature (4), information provided by the reagent manufacturer and by internal review of staining performance within the Pathology Department.
HER2 TEST VALIDATION
This HER2 immunohistochemical assay has been validated according to the recently revised recommendations and guidelines from the NCCN HER2 testing in Breast Cancer Task Force, and the jointly issued recommendations and guidelines from ASCO and the CAP (5). 80 randomly selected breast cancer samples were tested for HER2 by IHC as outline above and interpreted as, negative (score 0/1+) equivocal (score 2+) and positive (score 3+) without knowledge of the previous reported results.
These cases were also blindly read using two different FISH assay as amplified or non-amplified and the HER2/CEP17 ratios were recorded. After analyzing these results, there was 100% concordance between the IHC

and FISH results for cases that were interpreted as either positive or negative by IHC. 9 of the 80 cases were interpreted as equivocal by IHC and of these 3/9 (33%) were non-amplified by FISH and 6/9 (66%) were found to be amplified.

The Pathology Department Immunohistochemistry laboratory takes full responsibility for this tests performance and has programs in place to regularly monitor the proficiency and the interpretation of HER2 assays. The laboratory also participates in external quality assurance HER2 programs including the CAP proficiency testing program.

REFERENCE

1. Carlson RW, Anderson BO, Burstein HJ, et al., NCCN breast cancer clinical practice guidelines in oncology. J Natl Compr Canc Netw. 2005;3:238-289.
2. Carlson RW, Brown E, Burstein HJ, et al., NCCN Task Force Report: adjuvant therapy for breast cancer. J Natl Compr Canc Netw. 2006;4:S1-S26.
3. Romond EH, Perez EA, Bryant J, et al. Trastuzumab plus adjuvant chemotherapy for operable HER2-positive breast cancer. N Eng J Med 2005;353(16):1673-84
4. Leong ASY, Formby M, Haffajee Z, et al. *Refinement of immunohistologic parameters for Her2/neu scoring* validation by FISH and CISH. Appl Immunohistochem Mol Morphol. 2006;14:384-389.
5. Wolff AC, Hammond EH, Schwartz JN, et al., American Society of Clinical Oncology/College of American Pathologists Guideline Recommendations for Human Epidermal Growth Factor Recepto 2 Testing in Breast Cancer. Arch of Path and Lab Med 2007; 131:18-43.

Immunostain results done on the smaller second tumor (section B11) are as follows:

ER: Negative (0%)
PR: Negative (0%)

SYNOPTIC REPORT - BREAST HER-2 RESULTS
HER2 Status Results, Immunohistochemistry Evaluation
SPECIMEN
Surgical Excision
Block Number: Block
B1
Interpretation: Equivocal
Intensity: 2+
% Tumor Staining: 20%
FISH Ordered YES DATE
METHODOLOGY

Methodology: Fixation Type and Length: Tissue was fixed in 10% neutral buffered formalin [illegible] d, for no less than 8 and no longer than 24 hours. Antibody and Assay Methodology: Rabbit anti-human HER2, HerceptestTM (FDA-approved test kit), [illegible] Control Slides Examined: External kit-slides provided by manufacturer (cell lines with high, low and negative HER2 protein expression), and in-house known HER2 amplified control tissue were evaluated along with the test tissue. These control slides run along side of this patient's sample showed appropriate staining. Adequacy of Specimen: Adequate, well preserved, clear-cut invasive carcinoma identified for HER2 evaluation.

Scoring Criterion and Scoring System:
IHC Level of Expression(Score) /Tumor Cell Membrane Staining Pattern
Negative (0)/Absence of Staining
Negative (1+)/Faint Incomplete membrane Staining, >10% of Cells
Equivocal (2+)/Weak complete membrane Staining, >10% of Cells
Positive (3+)/Strong complete membrane Staining, >10% of Cells
Equivocal Category for HER2 IHC results: A HER2, 2+ staining result that is interpreted as equivocal may not indicate gene amplification. A FISH test for HER2 gene amplification will be ordered for all HER2 IHC 2+ results.

COMMENT

This assay can be used to select invasive breast cancer patients for Trastuzumab (Hereptin) therapy (1,2). Clinical Trials have shown that Trastuzumab substantially increases the likelihood for an objective response and overall survival for patients with metastatic HER2-positive breast cancer, regardless of whether HER2 tumor status was determined as IHC 3+ or FISH positive. Trastuzumab added to adjuvant chemotherapy substantially increase disease-free survival and decreases the risk of disease recurrence by about 50% for patients with early-stage HER2 protein over-expressed or gene amplified invasive breast cancer (3).

HER2 analysis was performed on this case by immunohistochemistry utilizing the FDA approved Dako HercepTest (TM) test kit following the manufacturer's instructions listed in the package insert. This assay was not modified, and adherence to all instruction and guidelines were strictly followed. Interpretation of the HER2 immunohistochemical staining characteristics is guided by published results in the medical literature (4), information provided by the reagent manufacturer and by internal review of staining performance within the Pathology Department.

HER2 TEST VALIDATION

This HER2 immunohistochemical assay has been validated according to the recently revised recommendations and guidelines from the NCCN HER2 testing in Breast Cancer Task Force, and the jointly issued recommendations and guidelines from ASCO and the CAP (5). 80 randomly selected breast cancer samples were tested for HER2 by IHC as outline above and interpreted as, negative (score 0/1+) equivocal (score 2+) and positive (score 3+) without knowledge of the previous reported results.

These cases were also blindly read using two different FISH assay as amplified or non-amplified and the HER2/CEP17 ratios were recorded. After analyzing these results, there was 100% concordance between the IHC and FISH results for cases that were interpreted as either positive or negative by IHC. 9 of the 80 cases were interpreted as equivocal by IHC and of these 3/9 (33%) were non-amplified by FISH and 6/9 (66%) were found to be amplified.

The Pathology Department Immunohistochemistry laboratory takes full responsibility for this tests performance and has programs in place to regularly monitor the proficiency and the interpretation of HER2 assays. The laboratory also participates in external quality assurance HER2 programs including the CAP proficiency testing program.

REFERENCE

1. Carlson RW, Anderson BO, Burstein HJ, et al., NCCN breast cancer clinical practice guidelines in oncology. J Natl Compr Canc Netw. 2005;3:238-289.
2. Carlson RW, Brown E, Burstein HJ, et al., NCCN Task Force Report: adjuvant therapy for breast cancer. J Natl Compr Canc Netw. 2006;4:S1-S26.
3. Romond EH, Perez EA, Bryant J, et al. Trastuzumab plus adjuvant chemotherapy for operable HER2-positive breast cancer. N Eng J Med 2005;353(16):1673-84
4. Leong ASY, Formby M, Haffajee Z, et al. Refinement of immunohistologic parameters for Her2/neu scoring validation by FISH and CISH. Appl Immunohistochem Mol Morphol. 2006;14:384-389.
5. Wolff AC, Hammond EH, Schwartz JN, et al., American Society of Clinical Oncology/College of American Pathologists Guideline Recommendations for Human Epidermal Growth Factor Recepto 2 Testing in Breast Cancer. Arch of Path and Lab Med 2007; 131:18-43.

Gross Dictation:
Microscopic/Diagnostic Dictation: Pathologist.
Final Review: Pathologist,
Microscopic/Diagnostic Dictation: Pathologist,
Microscopic/Diagnostic Dictation: Pathologist,
Final Review: Pathologist,
Final: Pathologist,
Addendum: Pathologist,
Addendum Review: Pathologist,
Addendum Final: Pathologist,
Addendum: PATHOLOGIST,
Addendum Review: PATHOLOGIST
Addendum Final: PATHOLOGIST.
Addendum: Pathologist
Addendum Review: Pathologist
Addendum Final: Pathologist,
Addendum: PATHOLOGIST,
Addendum Review: PATHOLOGIST,
Addendum Final: PATHOLOGIST,

Criteria	Yes	No
Diagnosis Discrepancy		✓
Primary Tumor Site Discrepancy		✓
HIPAA Discrepancy		✓
Prior Malignancy History		✓
Dual/Synchronous Primary Noted		✓
Case is (circle): QUALIFIED / DISQUALIFIED		
Reviewer Initials [illegible] Date Reviewed [illegible]		

ICD-0-3
Carcinoma, infiltrating duct, NOS
8500/3 12/8/10
lw
Path Site Code: breast, upper outer quadrant C50.4
CQCF Site: breast, NOS C50.9

TSS

UUID:42A1A073-69B4-4A66-A998-BE4366E0C89C
TCGA-E2-A14Q-01A-PR
Redacted

SPECIMENS:

A. WLE RIGHT BREAST NEEDLE LOCALIZATION
B. LEFT SIMPLE MASTECTOMY
C. SENTINEL LYMPH NODE #1 LEFT AXILLA
D. SENTINEL LYMPH NODE #2 LEFT AXILLA
E. SENTINEL LYMPH NODE #3 LEFT AXILLA
F. SENTINEL LYMPH NODE #4 LEFT AXILLA
G. SENTINEL LYMPH NODE #5 LEFT AXILLA

SPECIMEN(S):

A. WLE RIGHT BREAST NEEDLE LOCALIZATION
B. LEFT SIMPLE MASTECTOMY
C. SENTINEL LYMPH NODE #1 LEFT AXILLA
D. SENTINEL LYMPH NODE #2 LEFT AXILLA
E. SENTINEL LYMPH NODE #3 LEFT AXILLA
F. SENTINEL LYMPH NODE #4 LEFT AXILLA
G. SENTINEL LYMPH NODE #5 LEFT AXILLA

INTRAOPERATIVE CONSULTATION DIAGNOSIS:

TPC, D, E, F, G: Sentinel lymph nodes #1-5, biopsies: No tumor seen.
By Drcalled to Dr. a

GROSS DESCRIPTION:

A. WLE RIGHT BREAST NEEDLE LOCALIZATION
Received fresh labeled with the patient's name and "WLE right breast needle localization excision atypical hyperplasia, single-ant, double lateral, triple superior" is a 28 gm oriented wide local excision breast specimen, 6.0 cm from superior to inferior, 4.0 cm from lateral to medial and 2.0 cm from anterior to posterior, with needle localization wire and attached radiograph. The specimen is inked as follows: superior-red, inferior-orange, medial-green, lateral-yellow, anterior-blue, posterior-black. The specimen is serially sectioned from lateral to medial into 6 slices; slice 1 being most lateral, slice 6 being most medial to reveal multiple gray-white nodular areas, the largest of which measures 0.9 cm in greatest dimension and is located 0.3 cm from the anterior margin. The entire specimen is submitted as follows:

A1-A3: lateral margin perpendicular sections taken from superior to inferior slice 1
A4: superior margin slice 2
A5: anterior and posterior margin slice 2
A6: inferior margin slice 2
A7: superior margin slice 3
A8: anterior and deep margin slice 3
A9: inferior margin slice 3
A10: superior margin slice 4
A11: anterior and deep margins slice 4
A12: inferior margin slice 4
A13: superior margin slice 5
A14: anterior and deep margin slice 5
A15: inferior margin slice 5
A16-A17: medial margin perpendicular sections submitted sequentially from superior to inferior slice 6

B. LEFT SIMPLE MASTECTOMY
Received fresh labeled with the patient's name and "left simple mastectomy, stitch in axilla" is a 560 g, 21 x 20 x 3 cm mastectomy with an 11 x 5.7 cm skin ellipse with 0.5 cm well healed scar in the lower outer quadrant, 1.5 cm areola, and a 1 cm everted nipple. Inked as follows: superior anterior = blue, inferior anterior = orange, deep margin = black. The specimen is serially sectioned revealing a 2.5 x 2.3 x 1.7 cm well-circumscribed firm tan mass in the upper outer quadrant that is 1.5 cm from the deep margin. A portion of tumor is submitted for tissue procurement. In the lower outer quadarnt, there is a 1.2 x 1.0 x 0.6 cm hemorrhagic biopsy site, 3.7 cm from the mass. 0.3 cm lateral to the biopsy site is a firm tan nodule, 1.3 cm in diameter. One lymph node is identified near the axillary stitch.
Representatively submitted:

B1-B6: mass from upper outer quadrant
B7-B9: area of biopsy
B10: nodule near biopsy site
B11: upper inner quadrant
B12: upper outer quadrant
B13: lower outer quadrant
B14: lower inner quadrant
B15-B16: areas of possible calcification from upper outer quadrant
B17-B18: nipple
B19: possible axillary lymph nodes

C. SENTINEL LYMPH NODE #1, LEFT AXILLA

Received fresh are 2 tan-pink lymph nodes, 2.0 x 0.9 x 0.9 cm and 0.5 x 0.4 x 0.3 cm. The specimen is serially sectioned and 2 touch preps are taken. The specimen is submitted entirely as follows:
C1: 1 lymph node
C2: 1 lymph node

D. SENTINEL LYMPH NODE #2, LEFT AXILLA
Received fresh is a tan-pink lymph node, 1.0 x 0.6 x 0.6 cm. The specimen is serially sectioned and touch preps are taken. Toto D1.

E. SENTINEL LYMPH NODE #3, LEFT AXILLA
Received fresh is a tan-pink lymph node, 0.3 x 0.2 x 0.2 cm. The specimen is bisected and touch preps are taken. Toto E1.

F. SENTINEL LYMPH NODE #4, LEFT AXILLA
Received fresh is a tan-pink lymph node, 1.4 x 0.8 x 0.2 cm. The specimen is serially sectioned and touch preps are taken. Specimen is submitted entirely in cassette F1.

G. SENTINEL LYMPH NODE #5, LEFT AXILLA
Received fresh is a tan-pink lymph node, 1.8 x 1.0 x 1.0 cm. The specimen is serially sectioned and touch preps are taken and the specimen is submitted entirely in cassette G1.

RESULTS:

SUMMARY OF IMMUNOHISTOCHEMISTRY/SPECIAL STAINS

Material: Block B4
Population: Tumor Cells

Stain/Marker:	Result:	Comment:
ECADHERIN	Positive	

The interpretation of the above immunohistochemistry stain or stains is guided by published results in the medical literature, provided package information from the manufacturer and by internal review of staining performance and assay validation within the Immunohistochemistry Laboratory. The use of one or more reagents in the above tests is regulated as an analyte specific reagent (ASR). These tests were developed and their performance characteristic determined by the [illegible]. They have not been cleared or approved by the U.S. Food and Drug Administration. The FDA has determined that such clearance or approval is not necessary.

DIAGNOSIS:

A. BREAST, RIGHT, NEEDLE LOCALIZATION WIDE LOCAL EXCISION:
- LOBULAR CARCINOMA IN SITU.
- SMALL INTRADUCTAL PAPILLOMA, RADIAL SCAR, FLORID USUAL DUCTAL HYPERPLASIA, COLUMNAR CELL LESIONS, EXTENSIVE SCLEROSING ADENOSIS, DUCT ECTASIA, AND MICROCALCIFICATIONS.
- FOCAL PREVIOUS BIOPSY SITE CHANGES (SEE NOTE).

NOTE: Focal previous biopsy site changes are present in slide A5.

B. BREAST, LEFT, MASTECTOMY:
- INVASIVE DUCTAL CARCINOMA.
    - SBR GRADE 2.
    - 2.5 CM IN GREATEST DIMENSION.
    - MARGINS, NEGATIVE FOR CARCINOMA.
- EXTENSIVE DUCTAL CARCINOMA IN SITU (DCIS), SOLID AND CRIBRIFORM TYPES, NUCLEAR GRADES 2 & 3, WITH COMEDO NECROSIS AND MICROCALCIFICATIONS, INVOLVING LOBULES.
    - DCIS IS FOCALLY WITHIN 1 MM OF THE ANTERIOR-SUPERIOR MARGIN.
    - DCIS IS PRESENT IN UPPER OUTER AND LOWER OUTER QUADRANTS.
- NIPPLE, NEGATIVE FOR CARCINOMA.
- ONE LYMPH NODE, NEGATIVE FOR CARCINOMA (0/1).

NOTE: Three biopsy sites were identified, associated with the tumor, a fibroadenoma and hemorrhage.

C. SENTINEL LYMPH NODE #1, LEFT AXILLA, BIOPSY:
- TWO LYMPH NODES, NEGATIVE FOR CARCINOMA (0/2).

D. SENTINEL LYMPH NODE #2, LEFT AXILLA, BIOPSY:
- ONE LYMPH NODE, NEGATIVE FOR CARCINOMA (0/1).

E. SENTINEL LYMPH NODE #3, LEFT AXILLA, BIOPSY:
- ONE LYMPH NODE, NEGATIVE FOR CARCINOMA (0/1).

F. SENTINEL LYMPH NODE #4, LEFT AXILLA, BIOPSY:
- MICROMETASTATIC CARCINOMA (1.1 MILLIMETERS) TO ONE LYMPH NODE (1/1) (SEE NOTE).

NOTE: The touch prep was reviewed and shows no evidence of carcinoma.

G. SENTINEL LYMPH NODE #5, LEFT AXILLA, BIOPSY:
- ONE LYMPH NODE, NEGATIVE FOR CARCINOMA (0/1).

SYNOPTIC REPORT - BREAST
Specimen Type: Mastectomy
Needle Localization: No
Laterality: Left
Invasive tumor: Present
Multifocality: No
WHO CLASSIFICATION
Invasive ductal carcinoma, NOS 8500/3
Tumor size: 2.5cm
Tumor site: Upper outer quadrant
Margins: Negative
Distance from closest margin: 0.6cm
anterior
Tubular score: 3
Nuclear grade: 2
Mitotic score: 1
Modified Scarff Bloom Richardson Grade: 2
Necrosis: Absent
Vascular/Lymphatic Invasion: None identified
Lymph nodes: Sentinel lymph node only
Lymph node status: Positive 1 / 7
Micrometastases: Yes
Non-neoplastic areas: fibroadenoma, columnar cell change

DCIS present
Margins uninvolved by DCIS:
DCIS Quantity: Estimate 40%
DCIS type: Solid
Cribriform
DCIS location: Both associated and separate from invasive tumor mass
Nuclear grade: High
Necrosis: Present
Location of CA++: DCIS
Benign epithelium

ER/PR/HER2 Results
Performed on Case: (mastectomy)
ER: Positive
PR: Positive
HER2: Negative by IHC

Pathological staging (pTN): pT 2 N 1mi

SYNOPTIC REPORT - BREAST, ER/PR RESULTS
Specimen: Surgical Excision
Block Number: B1

ER: Positive Allred Score: 8 = Proportion score: 5 + Intensity Score 3
PR: Positive Allred Score: 8 = Proportion Score 5 + Intensity Score 3

COMMENT:
The Allred score for estrogen and progesterone receptors is calculated by adding the sum of the proportion score (0 = no staining, 1 = <1% of cells staining, 2 = 1 - 10% of cells staining, 3 = 11-30% of cells staining, 4 = 31-60% of

cells staining, 5 = >60% of cells staining) to the intensity score (1 = weak intensity of staining, 2 = intermediate intensity of staining, 3 = strong intensity of staining), with a scoring range from 0 to 8.
ER/PR positive is defined as an Allred score of >2 and ER/PR negative is defined as an Allred score of less than or equal to 2.
Methodology: Fixation Type and Length: Tissue was fixed in 10% neutral buffered formalin (
for no less than 8 and no longer than 24 hours. Antibody and Assay Methodology: Mouse anti-human ER and PR, (
Comment: This assay can be used to select invasive breast cancer patients for hormone therapy (1).
ER and PR analysis was performed on this case by immunohistochemistry utilizing the ER (ER 1D5, 1:100) and PR (PGR 136, 1:100) antibody provided by Dako, following the manufacturer's instructions listed in the package insert. This assay was not modified, and adherence to all instruction and guidelines were strictly followed. Interpretation of the ER/PR immunohistochemical staining characteristics is guided by published results in the medical literature (1), information provided by the reagent manufacturer and by internal review of staining performance within the Pathology Department.
1. Harvey JM, et al. Estrogen receptor status by immunohistochemistry is superior to the ligand-binding assay for predicting response to adjuvant endocrine therapy in breast cancer. J Clin Oncol. 17:1474-1481, 1999

SYNOPTIC REPORT - BREAST HER-2 RESULTS
HER2 Status Results, Immunohistochemistry Evaluation
Specimen: Surgical Excision
Block Number: B1

Interpretation: NEGATIVE
Intensity: 1+
% Tumor Staining: 1%
Fish Ordered: No

METHODOLOGY
Methodology: Fixation Type and Length: Tissue was fixed in 10% neutral buffered formalin (
for no less than 8 and no longer than 24 hours. Antibody and Assay Methodology: Rabbit anti-human HER2, HerceptestTM (FDA-approved test kit), [illegible]. Control Slides Examined: External kit-slides provided by manufacturer (cell lines with high, low and negative HER2 protein expression), and in-house known HER2 amplified control tissue were evaluated along with the test tissue. These control slides run along side of this patient's sample showed appropriate staining. Adequacy of Specimen: Adequate, well preserved, clear-cut invasive carcinoma identified for HER2 evaluation.
Scoring Criterion and Scoring System:
IHC Level of Expression(Score) /Tumor Cell Membrane Staining Pattern
Negative (0)/Absence of Staining
Negative (1+)/Faint Incomplete membrane Staining, >10% of Cells
Equivocal (2+)/Weak complete membrane Staining, >10% of Cells
Positive (3+)/Strong complete membrane Staining, >10% of Cells
Equivocal Category for HER2 IHC results: A HER2, 2+ staining result that is interpreted as equivocal may not indicate gene amplification. A FISH test for HER2 gene amplification will be ordered for all HER2 IHC 2+ results.
COMMENT
This assay can be used to select invasive breast cancer patients for Trastuzumab (Hereptin) therapy (1,2). Clinical Trials have shown that Trastuzumab substantially increases the likelihood for an objective response and overall survival for patients with metastatic HER2-positive breast cancer, regardless of whether HER2 tumor status was determined as IHC 3+ or FISH positive. Trastuzumab added to adjuvant chemotherapy substantially increase disease-free survival and decreases the risk of disease recurrence by about 50% for patients with early-stage HER2 protein over-expressed or gene amplified invasive breast cancer (3).
HER2 analysis was performed on this case by immunohistochemistry utilizing the FDA approved Dako HercepTest (TM) test kit following the manufacturer's instructions listed in the package insert. This assay was not modified, and adherence to all instruction and guidelines were strictly followed. Interpretation of the HER2 immunohistochemical staining characteristics is guided by published results in the medical literature (4), information provided by the reagent manufacturer and by internal review of staining performance within the Pathology Department.
HER2 TEST VALIDATION
This HER2 immunohistochemical assay has been validated according to the recently revised recommendations and guidelines from the NCCN HER2 testing in Breast Cancer Task Force, and the jointly issued recommendations and guidelines from ASCO and the CAP (5). 80 randomly selected breast cancer samples were tested for HER2 by IHC as outline above and interpreted as, negative (score 0/1+) equivocal (score 2+) and positive (score 3+) without knowledge of the previous reported results.
These cases were also blindly read using two different FISH assay as amplified or non-amplified and the HER2/CEP17 ratios were recorded. After analyzing these results, there was 100% concordance between the IHC and FISH results for cases that were interpreted as either positive or negative by IHC. 9 of the 80 cases were interpreted as equivocal by IHC and of these 3/9 (33%) were non-amplified by FISH and 6/9 (66%) were found to be amplified.
The Pathology Department Immunohistochemistry laboratory takes full responsibility for this tests performance and has programs in place to regularly monitor the proficiency and the interpretation of HER2 assays. The

laboratory also participates in external quality assurance HER2 programs including the CAP proficiency testing program.

REFERENCE

1. Carlson RW, Anderson BO, Burstein HJ, et al., NCCN breast cancer clinical practice guidelines in oncology. J Natl Compr Canc Netw. 2005;3:238-289.
2. Carlson RW, Brown E, Burstein HJ, et al., NCCN Task Force Report: adjuvant therapy for breast cancer. J Natl Compr Canc Netw. 2006;4:S1-S26.
3. Romond EH, Perez EA, Bryant J, et al. Trastuzumab plus adjuvant chemotherapy for operable HER2-positive breast cancer. N Eng J Med 2005;353(16):1673-84
4. Leong ASY, Formby M, Haffajee Z, et al. Refinement of immunohistologic parameters for Her2/neu scoring validation by FISH and CISH. Appl Immunohistochem Mol Morphol. 2006;14:384-389.
5. Wolff AC, Hammond EH, Schwartz JN, et al., American Society of Clinical Oncology/College of American Pathologists Guideline Recommendations for Human Epidermal Growth Factor Recepto 2 Testing in Breast Cancer. Arch of Path and Lab Med 2007; 131:18-43.

PRE-OPERATIVE DIAGNOSIS:

Left Breast Cancer, Right Atypical Hyperplasia

ADDENDUM:

NOTE: Addition to gross description for specimen B – a representative section of skin is submitted in cassette B20 and microscopically, shows no evidence of carcinoma.

ONCOTYPE DX BR[illegible]T CANCER ASSAY

RESULTS: Recurr[illegible]e Score =: 9

CLINICAL EXPE[illegible]NCE: Patients with a recurrence score of: 9 in the clinical validation study had an average rate of Distant [illegible]urrence at 10 years of 7%

ER Score: Positive 10.8
PR Score: Positive 8.5
Interpretation: Positive ER Score is >= 6.5 Positive PR Score is >= 5.5

See separate [illegible] report for further information.
Test performed at:

Gross Dictation: Pathologist,
Microscopic/Diagnostic Dictation: Patholog
Microscopic/Diagnostic Dictation: Pathologist
Final Review: Pathologist,
Final Review: Pathologist,
Final: Pathologist,
Addendum: Pathologist
Addendum Final: Pathologist,
Addendum: Pathologist,
Addendum Final: Pathologist

Criteria	Yes	No
Diagnosis Discrepancy		✓
Primary Tumor Site Discrepancy		✓
HIPAA Discrepancy		✓
Prior Malignancy History		✓
Dual/Synchronous Primary Noted		✓
Case is (circle): QUALIFIED / DISQUALIFIED		
Reviewer Initials [illegible] Date Reviewed: [illegible]		

ICD-0-3
Carcinoma, infiltrating duct, NOS
8500/3 12/8/10
Path Site Code: breast, central portion C50.1 lw
CQCF Site: breast, NOS C50.9

UUID:0B6DCA80-3108-4D25-B095-3EC704598B23
TCGA-E2-A14R-01A-PR Redacted

TSS

SPECIMENS:
- A. SENTINEL NODE #1 LEFT AXILLA
- B. SENTINEL NODE #2 LEFT AXILLA
- C. SENTINEL NODE #3 LEFT AXILLA
- D. WIDE LOCAL EXCISION LEFT BREAST NEEDLE LOCALIZATION
- E. INFERIOR MARGIN LEFT BREAST
- F. LATERAL MARGIN LEFT BREAST
- G. MEDIAL MARGIN LEFT BREAST
- H. POSTERIOR MARGIN LEFT BREAST
- I. SENTINEL NODE #4 LEFT AXILLA
- J. SENTINEL NODE #5 LEFT AXILLA
- K. SENTINEL NODE #6 LEFT AXILLA

SPECIMEN(S):
- A. SENTINEL NODE #1 LEFT AXILLA
- B. SENTINEL NODE #2 LEFT AXILLA
- C. SENTINEL NODE #3 LEFT AXILLA
- D. WIDE LOCAL EXCISION LEFT BREAST NEEDLE LOCALIZATION
- E. INFERIOR MARGIN LEFT BREAST
- F. LATERAL MARGIN LEFT BREAST
- G. MEDIAL MARGIN LEFT BREAST
- H. POSTERIOR MARGIN LEFT BREAST
- I. SENTINEL NODE #4 LEFT AXILLA
- J. SENTINEL NODE #5 LEFT AXILLA
- K. SENTINEL NODE #6 LEFT AXILLA

INTRAOPERATIVE CONSULTATION DIAGNOSIS:
TPA-TPC: One lymph node each part, negative for carcinoma
D: Gross assessment 2.0 cm mass >0.5 cm all margins and 1.0 cm satellite nodule at inferior margin
TPI: One lymph node, negative for carcinoma
TPJ/FSJ: 2 lymph nodes, negative for carcinoma
TPK: One lymph node, negative for carcinoma
By Dr. called to Dr. at (A), (B), C), (D) and (I, J, K).

GROSS DESCRIPTION:

A. SENTINEL NODE #1, LEFT AXILLA
Received fresh and labeled with the patient name designated "A – sentinel node #1, left axilla", is a fragment of beige-tan possible lymphoid tissue measuring 2.0 x 2.0 x 1.2 cm. The specimen is serially sectioned, touch prep performed. The entire specimen is submitted in cassette A1 and A2.

B. SENTINEL NODE #2, LEFT AXILLA
Received fresh and labeled with the patient name designated "B – sentinel node #2, left axilla", is a fragment of beige-tan possible lymphoid tissue measuring 1.0 x 1.0 x 1.3 cm. The specimen is serially sectioned, touch prep performed. The entire specimen is submitted in cassette B1 and B2.

C. SENTINEL NODE #3, LEFT AXILLA
Received fresh and labeled with the patient name designated "C – sentinel node #3", is a fragment of beige-tan possible lymphoid tissue measuring 0.7 x 0.5 x 0.3 cm. The specimen is serially sectioned, touch prep performed. The entire specimen is submitted in cassette C1.

D. LEFT BREAST WIDE NEEDLE LOCALIZATION
Received in fresh state the specimen labeled with patients name and identification number and labeled as wide local excision left breast needle localization. The specimen consists of 293 grams resected breast tissue measuring 12.5 cm from medial to lateral, 7.5 cm from anterior to posterior and 6.0 cm from superior to inferior. The margins of specimen are oriented with sutures, single indicating anterior, double-lateral and triple-superior. There is a localization needle coursing from the superior to the inferior aspect of the specimen and the accompanying radiogram that shows density located in the mid portion of the specimen. The margins of specimen are color coded as follows: Inferior-orange, superior-red, lateral-yellow, anterior-blue, medial-green and posterior-black. At mid portion of the specimen is a firm palpable mass which on cut section shows solid firm mass with nodular and lobulated pushing borders with a tan white cut surface with total dimensions of 4.0 x 3.0 x 0.9 cm. A metallic clip is identified within the main portion of the tumor and the surrounding breast tissue consists mostly of fatty parenchyma. The tumor grossly 0.9 cm from the nearest inferior margin. Multiple sections are submitted and labeled as follows:
D1-D8: One en bloc section of tumor with margins

D9: Cross section of tumor
D10-D18: En bloc section of tumor with margins, (block 15 section taken site of apparent previous biopsy site wherein a clip was identified)
D19: Sections of gross fat necrosis
D20: Sections from posterior and medial margins
D21: Sections from inferior and medial margins
D22-D23: Sections from the posterior margin
D24-D25: Sections from lateral margin
D26-D27: Additional medial margin

E. LEFT BREAST INFERIOR MARGIN
Stitch marks new margin. Received fresh is an oriented 17.0-gram fragment of fibrofatty tissue 6.0 x 5.0 x 2.0 cm. The new true margin is inked blue, the specimen is serially sectioned and submitted in toto in cassette E1-E11.

F. LEFT BREAST LATERAL MARGIN
Stitch at new lateral margin. Received fresh is an oriented tan-pink fragment of fibrofatty tissue 5.0 x 2.0 x 1.5 cm. The new true margin is inked blue. Also separate within the container is an unoriented tan-pink fragment of fibrofatty tissue 5.5 x 4.0 x 2.0 cm. The specimen is inked blue. The entire specimen is submitted as follows:
F1-F5: Oriented tissue fragment
F6-F12: Unoriented tissue fragment.

G. LEFT BREAST MEDIAL MARGIN
Irregular fragment of fibrofatty tissue measuring 3.5 x 2.0 x 1.0 cm. Submitted in toto in cassettes labeled G1-G2.

H. LEFT BREAST POSTERIOR MARGIN
Received fresh is an unoriented 3.0-gram tan-pink fragment of fibrofatty tissue 4.0 x 2.0 x 1.5 cm. The specimen is inked blue, serially sectioned and submitted in toto in cassette H1 and H2.

I. SENTINEL NODE #4, LEFT AXILLA
Received fresh is a tan-pink lymph node 0.8 x 0.7 x 0.7 cm. The specimen is serially sectioned, touch preps are taken. The specimen is submitted entirely in cassette I1.

J. SENTINEL NODE #5, LEFT AXILLA
Received fresh are 2 tan-pink lymph nodes 1.5 x 1.0 x 1.0 cm and 0.8 x 0.5 x 0.5 cm. A portion of the larger lymph node is submitted for frozen section in FSJ1. Touch preps are taken. The remainder of the lymph node is submitted as follows:
J2-J3: One lymph node
J4: One lymph node

K. SENTINEL NODE #6, LEFT AXILLA
Received fresh is a tan-pink lymph node 0.7 x 0.6 x 0.5 cm. The specimen is bisected. Touch preps are taken and the specimen is submitted entirely in cassette K1.

DIAGNOSIS:

A. SENTINEL LYMPH NODE #1, LEFT AXILLA
- ONE LYMPH NODE, NEGATIVE FOR TUMOR (0/1).

B. SENTINEL LYMPH NODE #2, LEFT AXILLA
- ONE LYMPH NODE, NEGATIVE FOR TUMOR (0/1).

C. SENTINEL LYMPH NODE #3, LEFT AXILLA
- ONE LYMPH NODE, NEGATIVE FOR TUMOR (0/1).

D. LEFT BREAST, NEEDLE LOCALIZATION WIDE LOCAL EXCISION:
- INVASIVE DUCTAL CARCINOMA WITH AREAS OF NECROSIS, SBR GRADE III OF LEFT BREAST.
- SIZE OF TUMOR: 4.0 x 3.0 x 0.9 CM.
- PREDOMINANTLY FATTY BREAST TISSUE WITH FOCAL AREAS OF COLUMNAR CELL CHANGE.
- POST BIOPSY SITE CHANGES.
- MARGINS OF RESECTION-NEGATIVE FOR TUMOR.

E. LEFT BREAST, INFERIOR MARGIN:
- PREDOMINANTLY FATTY BREAST TISSUE-NEGATIVE FOR TUMOR.

F. LEFT BREAST, LATERAL MARGIN:
- FATTY BREAST TISSUE-NEGATIVE FOR TUMOR.

G. LEFT BREAST, MEDIAL MARGIN:

- FATTY TISSUE AND 1 MM FOCUS OF LYMPH NODE-NEGATIVE FOR TUMOR (0/1).

H. LEFT BREAST, POSTERIOR MARGIN:
- FATTY BREAST TISSUE INCLUDING SKELETAL MUSCLE TISSUE-NEGATIVE FOR TUMOR.

I. SENTINEL LYMPH NODE #4, LEFT AXILLA:
- ONE LYMPH NODE, NEGATIVE FOR TUMOR (0/1).

J. SENTINEL LYMPH NODE, #5 LEFT AXILLA:
- TWO LYMPH NODES, NEGATIVE FOR TUMOR (0/2)

K. SENTINEL LYMPH NODE #6, LEFT AXILLA:
- ONE LYMPH NODE, NEGATIVE FOR TUMOR (0/1).

Note: specimen # D- grossly, of what appears clinically as two tumor is only one large tumor with lobulated and nodulat extensions.the main bulk of the specimen consist mostly of fatty breast tissue.

SYNOPTIC REPORT - BREAST
Specimens Involved
Specimens: D: WIDE LOCAL EXCISION LEFT BREAST NEEDLE LOCALIZATION

Specimen Type: Excision
Needle Localization: Yes
Laterality: Left
Invasive Tumor: Present
Multifocality: No
WHO CLASSIFICATION
Invasive ductal carcinoma, NOS 8500/3
Tumor size: 4cm
Additional dimensions: 3cm x 0.9cm
Tumor Site: Central
Margins: Negative
Distance from closest margin: 1.5cm
superior
Tubular Score: 3
Nuclear Grade: 3
Mitotic Score: 3
Modified Scarff Bloom Richardson Grade: 3
Necrosis: Present
Vascular/Lymphatic Invasion: Indeterminate
Lobular neoplasia: None
Lymph nodes: Sentinel lymph node only
Lymph node status: Negative 0 / 8
Non-neoplastic areas: Post biopsy site changes

DCIS not present

Pathological staging (pTN): pT 2 N 0

SYNOPTIC REPORT - BREAST, ER/PR RESULTS
Specimens Involved
Specimens: D: WIDE LOCAL EXCISION LEFT BREAST NEEDLE LOCALIZATION

Specimen: Surgical Excision
Block Number: D9

ER: Negative Allred Score: 0 = Proportion Score 0 + Intensity Score 0
PR: Negative Allred Score: 0 = Proportion Score 0 + Intensity Score 0

COMMENT:
The Allred score for estrogen and progesterone receptors is calculated by adding the sum of the proportion score (0 = no staining, 1 = <1% of cells staining, 2 = 1 - 10% of cells staining, 3 = 11-30% of cells staining, 4 = 31-60% of cells staining, 5 = >60% of cells staining) to the intensity score (1 = weak intensity of staining, 2 = intermediate intensity of staining, 3 = strong intensity of staining), with a scoring range from 0 to 8.
ER/PR positive is defined as an Allred score of >2 and ER/PR negative is defined as an Allred score of less than or equal to 2.
METHODOLOGY:
Tissue was fixed in 10% neutral buffered formalin for no less than 8 and no longer than 24 hours. Immunohistochemistry was performed using the mouse anti-human ER (ER 1D5, 1:100) and PR (PGR 136, 1:100)

provided by Dako ^) following the manufacturer s instructions. This assay was not modified. Interpretation of the ER/PR immunohistochemical stain is guided by published results in the medical literature, information provided by the reagent manufacturer and by internal review of staining performance.

CLINICAL HISTORY:

-year-old Caucasian female with palpable left breast mass. Ultrasound showed 2 masses at 12 o'clock position, largest one 2.0 cm. Core biopsy showed DCIS, here for wide local excision and sentinel node biopsy.

PRE-OPERATIVE DIAGNOSIS:

None given

ADDENDUM:

SYNOPTIC REPORT - BREAST HER-2 RESULTS
Specimen: Surgical Excision
Block Number: D9

Interpretation: NEGATIVE
Intensity: 1+
% Tumor Staining: 8%
Fish Ordered: No

METHODOLOGY:

Tissue was fixed in 10% neutral buffered formalin for no less than 8 and no longer than 24 hours. Her2 analysis was performed using the FDA approved Dako HercepTest (TM) test kit (Dako, Carpenteria, CA) using rabbit anti-human HER2. This assay was not modified. External kit-slides provided by the manufacturer (cell lines with high, low and negative HER2 protein expression) and in-house known HER2 amplified control tissue were evaluated along with the test tissue. Adequate, well preserved, clear-cut invasive carcinoma was identified for HER2 evaluation. Interpretation of the HER2 immunohistochemical stain is guided by published results in the medical literature, information provided by the reagent manufacturer and by internal review of staining performance.

This assay has been validated according to the 2007 joint recommendations and guidelines from ASCO and CAP and from the NCCN HER2 testing in Breast Cancer Task Force. The Pathology Department takes full responsibility for this test's performance.

Gross Dictation: Pathologist,
Microscopic/Diagnostic Dictation: Pathologist.
Microscopic/Diagnostic Dictation: Pathologist,
Final Review: Pathologist.
Final: Pathologist,
Addendum: Pathologist,
Addendum Final: Pathologist

Criteria	Yes	No
Diagnosis Discrepancy		✓
Primary Tumor Site Discrepancy		✓
HIPAA Discrepancy		✓
Prior Malignancy History		✓
Dual/Synchronous Primary Noted		✓
Case is (circle): QUALIFIED / DISQUALIFIED		
Reviewer Initials [illegible] Date Reviewed: 11/2/10		

ICD-O-3

Carcinoma, infiltrating duct, NOS

8500/3 12/8/10

Path Site Code: breast, upper inner quadrant C50.2

CQCF Site: breast, NOS C50.9

TSS

UUID:6C8B56C1-330B-4116-8B60-7A81A1983277
TCGA-E2-A14T-01A-PR Redacted

SPECIMENS:

A. SLN #1
B. SLN #2
C. SENTINEL NODE #3 LEFT AXILLA
D. LEFT BREAST AND LOWER AXILLA TAIL

SPECIMEN(S):

A. SLN #1
B. SLN #2
C. SENTINEL NODE #3 LEFT AXILLA
D. LEFT BREAST AND LOWER AXILLA TAIL

GROSS DESCRIPTION:

A. SLN #1
Received fresh are two tan pink lymph nodes 0.9 x 0.6 x 0.5cm and 0.5 x 0.4 x 0.2cm. The specimen is serially sectioned and two touch preps are taken.
A1: 1 lymph node
A2: 1 lymph node
B. SLN #2
Received fresh is a tan pink lymph node 0.6 x 0.4 x 0.3cm. The specimen is serially sectioned and a touch prep is taken. Toto B1.
C. SLN #3 LEFT AXILLA
Received fresh is a tan pink lymph node 1.1 x 0.9 x 0.6cm. The specimen is serially sectioned and a touch prep is taken. Toto C1.
D. LEFT BREAST AND LOWER AXILLA-Stitch in axilla
Received fresh is an oriented 1314g, 30 x 28 x 6cm mastectomy with 15 x 6cm tan pink skin ellipse, 1.2 cm centrally located, partially raised nipple and 1.5 cm areolar rim. The specimen is inked as follows: Anterior/Superior-Blue, Anterior/Inferior-Orange, Posterior-Black. The specimen is serially sectioned from medial to lateral into 12 slices, slice 1 being most medial, slice 12 being most lateral. The nipple is located in slice 7. The cut surfaces reveal a gray white ill defined firm mass 3.5 x 2.8 x 2cm, 1.8cm from the deep margin located in slices 4, 5, 6 and 7. The area surrounding the mass is remarkable for fibrosis and possible fat necrosis. The lower axillary tail is 6 x 5 x 3cm. Dissection reveals 4 possible lymph nodes ranging from 0.5 x 0.5 x 0.5cm to 0.8 x 0.8 x 0.5cm. A portion of the specimen is submitted for tissue procurement. Representative sections are submitted as follows:
D1: nipple slice 7
D2: base of nipple slice 7
D3: UIQ area next to mass slice 3
D4-D5: mass bisected UIQ slice 4
D6: anterior margin UIQ slice 4
D7: deep margin UIQ slice 4
D8: mass UIQ slice 5
D9: deep margin UIQ slice 5
D10: skin slice 5
D11-D12: mass UIQ slice 6
D13: deep margin UIQ slice 6
D14: LIQ slice 6
D15-D16: mass bisected UC slice 7
D17: deep margin UC slice 7
D18: LC with inferior margin slice 7
D19: UOQ next to mass slice 8
D20: LOQ slice 8
D21: 2 lymph nodes
D22: 2 lymph nodes
D23-D26: lower axillary tissue

DIAGNOSIS:

A. SENTINEL LYMPH NODE #1, LEFT BREAST, BIOPSY:
- TWO LYMPH NODES, NEGATIVE FOR CARCINOMA (0/2).

B. SENTINEL LYMPH NODE #2, LEFT BREAST, BIOPSY:
- ONE LYMPH NODE, NEGATIVE FOR CARCINOMA (0/1).

C. SENTINEL LYMPH NODE #3, LEFT BREAST, BIOPSY:
- ONE LYMPH NODE, NEGATIVE FOR CARCINOMA (0/1).

D. BREAST, LEFT, MASTECTOMY:
- INVASIVE DUCTAL CARCINOMA, MODERATELY DIFFERENTIATED (SBR GRADE 2), WITH MICROPAPILLARY FEATURES (SEE NOTE).
    - TUMOR MEASURES 3.5 CM IN GREATEST DIMENSION.
    - MARGINS, NEGATIVE FOR CARCINOMA.
- DUCTAL CARCINOMA IN SITU, CRIBRIFORM AND SOLID TYPES, NUCLEAR GRADE 2, WITH NECROSIS AND MICROCALCIFICATIONS, MINOR COMPONENT.
- PREVIOUS BIOPSY SITE CHANGES PRESENT.
- SKIN AND NIPPLE, NEGATIVE FOR CARCINOMA.
- THREE LYMPH NODES, NEGATIVE FOR CARCINOMA (0/3).

NOTE: A CD31 immunostain has been ordered to rule out lymphvascular invasion and ER, PR, and Her FISH has been ordered. Those results will be reported in an addendum.
SYNOPTIC REPORT - BREAST
Specimen Type: Mastectomy
Needle Localization: No
Laterality: Left
Invasive Tumor: Present
Multifocality: No
WHO CLASSIFICATION
Invasive ductal carcinoma, NOS 8500/3
Tumor size: 3.5cm
Tumor Site: Upper inner quadrant
Margins: Negative
Distance from closest margin: Greater than 1cm
deep
Tubular Score: 3
Nuclear Grade: 2
Mitotic Score: 2
Modified Scarff Bloom Richardson Grade: 2
Necrosis: Absent
Lobular neoplasia: None
Lymph nodes: Sentinel lymph node only
Lymph node status: Negative 0 / 7

DCIS present
Margins uninvolved by DCIS
DCIS Quantity: Estimate 5%
DCIS Type: Solid
Cribriform
DCIS Location: Associated with invasive tumor
Nuclear grade: Intermediate
Necrosis: Present
Location of CA++: DCIS

Pathological staging (pTN): pT 2 N 0

CLINICAL HISTORY:
year old with multifocal Invasive Cancer in Upper Inner Quadrant of Left Breast

PRE-OPERATIVE DIAGNOSIS:
Left Breast Cancer

INTRAOPERATIVE CONSULTATION:
TPA/TPB/TPC: Negative for tumor. Diagnosis called to Dr. at y Dr.

ADDENDUM:
RESULTS:
SUMMARY OF IMMUNOHISTOCHEMISTRY/SPECIAL STAINS

Material: Block D12
Population: Tumor Cells

Stain/Marker:	Result:	Comment:
CD31	Negative	Shows no evidence of lymphvascular invasion

The interpretation of the above immunohistochemistry stain or stains is guided by published results in the medical literature, provided package information from the manufacturer and by internal review of staining performance and assay validation within the Immunohistochemistry Laboratory. The use of one or more reagents in the above tests is regulated as an analyte specific reagent (ASR). These tests were developed and their performance characteristic determined by the Department of Pathology Laboratory. They have not been cleared or approved by the U.S. Food and Drug Administration. The FDA has determined that such clearance or approval is not necessary.

Special stains and/or immunohistochemical stains were performed with appropriately stained positive and negative controls.

SYNOPTIC REPORT - BREAST, ER/PR RESULTS
Specimen: Surgical Excision
Block Number: D11

ER: Positive	Allred Score:	8 = Proportion Score 5 +	Intensity Score 3
PR: Positive	Allred Score:	4 = Proportion Score 2 +	Intensity Score 2

COMMENT:

The Allred score for estrogen and progesterone receptors is calculated by adding the sum of the proportion score (0 = no staining, 1 = <1% of cells staining, 2 = 1 - 10% of cells staining, 3 = 11-30% of cells staining, 4 = 31-60% of cells staining, 5 = >60% of cells staining) to the intensity score (1 = weak intensity of staining, 2 = intermediate intensity of staining, 3 = strong intensity of staining), with a scoring range from 0 to 8.

ER/PR positive is defined as an Allred score of >2 and ER/PR negative is defined as an Allred score of less than or equal to 2.

METHODOLOGY:

Tissue was fixed in 10% neutral buffered formalin for no less than 8 and no longer than 24 hours. Immunohistochemistry was performed using the mouse anti-human ER (ER 1D5, 1:100) and PR (PGR 136, 1:100) provided by Dako (following the manufacturer s instructions. This assay was not modified. Interpretation of the ER/PR immunohistochemical stain is guided by published results in the medical literature, information provided by the reagent manufacturer and by internal review of staining performance.

PathVysion HER-2 DNA Probe Kit

Case No

Analytical Interpretation of Results: HER-2 NOT AMPLIFIED

Clinical Interpretation of results

Amplification of the HER-2 gene was evaluated with interphase fluorescence in-situ hybridization (FISH) on formalin-fixed paraffin embedded tissue sections using a chromosome 17 centromeric probe and a HER-2 probe that spans the entire HER-2 gene in the by Dr. A majority of tumors cells displayed extensive polysomy 17 with 4 to 6 chromosome 17 signals and 2 to 3 HER-2 signals, with a HER-2/CEP 17 Ratio <=2.0, consistent with no amplification of the HER2/neu gene.

Block used D11 Source of case:

Tissue fixation formalin-fixed tissue Outside Case No: NA

Tissue source breast Results interpreted: yes

HER2/CEP17 ratio: 1.29

This ratio is derived by dividing the total number of LSI HER-2/neu signals by the total number of CEP17 signals in at least 20 interphase nuclei with nonoverlapping nuclei in the neoplastic mammary epithelial cells. Cells with no signals or with signals of only one color are disregarded.

Method of ratio enumeration: manual count

Limitations

The Vysis PathVysion Kit is not intended for use to screen for or diagnose breast cancer. It is intended to be used as an adjunct to other prognostic factors currently used to predict disease-free and overall survival in stage II, node-positive breast cancer patients. In making decisions regarding adjuvant CAF treatment, all other available clinical information should also be taken into consideration, such as tumor size, number of involved lymph nodes, and steroid receptor status. No treatment decision for stage II, node-positive breast cancer patients should be based on HER-2/neu gene amplification status alone.

Overview of this test

FDA APPROVED REAGENT

PathVysion HER-2 DNA Probe Kit is FDA approved for selection of patients for whom Herceptin® therapy is being considered. These tests were performed in the

under the direction of Dr. The results of these studies should always be interpreted in the context of the clinical, morphological, and immunophenotypic diagnosis.

Gross Dictation: Pathologist,
Microscopic/Diagnostic Dictation: Patholoaist
Final Review: Patholoaist
Final: Pathologist,
Addendum: Pathologist,
Addendum Final: Patholoaist.
Addendum: Pathologist,
Addendum Final: Pathol[illegible]

Criteria	Yes	No
Diagnosis Discrepancy		✓
Primary Tumor Site Discrepancy		✓
HIPAA Discrepancy		✓
Prior Malignancy History		✓
Dual/Synchronous Primary Noted		✓
Case is (circle): QUALIFIED / DISQUALIFIED		
Reviewer Initials [illegible] Date Reviewed: [illegible]		

Carcinoma, infiltrating duct, NOS triple negative
8500/3 12/8/10
lw

Path Site Code: breast, upper outer quadrant C50.4
CQCF Site: breast, NOS C50.9

TSS: .

UUID:734305A3-5EC4-4DE4-B263-91A49082F146
TCGA-E2-A14X-01A-PR Redacted

SPECIMENS:
A. RIGHT BREAST WLE NEEDLE LOCALIZATION
B. ADDITIONAL ANTERIOR INFERIOR MARGIN
C. ADDITIONAL SUPERIOR MARGIN
D. SENTINEL LYMPH NODE #1
E. SENTINEL LYMPH NODE #2
F. RIGHT AXILLARY CONTENTS LEVELS 1 & 2

SPECIMEN(S):
A. RIGHT BREAST WLE NEEDLE LOCALIZATION
B. ADDITIONAL ANTERIOR INFERIOR MARGIN
C. ADDITIONAL SUPERIOR MARGIN
D. SENTINEL LYMPH NODE #1
E. SENTINEL LYMPH NODE #2
F. RIGHT AXILLARY CONTENTS LEVELS 1 & 2

GROSS DESCRIPTION:
A. RIGHT BREAST WLE NEEDLE LOCALIZATION
Received fresh labeled with the patients identification and "Right Breast WLE needle localization" is an oriented (Single-Anterior, Double-Lateral, Triple-Superior and Quadruple-Inferior) 59g, 8.5 x 8.5 x 2.5cm needle localized lumpectomy with 2 radiographs. Ink code: Anterior-Yellow, Posterior-Black, Superior-Blue, Inferior-Orange, Medial-Green, Lateral-Yellow. Specimen serially sectioned from medial to lateral into 7 slices revealing a 2.5 x 1.5 x 1.5cm tan white firm well circumscribed mass abutting the anterior and posterior margins in slices 3-5. A portion of the specimen is submitted for tissue procurement. Representative sections are submitted.
A1-A3: medial margin slice 1
A4: superior margin slice 2
A5-A6: anterior margin slice 3
A7-A8: deep margin slice 3
A9-A11: anterior margin slice 3
A12-A14: deep margin with mass in A13 slice 3
A15: superior margin slice 4
A16: mass with anterior/deep margin slice 4
A17-A18: mass with anterior margin slice 4
A19-A20: mass with deep margin slice 4
A21: superior margin slice 5
A22-A23: mass with anterior/deep margin slice 5
A24: inferior margin slice 5
A25: area next to mass with anterior/deep margin slice 6
A26: lateral margin slice 7
B. ADDITIONAL ANTERIOR INFERIOR MARGIN
Received fresh labeled with the patient's identification and "Additional Anterior/Inferior margin" is an oriented (Single-Anterior, Double-Inferior) 19g, 5 x 5 x 2.5cm fragment of fibrofatty tissue. Final Anterior margin is inked Yellow and the final Inferior margin is inked Orange. Serial sectioning reveals no discrete lesions. Toto B1-B14.
C. ADDITIONAL SUPERIOR MARGIN
Received fresh labeled with the patient's identification and "Additional Superior margin" is an oriented (Single-Anterior, Double-Inferior) 10g, 3 x 3 x 2cm fragment of fibrofatty tissue. Final margin is inked Black. Serial sectioning reveals no discrete lesions. Toto C1-C7.
D. SENTINEL LYMPH NODE #1
Received fresh labeled with the patient's identification and "SLN #1" are two possible lymph nodes 0.8 x 0.8 x 0.5cm and 0.5 x 0.3 x 0.2cm. A touch prep is taken and the larger lymph node is submitted in FSD. The smaller possible lymph node is submitted in D2.
E. SENTINEL LYMPH NODE #2 (CLUMP OF FREE NODES)
Received fresh labeled with the patient's identification and "SLN #2" are 3 tan pink lymph nodes ranging from 1.4 x 0.9 x 0.8cm to 1.4 x 0.8 x 0.6cm. Toto FSE1, FSE2 and FSE3.
F. RIGHT AXILLARY CONTENTS LEVELS 1 & 2
Received in formalin are multiple tan pink soft tissue fragments aggregating to 10 x 10 x 4cm. Dissection reveals multiple lymph nodes. Entirely submitted:
F1: 5 lymph nodes
F2: 5 lymph nodes
F3: 1 lymph node
F4: 1 lymph node
F5: 1 lymph node
F6: 1 lymph node

F7-F8: 1 lymph node
F9-F10: 1 lymph node
F11-F12: 1 lymph node
F13-F20: axillary tissue

RESULTS:
SUMMARY OF IMMUNOHISTOCHEMISTRY/SPECIAL STAINS

Material: Block A1
Population: Tissue

Stain/Marker:	Result:	Comment:
CALP	Positive	In DCIS

Material: Block A12
Population: Tissue

Stain/Marker:	Result:	Comment:
CD31	Positive	

Material: Block A24
Population: Tissue

Stain/Marker:	Result:	Comment:
CD31	Positive	

Material: Block B7
Population: Tissue

Stain/Marker:	Result:	Comment:
ESTROGEN RECEPTOR	Positive	Heterogeneous staining consistent with UDH

The interpretation of the above immunohistochemistry stain or stains is guided by published results in the medical literature, provided package information from the manufacturer and by internal review of staining performance and assay validation within the Immunohistochemistry Laboratory. The use of one or more reagents in the above tests is regulated as an analyte specific reagent (ASR). These tests were developed and their performance characteristic determined by the Department of Pathology Laboratory. They have not been cleared or approved by the U.S. Food and Drug Administration. The FDA has determined that such clearance or approval is not necessary.

Special stains and/or immunohistochemical stains were performed with appropriately stained positive and negative controls.

DIAGNOSIS:

A. BREAST, RIGHT, NEEDLE LOCALIZATION WIDE LOCAL EXCISION:
- INVASIVE DUCTAL CARCINOMA, SBR GRADE 3, WITH MICROPAPILLARY FEATURES (SEE NOTE).
  - INVASIVE CARCINOMA MEASURES 2.5 CM.
  - INVASIVE CARCINOMA IS PRESENT AT THE ANTERIOR MARGIN AND IS 0.3 CM FROM THE POSTERIOR MARGIN.
  - EXTENSIVE LYMPHVASCULAR INVASION IS PRESENT.
- DUCTAL CARCINOMA IN SITU (DCIS), SOLID TYPE, NUCLEAR GRADE 3, WITH NECROSIS, MINOR COMPONENT.
  - DCIS IS FOCALLY WITHIN 0.4 CM OF THE MEDIAL MARGIN.
- PREVIOUS BIOPSY SITE CHANGES PRESENT.

NOTE: The additional anterior inferior margin (specimen B) is free of invasive carcinoma. Surgical correlation is recommended. CD31 stains show positive staining around tumor foci near superior and posterior margins consistent with tumor in lymphvascular channels.

B. BREAST, RIGHT, ADDITIONAL ANTERIOR INFERIOR MARGIN, EXCISION:
- FOCAL ATYPICAL DUCTAL HYPERPLASIA (ADH) AND USUAL DUCTAL HYPERPLASIA (UDH).

C. BREAST, RIGHT, ADDITIONAL SUPERIOR MARGIN, EXCISION:
- INVASIVE DUCTAL CARCINOMA, SBR GRADE 3.
  - TUMOR MEASURES 0.6 CM.
  - TUMOR IS WITHIN 0.2 CM OF THE NEW MARGIN.
  - LYMPHVASCULAR INVASION IS PRESENT.
- DCIS, SOLID TYPE, NUCLEAR GRADE 3, WITH NECROSIS, MINOR COMPONENT.

D. SENTINEL LYMPH NODE #1, RIGHT AXILLA, BIOPSY:
- ONE LYMPH NODE, NO TUMOR SEEN (0/1).

E. SENTINEL LYMPH NODE #2, RIGHT AXILLA, BIOPSY:
- METASTATIC CARCINOMA TO TWO OF THREE LYMPH NODES, LARGEST METASTASIS IS 0.7 CM, WITH NO EXTRANODAL EXTENSION (2/3).

F. AXILLARY CONTENTS, RIGHT, LEVELS 1 AND 2, DISSECTION:
- METASTATIC CARCINOMA TO 3 OF 17 LYMPH NODES, LARGEST METASTASIS IS 1.5 CM WITH EXTRANODAL EXTENSION (3/17).

SYNOPTIC REPORT - BREAST
Specimen Type: Excision
Needle Localization: Yes - For mass
Laterality: Right
Invasive Tumor: Present
Multifocality: Yes
WHO CLASSIFICATION
Invasive ductal carcinoma, NOS 8500/3
Tumor size: 2.5cm
Tumor Site: Upper outer quadrant
Margins: Negative
Distance from closest margin: Less than 0.2cm
superior
Tubular Score: 3
Nuclear Grade: 3
Mitotic Score: 3
Modified Scarff Bloom Richardson Grade: 3
Necrosis: Absent
Vascular/Lymphatic Invasion: Present
Extent: extensive
Lobular neoplasia: None
Lymph nodes: Sentinel lymph node and axillary dissection
Lymph node status: Positive 5 / 21 Extranodal extension
Non-neoplastic areas: fibroadenoma

DCIS present
Margins uninvolved by DCIS
DCIS Quantity: Estimate 5%
DCIS Type: Solid
DCIS Location: Associated with invasive tumor
Nuclear grade: High
Necrosis: Present

ER/PR/HER2 Results
ER: Negative
PR: Negative
HER2: Negative by IHC

Pathological staging (pTN): pT 2 N 2

SYNOPTIC REPORT - BREAST, ER/PR RESULTS
Specimen: Surgical Excision
Block Number: A22 and F12 (lymph node)

ER: Negative Allred Score: 0 = Proportion Score 0 + Intensity Score 0
PR: Negative Allred Score: 0 = Proportion Score 0 + Intensity Score 0

COMMENT:

The Allred score for estrogen and progesterone receptors is calculated by adding the sum of the proportion score (0 = no staining, 1 = <1% of cells staining, 2 = 1 - 10% of cells staining, 3 = 11-30% of cells staining, 4 = 31-60% of cells staining, 5 = >60% of cells staining) to the intensity score (1 = weak intensity of staining, 2 = intermediate intensity of staining, 3 = strong intensity of staining), with a scoring range from 0 to 8.

ER/PR positive is defined as an Allred score of >2 and ER/PR negative is defined as an Allred score of less than or equal to 2.

METHODOLOGY:

Tissue was fixed in 10% neutral buffered formalin for no less than 8 and no longer than 24 hours. Immunohistochemistry was performed using the mouse anti-human ER (ER 1D5, 1:100) and PR (PGR 136, 1:100) provided by Dako ,) following the manufacturer s instructions. This assay was not modified. Interpretation of the ER/PR immunohistochemical stain is guided by published results in the medical literature, information provided by the reagent manufacturer and by internal review of staining performance.

SYNOPTIC REPORT - BREAST HER-2 RESULTS
Specimen: Surgical Excision
Block Number: F12 (lymph node)

Interpretation: NEGATIVE
Intensity: 1+
% Tumor Staining: 5%
Fish Ordered: No

METHODOLOGY:

Tissue was fixed in 10% neutral buffered formalin for no less than 8 and no lonaer than 24 hours. Her2 analysis was performed using the FDA approved Dako HercepTest (TM) test kit using rabbit anti-human HER2. This assay was not modified. External kit-slides providea by the manufacturer (cell lines with high, low and negative HER2 protein expression) and in-house known HER2 amplified control tissue were evaluated along with the test tissue. Adequate, well preserved, clear-cut invasive carcinoma was identified for HER2 evaluation. Interpretation of the HER2 immunohistochemical stain is guided by published results in the medical literature, information provided by the reagent manufacturer and by internal review of staining performance.

This assay has been validated according to the 2007 joint recommendations and guidelines from ASCO and CAP and from the NCCN HER2 testing in Breast Cancer Task Force. The Pathology Department takes full responsibility for this test's performance.

CLINICAL HISTORY:

year old female with Abnormal MMG- Bx Right Breast at 9-10 o'clock IDC ER-. MRI showed additional abnormality 2.6cm posterior to this.

PRE-OPERATIVE DIAGNOSIS:

Right Breast Cancer

INTRAOPERATIVE CONSULTATION:
FSD-TPD: One lymph node negative for tumor.
FSE1-FSE2-FSE3: Positive for metastatic Adenocarcinoma.
Diagnoses called to Dr. at (D) and (E) by Dr.

Gross Dictation: Pathologist,
Microscopic/Diagnostic Dictation: Pathologist,
Final Review: Pathologist,
Final Review: Pathologist,
Final: Pathologist,

Criteria	Yes	No
Diagnosis Discrepancy		✓
Primary Tumor Site Discrepancy		✓
HIPAA Discrepancy		✓
Prior Malignancy History		✓
Dual/Synchronous Primary Noted		✓
Case is (circle): QUALIFIED / DISQUALIFIED		
Reviewer Initials [illegible] Date Reviewed: 11/23/10		

Carcinoma, infiltrating duct, NOS
8500/3 12/8/10

Path Site Code: breast, upper outer quadrant C50.4
CQCF Site: breast, NOS C50.9

UUID:957AC7B0-D613-4F97-BBBC-930EB95EE2E4
TCGA-E2-A14Y-01A-PR Redacted

TSS:

SPECIMENS:
- A. SLN #1 RIGHT AXILLA
- B. SLN #2 RIGHT AXILLA
- C. SLN #3 RIGHT AXILLA
- D. SLN #4 RIGHT AXILLA
- E. RIGHT BREAST
- F. ADDITIONAL AXILLARY TAIL RIGHT BREAST
- G. SCALP LESION

SPECIMEN(S):
- A. SLN #1 RIGHT AXILLA
- B. SLN #2 RIGHT AXILLA
- C. SLN #3 RIGHT AXILLA
- D. SLN #4 RIGHT AXILLA
- E. RIGHT BREAST
- F. ADDITIONAL AXILLARY TAIL RIGHT BREAST
- G. SCALP LESION

GROSS DESCRIPTION:

A. SLN #1 RIGHT AXILLA
Received fresh labeled with the patient's identification and "SLN #1 right axilla" is a tan pink lymph node 2.5 x 1.5 x 1.1cm. The specimen is sectioned and a touch prep is taken. Toto A1-A2.

B. SLN #2 RIGHT AXILLA
Received fresh labeled with the patient's identification and "SLN #2 right axilla" is a tan pink lymph node 0.8 x 0.5 x 0.4cm. The specimen is sectioned and a touch prep is taken. Toto B1.

C. SLN #3 RIGHT AXILLA
Received fresh labeled with the patient's identification and "SLN #3 right axilla" is a tan pink lymph node 0.7 x 0.5 x 0.2cm. The specimen is sectioned and a touch prep is taken. Toto C1.

D. SLN #4 RIGHT AXILLA
Received fresh labeled with the patient's identification and "SLN #4 right axilla" are two tan pink lymph nodes 1.1cm and 0.6cm in greatest dimension. The specimens are sectioned and a touch prep is taken.
D1: 1 lymph node
D2: 1 lymph node

E. RIGHT BREAST
Received fresh labeled with the patient's name and "right breast" is an oriented 466g, 17.5 x 16 x 2.5 cm mastectomy with a 7.5 x 3.2 cm skin ellipse and 1.5 cm everted nipple. Ink code: Anterior/superior-blue, anterior/inferior-orange, posterior-black. The specimen is serially sectioned into 12 slices from lateral to medial with nipple in slice 8 revealing a 2.7 x 2.5 x 2.5 cm white-tan firm infiltrating mass in the mid upper breast in slices 7-9 that is closest to the anterior margin at 0.5 cm. There is a clip located within the mass. Adjacent to the mass is a biopsy site with biopsy clip and surrounding granular tissue spanning ~ 4 cm in slices 4-7 in the upper outer quadrant. Tissue is procured. Representatively submitted:
E1: slice 7, mid anterior including bisected mass
E2: slice 7, mid posterior including bisected mass (clip)
E3: slice 7, fibrous tissue inferior to mass
E4: slice 8, margin deep to mass
E5: slice 8, mass
E6: slice 9, mass, UIQ
E7: slice 6, mid-superior
E8: slice 6, mid anterior (with clip)
E9: slice 5, mid superior
E10: slice 5, deep margin
E11: slice 4, upper anterior margin
E12: slice 4, lower anterior margin
E13: slice 3, superior mid
E14: slice 2, midsection
E15: slice 4, lower outer quadrant
E16: slice 5, lower outer quadrant
E17: slices 7-8, lower inner quadrant
E18: slices 9-10, lower inner quadrant
E19: nipple
E20: nipple and skin

F. ADDITIONAL AXILLARY TAIL RIGHT BREAST
Received in formalin labeled with the patient's identification and "additional axillary tail right breast" are multiple tan pink soft tissue fragments aggregating to 3 x 2 x 1cm. No lymph node is grossly identified. Toto F1-F3.
G. SCALP LESION
Received in formalin labeled with the patient's identification and "scalp lesion" is a tan white firm well circumscribed mass 1.4 x 1.3 x 1cm. The resection margin is inked black and the specimen is trisected. Toto G1.

DIAGNOSIS:

A. SENTINEL LYMPH NODE #1, RIGHT AXILLA, BIOPSY:
- ONE LYMPH NODE, NO TUMOR SEEN (0/1).

B. SENTINEL LYMPH NODE #2, RIGHT AXILLA, BIOPSY:
- ONE LYMPH NODE, NO TUMOR SEEN (0/1).

C. SENTINEL LYMPH NODE #3, RIGHT AXILLA, BIOPSY:
- ONE LYMPH NODE, NO TUMOR SEEN (0/1).

D. SENTINEL LYMPH NODE #4, RIGHT AXILLA, BIOPSY:
- TWO LYMPH NODES, NO TUMOR SEEN (0/2).

E. BREAST, RIGHT, MASTECTOMY:
- INVASIVE DUCTAL CARCINOMA, SBR GRADE 3, WITH FOCAL SQUAMOUS FEATURES AND NECROSIS.
  - TUMOR MEASURES 2.7 CM.
  - MARGINS, NO TUMOR SEEN.
- DUCTAL CARCINOMA IN SITU (DCIS), SOLID TYPE, NUCLEAR GRADE 3, WITH NECROSIS AND MICROCALCIFICATIONS, INVOLVING LOBULES.
- SKIN AND NIPPLE, NO TUMOR SEEN.

F. ADDITIONAL AXILLARY TAIL, RIGHT, EXCISION:
- FIBROADIPOSE TISSUE, NO TUMOR OR LYMPH NODES SEEN.

G. SCALP, LESION, EXCISION:
- PILAR CYST.

SYNOPTIC REPORT - BREAST
Specimen Type: Mastectomy
Needle Localization: No
Laterality: Right
Invasive Tumor: Present
Multifocality: No
WHO CLASSIFICATION
Invasive ductal carcinoma, NOS 8500/3
Tumor size: 2.7cm
Tumor Site: 12:00
Margins: Negative
Distance from closest margin: 0.5cm
anterior
Tubular Score: 3
Nuclear Grade: 3
Mitotic Score: 3
Modified Scarff Bloom Richardson Grade: 3
Necrosis: Present
Vascular/Lymphatic Invasion: None identified
Lobular neoplasia: None
Lymph nodes: Sentinel lymph node only
Lymph node status: Negative 0 / 5

DCIS present
Margins uninvolved by DCIS
DCIS Quantity: Estimate 15%
DCIS Type: Solid
Cribriform
DCIS Location: Both associated and separate from invasive tumor mass
Nuclear grade: High
Necrosis: Present
Location of CA++: DCIS

ER/PR/HER2 Results
ER: Positive
PR: Positive
HER2: Positive by FISH

Pathological staging (pTN): pT 2 N 0

CLINICAL HISTORY:
None provided

PRE-OPERATIVE DIAGNOSIS:
Right axilla

INTRAOPERATIVE CONSULTATION:
TPA-TPB-TPC-TPD: Negative for tumor on touch prep. Diagnosis called by Dr. to Dr. at (A-C) and (D).

Gross Dictation: Pathologist,
Microscopic/Diagnostic Dictation: Pathologist,
Final Review: Pathologist,
Final: Pathologist,

Criteria	Yes	No
Diagnosis Discrepancy		✓
Primary Tumor Site Discrepancy		✓
HIPAA Discrepancy		✓
Prior Malignancy History		✓
Dual/Synchronous Primary Noted		✓
Case is (circle): QUALIFIED / DISQUALIFIED		
Reviewer Initials [illegible] Date Reviewed: 11/23/10		

ICD-O-3
Carcinoma, infiltrating duct, NOS
8500/3 12/8/10
[illegible]
Site Code: breast, NOS C50.9

TSS

UUID:04FF2CF1-737B-4FD2-8A1D-F9B5488DE510
TCGA-E2-A15C-01A-PR
Redacted

SPECIMENS:
A. WLE RIGHT BREAST NEEDLE LOCALIZATION
B. SENTINEL L.N. #1 RIGHT AXILLA
C. SENTINEL L.N. #2
D. SENTINEL L.N. #3
E. SENTINEL L.N. #4

SPECIMEN(S):
A. WLE RIGHT BREAST NEEDLE LOCALIZATION
B. SENTINEL L.N. #1 RIGHT AXILLA
C. SENTINEL L.N. #2
D. SENTINEL L.N. #3
E. SENTINEL L.N. #4

GROSS DESCRIPTION:
A. WLE RIGHT BREAST NEEDLE LOCALIZATION
Received fresh labeled with the patient's identification and "WLE right breast needle localization" is a previously inked 70g, 8 x 5 x 4cm needle localized lumpectomy with radiograph. Ink code: anterior-yellow, posterior-black, superior-blue, inferior-orange, medial-green, lateral-red. Specimen is serially sectioned from lateral to medial into 9 slices revealing a tan white firm stellate 1.9 x 1.6 x 1.5cm mass, 0.7cm from the closest posterior margin in slices 3-6. Representatively submitted:
A1-A2: lateral margin slice 1
A3: next to mass slice 2
A4-A8: slice 3 with mass – deep margin A4
A9: mass with deep margin slice 4
A10: inferior margin slice 4
A11-A12: slice 5
A13: mass with deep margin slice 6
A14: slice 7
A15: slice 8
A16: medial margin slice 9
B. SLN #1 RIGHT AXILLA
Received fresh is a tan pink lymph node 0.5 x 0.5 x 0.5cm. The specimen is sectioned and a touch prep is taken. Toto B1.
C. SLN #2 RIGHT AXILLA
Received fresh is a tan pink lymph node 0.8 x 0.6 x 0.6cm. The specimen is sectioned and a touch prep is taken. Toto C1.
D. SLN #3 RIGHT AXILLA
Received fresh is a tan pink lymph node 0.9 x 0.5 x 0.5cm. The specimen is sectioned and a touch prep is taken. Toto D1.
E. SLN #4 RIGHT AXILLA
Received fresh is a tan pink lymph node 0.6 x 0.6 x 0.5cm. The specimen is sectioned and a touch prep is taken. Toto E1.

DIAGNOSIS:
A. BREAST, RIGHT, WIDE LOCAL EXCISION:
- INVASIVE DUCTAL CARCINOMA, SBR GRADE 2, MEASURING 1.7-CM, INVOLVING SKELETAL MUSCLE
- INTERMEDIATE NUCLEAR GRADE, DUCTAL CARCINOMA IN SITU, CRIBRIFORM TYPE
- SURGICAL RESECTION MARGINS NEGATIVE FOR TUMOR
- BIOPSY SITE CHANGES WITH FIBROSIS AND GRANULATION TISSUE
- SEE SYNOPTIC REPORT.

B. LYMPH NODE, SENTINEL #1, RIGHT AXILLA, EXCISION:
- ONE LYMPH NODE, NEGATIVE FOR METASTASES (0/1).

C. LYMPH NODE, SENTINEL #2, RIGHT AXILLA, EXCISION:
- ONE LYMPH NODE, NEGATIVE FOR METASTASES (0/1).

D. LYMPH NODE, SENTINEL #3, RIGHT AXILLA, EXCISION:
- ONE LYMPH NODE, NEGATIVE FOR METASTASES (0/1).

E. LYMPH NODE, SENTINEL #4, RIGHT AXILLA, EXCISION:
- ONE LYMPH NODE, NEGATIVE FOR METASTASES (0/1).

SYNOPTIC REPORT - BREAST
Specimen Type: Excision
Needle Localization: Yes - For mass
Laterality: Right
Invasive Tumor: Present
Multifocality: No
WHO CLASSIFICATION
Invasive ductal carcinoma, NOS 8500/3
Tumor size: 1.7cm
Tumor Site: Not specified
Margins: Negative
Distance from closest margin: 0.3cm
inferior
Tubular Score: 2
Nuclear Grade: 2
Mitotic Score: 2
Modified Scarff Bloom Richardson Grade: 2
Necrosis: Absent
Vascular/Lymphatic Invasion: None identified
Lobular neoplasia: None
Lymph nodes: Sentinel lymph node only
Lymph node status: Negative 0 / 4

DCIS present
Margins uninvolved by DCIS
DCIS Quantity: Estimate 1%
DCIS Type: Cribriform
DCIS Location: Associated with invasive tumor
Nuclear grade: Intermediate
Necrosis: Absent

ER/PR/HER2 Results
ER: Positive
PR: Positive
HER2: Pending
Performed on Case:

Pathological staging (pTN): pT 1c N 0

CLINICAL HISTORY:
None provided.

PRE-OPERATIVE DIAGNOSIS:
None provided.

INTRAOPERATIVE CONSULTATION:
A. GROSS EXAMINATION: WLE right breast- 1.9cm mass 0.7cm from closest deep margin. Diagnosis called to Dr. at by Dr.
TPB-TPC-TPD-TPE: Negative for carcinoma. Diagnosis called to Dr. at by Dr.

ADDENDUM:
SYNOPTIC REPORT - BREAST HER-2 RESULTS
Specimen: Surgical Excision
Block Number: A9

Interpretation: EQUIVOCAL
Intensity: 2+
% Tumor Staining: 20%
Fish Ordered: Yes , on Date

METHODOLOGY:
Tissue was fixed in 10% neutral buffered formalin for no less than 8 and no longer than 24 hours. Her2 analysis was performed using the FDA approved Dako HercepTest (TM) test kit) using rabbit anti-human HER2. This assay was not modified. External kit-slides provided by the manufacturer (cell lines with high, low and negative HER2 protein expression) and in-house known HER2 amplified control tissue were evaluated along

with the test tissue. Adequate, well preserved, clear-cut invasive carcinoma was identified for HER2 evaluation. Interpretation of the HER2 immunohistochemical stain is guided by published results in the medical literature, information provided by the reagent manufacturer and by internal review of staining performance.

This assay has been validated according to the 2007 joint recommendations and guidelines from ASCO and CAP and from the NCCN HER2 testing in Breast Cancer Task Force. The Pathology Department takes full responsibility for this test's performance.

PathVysion HER-2 DNA Probe Kit
Case No
Analytical Interpretation of Results: HER-2 NOT AMPLIFIED
Clinical Interpretation of results
Amplification of the HER-2 gene was evaluated with interphase fluorescence in-situ hybridization (FISH) on formalin-fixed paraffin embedded tissue sections using a chromosome 17 centromeric probe and a HER-2 probe that spans the entire HER-2 gene in the Pathology Core Facility by Dr. A majority of tumors cells displayed 2 chromosome 17 signals and 2 HER-2 signals, with a HER-2/CEP 17 Ratio </=2.0, consistent with no amplification of the HER2/neu gene.
Block used A9 Source of case:
Tissue fixation formalin-fixed tissue Outside Case No: NA
Tissue source breast Results interpreted: yes
HER2/CEP17 ratio: 1.02
This ratio is derived by dividing the total number of LSI HER-2/neu signals by the total number of CEP17 signals in at least 20 interphase nuclei with nonoverlapping nuclei in the neoplastic mammary epithelial cells. Cells with no signals or with signals of only one color are disregarded.
Method of ratio enumeration: manual count
Limitations
The Vysis PathVysion Kit is not intended for use to screen for or diagnose breast cancer. It is intended to be used as an adjunct to other prognostic factors currently used to predict disease-free and overall survival in stage II, node-positive breast cancer patients. In making decisions regarding adjuvant CAF treatment, all other available clinical information should also be taken into consideration, such as tumor size, number of involved lymph nodes, and steroid receptor status. No treatment decision for stage II, node-positive breast cancer patients should be based on HER-2/neu gene amplification status alone.
Overview of this test
FDA APPROVED REAGENT
PathVysion HER-2 DNA Probe Kit is FDA approved for selection of patients for whom Herceptin® therapy is being considered. These tests were performed in the under the direction of Dr. The results of these studies should always be interpreted in the context of the clinical, morphological, and immunophenotypic diagnosis.
ONCOTYPE DX BREAST CANCER ASSAY

RESULTS: Recurrence Score: 19
CLINICAL EXPERIENCE: Patients with a recurrence score of: 19 in the clinical validation study had an average rate of Distant Recurrence at 10 years of 12%

ER Score: 10.2 Positive
PR Score: 8.8 Positive
Her2 Score: 8.9 Negative

Interpretation:
ER Negative < 6.5 Positive >= 6.5
PR Negative < 5.5 Positive >= 5.5
Her2 Negative <10.7 Positive >=11.5 Equivocal = 10.7 - 11.4

See separate report for further information.
Test performed at:

Gross Dictation:
Microscopic/Diagnostic Dictation: Pathologist,
Final Review: Pathologist,
Final: Pathologist,
Addendum: Pathologist,
Addendum Final: Pathologist,
Addendum: Pathologist,
Addendum Final: Pathologist,
Addendum: Pathologist, 1
Addendum Final: Pathologist,

Criteria	Yes	No
Diagnosis Discrepancy		/
Primary Tumor Site Discrepancy		/
HIPAA Discrepancy		/
Prior Malignancy History		/
Dual/Synchronous Primary Noted		
Case is (circle): QUALIFIED / DISQUALIFIED		
Reviewer Initials: [illegible] Date Reviewed: [illegible]		

ICD-0-3
Carcinoma, infiltrating duct, NOS
8500/3 12/8/10
[illegible]
Site Code: breast, NOS C50.9

TSS:

UUID:1FE4EB4C-FEF8-486A-B39D-6F9F023FDAC4
TCGA-E2-A15D-01A-PR Redacted

SPECIMENS:
A. RIGHT BREAST CANCER
B. SENTINEL L.N. #1 RIGHT AXILLA
C. SENTINEL L.N. #2 RIGHT AXIL;LA
D. SENTINEL L.N. #3 RIGHT AXILLA
E. SENTINEL L.N. #4 RIGHT AXILLA

SPECIMEN(S):
A. RIGHT BREAST CANCER
B. SENTINEL L.N. #1 RIGHT AXILLA
C. SENTINEL L.N. #2 RIGHT AXIL;LA
D. SENTINEL L.N. #3 RIGHT AXILLA
E. SENTINEL L.N. #4 RIGHT AXILLA

GROSS DESCRIPTION:
A. RIGHT BREAST CANCER
Received fresh labeled with the patient's identification and "right breast needle localization" is a previously inked 53g, 6 x 5 x 4cm needle localized lumpectomy with radiograph. Ink code: anterior-yellow, posterior-black, superior-blue, inferior-orange, medial-green, lateral-red. Specimen is serially sectioned from lateral to medial into 8 slices revealing a 2.5 x 2.3 x 1.5cm tan white firm well circumscribed mass, 0.6cm from the closest inferior margin in slices 2-7. A surgical clip is identified in slice 5. A portion of the specimen is submitted for tissue procurement. Representatively submitted:
A1-A2: lateral margin slice 1
A3-A5: slice 2
A6-A8: slice 3
A9-A15: slice 4
A16: slice 5 with clip ID
A17-A18: slice 6
A19-A20: slice 7
A21: medial margin
B. SENTINEL L.N. #1 RIGHT AXILLA
Received fresh is a tan pink lymph node 1.2 x 1 x 1cm. The specimen is sectioned and a touch prep is taken. Toto B1.
C. SENTINEL L.N. #2 RIGHT AXILLA
Received fresh is a tan pink lymph node 0.8 x 0.6 x 0.5cm. The specimen is bisected and a touch prep is taken. Toto C1.
D. SENTINEL L.N. #3 RIGHT AXILLA
Received fresh is a tan pink lymph node 1.2 x 1 x 0.8cm. The specimen is bisected and a touch prep is taken. Toto D1.
E. SENTINEL L.N. #4 RIGHT AXILLA
Received fresh is a tan pink lymph node 1.4 x 1 x 0.6cm. The specimen is bisected and a touch prep is taken. Toto E1.

DIAGNOSIS:
A. BREAST, RIGHT, WIDE LOCAL EXCISION:
- INVASIVE DUCTAL CARCINOMA, SBR GRADE 1, MEASURING 2.2-CM
- INTERMEDIATE NUCLEAR GRADE, DUCTAL CARCINOMA IN SITU, CRIBRIFORM TYPE WITH CENTRAL NECROSIS
- SURGICAL RESECTION MARGINS NEGATIVE FOR TUMOR
- DCIS PRESENT WITHIN 0.15-CM FROM INFERIOR SURGICAL RESECTION MARGIN
- BIOPSY SITE CHANGES WITH FIBROSIS AND GRANULATION TISSUE
- SEE SYNOPTIC REPORT.

B. LYMPH NODE, SENTINEL #1, RIGHT AXILLA, EXCISION:
- ONE LYMPH NODE, NEGATIVE FOR METASTASES (0/1).

C. LYMPH NODE, SENTINEL #2, RIGHT AXILLA, EXCISION:
- ONE LYMPH NODE, NEGATIVE FOR METASTASES (0/1).

D. LYMPH NODE, SENTINEL #3, RIGHT AXILLA, EXCISION:
- ONE LYMPH NODE, NEGATIVE FOR METASTASES (0/1).

E. LYMPH NODE, SENTINEL #4, RIGHT AXILLA, EXCISION:

- ONE LYMPH NODE, NEGATIVE FOR METASTASES (0/1).

SYNOPTIC REPORT - BREAST
Specimen Type: Excision
Needle Localization: Yes - For mass
Laterality: Right
Invasive Tumor: Present
Multifocality: No
WHO CLASSIFICATION
Invasive ductal carcinoma, NOS 8500/3
Tumor size: 2.2cm
Tumor Site: Not specified
Margins: Negative
Distance from closest margin: 0.4cm
inferior
Tubular Score: 2
Nuclear Grade: 2
Mitotic Score: 1
Modified Scarff Bloom Richardson Grade: 1
Necrosis: Absent
Vascular/Lymphatic Invasion: None identified
Lobular neoplasia: None
Lymph nodes: Sentinel lymph node only
Lymph node status: Negative 0 / 4

DCIS present
Margins uninvolved by DCIS 0.15-cm from inferior
DCIS Quantity: Estimate 30%
DCIS Type: Cribriform
DCIS Location: Associated with invasive tumor
Nuclear grade: Intermediate
Necrosis: Present

ER/PR/HER2 Results
ER: Positive
PR: Positive
HER2: Negative by FISH
Performed on Case:

Pathological staging (pTN): pT 2 N 0

CLINICAL HISTORY:
None provided.

PRE-OPERATIVE DIAGNOSIS:
Right breast cancer.

INTRAOPERATIVE CONSULTATION:
A. GROSS EXAMINATION: Right breast- mass is 2.5cm and 0.6cm from the closest inferior margin. Diagnosis called to Dr. at by Dr.
TPB-TPC-TPD-TPE: SLN #1-4: Negative for carcinoma. Diagnosis called to Dr. at by Dr.

ADDENDUM:
ONCOTYPE DX BREAST CANCER ASSAY

RESULTS: Recurrence Score: 17
CLINICAL EXPERIENCE: Patients with a recurrence score of: 17 in the clinical validation study had an average rate of Distant Recurrence at 10 years of 11%

ER Score: 9.8 Positive
PR Score: 8.4 Positive
Her2 Score: 9.5 Negative

Interpretation:
ER Negative < 6.5 Positive >= 6.5
PR Negative < 5.5 Positive >= 5.5

Her2 Negative <10.7 Positive >=11.5 Equivocal = 10.7 - 11.4

See separate report for further information.
Test performed at:

Gross Dictation:
Microscopic/Diagnostic Dictation: Pathologist,
Final Review: Pathologist
Final: Pathologist,
Addendum: Pathologist,
Addendum Final: Pathologist,

riteria	Yes	No
iagnosis Discrepancy		/
rimary Tumor Site Discrepancy		/
IIPAA Discrepancy		/
rior Malignancy History		/
ual/Synchronous Primary Noted		/
ase Is (Circle): QUALIFIED / [illegible]		
eviewer Initials [illegible] Date Reviewed: [illegible]		

ICD-0-3
Carcinoma, infiltrating duct, NOS
8500/3 12/8/10
lw

Path Site Code: breast, upper outer quadrant C50.4
CQCF Site: breast, NOS C50.9

TSS:

UUID:352BF6A2-974B-440E-BB0B-DCD79874DAC3
TCGA-E2-A153-01A-PR Redacted

SPECIMENS:
A. SENTINEL LYMPH NODE #1 LEFT AXILLA
B. SENTINEL LYMPH NODE #2 LEFT AXILLA
C. SENTINEL LYMPH NODE #3 LEFT AXILLA
D. SENTINEL LYMPH NODE #4 LEFT AXILLA
E. LEFT BREAST

SPECIMEN(S):
A. SENTINEL LYMPH NODE #1 LEFT AXILLA
B. SENTINEL LYMPH NODE #2 LEFT AXILLA
C. SENTINEL LYMPH NODE #3 LEFT AXILLA
D. SENTINEL LYMPH NODE #4 LEFT AXILLA
E. LEFT BREAST

INTRAOPERATIVE CONSULTATION DIAGNOSIS:
TPA/TPB/TPC/TPD1/TPD2/TPD3/TPD4-SLN #1, #2, #3, #4: No tumor seen.
Diagnoses called a Dr to Dr. at (A, B, C, D).

GROSS DESCRIPTION:
A. SENTINEL LYMPH NODE #1 LEFT AXILLA
Received fresh labeled with the patient's identification and "SLN #1" is a 1.1 x 0.7 x 0.5-cm lymph node; sectioned, a touch prep is performed, and lymph node is submitted entirely in cassette A1.
B. SENTINEL LYMPH NODE #2 LEFT AXILLA
Received fresh labeled with the patient's identification and "SLN #2" is a 0.8 x 0.6 x 0.4 cm lymph node; sectioned, touch prep is performed, submitted entirely in cassette B1.
C. SENTINEL LYMPH NODE #3 LEFT AXILLA
Received fresh labeled with the patient's identification and "SLN #3" is a 1 x 0.8 x 0.6 cm lymph node; sectioned, a touch prep is performed, and lymph node is submitted entirely in cassette C1.
D. SENTINEL LYMPH NODE #4 LEFT AXILLA
Received fresh labeled with the patient's identification and "SLN #4" are 4 lymph nodes, 0.4 x 0.4 x 0 .2 cm, 0.3 x 0.3 x 0.2 cm, 0.3 x 0.2 x 0 .2 cm, and 0.3 x 0.2 x 0.2 cm; 4 touch preps are performed, and lymph nodes are submitted respectively and entirely in cassettes D1-D4.
E. LEFT BREAST
Received fresh labeled with the patient's identification and "left breast" is an oriented 574 g, 24 x 19 x 4.5 cm mastectomy with 10.5 x 4 cm skin ellipse and 1.3-cm everted nipple. Ink code: Anterior/superior-blue, anterior/inferior-orange, and posterior-black. Specimen is serially sectioned from lateral to medial into 10 slices with nipple in slice 7 revealing:
1) 3.5 x 3.4 x 2.2 cm area of hemorrhage and containing a biopsy site in the lower outer quadrant to the lower mid section in slices 4-6 at is closest to the anterior margin at 2.3 cm
2) 5.5 cm superior to the biopsy site, in slice 6, is a 1.5 x 1.4 x 1.4 cm irregularly shaped firm tan mass in the upper inner quadrant that is closest to the anterior margin at 0.2 cm.
Within the axillary region is a 1 cm firm tan lymph node. Representatively submitted:
E1: slice 4, LOQ granular region lateral to biopsy site
E2: slice 5, most lateral aspect of area of hemorrhage and biopsy site
E3: slice 6, most inferior aspect of biopsy site extending to the anterior margin/skin
E4: slice 6, most inferior aspect of biopsy site extending to the posterior margin
E5: slice 6, mid anterior region of biopsy site
E6: slice 6, mid posterior region of biopsy site
E7-E8: slice 6, fibrous tissue connecting biopsy site of lesion
E9-E11: slice 6, upper inner quadrant mass including posterior margin (trisected)
E12: slice 7, fibrous tissue medial to biopsy site
E13: slice 7, fibrous tissue medial to mass
E14: slice 10, LIQ
E15: slice 9, UIQ
E16: slice 3, UOQ
E17: slice 2, LOQ
E18-E19: nipple, perpendicular sections
E20: skin and bisected lymph node

DIAGNOSIS:
A. LYMPH NODE, SENTINEL #1, LEFT AXILLA, EXCISION:
- ONE LYMPH NODE, NEGATIVE FOR METASTASES (0/1).

B. LYMPH NODE, SENTINEL #2, LEFT AXILLA, EXCISION:
- ONE LYMPH NODE, NEGATIVE FOR METASTASES (0/1).

C. LYMPH NODE, SENTINEL #3, LEFT AXILLA, EXCISION:
- ONE LYMPH NODE, NEGATIVE FOR METASTASES (0/1).

D. LYMPH NODE, SENTINEL #4, LEFT AXILLA, EXCISION:
- FOUR LYMPH NODES, NEGATIVE FOR METASTASES (0/4).

E. BREAST, LEFT, MASTECTOMY:
- INVASIVE DUCTAL CARCINOMA, SBR GRADE 1, MEASURING 1.7-CM
- INTERMEDIATE NUCLEAR GRADE, DUCTAL CARCINOMA IN SITU, SOLID, CRIBRIFORM, MICROPAPILLARY AND PAPILLARY TYPES WITH CENTRAL NECROSIS, MICROCALCIFICATIONS AND LOBULAR EXTENSION
- SURGICAL RESECTION MARGINS NEGATIVE FOR TUMOR
- FIVE LYMPH NODES, NEGATIVE FOR METASTASES (0/5)
- LOW GRADE AND HIGH GRADE (PLEOMORPHIC) LOBULAR CARCINOMA IN SITU
- TWO BIOPSY SITES WITH FIBROSIS ABD GRANULATION TISSUE
- SEE SYNOPTIC REPORT AND SEE NOTE.

NOTE: Two lesions are grossly identified, both located in slice #6 (outer quadrants/central). The upper lesion is DCIS extending from slice #4 to slice #6, measuring approximately 3-cm. The second lesion is located centrally showing invasive ductal carcinoma, measuring 1.7-cm. The tissue sections between two lesions (slides #7 and #8) show no invasive or in situ ductal carcinoma.

SYNOPTIC REPORT - BREAST
Specimen Type: Mastectomy
Needle Localization: No
Laterality: Left
Invasive Tumor: Present
Multifocality: No
WHO CLASSIFICATION
Invasive ductal carcinoma, NOS 8500/3
Tumor size: 1.7cm
Tumor Site: Upper outer quadrant
Central
Margins: Negative
Tubular Score: 2
Nuclear Grade: 2
Mitotic Score: 1
Modified Scarff Bloom Richardson Grade: 1
Necrosis: Absent
Vascular/Lymphatic Invasion: None identified
Lobular neoplasia: LCIS
Lymph nodes: Sentinel lymph node only
Lymph node status: Negative 0 / 12

DCIS present
Margins uninvolved by DCIS
DCIS Quantity: Estimate 60%
DCIS Type: Solid
Cribriform
Micropapillary
Papillary
DCIS Location: Both associated and separate from invasive tumor mass
Nuclear grade: Intermediate
Necrosis: Present
Location of CA++: DCIS

ER/PR/HER2 Results
ER: Positive
PR: Positive
HER2: Negative by FISH
Performed on Case:

Pathological staging (pTN): pT 1c N 0

CLINICAL HISTORY:

Left breast invasive cancer with extensive surrounding DCIS

PRE-OPERATIVE DIAGNOSIS:

Left breast cancer

ADDENDUM:

ONCOTYPE DX BREAST CANCER ASSAY

RESULTS: Recurrence Score: 20

CLINICAL EXPERIENCE: Patients with a recurrence score of: 20 in the clinical validation study had an average rate of Distant Recurrence at 10 years of 13%

ER Score: 8.9 Positive
PR Score: 7.6 Positive
Her2 Score: 9.9 Negative

Interpretation:

ER Negative < 6.5 Positive >= 6.5
PR Negative < 5.5 Positive >= 5.5
Her2 Negative <10.7 Positive >=11.5 Equivocal = 10.7 - 11.4

See separate (report for further information.
Test performed at:

Microscopic/Diagnostic Dictation: Pathologist,
Final Review: Pathologist.
Final: Pathologist,
Addendum: Pathologist,
Addendum Final: Pathologist, (

Criteria	Yes	No
Diagnosis Discrepancy		/
Primary Tumor Site Discrepancy		/
HIPAA Discrepancy		/
Prior Malignancy History		/
Dual/Synchronous Primary Noted		/
Case is (circle): QUALIFIED / DISQUALIFIED		
Reviewer Initials [illegible] Date Reviewed: [illegible]		

ICD-0-3
Carcinoma, infiltrating duct, NOS
8500/3 12/8/10
Site Code: breast, NOS C50.9

UUID:2BAC50DA-016F-4B5C-B8D1-DE75377EF0C7
TCGA-E2-A15K-01A-PR Redacted

TSS:

SPECIMENS:
A. RIGHT BREAST LUMPECTOMY
B. RIGHT AXILLARY CONTENTS LEVELS 1,2
C. ADDITIONAL RIGHT AXILLARY TISSUE

SPECIMEN(S):
A. RIGHT BREAST LUMPECTOMY
B. RIGHT AXILLARY CONTENTS LEVELS 1,2
C. ADDITIONAL RIGHT AXILLARY TISSUE

GROSS DESCRIPTION:
A. RIGHT BREAST LUMPECTOMY
Received fresh labeled with the patient's identification and "right breast lumpectomy" is a previously inked, oriented 97g, 8 x 6.5 x 4.5cm lumpectomy. Ink code: anterior-yellow, posterior-black, superior-blue, inferior-orange, medial-green, lateral-red. Specimen is serially sectioned from lateral to medial into 8 slices revealing a 2 x 1.8 x 1.6cm tan white stellate mass, 0.6cm from the closest inferior margin in slices 2-5cm. A portion of the specimen is submitted for tissue procurement. Representatively submitted:
A1: lateral margin slice 1
A2-A4: slice 2 with mass in A4
A5-A7: slice 3 with mass in A6
A8-A11: slice 4
A12-A15: slice 5 with mass in A15
A16-A17: slice 6
A18-A19: slice 7
A20: medial margin slice 8
B. RIGHT AXILLARY CONTENTS LEVELS 1,2
Received fresh is a tan pink soft tissue fragment 15 x 12 x 4cm. Dissection reveals 14 lymph nodes ranging from 0.3 x 0.3 x 0.2cm to 5 x 3.2 x 1.5cm. The largest lymph node is sectioned to reveal a firm homogenous white cut surface.
B1: 5 lymph nodes
B2: 4 lymph nodes
B3: 2 lymph nodes
B4: 1 lymph node
B5: 1 lymph node
B6: 1 lymph node
B7: 1 lymph node
B8: 1 lymph node
B9-B10: 1 lymph node
B11-B12: 1 lymph node
B13-B16: representative sections of 1 lymph node
C. ADDITIONAL RIGHT AXILLARY TISSUE
Received fresh is a tan pink soft tissue fragment 4.3 x 2.7 x 2cm. Dissection reveals a possible necrotic lymph node 2.8 x 1.3 x 1cm. Representatively submitted in C1-C4.

DIAGNOSIS:
A. BREAST, RIGHT, WIDE LOCAL EXCISION:
- INVASIVE DUCTAL CARCINOMA, SBR GRADE 3, MEASURING 2.4-CM
- INTERMEDIATE NUCLEAR GRADE, DUCTAL CARCINOMA IN SITU, SOLID TYPE
- INVASIVE TUMOR INVOLVES INFERIOR SURGICAL RESECTION MARGIN AND PRESENT WITHIN 1 MM FROM MEDIAL SURGICAL RESECTION MARGIN
- BIOPSY SITE CHANGES WITH FIBROSIS
- SEE SYNOPTIC REPORT.

B. LYMPH NODES, RIGHT, AXILLARY DISSECTION:
- METASTATIC CARCINOMA TO TWO OF SEVENTEEN LYMPH NODES (2/17), LARGEST MEASURING 2.5-CM, WITH EXTRANODAL EXTENSION.

C. SOFT TISSUE, ADDITIONAL WHITE AXILLARY, EXCISION:
- FIBROADIPOSE TISSUE WITH FAT NECROSIS, NO TUMOR SEEN.

SYNOPTIC REPORT - BREAST
Specimen Type: Excision

Needle Localization: No
Laterality: Right
Invasive Tumor: Present
Multifocality: No
WHO CLASSIFICATION
Invasive ductal carcinoma, NOS 8500/3
Tumor size: 2.4cm
Margins: Involved at inferior
Extent:: focal
Tubular Score: 3
Nuclear Grade: 2
Mitotic Score: 3
Modified Scarff Bloom Richardson Grade: 3
Necrosis: Absent
Vascular/Lymphatic Invasion: Present
Extent: focal
Lobular neoplasia: None
Lymph nodes: Axillary dissection
Lymph node status: Positive 2 / 17 Extranodal extension
Micrometastases: No

DCIS present
Margins uninvolved by DCIS
DCIS Quantity: Estimate 2%
DCIS Type: Solid
DCIS Location: Associated with invasive tumor
Nuclear grade: Intermediate
Necrosis: Absent

ER/PR/HER2 Results
ER: Positive
PR: Positive
HER2: Pending by FISH

Pathological staging (pTN): pT 2 N 1a
Pathological staging is based on the AJCC Cancer Staging Manual, 7th Edition

SYNOPTIC REPORT - BREAST, ER/PR RESULTS
Specimen: Surgical Excision
Block Number: A4

ER: Positive Allred Score: 8 = Proportion Score 5 + Intensity Score 3
PR: Positive Allred Score: 4 = Proportion Score 2 + Intensity Score 2

COMMENT:
The Allred score for estrogen and progesterone receptors is calculated by adding the sum of the proportion score (0 = no staining, 1 = <1% of cells staining, 2 = 1 - 10% of cells staining, 3 = 11-30% of cells staining, 4 = 31-60% of cells staining, 5 = >60% of cells staining) to the intensity score (1 = weak intensity of staining, 2 = intermediate intensity of staining, 3 = strong intensity of staining), with a scoring range from 0 to 8.
ER/PR positive is defined as an Allred score of >2 and ER/PR negative is defined as an Allred score of less than or equal to 2.
METHODOLOGY:
Tissue was fixed in 10% neutral buffered formalin for no less than 8 and no longer than 24 hours. Immunohistochemistry was performed using the mouse anti-human ER (ER 1D5, 1:100) and PR (PGR 136, 1:100) provided by following the manufacturer s instructions. This assay was not modified. Interpretation of the ER/PR immunohistochemical stain is guided by published results in the medical literature, information provided by the reagent manufacturer and by internal review of staining performance.

SYNOPTIC REPORT - BREAST HER-2 RESULTS
Specimen: Surgical Excision
Block Number: A4

Interpretation: EQUIVOCAL
Intensity: 2+
% Tumor Staining: 10%
Fish Ordered: Yes , on Date

METHODOLOGY:

Tissue was fixed in 10% neutral buffered formalin for no less than 8 and no longer than 24 hours. Her2 analysis was performed using the FDA approved Dako HercepTest (TM) test kit ( ) using rabbit anti-human HER2. This assay was not modified. External kit-slides provided by the manufacturer (cell lines with high, low and negative HER2 protein expression) and in-house known HER2 amplified control tissue were evaluated along with the test tissue. Adequate, well preserved, clear-cut invasive carcinoma was identified for HER2 evaluation. Interpretation of the HER2 immunohistochemical stain is guided by published results in the medical literature, information provided by the reagent manufacturer and by internal review of staining performance.

This assay has been validated according to the 2007 joint recommendations and guidelines from ASCO and CAP and from the NCCN HER2 testing in Breast Cancer Task Force. The Pathology Department takes full responsibility for this test's performance.

CLINICAL HISTORY:

None provided.

PRE-OPERATIVE DIAGNOSIS:

Right breast cancer.

INTRAOPERATIVE CONSULTATION DIAGNOSIS:

A: Right breast, lumpectomy: Mass is 0.6 cm from the closest inferior margin. By Dr., called to Dr. at P.M.

ADDENDUM:

Results of the gross examination performed on specimen A were omitted from the original report and are as follows:

PathVysion HER-2 DNA Probe Kit

Case No

Analytical Interpretation of Results: HER-2 NOT AMPLIFIED

Clinical Interpretation of results

Amplification of the HER-2 gene was evaluated with interphase fluorescence in-situ hybridization (FISH) on formalin-fixed paraffin embedded tissue sections using a chromosome 17 centromeric probe and a HER-2 probe that spans the entire HER-2 gene in the by Dr. A majority of tumors cells displayed 2 chromosome 17 signals and 2 HER-2 signals, with a HER-2/CEP 17 Ratio </=2.0, consistent with no amplification of the HER2/neu gene.

Block used A4 Source of case:

Tissue fixation formalin-fixed tissue Outside Case No: NA

Tissue source breast Results interpreted: yes

HER2/CEP17 ratio: 0.9

This ratio is derived by dividing the total number of LSI HER-2/neu signals by the total number of CEP17 signals in at least 20 interphase nuclei with nonoverlapping nuclei in the neoplastic mammary epithelial cells. Cells with no signals or with signals of only one color are disregarded.

Method of ratio enumeration: manual count

Limitations

The Vysis PathVysion Kit is not intended for use to screen for or diagnose breast cancer. It is intended to be used as an adjunct to other prognostic factors currently used to predict disease-free and overall survival in stage II, node-positive breast cancer patients. In making decisions regarding adjuvant CAF treatment, all other available clinical information should also be taken into consideration, such as tumor size, number of involved lymph nodes, and steroid receptor status. No treatment decision for stage II, node-positive breast cancer patients should be based on HER-2/neu gene amplification status alone.

Overview of this test

FDA APPROVED REAGENT

PathVysion HER-2 DNA Probe Kit is FDA approved for selection of patients for whom Herceptin® therapy is being considered. These tests were performed in the under the direction of Dr. The results of these studies should always be interpreted in the context of the clinical, morphological, and immunophenotypic diagnosis.

Gross Dictation:
Microscopic/Diagnostic Dictation: Pathologist,
Final Review: Pathologist
Final: Pathologist, (
Addendum Review: Pathologist,
Addendum Final: Pathologist
Addendum: Pathologist,
Addendum Final: Pathologist,

Criteria	Yes	No
Diagnosis Discrepancy		/
Primary Tumor Site Discrepancy		/
HIPAA Discrepancy		/
Prior Malignancy History		/
Dual/Synchronous Primary Noted		/
Case is (circle): QUALIFIED / DISQUALIFIED		
Reviewer Initials [illegible] Date Reviewed: [illegible]		

Metastatic Lymph Node

UUID:72FE0659-6B7E-4129-B61D-C376C59C428F
TCGA-E2-A15K-06A-PR Redacted

TSS:

ICD-O-3
carcinoma, infiltrating duct, NOS 8500/3
Site: lymph node, axillary C77.3
jw 3/5/14

SPECIMENS:
A. RIGHT BREAST LUMPECTOMY
B. RIGHT AXILLARY CONTENTS LEVELS 1,2
C. ADDITIONAL RIGHT AXILLARY TISSUE

SPECIMEN(S):
A. RIGHT BREAST LUMPECTOMY
B. RIGHT AXILLARY CONTENTS LEVELS 1,2
C. ADDITIONAL RIGHT AXILLARY TISSUE

GROSS DESCRIPTION:
A. RIGHT BREAST LUMPECTOMY
Received fresh labeled with the patient's identification and "right breast lumpectomy" is a previously inked, oriented 97g, 8 x 6.5 x 4.5cm lumpectomy. Ink code: anterior-yellow, posterior-black, superior-blue, inferior-orange, medial-green, lateral-red. Specimen is serially sectioned from lateral to medial into 8 slices revealing a 2 x 1.8 x 1.6cm tan white stellate mass, 0.6cm from the closest inferior margin in slices 2-5cm. A portion of the specimen is submitted for tissue procurement. Representatively submitted:
A1: lateral margin slice 1
A2-A4: slice 2 with mass in A4
A5-A7: slice 3 with mass in A6
A8-A11: slice 4
A12-A15: slice 5 with mass in A15
A16-A17: slice 6
A18-A19: slice 7
A20: medial margin slice 8
B. RIGHT AXILLARY CONTENTS LEVELS 1,2
Received fresh is a tan pink soft tissue fragment 15 x 12 x 4cm. Dissection reveals 14 lymph nodes ranging from 0.3 x 0.3 x 0.2cm to 5 x 3.2 x 1.5cm. The largest lymph node is sectioned to reveal a firm homogenous white cut surface.
B1: 5 lymph nodes
B2: 4 lymph nodes
B3: 2 lymph nodes
B4: 1 lymph node
B5: 1 lymph node
B6: 1 lymph node
B7: 1 lymph node
B8: 1 lymph node
B9-B10: 1 lymph node
B11-B12: 1 lymph node
B13-B16: representative sections of 1 lymph node
C. ADDITIONAL RIGHT AXILLARY TISSUE
Received fresh is a tan pink soft tissue fragment 4.3 x 2.7 x 2cm. Dissection reveals a possible necrotic lymph node 2.8 x 1.3 x 1cm. Representatively submitted in C1-C4.

DIAGNOSIS:
A. BREAST, RIGHT, WIDE LOCAL EXCISION:
- INVASIVE DUCTAL CARCINOMA, SBR GRADE 3, MEASURING 2.4-CM
- INTERMEDIATE NUCLEAR GRADE, DUCTAL CARCINOMA IN SITU, SOLID TYPE
- INVASIVE TUMOR INVOLVES INFERIOR SURGICAL RESECTION MARGIN AND PRESENT WITHIN 1 MM FROM MEDIAL SURGICAL RESECTION MARGIN
- BIOPSY SITE CHANGES WITH FIBROSIS
- SEE SYNOPTIC REPORT.

B. LYMPH NODES, RIGHT, AXILLARY DISSECTION:
- METASTATIC CARCINOMA TO TWO OF SEVENTEEN LYMPH NODES (2/17), LARGEST MEASURING 2.5-CM, WITH EXTRANODAL EXTENSION.

C. SOFT TISSUE, ADDITIONAL WHITE AXILLARY, EXCISION:
- FIBROADIPOSE TISSUE WITH FAT NECROSIS, NO TUMOR SEEN.

SYNOPTIC REPORT - BREAST
Specimen Type: Excision

Needle Localization: No
Laterality: Right
Invasive Tumor: Present
Multifocality: No
WHO CLASSIFICATION
Invasive ductal carcinoma, NOS 8500/3
Tumor size: 2.4cm
Margins: Involved at
inferior
Extent:: focal
Tubular Score: 3
Nuclear Grade: 2
Mitotic Score: 3
Modified Scarff Bloom Richardson Grade: 3
Necrosis: Absent
Vascular/Lymphatic Invasion: Present
Extent: focal
Lobular neoplasia: None
Lymph nodes: Axillary dissection
Lymph node status: Positive 2 / 17 Extranodal extension
Micrometastases: No

DCIS present
Margins uninvolved by DCIS
DCIS Quantity: Estimate 2%
DCIS Type: Solid
DCIS Location: Associated with invasive tumor
Nuclear grade: Intermediate
Necrosis: Absent

ER/PR/HER2 Results
ER: Positive
PR: Positive
HER2: Pending by FISH

Pathological staging (pTN): pT 2 N 1a
Pathological staging is based on the AJCC Cancer Staging Manual, 7th Edition

SYNOPTIC REPORT - BREAST, ER/PR RESULTS
Specimen: Surgical Excision
Block Number: A4

ER: Positive Allred Score: 8 = Proportion Score 5 + Intensity Score 3
PR: Positive Allred Score: 4 = Proportion Score 2 + Intensity Score 2

COMMENT:
The Allred score for estrogen and progesterone receptors is calculated by adding the sum of the proportion score (0 = no staining, 1 = <1% of cells staining, 2 = 1 - 10% of cells staining, 3 = 11-30% of cells staining, 4 = 31-60% of cells staining, 5 = >60% of cells staining) to the intensity score (1 = weak intensity of staining, 2 = intermediate intensity of staining, 3 = strong intensity of staining), with a scoring range from 0 to 8.
ER/PR positive is defined as an Allred score of >2 and ER/PR negative is defined as an Allred score of less than or equal to 2.
METHODOLOGY:
Tissue was fixed in 10% neutral buffered formalin for no less than 8 and no longer than 24 hours. Immunohistochemistry was performed using the mouse anti-human ER (ER 1D5, 1:100) and PR (PGR 136, 1:100) provided by following the manufacturer s instructions. This assay was not modified. Interpretation of the ER/PR immunohistochemical stain is guided by published results in the medical literature, information provided by the reagent manufacturer and by internal review of staining performance.

SYNOPTIC REPORT - BREAST HER-2 RESULTS
Specimen: Surgical Excision
Block Number: A4

Interpretation: EQUIVOCAL
Intensity: 2+
% Tumor Staining: 10%
Fish Ordered: Yes , on Date

METHODOLOGY:

Tissue was fixed in 10% neutral buffered formalin for no less than 8 and no longer than 24 hours. Her2 analysis was performed using the FDA approved using rabbit anti-human HER2. This assay was not modified. External kit-slides provided by the manufacturer (cell lines with high, low and negative HER2 protein expression) and in-house known HER2 amplified control tissue were evaluated along with the test tissue. Adequate, well preserved, clear-cut invasive carcinoma was identified for HER2 evaluation. Interpretation of the HER2 immunohistochemical stain is guided by published results in the medical literature, information provided by the reagent manufacturer and by internal review of staining performance.

This assay has been validated according to the 2007 joint recommendations and guidelines from ASCO and CAP and from the NCCN HER2 testing in Breast Cancer Task Force. The Pathology Department takes full responsibility for this test's performance.

CLINICAL HISTORY:

None provided.

PRE-OPERATIVE DIAGNOSIS:

Right breast cancer.

INTRAOPERATIVE CONSULTATION DIAGNOSIS:

A: Right breast, lumpectomy: Mass is 0.6 cm from the closest inferior margin. By Dr., called to Dr. at

ADDENDUM:

Results of the gross examination performed on specimen A were omitted from the original report and are as follows:

HER-2 DNA Probe Kit

Case No

Analytical Interpretation of Results: HER-2 NOT AMPLIFIED

Clinical Interpretation of results

Amplification of the HER-2 gene was evaluated with interphase fluorescence in-situ hybridization (FISH) on formalin-fixed paraffin embedded tissue sections using a chromosome 17 centromeric probe and a HER-2 probe that spans the entire HER-2 gene in the by Dr. A majority of tumors cells displayed 2 chromosome 17 signals and 2 HER-2 signals, with a HER-2/CEP 17 Ratio </=2.0, consistent with no amplification of the HER2/neu gene.

Block used A4 Source of case:

Tissue fixation formalin-fixed tissue Outside Case No: NA

Tissue source breast Results interpreted: yes

HER2/CEP17 ratio: 0.9

This ratio is derived by dividing the total number of HER-2/neu signals by the total number of CEP17 signals in at least 20 interphase nuclei with nonoverlapping nuclei in the neoplastic mammary epithelial cells. Cells with no signals or with signals of only one color are disregarded.

Method of ratio enumeration: manual count

Limitations

The is not intended for use to screen for or diagnose breast cancer. It is intended to be used as an adjunct to other prognostic factors currently used to predict disease-free and overall survival in stage II, node-positive breast cancer patients. In making decisions regarding adjuvant CAF treatment, all other available clinical information should also be taken into consideration, such as tumor size, number of involved lymph nodes, and steroid receptor status. No treatment decision for stage II, node-positive breast cancer patients should be based on HER-2/neu gene amplification status alone.

Overview of this test

FDA APPROVED REAGENT

HER-2 DNA Probe Kit is FDA approved for selection of patients for whom Herceptin® therapy is being considered. These tests were performed in the under the direction of Dr. The results of these studies should always be interpreted in the context of the clinical, morphological, and immunophenotypic diagnosis.

Gross Dictation:
Microscopic/Diagnostic Dictation: Pathologist,
Final Review: Pathologist,
Final: Pathologist,
Addendum Review: Pathologist,
Addendum Final: Pathologist,
Addendum: Pathologist,
Addendum Final: Pathologist,

Metastatic Axillary Lymph Node.

Criteria [illegible] 3/5/14	Yes	No
Diagnosis Discrepancy		✓
Primary Tumor Site Discrepancy Met		✓
HIPAA Discrepancy		✓
Prior Malignancy History		✓
Dual/Synchronous Primary Noted		✓
Case is (circle): QUALIFIED / DISQUALIFIED		
Reviewer Initials [illegible] Date Reviewed: [illegible]		

ICD-O-3
Carcinoma, infiltrating duct, NOS
8500/3 12/8/10
lw
Site Code · breast, NOS C50.9

TSS

UUID:AA4B6E2D-7503-4E51-BEA4-632CC994770A
TCGA-E2-A150-01A-PR Redacted

SPECIMENS:
A. WLE RIGHT BREAST NEEDLE LOC.
B. ADDITIONAL MARGIN RIGHT BREAST
C. WLE LEFT BREAST NEEDLE LOCALIZATION

SPECIMEN(S):
A. WLE RIGHT BREAST NEEDLE LOC.
B. ADDITIONAL MARGIN RIGHT BREAST
C. WLE LEFT BREAST NEEDLE LOCALIZATION

GROSS DESCRIPTION:
A. WLE RIGHT BREAST NEEDLE LOC.
Received fresh labeled with the patient's identification and "WLE right breast needle loc" is an oriented (short/1 clip-superior, long/2 clips-lateral, air knot-anterior) needle localized lumpectomy with radiograph. Ink code: Anterior-yellow, posterior-black, medial-green, lateral-red, superior-blue, inferior-orange. The specimen is serially sectioned from lateral to medial into 8 slices revealing a 2.4 x 1.9 x 1.6 cm firm tan stellate mass that is closest to the superior margin at 0.2 cm. Tissue is procured. Representatively submitted:
A1-A2: lateral margin, perpendicular resection
A3: slice 2, anterior/superior
A4: slice 2, posterior superior (mass)
A5: slice 3, anterior/superior (mass)
A6: slice 3, posterior superior (mass)
A7: slice 3, anterior/inferior (mass)
A8: slice 3, posterior inferior (mass)
A9: slice 4, anterior/superior (mass)
A10: slice 4, mid superior (mass)
A11: slice 4, posterior superior (mass)
A12: slice 4, anterior inferior (mass)
A13: slice 4, posterior inferior (mass)
A14: slice 5, anterior superior (mass)
A15: slice 5, mid superior (mass)
A16: slice 5, anterior inferior (mass)
A17: slice 5, mid inferior (mass)
A18: slice 6, anterior superior
A19: slice 6, mid superior (mass)
A20: slice 6, posterior superior
A21: slice 6, mid inferior
A22: medial margin, perpendicular sections
B. ADDITIONAL MARGIN RIGHT BREAST
Received fresh labeled with the patient's identification and "additional margin right breast" is in a oriented (short-superior, long-lateral) 11 g, 0.2 x 3.4 x 1.1 cm fibrofatty tissue. Margins are inked black. Serial sectioning reveals no discrete lesions. Inked margin submitted entirely in cassettes B1-B7.
NOTE: specimen was received with only 1 suture
C. WLE LEFT BREAST NEEDLE LOCALIZATION
Received fresh labeled with patient's identification and "WLE left breast needle localization" is an oriented (short/1 clip-superior, long/2 clips-lateral, loop-anterior) 42 g, 4 x 4 x 3.5 cm needle localized lumpectomy with radiograph. Ink code: Anterior-yellow, posterior-black, medial-green, lateral-red, superior-blue, inferior-orange. Specimen is serially sectioned from lateral to medial into 6 slices revealing a 2.4 x 1.9 x 1.8 cm firm tan stellate mass that is closest to the superior margin at 0.1 cm. Tissue is procured. Representatively submitted:
C1-C2: lateral margin, perpendicular sections
C3: slice 2, anterior superior (mass)
C4: slice 2, posterior superior (mass)
C5: slice 2, anterior inferior (mass)
C6: slice 2, posterior inferior (mass)
C7: slice 3, mid superior (mass)
C8: slice 3, posterior superior (mass)
C9: slice 3, mid inferior (mass)
C10: slice 3, posterior inferior (mass)
C11: slice 4, anterior superior
C12: slice 4, mid superior (mass)
C13: slice 4, posterior superior
C14: slice 5, anterior inferior

C15: slice 5, mid anterior
C16: slice 5, anterior superior
C17-C18: medial margin, perpendicular sections

DIAGNOSIS:

A. BREAST, RIGHT, WIDE LOCAL EXCISION:
- INVASIVE DUCTAL CARCINOMA, SBR GRADE 2, MEASURING 2.4-CM
- INTERMEDIATE NUCLEAR GRADE, DUCTAL CARCINOMA IN SITU, SOLID AND CRIBRIFORM TYPES WITH CENTRAL NECROSIS AND MICROCALCIFICATIONS
- INVASIVE TUMOR PRESENT WITHIN 1-MM FROM SUPERIOR/MEDIAL SURGICAL RESECTION MARGIN
- LOBULAR CARCINOMA IN SITU
- BIOPSY SITE CHANGES WITH FIBROSIS
- SEE SYNOPTIC REPORT AND SEE NOTE.

B. BREAST, RIGHT, ADDITIONAL SUPERIOR MARGIN, EXCISION:
- INVASIVE DUCTAL CARCINOMA INVOLVES NEW INKED SURGICAL RESECTION MARGIN, SEE NOTE.

C. BREAST, LEFT, WIDE LOCAL EXCISION:
- INVASIVE DUCTAL CARCINOMA, SBR GRADE 2, MEASURING 1.8-CM
- SURGICAL RESECTION MARGIN NEGATIVE FOR TUMOR
- LOBULAR CARCINOMA IN SITU
- BIOPSY SITE CHANGES WITH FIBROSIS
- SEE SYNOPTIC REPORT.

NOTE: Part B. was designated as superior by Dr. Therefore, invasive tumor is present at superior (part B) and within 1-mm from medial (part A) surgical resection margin.

E-cadherin is negative in LCIS component.

SYNOPTIC REPORT - BREAST
Specimen Type: Excision
Needle Localization: Yes
Laterality: Right
Invasive Tumor: Present
Multifocality: No
WHO CLASSIFICATION
Invasive ductal carcinoma, NOS 8500/3
Tumor size: 2.4cm
Margins: Involved at
superior
and within 1-mm from medial
Extent:: focal
Tubular Score: 2
Nuclear Grade: 2
Mitotic Score: 3
Modified Scarff Bloom Richardson Grade: 2
Necrosis: Absent
Vascular/Lymphatic Invasion: None identified
Lobular neoplasia: LCIS
Lymph nodes: Non-sentinel lymph node

DCIS present
Margins uninvolved by DCIS
DCIS Quantity: Estimate 10%
DCIS Type: Solid
Cribriform
DCIS Location: Associated with invasive tumor
Nuclear grade: Intermediate
Necrosis: Present

ER/PR/HER2 Results
ER: Positive
PR: Positive
HER2: Negative by IHC
Performed on Case:

Pathological staging (pTN): pT 2 N x
Pathological staging is based on the AJCC Cancer Staging Manual, 7th Edition

SYNOPTIC REPORT - BREAST
Specimen Type: Excision
Needle Localization: Yes
Laterality: Left
Invasive Tumor: Present
Multifocality: No
WHO CLASSIFICATION
Invasive ductal carcinoma, NOS 8500/3
Tumor size: 1.8cm
Margins: Negative
Distance from closest margin: 0.3cm
superior
Tubular Score: 2
Nuclear Grade: 2
Mitotic Score: 2
Modified Scarff Bloom Richardson Grade: 2
Necrosis: Absent
Vascular/Lymphatic Invasion: None identified
Lobular neoplasia: LCIS
Lymph nodes: No lymph node sampling

DCIS not present

ER/PR/HER2 Results
ER: Positive
PR: Positive
HER2: Negative by FISH
Performed on Case:

Pathological staging (pTN): pT 1c N x
Pathological staging is based on the AJCC Cancer Staging Manual, 7th Edition

CLINICAL HISTORY:

female found with left breast lump. She had bilateral mammogram done showing bilateral breast masses. Bilateral core needle biopsies done on showed bilateral invasive ductal carcinoma. Bilateral needle localization scheduled and no sentinel lymph nodes will ~~be taken as her axilla are clinically~~ negative and it would not change post op management she will likely received radiation and endocrine therapy.

PRE-OPERATIVE DIAGNOSIS:

Bilateral invasive ductal carcinoma
Right-ER/PR+, Her2/neu -, SBR grade 2
Left-ER/PR+, SBR grade 1

ADDENDUM:

The right breast excision (Part A) measures 5x5x4.5-cm and weighs 63 gms.

Microscopic/Diagnostic Dictation: Pathologist,
Final Review: Pathologist.
Final: Pathologist,
Addendum: Pathologist,
Addendum Final: Pathologist.

Criteria	Yes	No
Diagnosis Discrepancy		/
Primary Tumor Site Discrepancy		/
HIPAA Discrepancy		/
Prior Malignancy History		/
Dual/Synchronous Primary Noted	/	
Case is (circle): QUALIFIED / DISQUALIFIED		
Reviewer Initials: [illegible] Date Reviewed: [illegible]		

ICD-O-3
Carcinoma, Infiltrating duct, NOS
8500/3
Site Code: breast, NOS C50.9
12/9/10

TSS Pt ID:

UUID:DDBEE56C-F8E8-4A7D-8CF6-E0D63EE53FC4
TCGA-E2-A108-01A-PR
Redacted

SPECIMENS:
A. SENTINEL LYMPH NODE #1 RIGHT AXILLA
B. SENTINEL LYMPH NODE #2 RIGHT AXILLA
C. RIGHT BREAST LUMPECTOMY
D. RIGHT AXILLARY CONTENTS LEVELS 1 AND 2
E. SUPERIOR MARGIN
F. MEDIAL MARGIN
G. INFERIOR MARGIN

SPECIMEN(S):
A. SENTINEL LYMPH NODE #1 RIGHT AXILLA
B. SENTINEL LYMPH NODE #2 RIGHT AXILLA
C. RIGHT BREAST LUMPECTOMY
D. RIGHT AXILLARY CONTENTS LEVELS 1 AND 2
E. SUPERIOR MARGIN
F. MEDIAL MARGIN
G. INFERIOR MARGIN

INTRAOPERATIVE CONSULTATION DIAGNOSIS:
TPA1: LN positive for carcinoma (SLN #1)
TPB1 and TPB2: LY negative for carcinoma (SLN #2)
C: Gross: Right breast, 1st lesion 2.2 cm, at distance from all margins (1.5 from closest superior margin)
2nd lesion 3.0 cm from the 1st lesion, size 0.8 cm, 0.4 cm from the closest inferior margin.
By Dr. called to Dr. at . (A, B) and [illegible])

GROSS DESCRIPTION:
A. SENTINEL LYMPH NODE #1 RIGHT AXILLA
Received fresh labeled with the patient identification and "sentinel lymph node #1", is a 2.0 x 1.0 x 0.8-cm fatty lymph node displaying a 0.5 x 0.4 x 0.4-cm firm white tumor nodule. Touch preparations are performed. The lymph node is representatively submitted in cassette A1.
B. SENTINEL LYMPH NODE #2 RIGHT AXILLA
Received fresh labeled with the patient identification and "sentinel lymph node #2, right axilla", are 2 tan-pink to fatty lymph nodes, 0.5 cm (B1) and 1.2 x 0.6 x 0.5 cm (B2). A touch preparation on each lymph node is performed and the lymph nodes are entirely submitted as follows:
B1: Smaller lymph node
B2: Largest lymph node
C. RIGHT BREAST LUMPECTOMY
Received fresh labeled with the patient identification and "right breast lumpectomy", is an oriented (single - anterior, double - lateral, triple - superior), 153.0-gram, 11.5 x 8.5 x 3.0-cm needle localized lumpectomy with radiograph. Ink code: Anterior - yellow, posterior - black, medial - green, lateral - red, superior - blue, inferior - orange. The specimen is serially sectioned into 9 slices revealing a 2.2 x 1.8 x 1.8-cm infiltrative firm gritty white tumor (mass #1 - 12 o'clock). Mass #1 is closest to the anterior and superior margins at 1.5 cm each. Tissue is procured. There is a second mass/biopsy site, 0.8 cm which is closest to the inferior margin at 0.4 cm and 3.0 cm from the first mass. The remaining fibrous tissue is finely lobulated with interspersed clear fluid-filled 0.2-cm cysts. No additional nodules are identified. A gross evaluation is performed. Representatively submitted:
C1: Slice 1, medial margin, perpendicular
C2-C3: Slice 4, tumor to closest anterior superior margins
C4-C6: Slice 6, tumor to superior posterior margins
C7: Slice 6, anterior margin
C8: Slice 6
C9: Slice 6, posterior margin
C10: Slice 6, anterior margin
C11: Slice 6
C12: Slice 6, posterior margin
C13: Slice 7, superior margin
C14: Slice 7, anterior margin
C15: Slice 7, posterior margin
C16: Slice 7, anterior margin

C17: Slice 7
C18: Slice 7, posterior margin
C19: Slice 7, anterior margin
C20: Slice 7, mass #2/biopsy cavity
C21: Slice 7, posterior margin
C22: Slice 9, lateral margin, perpendicular

D. RIGHT AXILLARY CONTENTS LEVELS 1 AND 2

Received in formalin labeled with the patient identification and "right axillary contents levels 1 and 2", is a 9.0 x 5.0 x 1.8-cm portion of adipose tissue, within which 20 possible lymph nodes are identified ranging from 0.2 to 3.5 cm. There are 2 tan-pink to fatty lymph nodes exhibiting infiltrative tumor, 0.4 and 0.5 cm. Also identified are 3 matted lymph nodes with an overall dimension of 2.2 cm. The specimen is representatively submitted as follows:

D1: Four whole lymph nodes
D2: Four whole lymph nodes
D3: Three whole lymph nodes
D4: One lymph node bisected
D5: One lymph node bisected
D6: Two whole lymph nodes
D7: Two positive lymph nodes
D8: Three possible matted lymph nodes
D9-D20: Remaining soft tissue

E. SUPERIOR MARGIN

Received in formalin labeled with the patient identification and "new superior margin", is a 5.0-gram, 6.0 x 2.0 x 0.8-cm oriented portion of breast (suture at final margin). The final margin is inked black and on serial sectioning, no discrete lesions are identified. Entirely submitted in cassettes E1-E4.

F. MEDIAL MARGIN

Received in formalin labeled with the patient identification and "medial margin", is an oriented (suture at final margin) 5.0-gram, 4.5 x 2.8 x 1.7-cm fibrofatty tissue. The final margin is inked black and on serially sectioning, no discrete lesions are identified. Entirely submitted in cassettes F1-F4.

G. INFERIOR MARGIN

Received in formalin labeled with the patient identification and "inferior margin", is an oriented (suture at final margin) 4.0-gram, 4.0 x 1.5 x 1.3-cm fibrofatty tissue. The final margin is inked black and on serially sectioning, no discrete lesions are identified. Entirely submitted in cassettes G1-G3.

DIAGNOSIS:

A. LYMPH NODE, SENTINEL #1, RIGHT AXILLA, EXCISION:
- METASTATIC CARCINOMA TO ONE OF ONE LYMPH NODE (1/1), MEASURING 0.4-CM WITH NO EXTRANODAL EXTENSION.

B. LYMPH NODES, SENTINEL #2, RIGHT AXILLA, EXCISION:
- TWO LYMPH NODES, NEGATIVE FOR METASTASES (0/2).

C. BREAST, RIGHT, WIDE LOCAL EXCISION:
- INVASIVE DUCTAL CARCINOMA, SBR GRADE 3, MEASURING 2.2-CM
- HIGH NUCLEAR GRADE, DUCTAL CARCINOMA IN SITU, SOLID TYPE WITH CENTRAL NECROSIS AND LOBULAR EXTENSION
- SURGICAL RESECTION MARGINS NEGATIVE FOR TUMOR
- BIOPSY SITE CHANGES WITH FIBROSIS AND GRANULATION TISSUE
- SEE SYNOPTIC REPORT AND SEE NOTE.

D. LYMPH NODES, RIGHT AXILLARY CONTENTS, LEVELS 1 AND 2, DISSECTION:
- METASTATIC CARCINOMA TO FOUR OF TWENTY FOUR LYMPH NODES (4/24), LARGEST MEASURING 1.0-CM WITH FOCAL EXTRANODAL EXTENSION.

E. BREAST, SUPERIOR MARGIN, EXCISION:
- BREAST TISSUE, NO TUMOR SEEN.

F. BREAST, MEDIAL MARGIN, EXCISION:
- BREAST TISSUE, NO TUMOR SEEN.

G. BREAST, INFERIOR MARGIN, EXCISION:
- BREAST TISSUE, NO TUMOR SEEN.

NOTE: Grossly, two tumor masses are identified, one larger located in the superior anterior and one smaller located in the inferior lateral aspect of the specimen. The larger mass is composed of invasive ductal carcinoma measuring 2.2-cm. The smaller tumor is composed of biopsy site changes with granulation tissue intermixed with clusters of neoplastic ducts located only in the lymphatic channels (CD31 and D2-40 mark involved spaces). Located in the same level (slice #7) more towards the center of the specimen is a microscopic focus of DCIS.

SYNOPTIC REPORT - BREAST
Specimen Type: Excision
Needle Localization: Yes
Laterality: Right
Invasive Tumor: Present
Multifocality: No
WHO CLASSIFICATION
Invasive ductal carcinoma, NOS 8500/3
Tumor size: 2.2cm
Tumor Site: Not specified
Margins: Negative
Tubular Score: 3
Nuclear Grade: 3
Mitotic Score: 3
Modified Scarff Bloom Richardson Grade: 3
Necrosis: Absent
Vascular/Lymphatic Invasion: Present
Extent: at 9:00 position
Lobular neoplasia: None
Lymph nodes: Sentinel lymph node and axillary dissection
Lymph node status: Positive 5 / 27 Extranodal extension
Micrometastases: No

DCIS present
Margins uninvolved by DCIS
DCIS Quantity: Estimate 2%
DCIS Type: Solid
DCIS Location: Separate from invasive tumor mass
Nuclear grade: High
Necrosis: Present

ER/PR/HER2 Results
ER: Positive
PR: Positive
HER2: Negative
Performed on Case:

Pathological staging (pTN): pT 2 N 2a

CLICAL HISTORY:

-year-old female multifocal IDC right breast, 2 areas at 12 and 9 o'clock. Now for lumpectomy/SNB.

PRE-OPERATIVE DIAGNOSIS:

None given

Gross Dictation: Pathologist,
Microscopic/Diagnostic Dictation: Pathologist,
Final Review:., Pathologist,
Final: Pathologist,

Criteria	Yes	No
Diagnosis Discrepancy		✓
Primary Tumor Site Discrepancy		✓
HIPAA Discrepancy		✓
Prior Malignancy History		✓
Dual/Synchronous Primary Noted		✓
Case is (circle): QUALIFIED / DISQUALIFIED		
Reviewer Initials ______ Date Reviewed: ___/___/___		

ICD-O-3
Carcinoma, infiltrating duct, NOS 8500/3
Path Site Code: breast, upper-outer quadrant C50.4
CQCF Site: breast, NOS C50.9
12/19/10

TSS Pat ID:

UUID:089E0FB9-6B45-4E3E-843E-EC98D39863F6
TCGA-E2-A109-01A-PR Redacted

SPECIMENS:
A. SENTINEL LYMPH NODE #1
B. SENTINEL LYMPH NODE 2 RIGHT AXILLA
C. SENTINEL LYMPH NODE 3 RIGHT AXILLA
D. SENTINEL LYMPH NODE 4 RIGHT AXILLA
E. SENTINEL LYMPH NODE #5
F. RIGHT BREAST
G. ADDITIONAL LATERAL TISSUE RIGHT BREAST
H. ADDITIONAL SUPERIOR RIGHT BREAST TISSUE

SPECIMEN(S):
A. SENTINEL LYMPH NODE #1
B. SENTINEL LYMPH NODE 2 RIGHT AXILLA
C. SENTINEL LYMPH NODE 3 RIGHT AXILLA
D. SENTINEL LYMPH NODE 4 RIGHT AXILLA
E. SENTINEL LYMPH NODE #5
F. RIGHT BREAST
G. ADDITIONAL LATERAL TISSUE RIGHT BREAST
H. ADDITIONAL SUPERIOR RIGHT BREAST TISSUE

INTRAOPERATIVE CONSULTATION DIAGNOSIS:
TPA, B, C, D, E. Sentinel lymph nodes #1, 2, 3, 4, 5, biopsies: No tumor seen.
By Dr, called to Dr. at . (A,B), . C, D) and at . (E).

GROSS DESCRIPTION:

A. SENTINEL LYMPH NODE #1
Received is a tan-pink fatty lymph node (1.8 x 0.9 x 0.3 cm). The specimen is serially sectioned, touch prep was performed. The specimen is submitted is cassettes A1-A3.

B. SENTINEL LYMPH NODE #2 RIGHT AXILLA
Received is a tan-pink fatty lymph node (1.5 x .6 x .3 cm). The specimen is serially sectioned and touch preps are taken. The specimen is submitted in toto in B.

C. SENTINEL LYMPH NODE #3 RIGHT AXILLA
Received are two tan-pink lymph nodes (1.0 x 0.3 x 0.2 cm and 1.4 x .6 x .5 cm). The specimen is serially sectioned and touch preps are taken. The specimen is submitted as follows:
C1: one lymph node, trisected
C2: one lymph node serially sectioned

D. SENTINEL LYMPH NODE #4 RIGHT AXILLA
Received is a tan-pink lymph node (1.0 x .6 x .3 cm). The specimen is serially sectioned and touch preps are taken. The specimen is submitted in toto in cassette D.

E. SENTINEL LYMPH NODE #5
Received is a tan-pink lymph node (.9 x .7 x .2 cm). The specimen is serially sectioned and touch preps are taken. The specimen is submitted in toto in cassette E.

F. RIGHT BREAST
Received fresh labeled with the patient name, designated "right breast", is a simple mastectomy specimen weighing 1181 grams and measuring overall 28 x 23 x 4.5 cm. The specimen is received with orientation, a suture indicating the axillary aspect. The overlying beige-tan ellipse of skin measures 21 x 11 cm. The surface demonstrates three areas of brown hyperpigmentation, the largest measuring 1 x 0.6 cm to the smallest measuring 0.5 x 0.3 cm. A light tan raised lesion is noted at 3 o'clock on the skin measuring 0.5 x 0.5 cm. The light tan areola measures 2.3 cm in diameter, the everted nipple measures 1 cm in diameter. The deep margin is inked black. The specimen is serially sectioned from axilla to medial aspect and shows a firm beige-tan lesion in the upper outer quadrant at approximately 10 o'clock approaching the deep surgical margin at a distance of 4.3 cm. The lesion measures 2.2 x 1.9 x 1.5 cm. An ill defined white firm fibrous area is also demonstrated at approximately 12 o'clock measuring 1.5 x 1.2 x 1 cm. This area is located 5.5 cm from the lesion. An ill defined irregular dense white area is shown in the lower inner quadrant measuring 2.5 x 2 x 2 cm. This area is located approximately 10.5 cm from the lesion. The remainder of the breast parenchyma shows dark yellow adipose tissue. A portion of the specimen was submitted for tissue procurement. Representative sections are submitted as follows:
F1-F4: the lesion in the upper outer quadrant
F5: deep margin overlying lesion
F6-F8: sections of white firm fibrous tissue at 12 o'clock

F9-F16:	multiple sections of ill defined firm area in lower inner quadrant
F17-F18:	representative sections of upper inner quadrant
F19-F21:	representative sections of the lower outer quadrant
F22-F23:	additional sections from the upper outer quadrant adjacent to lesion
F24:	section of nipple
F25:	section of skin demonstrating the raised tan lesion at 3 o'clock
F26:	additional section of skin
F27-F28:	possible lymph nodes

G. ADDITIONAL LATERAL TISSUE RIGHT BREAST
Received in formalin in a container labeled with the patient name, designated "additional lateral tissue", is a fragment of dark yellow adipose tissue measuring 8 x 2.2 x 0.5 cm. The exterior surface is inked black. The entire specimen is submitted in cassettes G1-G4.

H. ADDITIONAL SUPERIOR RIGHT BREAST TISSUE
Received in formalin in a container labeled with the patient name designated "additional superior right breast tissue", is a fragment of yellow adipose tissue measuring 3 x 2.2 x 1 cm. The entire specimen is submitted in cassettes H1 and H2.

DIAGNOSIS:

A. SENTINEL LYMPH NODE #1, EXCISION:
- ONE LYMPH NODE, NEGATIVE FOR TUMOR (0/1).

B. SENTINEL LYMPH NODE #2, RIGHT AXILLA, EXCISION:
- ONE LYMPH NODE, NEGATIVE FOR TUMOR (0/1).

C. SENTINEL LYMPH NODE #3, RIGHT AXILLA, EXCISION:
- TWO LYMPH NODES, NEGATIVE FOR TUMOR (0/2).

D. SENTINEL LYMPH NODE #4, RIGHT AXILLA, EXCISION:
- ONE LYMPH NODE, NEGATIVE FOR TUMOR (0/1).

E. SENTINEL LYMPH NODE #5, EXCISION:
- ONE LYMPH NODE, NEGATIVE FOR TUMOR (0/1).

F. RIGHT BREAST, SIMPLE MASTECTOMY:
- TWO FOCI OF INVASIVE DUCTAL CARCINOMA, TUMOR SIZE 2.2 x 1.9 x 1.5 CM. AND 1 x 1 CM. RESPECTIVELY, SBR GRADE III IN LARGE TUMOR FOCUS.
- DUCTAL CARCINOMA IN-SITU, COMEDO AND SOLID TYPES, HIGH NUCLEAR GRADE WITH MICROCALCIFICATIONS.
- MARKED FIBROCYSTIC DISEASE AND ADENOSIS WITH EXTENSIVE MICROCALCIFICATIONS.
- SURGICAL RESECTION MARGINS, NEGATIVE FOR TUMOR.
- FOCAL SEBORRHEIC KERATOSIS OF SKIN.
- SEE TEMPLATE.

Note: There are two foci of invasive ductal carcinoma identified: the large one is present in the upper outer quadrant measuring 2.2 cm. This focus is SBR grade III. Another small tumor focus is present in the central area at the 12 o'clock position measuring 1 x 1 cm. This focus of tumor is SBR grade I, with tubular formation. Ductal carcinoma in-situ containing microcalcifications is associated with the large focus of invasive carcinoma. In addition microcalcifications are also present in mutifoci of adenosis and fibrocystic disease.

G. ADDITIONAL LATERAL TISSUE RIGHT BREAST, EXCISION:
- BENIGN ADIPOSE TISSUE, NEGATIVE FOR TUMOR.

H. ADDITIONAL SUPERIOR RIGHT BREAST TISSUE, EXCISION:
- BENIGN ADIPOSE TISSUE, NEGATIVE FOR TUMOR.

SYNOPTIC REPORT - BREAST
Specimens Involved
Specimens: F: RIGHT BREAST

Specimen Type: Mastectomy
Needle Localization: No
Laterality: Right
Invasive tumor: Present
Multifocality: Yes
WHO CLASSIFICATION
Invasive ductal carcinoma, NOS 8500/3
Specimen size: Size of Invasive focus 2.2cm

Additional dimensions: 1.9cm x 1.5cm
Tumor Site: Upper outer quadrant
Central
Margins: Negative
Distance from closest margin: 4.3cm
Tubular score: 3 (<10% tubule)
Nuclear grade: 3
Mitotic score (Olympus 40x): 2 (7-13/10
Modified Scarff Bloom Richardson Grade: III (8-9 points)
Necrosis: Absent
Vascular/Lymphatic Invasion: None identified
Lobular neoplasia: None
Lymph nodes: Sentinel lymph node only
Lymph node status: Negative 0 / 6
DCIS present
Margins uninvolved by DCIS
DCIS Quantity: Estimate % 15
DCIS type: Comedo
Solid
DCIS location: Associated with invasive tumor
Nuclear grade: High
Necrosis: Present
Location of CA++: DCIS
Benign epithelium
Pathological staging (pTN): pT 2 N 0

SYNOPTIC REPORT - BREAST, ER/PR RESULTS
Specimens Involved
Specimens: F: RIGHT BREAST

SPECIMEN:
Other
simple mastectomy
Block Number: F1
ER: Positive - Allred Score: 8 = Proportion score: 5 + Intensity Score 3
PR: Negative - Allred Score: 0 = Proportion Score 0 + Intensity Score 0
COMMENT:
The Allred score for estrogen and progesterone receptors is calculated by adding the sum of the proportion score (0 = no staining, 1 = <1% of cells staining, 2 = 1 - 10% of cells staining, 3 = 11-30% of cells staining, 4 = 31-60% of cells staining, 5 = >60% of cells staining) to the intensity score (1 = weak intensity of staining, 2 = intermediate intensity of staining, 3 = strong intensity of staining), with a scoring range from 0 to 8.
ER/PR positive is defined as an Allred score of >2 and ER/PR negative is defined as an Allred score of less than or equal to 2.
Methodology: Fixation Type and Length: Tissue was fixed in 10% neutral buffered formalin (for no less than 8 and no longer than 24 hours. Antibody and Assay Methodology: Mouse anti-human ER and PR, ( ).
Comment: This assay can be used to select invasive breast cancer patients for hormone therapy (1). ER and PR analysis was performed on this case by immunohistochemistry utilizing the ER (ER 1D5, 1:100) and PR (PGR 136, 1:100) antibody provided by following the manufacturer's instructions listed in the package insert. This assay was not modified, and adherence to all instruction and guidelines were strictly followed. Interpretation of the ER/PR immunohistochemical staining characteristics is guided by published results in the medical literature (1), information provided by the reagent manufacturer and by internal review of staining performance within the

1. Harvey JM, et al. Estrogen receptor status by immunohistochemistry is superior to the ligand-binding assay for predicting response to adjuvant endocrine therapy in breast cancer. J Clin Oncol. 17:1474-1481, 1999

CLINICAL HISTORY:

Patient is a year old white female who underwent an ultrasound guided core biopsy on which revealed invasive ductal carcinoma of right breast with extensive pleomorphic malignant appearing microcalcifications on mammogram. The patient opted for a right simple mastectomy and sentinel lymph node biopsy after consideration.

PRE-OPERATIVE DIAGNOSIS:

Right breast cancer.

ADDENDUM:

SYNOPTIC REPORT - BREAST HER-2 RESULTS
Specimens Involved
Specimens: F: RIGHT BREAST

HER2 Status Results, Immunohistochemistry Evaluation
SPECIMEN
Surgical Excision
Block Number: Block
F1
Interpretation: Equivocal
Intensity: 2+
% Tumor Staining: 50%
FISH Ordered YES DATE
METHODOLOGY

Methodology: Fixation Type and Length: Tissue was fixed in 10% neutral buffered formalin ( ) for no less than 8 and no longer than 24 hours. Antibody and Assay Methodology: Rabbit anti-human HER2, HerceptestTM (FDA-approved test kit), Control Slides Examined: External kit-slides provided by manufacturer (cell lines with high, low and negative HER2 protein expression), and in-house known HER2 amplified control tissue were evaluated along with the test tissue. These control slides run along side of this patient's sample showed appropriate staining. Adequacy of Specimen: Adequate, well preserved, clear-cut invasive carcinoma identified for HER2 evaluation.

Scoring Criterion and Scoring System:
IHC Level of Expression(Score) /Tumor Cell Membrane Staining Pattern
Negative (0)/Absence of Staining
Negative (1+)/Faint Incomplete membrane Staining, >10% of Cells
Equivocal (2+)/Weak complete membrane Staining, >10% of Cells
Positive (3+)/Strong complete membrane Staining, >10% of Cells

Equivocal Category for HER2 IHC results: A HER2, 2+ staining result that is interpreted as equivocal may not indicate gene amplification. A FISH test for HER2 gene amplification will be ordered for all HER2 IHC 2+ results.

COMMENT

This assay can be used to select invasive breast cancer patients for Trastuzumab (Hereptin) therapy (1,2). Clinical Trials have shown that Trastuzumab substantially increases the likelihood for an objective response and overall survival for patients with metastatic HER2-positive breast cancer, regardless of whether HER2 tumor status was determined as IHC 3+ or FISH positive. Trastuzumab added to adjuvant chemotherapy substantially increase disease-free survival and decreases the risk of disease recurrence by about 50% for patients with early-stage HER2 protein over-expressed or gene amplified invasive breast cancer (3).

HER2 analysis was performed on this case by immunohistochemistry utilizing the FDA approved (TM) test kit following the manufacturer's instructions listed in the package insert. This assay was not modified, and adherence to all instruction and guidelines were strictly followed. Interpretation of the HER2 immunohistochemical staining characteristics is guided by published results in the medical literature (4), information provided by the reagent manufacturer and by internal review of staining performance within the Institute Pathology Department.

HER2 TEST VALIDATION

This HER2 immunohistochemical assay has been validated according to the recently revised recommendations and guidelines from the NCCN HER2 testing in Breast Cancer Task Force, and the jointly issued recommendations and guidelines from ASCO and the CAP (5). 80 randomly selected breast cancer samples were tested for HER2 by IHC as outline above and interpreted as, negative (score 0/1+) equivocal (score 2+) and positive (score 3+) without knowledge of the previous reported results.

These cases were also blindly read using two different FISH assay as amplified or non-amplified and the HER2/CEP17 ratios were recorded. After analyzing these results, there was 100% concordance between the IHC and FISH results for cases that were interpreted as either positive or negative by IHC. 9 of the 80 cases were interpreted as equivocal by IHC and of these 3/9 (33%) were non-amplified by FISH and 6/9 (66%) were found to be amplified.

Pathology Department Immunohistochemistry laboratory takes full responsibility for this tests performance and has programs in place to regularly monitor the proficiency and the interpretation of HER2 assays. The laboratory also participates in external quality assurance HER2 programs including the CAP proficiency testing program.

REFERENCE

1. Carlson RW, Anderson BO, Burstein HJ, et al., NCCN breast cancer clinical practice guidelines in oncology. J Natl Compr Canc Netw. 2005;3:238-289.
2. Carlson RW, Brown E, Burstein HJ, et al., NCCN Task Force Report: adjuvant therapy for breast cancer. J Natl Compr Canc Netw. 2006;4:S1-S26.
3. Romond EH, Perez EA, Bryant J, et al. Trastuzumab plus adjuvant chemotherapy for operable HER2-positive breast cancer. N Eng J Med 2005;353(16):1673-84

4. Leong ASY, Formby M, Haffajee Z, et al. Refinement of immunohistologic parameters for Her2/neu scoring validation by FISH and CISH. Appl Immunohistochem Mol Morphol. 2006;14:384-389.
5. Wolff AC, Hammond EH, Schwartz JN, et al., American Society of Clinical Oncology/College of American Pathologists Guideline Recommendations for Human Epidermal Growth Factor Recepto 2 Testing in Breast Cancer. Arch of Path and Lab Med 2007; 131:18-43.
The followings are ER and PR results of the second tumor focus measuring 1 cm. at the central area of breast tissue.

SYNOPTIC REPORT - BREAST, ER/PR RESULTS
Specimens Involved
Specimens: F: RIGHT BREAST

SPECIMEN:
Surgical Excision
Block Number: F7
ER: Positive - Allred Score: 8 = Proportion score: 5 + Intensity Score 3
PR: Positive - Allred Score: 7 = Proportion Score 4 + Intensity Score 3
COMMENT:
The Allred score for estrogen and progesterone receptors is calculated by adding the sum of the proportion score (0 = no staining, 1 = <1% of cells staining, 2 = 1 - 10% of cells staining, 3 = 11-30% of cells staining, 4 = 31-60% of cells staining, 5 = >60% of cells staining) to the intensity score (1 = weak intensity of staining, 2 = intermediate intensity of staining, 3 = strong intensity of staining), with a scoring range from 0 to 8.
ER/PR positive is defined as an Allred score of >2 and ER/PR negative is defined as an Allred score of less than or equal to 2.
Methodoloav: Fixation Type and Length: Tissue was fixed in 10% neutral buffered formalin ( ) for no less than 8 and no longer than 24 hours. Antibody and Assay Methodology: Mouse anti-human ER and PR, (Dako, Carpenteria, CA).
Comment: This assay can be used to select invasive breast cancer patients for hormone therapy (1). ER and PR analysis was performed on this case by immunohistochemistry utilizing the ER (ER 1D5, 1:100) and PR (PGR 136, 1:100) antibody provided by following the manufacturer's instructions listed in the package insert. This assay was not modified, and adherence to all instruction and guidelines were strictly followed. Interpretation of the ER/PR immunohistochemical staining characteristics is guided by published results in the medical literature (1), information provided by the reagent manufacturer and by internal review of staining performance within the Institute Pathology Department.
1. Harvey JM, et al. Estrogen receptor status by immunohistochemistry is superior to the ligand-binding assay for predicting response to adjuvant endocrine therapy in breast cancer. J Clin Oncol. 17:1474-1481, 1999
SYNOPTIC REPORT - BREAST HER-2 RESULTS
Specimens Involved
Specimens: F: RIGHT BREAST

HER2 Status Results, Immunohistochemistry Evaluation
SPECIMEN
Surgical Excision
Block Number: Block
F7
Interpretation: Negative
Intensity: 1+
% Tumor Staining: 10%
FISH Ordered NO DATE
METHODOLOGY
Methodology: Fixation Type and Length: Tissue was fixed in 10% neutral buffered formalin (Pharmco Inc. Brookfield, CT) for no less than 8 and no longer than 24 hours. Antibody and Assav Methodology: Rabbit anti-human HER2, HerceptestTM (FDA-approved test kit), (Control Slides Examined: External kit-slides provided by manufacturer (cell lines with nign, low and negative HER2 protein expression), and in-house known HER2 amplified control tissue were evaluated along with the test tissue. These control slides run along side of this patient's sample showed appropriate staining. Adequacy of Specimen: Adequate, well preserved, clear-cut invasive carcinoma identified for HER2 evaluation.
Scoring Criterion and Scoring System:
IHC Level of Expression(Score) /Tumor Cell Membrane Staining Pattern
Negative (0)/Absence of Staining
Negative (1+)/Faint Incomplete membrane Staining, >10% of Cells
Equivocal (2+)/Weak complete membrane Staining, >10% of Cells
Positive (3+)/Strong complete membrane Staining, >10% of Cells

Equivocal Category for HER2 IHC results: A HER2, 2+ staining result that is interpreted as equivocal may not indicate gene amplification. A FISH test for HER2 gene amplification will be ordered for all HER2 IHC 2+ results.

COMMENT

This assay can be used to select invasive breast cancer patients for Trastuzumab (Hereptin) therapy (1,2). Clinical Trials have shown that Trastuzumab substantially increases the likelihood for an objective response and overall survival for patients with metastatic HER2-positive breast cancer, regardless of whether HER2 tumor status was determined as IHC 3+ or FISH positive. Trastuzumab added to adjuvant chemotherapy substantially increase disease-free survival and decreases the risk of disease recurrence by about 50% for patients with early-stage HER2 protein over-expressed or gene amplified invasive breast cancer (3).

HER2 analysis was performed on this case by immunohistochemistry utilizing the FDA approved ,TM) test kit following the manufacturer's instructions listed in the package insert. This assay was not modified, and adherence to all instruction and guidelines were strictly followed. Interpretation of the HER2 immunohistochemical staining characteristics is guided by published results in the medical literature (4), information provided by the reagent manufacturer and by internal review of staining performance within the Pathology Department.

HER2 TEST VALIDATION

This HER2 immunohistochemical assay has been validated according to the recently revised recommendations and guidelines from the NCCN HER2 testing in Breast Cancer Task Force, and the jointly issued recommendations and guidelines from ASCO and the CAP (5). 80 randomly selected breast cancer samples were tested for HER2 by IHC as outline above and interpreted as, negative (score 0/1+) equivocal (score 2+) and positive (score 3+) without knowledge of the previous reported results.

These cases were also blindly read using two different FISH assay as amplified or non-amplified and the HER2/CEP17 ratios were recorded. After analyzing these results, there was 100% concordance between the IHC and FISH results for cases that were interpreted as either positive or negative by IHC. 9 of the 80 cases were interpreted as equivocal by IHC and of these 3/9 (33%) were non-amplified by FISH and 6/9 (66%) were found to be amplified.

Institute Pathology Department Immunohistochemistry laboratory takes full responsibility for this tests performance and has programs in place to regularly monitor the proficiency and the interpretation of HER2 assays. The laboratory also participates in external quality assurance HER2 programs including the CAP proficiency testing program.

REFERENCE

1. Carlson RW, Anderson BO, Burstein HJ, et al., NCCN breast cancer clinical practice guidelines in oncology. J Natl Compr Canc Netw. 2005;3:238-289.
2. Carlson RW, Brown E, Burstein HJ, et al., NCCN Task Force Report: adjuvant therapy for breast cancer. J Natl Compr Canc Netw. 2006;4:S1-S26.
3. Romond EH, Perez EA, Bryant J, et al. Trastuzumab plus adjuvant chemotherapy for operable HER2-positive breast cancer. N Eng J Med 2005;353(16):1673-84
4. Leong ASY, Formby M, Haffajee Z, et al. Refinement of immunohistologic parameters for Her2/neu scoring validation by FISH and CISH. Appl Immunohistochem Mol Morphol. 2006;14:384-389.
5. Wolff AC, Hammond EH, Schwartz JN, et al., American Society of Clinical Oncology/College of American Pathologists Guideline Recommendations for Human Epidermal Growth Factor Recepto 2 Testing in Breast Cancer. Arch of Path and Lab Med 2007; 131:18-43.

PathVysion HER-2 DNA Probe Kit

Analytical Interpretation of Results: HER-2 NOT AMPLIFIED

Clinical Interpretation of results

Amplification of the HER-2 gene was evaluated with interphase fluorescence in-situ hybridization (FISH) on formalin-fixed paraffin embedded tissue sections using a chromosome 17 centromeric probe and a HER-2 probe that spans the entire HER-2 gene in the Pathology Core Facility by Dr.. A majority of tumors cells displayed moderate polysomy 17 with 2 to 4 chromosome 17 signals and 2 to 4 HER-2 signals, with a HER-2/CEP 17 Ratio </=2.0, consistent with no amplification of the HER2/neu gene.

Block used F1 Source of case: RPCI
Tissue fixation formalin-fixed tissue Outside Case No: NA
Tissue source breast Results interpreted: yes
HER2/CEP17 ratio: 1.46

This ratio is derived by dividing the total number of LSI HER-2/neu signals by the total number of CEP17 signals in at least 20 interphase nuclei with nonoverlapping nuclei in the neoplastic mammary epithelial cells. Cells with no signals or with signals of only one color are disregarded.

Method of ratio enumeration: manual count

Limitations

The Vysis PathVysion Kit is not intended for use to screen for or diagnose breast cancer. It is intended to be used as an adjunct to other prognostic factors currently used to predict disease-free and overall survival in stage II, node-positive breast cancer patients. In making decisions regarding adjuvant CAF treatment, all other available clinical information should also be taken into consideration, such as tumor size, number of involved lymph nodes, and steroid receptor status.

No

treatment decision for stage II, node-positive breast cancer patients should be based on HER-2/neu gene amplification status alone.

Overview of this test

FDA APPROVED REAGENT

PathVysion HER-2 DNA Probe Kit is FDA approved for selection of patients for whom Herceptin® therapy is being considered. These tests were performed in the ', under the direction of Dr.. The results of these studies should always be interpreted in the context of the clinical, morphological, and immunophenotypic diagnosis.

Gross Dictation:, M.D., Pathologist,
Microscopic/Diagnostic Dictation: M.D., Pathologist.
Final Review: M.D., Pathologist,
Final: M.D., Pathologist, 1
Addendum:., Pathologist,
Addendum Final:., Pathologist,
Addendum: M.D., Pathologist, 1
Addendum Final: M.D., Pathologist,
Addendum:, M.D., Pathologist,
Addendum Final:, M.D., Pathologist, 1
Addendum:., Pathologist,
Addendum Final:, M.D., Pathologist,
Addendum:, M.D., Pathologist,
Addendum Final:, M.D., Pathologist,

Criteria	Yes	No
Diagnosis Discrepancy		✓
Primary Tumor Site Discrepancy		✓
HIPAA Discrepancy		✓
Prior Malignancy History		✓
Dual/Synchronous Primary Noted		✓
Case is (circle): QUALIFIED / DISQUALIFIED		
Reviewer Initials [illegible] Date Reviewed: [illegible]		

ICD-0-3
Carcinoma, infiltrating duct, NOS
8500/3 12/8/10
lw
Site Code: breast, NOS C50.9

UUID:8F8DCA87-B27A-429C-952A-186F8D4502AE
TCGA-E2-A154-01A-PR
Redacted

TSS:

SPECIMENS:
A. SENTINEL LYMPH NODE #1 LEFT AXILLA
B. SENTINEL LYMPH NODE #2 LEFT AXILLA
C. LEFT BREAST WLE

SPECIMEN(S):
A. SENTINEL LYMPH NODE #1 LEFT AXILLA
B. SENTINEL LYMPH NODE #2 LEFT AXILLA
C. LEFT BREAST WLE

GROSS DESCRIPTION:
A. SENTINEL LYMPH NODE #1 LEFT AXILLA
Received fresh labeled with the patient's identification and "SLN #1, left axilla" is a 2.0 x 1.5 x 0.5 cm possible lymph node. The tissue is serially sectioned and touch imprints are performed. The specimen is entirely submitted, A1-A2.
B. SENTINEL LYMPH NODE #2 LEFT AXILLA
Received fresh labeled with the patient's identification and "SLN #2, left axilla" is a 1.6 x 1.4 x 0.4 cm possible lymph node. The tissue is serially sectioned and touch imprints are performed. The specimen is entirely submitted, B1.
C. LEFT BREAST
Received fresh and subsequently placed in formalin labeled with the patient's identification and "left breast" is an oriented (short-superior, long-lateral) 26 gm, 5.8 x 5.1 x 1.8 cm lumpectomy. Ink code (previously inked in OR): anterior-yellow, posterior-black, medial-green, lateral-red, superior-blue, inferior-orange. The specimen is serially sectioned into seven slices revealing a 1.5 x 1.5 x 1.3 cm stellate, gritty white tumor that is closest to the anterior margin at less than 0.1 cm. Tissue is procured. Also, identified is a second 0.5 x 0.4 x 0.2 cm, firm tan-white, suspicious nodule that is closest to the inferior margin at 0.2 cm. The second nodule is at least 0.4 cm inferior to the tumor. No additional masses are identified. Representative sections:
C1-C2-slice 7, inferior margin, perpendicular
C3-slice 6, tumor-lateral half
C4-slice 6, tumor-medial half
C5-slice 5, tumor to anterior margin
C6-C7-slice 4, tumor to anterior and posterior margins
C8-slice 2, representative posterior and lateral margins
C9-slice 1, superior margin, perpendicular

DIAGNOSIS:
A. LYMPH NODE, SENTINEL #1, LEFT AXILLA, EXCISION:
- ONE LYMPH NODE, NEGATIVE FOR METASTASES (0/1).

B. LYMPH NODE, SENTINEL #2, LEFT AXILLA, EXCISION:
- ONE LYMPH NODE, NEGATIVE FOR METASTASES (0/1).

C. BREAST, LEFT, WIDE LOCAL EXCISION:
- INVASIVE DUCTAL CARCINOMA, SBR GRADE 2, MEASURING 1.2-CM
- SATELLITE INVASIVE TUMOR, MEASURING 1 MM, PRESENT 2.5 MM FROM POSTERIOR
- INVASIVE TUMOR PRESENT AT ANTERIOR SURGICAL RESECTION MARGIN
SURGICAL RESECTION MARGIN
- INTERMEDIATE NUCLEAR GRADE, DUCTAL CARCINOMA IN SITU, CRIBRIFORM TYPE WITH FOCAL NECROSIS
- SEE SYNOPTIC REPORT.

SYNOPTIC REPORT - BREAST
Specimen Type: Excision
Needle Localization: No
Laterality: Left
Invasive Tumor: Present
Multifocality: Yes
WHO CLASSIFICATION
Invasive ductal carcinoma, NOS 8500/3
Tumor size: 1.2cm
Margins: Involved at
anterior
Extent:: 4 mm
Tubular Score: 2

- SURGICAL RESECTION MARGINS [illegible]

- METASTATIC CARCINOMA TO ONE OF TWELVE AXILLARY LYMPH NODES (1/12), MEASURING 3.5-CM WITH FOCAL EXTRANODAL EXTENSION
- METASTATIC CARCINOMA TO ONE OF TWO INTRAMAMMARY LYMPH NODES (1/2), MEASURING 0.8-CM WITH FOCAL EXTRANODAL EXTENSION
- SEE SYNOPTIC REPORT AND SEE NOTE.

B. LYMPH NODES, ADDITIONAL LEFT AXILLARY CONTENTS, DISSECTION:
- ONE LYMPH NODE, NEGATIVE FOR METASTASES (0/1).

NOTE: Four tumor nodules are grossly identified. Microscopically 3 of them are invasive ductal carcinoma that have the same morphology and one is DCIS. The tissues in between these nodules show scattered foci of DCIS. Therefore, these foci may be interconnected through DCIS. Largest confluent invasive tumor measures 2.5-cm.

Two intramammary lymph nodes are identified, one is positive for metastatic carcinoma.

SYNOPTIC REPORT - BREAST
Specimen Type: Mastectomy
Needle Localization: No
Laterality: Left
Invasive Tumor: Present
Multifocality: Yes
WHO CLASSIFICATION
Invasive ductal carcinoma, NOS 8500/3
Tumor size: 2.5cm
Tumor Site: Upper inner quadrant
Lower inner quadrant
Margins: Negative
Tubular Score: 3
Nuclear Grade: 2
Mitotic Score: 3
Modified Scarff Bloom Richardson Grade: 3
Necrosis: Absent
Vascular/Lymphatic Invasion: Indeterminate
Lobular neoplasia: None
Lymph nodes: Axillary dissection
Lymph node status: Positive 2 / 15 Extranodal extension

DCIS present
DCIS Quantity: Estimate 10%
DCIS Type: Solid
Cribriform
DCIS Location: Associated with invasive tumor
Nuclear grade: High
Necrosis: Present

ER/PR/HER2 Results
ER: Positive
PR: Negative
HER2: Negative by IHC
Performed on Case:

Pathological staging (pTN): pT 2 N 1c

CLINICAL HISTORY:
None given

PRE-OPERATIVE DIAGNOSIS:
Left breast cancer

Microscopic/Diagnostic Dictation: Pathologist
Final Review: Pathologist, [illegible]
Final: Pathologist, [illegible]

Criteria	Yes	No
Diagnosis Discrepancy		✓
Primary Tumor Site Discrepancy		✓

UUID:1CEBF20D-767A-4A19-AA41-B9EAFCF05176
TCGA-E2-A158-01A-PR Redacted

ICD-0-3
Carcinoma, infiltrating duct, NOS
8500/3 12/8/10
Path Site Code: breast, upper outer quadrant C50.4
CQCF Site: breast, NOS C50.9

TSS:

SPECIMENS:

A. SENTINEL LYMPH NODE #1 LEFT AXILLA
B. SENTINEL LYMPH NODE #2 LEFT AXILLA
C. LEFT BREAST
D. SENTINEL LYMPH NODE #1 RIGHT AXILLA
E. RIGHT BREAST

SPECIMEN(S):

A. SENTINEL LYMPH NODE #1 LEFT AXILLA
B. SENTINEL LYMPH NODE #2 LEFT AXILLA
C. LEFT BREAST
D. SENTINEL LYMPH NODE #1 RIGHT AXILLA
E. RIGHT BREAST

INTRAOPERATIVE CONSULTATION DIAGNOSIS:

TPA, Sentinel lymph node #1, left axilla: Negative for carcinoma
TPB, Sentinel lymph node #2, left axilla: Negative for carcinoma
Diagnosis called at by Dr.
TPC, Sentinel lymph node #1, right axilla: negative for carcinoma
Diagnosis called at by Dr.

GROSS DESCRIPTION:

A. SENTINEL LYMPH NODE #1 LEFT AXILLA
Received fresh labeled with the patient's identification and designated "sentinel lymph node number one left axilla" is a fragment of fibroadipose tissue, 1.4 x 1 x 0.6 cm, consisting of one possible lymph node, 1.4 x 0.7 x 0.4 cm. Touch preparation is performed. The entire lymph node is submitted, A1.
B. SENTINEL LYMPH NODE #2 LEFT AXILLA
Received fresh labeled with the patient's identification and designated "sentinel lymph node number two left axilla" is a fragment of fibroadipose tissue, 2 x 1.4 x 0.8 cm, consisting of one possible lymph node, 2 x 0.9 x 0.5 cm. Touch preparation is performed. The entire lymph node is submitted, B1.
C. LEFT BREAST
Received fresh labeled with the patient's identification and designated "left breast" is an oriented (suture in axilla), 573-g, 25 x 18 x 4.5 cm mastectomy specimen with 3.5 x 2 cm light tan skin ellipse, and 1.4-cm diameter everted nipple. Ink code: Posterior-black, anterior/superior-blue, anterior/inferior-orange. The specimen is serially sectioned from lateral to medial revealing a tan stellate mass in the UOQ (slices 3-4), 3 x 2.8 x 1.5 cm, located 0.2-cm from the nearest anterior margin, and 2.2-cm from the deep margin. A smaller well defined nodule present in the posterior UIQ is seen. A portion the specimen is submitted for tissue procurement. Representatively submitted:
C1-C3: Nipple, C3 contains representative section of skin
C4: Mass with anterior margin, slice 3, UOQ
C5: Mass with anterior margin, slice 4, UOQ
C6-C7: Remainder of mass, slice 4, UOQ
C8: Deep margin, slice 4, UOQ
C9: Additional section, UOQ, slice 5
C10: Representative section, LOQ, slice 5
C11-C12: Representative sections, UIQ, slices 7-8, respectively
C13: Representative section, LIQ, slice 7
C14-C15: nodule in posterior UIQ

D. SENTINEL LYMPH NODE #1 RIGHT AXILLA
Received fresh labeled with matching patient identifiers is a piece of adipose tissue 3.4 x 3 x 1.1 cm containing two lymph nodes, the smaller is 0 .5 cm in diameter, larger one measures 2.4 x 0.8 x 0.8 cm. Touch preps are performed the specimen is submitted entirely/separately in cassettes D1-D2.
E. RIGHT BREAST
Received fresh labeled with the patient's identification and "right breast" is an oriented 454 g, 19 x 16 x 3 cm mastectomy with 3 x 3 cm skin ellipse and 1.8 cm everted nipple. Ink code: Anterior/superior-blue, anterior/inferior-orange, posterior-black. Specimen is serially sectioned into 9 slices from medial to the lateral with nipple in slice 4 revealing 4 lesions.
1- 1.3 x 0.8 x 0.5 cm firm tan mass located in slics 5-6 in the upper outer quadrant; 2.7 cm from the deep margin and 0.2 cm from the anterior margin
2- 1.2 x 0.9 x 0.8 cm firm tan stellate mass with central area of hemorrhage located in the upper mid-quadrant; 2.5 cm from the deep margin, 1.8 cm from the anterior margin, 1.4 cm the medial to lesion #1, and 1.8 cm posterior/superior from nipple
3- 0.6 x 0.3 x 0.3 cm firm tan nodule located in the upper inner quadrant; 1.2 cm from the deep margin, 0.9 cm from the anterior margin and 3.2 cm medial to lesion #2

4- 0.6 x 0.4 x 0.2 cm firm tan nodule located in the lower inner quadrant; 1.2 cm from the deep margin, 0.6 cm from the anterior margin, and 6.2 cm inferior to lesion #1
Representatively submitted.
E1: slice 1, upper inner
E2-E3: slice 2, lesion #3(posterior to anterior)
E4: slice 3, tissue connecting lesion #3 and #2
E5-E7: slice 4, lesion #2 submitted anterior to posterior
E8: slice 5, lesion #1
E9: slice, 6, lesion #1
E10: slice 7, upper outer (lateral to lesion #1)
E11: slice 7, lower outer
E12: slice 6, lower outer
E13-E14: slice 5, lesion #4 (anterior to posterior)
E15-E16: slice 5, tissue connecting lesions #1 and #4
E17: slice 3, lower inner
E18: slice 2, lower inner
E19-E21: nipple, perpendicular sections
E22: skin

DIAGNOSIS:

A. LYMPH NODE, SENTINEL #1, LEFT AXILLA, EXCISION:
- ONE LYMPH NODE, NEGATIVE FOR METASTASES (0/1).

B. LYMPH NODE, SENTINEL #2, LEFT AXILLA, EXCISION:
- METASTATIC CARCINOMA TO ONE OF ONE LYMPH NODE (1/1), MEASURING 0.1-CM (MICROMETASTASES) WITH NO EXTRANODAL EXTENSION, SEE NOTE.

C. BREAST, LEFT, MASTECTOMY:
- TWO FOCI OF INVASIVE DUCTAL CARCINOMA
- SBR GRADE 3, MEASURING 1.6-CM
- SBR GRADE 1, MEASURING 0.5-CM
- INTERMEDIATE NUCLEAR GRADE, DUCTAL CARCINOMA IN SITU, SOLID TYPE WITH CENTRAL NECROSIS AND MICROCALCIFICATIONS
- SURGICAL RESECTION MARGINS NEGATIVE FOR TUMOR
- BIOPSY SITE CHANGES WITH FIBROSIS AND GRANULATION TISSUE
- SEE SYNOPTIC REPORT AND SEE NOTE.

D. LYMPH NODE, SENTINEL #1, RIGHT AXILLA, EXCISION:
- ONE LYMPH NODE, NEGATIVE FOR METASTASES (0/1).

E. BREAST, RIGHT, MASTECTOMY:
- TWO FOCI OF INVASIVE DUCTAL CARCINOMA,
- SBR GRADE 3, MEASURING 1.1-CM
- SBR GRADE 2, MEASURING 0.6-CM
- INTERMEDIATE NUCLEAR GRADE, DUCTAL CARCINOMA IN SITU, SOLID AND CRIBRIFORM TYPES
- SURGICAL RESECTION MARGINS NEGATIVE FOR TUMOR
- BIOPSY SITE CHANGES WITH FIBROSIS
- FIBROADENOMA AND SCLEROSING ADENOSIS
- SEE SYNOPTIC REPORT AND SEE NOTE.

NOTE: Left axillary sentinel lymph node #2 touch preparation is negative. Therefore, the false- negativity is due to sampling error. The morphology of metastatic tumor is similar to the larger grade 3 tumor (see below).

In the left mastectomy specimen, 2 nodules are grossly identified, larger nodule located in UOQ measuring 1.6 and is of grade 3; and a smaller nodule, located in posterior UIQ, measuring 0.5-cm and is of grade 1. Breast biomarkers on both nodules are pending.

In the right mastectomy specimen, 4 nodules are grossly identified, one is fibroadenoma, one is sclerosing adenosis, and the other two are separate foci of invasive ductal carcinoma. The largest invasive tumor measures 1.1-cm and it is of grade 3. Breast biomarkers are as follows, ER negative, PR negative and HER-2/neu equivocal (2+, FISH pending). The smaller nodule measures 0.6-cm and it is of grade 2. The breast biomarkers are as follows ER positive, PR positive and HER-2/neu equivocal (2+, FISH pending).

Also, the morphology of grade 3 tumors (right and left) is different. It seems that there are 4 different primary tumors, 2 in each breast.

SYNOPTIC REPORT - BREAST

Specimens Involved
Specimens: B: SENTINEL LYMPH NODE #2 LEFT AXILLA
C: LEFT BREAST

Specimen Type: Mastectomy
Needle Localization: No
Laterality: Left
Invasive Tumor: Present
Multifocality: Yes
WHO CLASSIFICATION
Invasive ductal carcinoma, NOS 8500/3
Tumor size: 1.6cm
Tumor Site: Upper outer quadrant
Upper inner quadrant
Margins: Negative
Tubular Score: 3
Nuclear Grade: 2
Mitotic Score: 3
Modified Scarff Bloom Richardson Grade: 3
Necrosis: Present
Vascular/Lymphatic Invasion: None identified
Lobular neoplasia: None
Lymph nodes: Sentinel lymph node only
Lymph node status: Positive 1 / 2
Micrometastases: Yes

DCIS present
Margins uninvolved by DCIS
DCIS Quantity: Estimate 5%
DCIS Type: Solid
DCIS Location: Associated with invasive tumor
Nuclear grade: Intermediate
Necrosis: Present

ER/PR/HER2 Results
Performed on Case: see note

Pathological staging (pTN): pT 1c N 1mic

SYNOPTIC REPORT - BREAST
Specimens Involved
Specimens: D: SENTINEL LYMPH NODE #1 RIGHT AXILLA
E: RIGHT BREAST

Specimen Type: Mastectomy
Needle Localization: No
Laterality: Right
Invasive Tumor: Present
Multifocality: Yes
WHO CLASSIFICATION
Invasive ductal carcinoma, NOS 8500/3
Tumor size: 1.1cm
Margins: Negative
Tubular Score: 3
Nuclear Grade: 3
Mitotic Score: 2
Modified Scarff Bloom Richardson Grade: 3
Necrosis: Present
Vascular/Lymphatic Invasion: None identified
Lobular neoplasia: None
Lymph nodes: Sentinel lymph node only
Lymph node status: Negative 0 / 1

DCIS present
Margins uninvolved by DCIS
DCIS Quantity: Estimate 2%
DCIS Type: Solid
Cribriform
DCIS Location: Associated with invasive tumor

Nuclear grade: Intermediate
Necrosis: Absent

ER/PR/HER2 Results
Performed on Case: see note

Pathological staging (pTN): pT 1c N 0

SYNOPTIC REPORT - BREAST, ER/PR RESULTS
Specimens Involved
Specimens: E: RIGHT BREAST

Specimen: Surgical Excision
Block Number: E5, larger tumor

ER: Negative Allred Score: 0 = Proportion Score 0 + Intensity Score 0
PR: Negative Allred Score: 0 = Proportion Score 0 + Intensity Score 0

COMMENT:
The Allred score for estrogen and progesterone receptors is calculated by adding the sum of the proportion score (0 = no staining, 1 = <1% of cells staining, 2 = 1 - 10% of cells staining, 3 = 11-30% of cells staining, 4 = 31-60% of cells staining, 5 = >60% of cells staining) to the intensity score (1 = weak intensity of staining, 2 = intermediate intensity of staining, 3 = strong intensity of staining), with a scoring range from 0 to 8.
ER/PR positive is defined as an Allred score of >2 and ER/PR negative is defined as an Allred score of less than or equal to 2.
METHODOLOGY:
Tissue was fixed in 10% neutral buffered formalin for no less than 8 and no longer than 24 hours. Immunohistochemistry was performed using the mouse anti-human ER (ER 1D5, 1:100) and PR (PGR 136, 1:100) provided by Dako) following the manufacturer s instructions. This assay was not modified. Interpretation of the ER/PR immunohistochemical stain is guided by published results in the medical literature, information provided by the reagent manufacturer and by internal review of staining performance.

SYNOPTIC REPORT - BREAST HER-2 RESULTS
Specimens Involved
Specimens: E: RIGHT BREAST

Specimen: Surgical Excision
Block Number: E5 larger tumor

Interpretation: EQUIVOCAL
Intensity: 2+
% Tumor Staining: 10%
Fish Ordered: Yes , on Date

METHODOLOGY:
Tissue was fixed in 10% neutral buffered formalin for no less than 8 and no longer than 24 hours. Her2 analysis was performed using the FDA approved Dako HercepTest (TM) test kit (Dako, Carpenteria, CA) using rabbit anti-human HER2. This assay was not modified. External kit-slides provided by the manufacturer (cell lines with high, low and negative HER2 protein expression) and in-house known HER2 amplified control tissue were evaluated along with the test tissue. Adequate, well preserved, clear-cut invasive carcinoma was identified for HER2 evaluation. Interpretation of the HER2 immunohistochemical stain is guided by published results in the medical literature, information provided by the reagent manufacturer and by internal review of staining performance.
This assay has been validated according to the 2007 joint recommendations and guidelines from ASCO and CAP and from the NCCN HER2 testing in Breast Cancer Task Force. The Pathology Department takes full responsibility for this test's performance.

SYNOPTIC REPORT - BREAST, ER/PR RESULTS
Specimens Involved
Specimens: E: RIGHT BREAST

Specimen: Surgical Excision
Block Number: E13 smaller tumor

ER: Positive Allred Score: 8 = Proportion Score 5 + Intensity Score 3
PR: Positive Allred Score: 8 = Proportion Score 5 + Intensity Score 3

COMMENT:

The Allred score for estrogen and progesterone receptors is calculated by adding the sum of the proportion score (0 = no staining, 1 = <1% of cells staining, 2 = 1 - 10% of cells staining, 3 = 11-30% of cells staining, 4 = 31-60% of cells staining, 5 = >60% of cells staining) to the intensity score (1 = weak intensity of staining, 2 = intermediate intensity of staining, 3 = strong intensity of staining), with a scoring range from 0 to 8.

ER/PR positive is defined as an Allred score of >2 and ER/PR negative is defined as an Allred score of less than or equal to 2.

METHODOLOGY:

Tissue was fixed in 10% neutral buffered formalin for no less than 8 and no longer than 24 hours. Immunohistochemistry was performed using the mouse anti-human ER (ER 1D5, 1:100) and PR (PGR 136, 1:100) provided by Dako) following the manufacturer s instructions. This assay was not modified. Interpretation of the ER/PR immunohistochemical stain is guided by published results in the medical literature, information provided by the reagent manufacturer and by internal review of staining performance.

SYNOPTIC REPORT - BREAST HER-2 RESULTS
Specimens Involved
Specimens: E: RIGHT BREAST

Specimen: Surgical Excision
Block Number: E13 larger tumor

Interpretation: EQUIVOCAL
Intensity: 2+
% Tumor Staining: 50%
Fish Ordered: Yes , on Date

METHODOLOGY:

Tissue was fixed in 10% neutral buffered formalin for no less than 8 and no longer than 24 hours. Her2 analysis was performed using the FDA approved Dako HercepTest (TM) test kit using rabbit anti-human HER2. This assay was not modified. External kit-slides provided by the manufacturer (cell lines with high, low and negative HER2 protein expression) and in-house known HER2 amplified control tissue were evaluated along with the test tissue. Adequate, well preserved, clear-cut invasive carcinoma was identified for HER2 evaluation. Interpretation of the HER2 immunohistochemical stain is guided by published results in the medical literature, information provided by the reagent manufacturer and by internal review of staining performance.

This assay has been validated according to the 2007 joint recommendations and guidelines from ASCO and CAP and from the NCCN HER2 testing in Breast Cancer Task Force. The Pathology Department takes full responsibility for this test's performance.

CLINICAL HISTORY:

Bilateral invasive breast carcinoma

PRE-OPERATIVE DIAGNOSIS:

Same

INTRAOPERATIVE CONSULTATION DIAGNOSIS:

TPD, Sentinel lymph node #1, right axilla: negative for carcinoma
Diagnosis called at .m. by Dr

ADDENDUM:

Results for touch prep on specimen D was incorrectly designated in the Intraoperative Consultation Diagnosis above as "TPC". Correct information is as follows:
SYNOPTIC REPORT - BREAST, ER/PR RESULTS
Specimens Involved
Specimens: C: LEFT BREAST

Specimen: Surgical Excision
Block Number: C4 larger tumor

ER: Negative Allred Score: 0 = Proportion Score 0 + Intensity Score 0
PR: Negative Allred Score: 0 = Proportion Score 0 + Intensity Score 0

COMMENT:

The Allred score for estrogen and progesterone receptors is calculated by adding the sum of the proportion score (0 = no staining, 1 = <1% of cells staining, 2 = 1 - 10% of cells staining, 3 = 11-30% of cells staining, 4 = 31-60% of cells staining, 5 = >60% of cells staining) to the intensity score (1 = weak intensity of staining, 2 = intermediate intensity of staining, 3 = strong intensity of staining), with a scoring range from 0 to 8.

ER/PR positive is defined as an Allred score of >2 and ER/PR negative is defined as an Allred score of less than or equal to 2.

METHODOLOGY:

Tissue was fixed in 10% neutral buffered formalin for no less than 8 and no longer than 24 hours. Immunohistochemistry was performed using the mouse anti-human ER (ER 1D5, 1:100) and PR (PGR 136, 1:100) provided by Dako following the manufacturer s instructions. This assay was not modified. Interpretation of the ER/PR immunohistochemical stain is guided by published results in the medical literature, information provided by the reagent manufacturer and by internal review of staining performance.

SYNOPTIC REPORT - BREAST HER-2 RESULTS
Specimens Involved
Specimens: C: LEFT BREAST

Specimen: Surgical Excision
Block Number: C4 larger tumor

Interpretation: NEGATIVE
Intensity: 1+
% Tumor Staining: 5%
Fish Ordered: No

METHODOLOGY:

Tissue was fixed in 10% neutral buffered formalin for no less than 8 and no longer than 24 hours. Her2 analysis was performed using the FDA approved Dako HercepTest (TM) test kit using rabbit anti-human HER2. This assay was not modified. External kit-slides provided by the manufacturer (cell lines with high, low and negative HER2 protein expression) and in-house known HER2 amplified control tissue were evaluated along with the test tissue. Adequate, well preserved, clear-cut invasive carcinoma was identified for HER2 evaluation. Interpretation of the HER2 immunohistochemical stain is guided by published results in the medical literature, information provided by the reagent manufacturer and by internal review of staining performance.

This assay has been validated according to the 2007 joint recommendations and guidelines from ASCO and CAP and from the NCCN HER2 testing in Breast Cancer Task Force. The Pathology Department takes full responsibility for this test's performance.

SYNOPTIC REPORT - BREAST, ER/PR RESULTS
Specimens Involved
Specimens: C: LEFT BREAST

Specimen: Surgical Excision
Block Number: C14 smaller tumor

ER: Positive Allred Score: 8 = Proportion Score 5 + Intensity Score 3
PR: Positive Allred Score: 8 = Proportion Score 5 + Intensity Score 3

COMMENT:

The Allred score for estrogen and progesterone receptors is calculated by adding the sum of the proportion score (0 = no staining, 1 = <1% of cells staining, 2 = 1 - 10% of cells staining, 3 = 11-30% of cells staining, 4 = 31-60% of cells staining, 5 = >60% of cells staining) to the intensity score (1 = weak intensity of staining, 2 = intermediate intensity of staining, 3 = strong intensity of staining), with a scoring range from 0 to 8.

ER/PR positive is defined as an Allred score of >2 and ER/PR negative is defined as an Allred score of less than or equal to 2.

METHODOLOGY:

Tissue was fixed in 10% neutral buffered formalin for no less than 8 and no longer than 24 hours. Immunohistochemistry was performed using the mouse anti-human ER (ER 1D5, 1:100) and PR (PGR 136, 1:100) provided by Dako following the manufacturer s instructions. This assay was not modified. Interpretation of the ER/PR immunohistochemical stain is guided by published results in the medical literature, information provided by the reagent manufacturer and by internal review of staining performance.

SYNOPTIC REPORT - BREAST HER-2 RESULTS
Specimens Involved
Specimens: C: LEFT BREAST

Specimen: Surgical Excision
Block Number: C14 smaller tumor

Interpretation: EQUIVOCAL
Intensity: 2+
% Tumor Staining: 10%
Fish Ordered: Yes , on Date

METHODOLOGY:

Tissue was fixed in 10% neutral buffered formalin for no less than 8 and no longer than 24 hours. Her2 analysis was performed using the FDA approved Dako HercepTest (TM) test kit (Dako, Carpenteria, CA) using rabbit anti-human HER2. This assay was not modified. External kit-slides provided by the manufacturer (cell lines with high, low and negative HER2 protein expression) and in-house known HER2 amplified control tissue were evaluated along with the test tissue. Adequate, well preserved, clear-cut invasive carcinoma was identified for HER2 evaluation. Interpretation of the HER2 immunohistochemical stain is guided by published results in the medical literature, information provided by the reagent manufacturer and by internal review of staining performance.

This assay has been validated according to the 2007 joint recommendations and guidelines from ASCO and CAP and from the NCCN HER2 testing in Breast Cancer Task Force. The Pathology Department takes full responsibility for this test's performance.

PathVysion HER-2 DNA Probe Kit

Case No

Analytical Interpretation of Results: HER-2 NOT AMPLIFIED

Clinical Interpretation of results

Amplification of the HER-2 gene was evaluated with interphase fluorescence in-situ hybridization (FISH) on formalin-fixed paraffin embedded tissue sections using a chromosome 17 centromeric probe and a HER-2 probe that spans the entire HER-2 gene in the by Dr. A majority of tumors cells displayed 2 chromosome 17 signals and 2 HER-2 signals, with a HER-2/CEP 17 Ratio </=2.0, consistent with no amplification of the HER2/neu gene.

Block used E5 Source of case:

Tissue fixation formalin-fixed tissue Outside Case No: NA

Tissue source breast Results interpreted: yes

HER2/CEP17 ratio: 1.03

This ratio is derived by dividing the total number of LSI HER-2/neu signals by the total number of CEP17 signals in at least 20 interphase nuclei with nonoverlapping nuclei in the neoplastic mammary epithelial cells. Cells with no signals or with signals of only one color are disregarded.

Method of ratio enumeration: manual count

Limitations

The Vysis PathVysion Kit is not intended for use to screen for or diagnose breast cancer. It is intended to be used as an adjunct to other prognostic factors currently used to predict disease-free and overall survival in stage II, node-positive breast cancer patients. In making decisions regarding adjuvant CAF treatment, all other available clinical information should also be taken into consideration, such as tumor size, number of involved lymph nodes, and steroid receptor status. No treatment decision for stage II, node-positive breast cancer patients should be based on HER-2/neu gene amplification status alone.

Overview of this test

FDA APPROVED REAGENT

PathVysion HER-2 DNA Probe Kit is FDA approved for selection of patients for whom Herceptin® therapy is being considered. These tests were performed in the under the direction of Dr. The results of these studies should always be interpreted in the context of the clinical, morphological, and immunophenotypic diagnosis.

PathVysion HER-2 DNA Probe Kit

Case No

Analytical Interpretation of Results: HER-2 NOT AMPLIFIED

Clinical Interpretation of results

Amplification of the HER-2 gene was evaluated with interphase fluorescence in-situ hybridization (FISH) on formalin-fixed paraffin embedded tissue sections using a chromosome 17 centromeric probe and a HER-2 probe that spans the entire HER-2 gene in the by Dr. A majority of tumors cells displayed 2 chromosome 17 signals and 2 HER-2 signals, with a HER-2/CEP 17 Ratio </=2.0, consistent with no amplification of the HER2/neu gene.

Block used E13 Source of case:

Tissue fixation formalin-fixed tissue Outside Case No: NA

Tissue source breast Results interpreted: yes

HER2/CEP17 ratio: 0.91

This ratio is derived by dividing the total number of LSI HER-2/neu signals by the total number of CEP17 signals in at least 20 interphase nuclei with nonoverlapping nuclei in the neoplastic mammary epithelial cells. Cells with no signals or with signals of only one color are disregarded.

Method of ratio enumeration: manual count

Limitations

The Vysis PathVysion Kit is not intended for use to screen for or diagnose breast cancer. It is intended to be used as an adjunct to other prognostic factors currently used to predict disease-free and overall survival in stage II, node-positive breast cancer patients. In making decisions regarding adjuvant CAF treatment, all other available clinical information should also be taken into consideration, such as tumor size, number of involved lymph nodes, and steroid receptor status. No treatment decision for stage II, node-positive breast cancer patients should be based on HER-2/neu gene amplification status alone.

Overview of this test

FDA APPROVED REAGENT

PathVysion HER-2 DNA Probe Kit is FDA approved for selection of patients for whom Herceptin® therapy is being considered. These tests were performed in the under the direction of Dr. The results of these studies should always be interpreted in the context of the clinical, morphological, and immunophenotypic diagnosis.

Case No
Analytical Interpretation of Results: HER-2 NOT AMPLIFIED
Clinical Interpretation of results
Amplification of the HER-2 gene was evaluated with interphase fluorescence in-situ hybridization (FISH) on formalin-fixed paraffin embedded tissue sections using a chromosome 17 centromeric probe and a HER-2 probe that spans the entire HER-2 gene in the by Dr. A majority of tumors cells displayed 2 chromosome 17 signals and 2 HER-2 signals, with a HER-2/CEP 17 Ratio </=2.0, consistent with no amplification of the HER2/neu gene.

Block used C14 Source of case:
Tissue fixation formalin-fixed tissue Outside Case No: NA
Tissue source breast Results interpreted: yes
HER2/CEP17 ratio: 0.94

This ratio is derived by dividing the total number of LSI HER-2/neu signals by the total number of CEP17 signals in at least 20 interphase nuclei with nonoverlapping nuclei in the neoplastic mammary epithelial cells. Cells with no signals or with signals of only one color are disregarded.

Method of ratio enumeration: manual count

Limitations
The Vysis PathVysion Kit is not intended for use to screen for or diagnose breast cancer. It is intended to be used as an adjunct to other prognostic factors currently used to predict disease-free and overall survival in stage II, node-positive breast cancer patients. In making decisions regarding adjuvant CAF treatment, all other available clinical information should also be taken into consideration, such as tumor size, number of involved lymph nodes, and steroid receptor status. No treatment decision for stage II, node-positive breast cancer patients should be based on HER-2/neu gene amplification status alone.

Overview of this test
FDA APPROVED REAGENT
PathVysion HER-2 DNA Probe Kit is FDA approved for selection of patients for whom Herceptin® therapy is being considered. These tests were performed in the under the direction of Dr. The results of these studies should always be interpreted in the context of the clinical, morphological, and immunophenotypic diagnosis.

Gross Dictation:
Microscopic/Diagnostic Dictation:
Final Review: Pathologist,
Final Review: Pathologist,
Final: Pathologist,
Addendum Review: Pathologist
Addendum Final: Pathologist,
Addendum: Pathologist,
Addendum Final: M.D., Pathologist,
Addendum: Pathologist,
Addendum Final: Pathologist,
Addendum: Pathologist,
Addendum Final: Pathologist

Criteria	Yes	No
Diagnosis Discrepancy		/
Primary Tumor Site Discrepancy		/
HIPAA Discrepancy		/
Prior Malignancy History		/
Dual/Synchronous Primary Noted	/	
Case is (circle): QUALIFIED / DISQUALIFIED		
Reviewer Initials: [illegible] Date Reviewed: 11/24/10		

UUID:14EB270E-0AAB-4C12-8D71-ED79076C2E93
TCGA-E2-A573-01A-PR Redacted

SPECIMEN(S): A. RIGHT BREAST PARTIAL MASTECTOMY
B. SLN #1 RIGHT AXILLA
C. ADDITIONAL AXILLARY TISSUE RIGHT AXILLA

CLINICAL HISTORY:
This is a year old female with a 1.4 cm tumor in the right breast, IDC at 11:00. S/P benign MRI biopsy inferior and lateral to this index lesion, not clipped. Here for N/L lumpectomy with SLN biopsy.

INTRAOPERATIVE CONSULTATION DIAGNOSIS:
A. Right breast, partial mastectomy: Tumor is 0.3 cm from anterior margin.
TPB1-TPB4: SLN#1, right axilla, excision: Four lymph nodes, negative for carcinoma.
Diagnosis called to Dr. at by Dr.

ICD-O-3
carcinoma, infiltrating duct, NOS 8500/3
Site: breast, NOS C50.9
[illegible]
11/25/12

PRE-OPERATIVE DIAGNOSIS:
Right breast cancer

GROSS DESCRIPTION:
A. RIGHT BREAST PARTIAL MASTECTOMY
Received fresh in a container labeled with the patients name and "right breast partial mastectomy" is an oriented (straight: superior= 1 clip, long: lateral = 2 clips, double: deep, air knot in axillary tail), previously inked, 130g, 11 x 9 x 3.5 cm partial mastectomy with accompanying radiograph. Ink code: anterior-yellow, posterior-black, medial-green, lateral-red, superior-blue, inferior-orange. The specimen is serially sectioned from superior to inferior into 8 slices revealing a 1.6 x 1.4 x 0.8 cm firm, circumscribed, tan mass that is closest to the anterior margin at 0.3 cm. Tissue is procured. Representatively submitted:
A1: slice 1, superior margin
A2-A3: slice 4, mass with skin and anterior margin, bisected
A4-A5: slice 4, posterior margin underlying mass, bisected
A6-A7: slice 4, anterior and posterior margins, bisected
A8-A9: slice 4, lateral and posterior margins, bisected
A10-A11: slice 4, anterior and medial margins, bisected
A12-A13: slice 4, medial and posterior margins, bisected
A14: slice 5, mass with skin and anterior margin
A15-A16: slice 8, inferior margin
B. SLN #1 RIGHT AXILLA
Received fresh labeled with the patients name and "SLN #1 right axilla" is a 5 x 2 x 0.7 cm aggregate of fatty tissue within which four lymph nodes, 2 x 0.6 x 0.6 cm, 2 x 1 x 0.6 cm, 2 x 1.1 x 0.4 cm, 1.2 x 1 x 0.6 cm are identified. Touch preps are performed. Lymph nodes are entirely submitted:
B1-B2: one lymph node

B3: one lymph node
B4: one lymph node
B5: one lymph node
C. ADDITIONAL AXILLARY TISSUE RIGHT AXILLA
Received in formalin in a container labeled with the patients name and designated "additional axillary tissue" is a 1.7 x 1 x 0.2 cm fragment of soft fatty tissue. The specimen is bisected and entirely submitted.

DIAGNOSIS:

A. BREAST, RIGHT, PARTIAL MASTECTOMY:
- INVASIVE DUCTAL CARCINOMA WITH LYMPHOPLASMACYTIC INFILTRATE AND GEOGRAPHIC NECROSIS, SBR GRADE 3, MEASURING 1.1-CM
- SURGICAL RESECTION MARGINS NEGATIVE FOR TUMOR
- BIOPSY SITE CHANGES WITH FIBROSIS AND FAT NECROSIS
- SEE SYNOPTIC REPORT.

B. LYMPH NODE, SENTINEL #1, RIGHT AXILLA, EXCISION:
- FOUR LYMPH NODES, NEGATIVE FOR METASTASES (0/4).

C. ADDITIONAL AXILLARY TISSUE, RIGHT, AXILLARY DISSECTION:
- FIBROADIPOSE TISSUE, NO TUMOR OR LYMPHOID TISSUE IDENTIFIED.

SYNOPTIC REPORT - BREAST

Specimen Type: Partial mastectomy
Needle Localization: No
Laterality: Right
Invasive Tumor: Present
Multifocality: No
WHO CLASSIFICATION
Invasive ductal carcinoma, NOS 8500/3
Tumor size: 1.1cm
Tumor Site: Upper outer quadrant
Margins: Negative
Distance from closest margin: 0.3cm
anterior
Tubular Score: 3
Nuclear Grade: 3
Mitotic Score: 2
Modified Scarff Bloom Richardson Grade: 3
Necrosis: Present
Vascular/Lymphatic Invasion: None identified
Lobular neoplasia: None
Lymph nodes: Sentinel lymph node
Lymph node status: Negative 0 / 4

DCIS not present

ER/PR/HER2 Results

ER: Pending
PR: Pending
HER2: Pending

Pathological staging (pTN): pT 1c N 0
Pathological staging is based on the AJCC Cancer Staging Manual, 7th Edition

ADDENDUM:

SYNOPTIC REPORT - BREAST, ER/PR RESULTS

Specimen: Surgical Excision
Block Number: A2

ER:	Negative	Allred Score:	0 = Proportion Score 0 + Intensity Score 0
PR:	Negative	Allred Score:	0 = Proportion Score 0 + Intensity Score 0

COMMENT:

The Allred score for estrogen and progesterone receptors is calculated by adding the sum of the proportion score (0 = no staining, 1 = <1% of cells staining, 2 = 1 - 10% of cells staining, 3 = 11-30% of cells staining, 4 = 31-60% of cells staining, 5 = >60% of cells staining) to the intensity score (1 = weak intensity of staining, 2 = intermediate intensity of staining, 3 = strong intensity of staining), with a scoring range from 0 to 8.

ER/PR positive is defined as an Allred score of >2 and ER/PR negative is defined as an Allred score of less than or equal to 2.

METHODOLOGY:

Tissue was fixed in 10% neutral buffered formalin for no less than 8 and no longer than 24 hours. Immunohistochemistry was performed using the mouse anti-human ER (ER 1D5, 1:100) and PR (PGR 136, 1:100) provided by following the manufacturer s instructions. This assay was not modified. Interpretation of the ER/PR immunohistochemical stain is guided by published results in the medical literature, information provided by the reagent manufacturer and by internal review of staining performance.

SYNOPTIC REPORT - BREAST HER-2 RESULTS

Specimen: Surgical Excision
Block Number: A2

Interpretation: EQUIVOCAL
Intensity: 2+
% Tumor Staining: 10%
Fish Ordered: Yes

METHODOLOGY:

Tissue was fixed in 10% neutral buffered formalin for no less than 8 and no longer than 24 hours. Her2 analysis was performed using the FDA approved HercepTest (TM) test kit ısing rabbit anti-human HER2. This assay was not modified. External kit-slides provided by the manufacturer (cell lines with high, low and negative HER2 protein expression) and in-house known HER2 amplified control tissue were evaluated along with the test tissue. Adequate, well preserved, clear-cut invasive carcinoma was identified for HER2 evaluation. Interpretation of the HER2 immunohistochemical stain is guided by published results in the medical literature, information provided by the reagent manufacturer and by internal review of staining performance. This assay has been validated according to the joint recommendations and guidelines from ASCO and CAP and from the NCCN HER2 testing in Breast Cancer Task Force. The Pathology Department takes full responsibility for this test's performance.

ADDENDUM:

FISH/ISH ANALYSIS REPORT 3

Specimens Involved

Specimens: A: RIGHT BREAST PARTIAL MASTECTOMY

HER2/NEU RESULTS

ANALYTICAL INTERPRETATION OF RESULTS

HER-2 NOT AMPLIFIED

Clinical interpretation of the results

A majority of tumors cells displayed 2 chromosome 17 centimeter signals and 2 HER2 signals, with a HER2/CEP 17 Ratio 1.3, consistent with no amplification of the HER2/neu gene.

Probes identification

LSI Her-2/neu 17q11.2-12, spectrumorange

CEP 17, 17 p11.1-q11.1 alpha satellite DNA, spectrumgreen

Image analysis method - Manual

Results interpreted

Yes

ISCN

nuc ish: (CEP17,HER2)x2[200]

Number of invasive tumor cells counted

200

Number of observers

1

Number of Her2 signals/nucleus

2.3

Number of CEP 17 signals/nucleus

1.8

Her2/CEP 17 ratio

1.3

TEST CHARACTERISTICS: PathVysion HER-2 DNA Probe Kit is FDA approved for selection of patients for whom Herceptin therapy is being considered. These tests were performed in the Pathology Core Facility, Department of Pathology, under the direction of Dr.. The results of these studies should always be interpreted in the context of the clinical, morphological, and immunophenotypic diagnosis. The PathVysion Kit is not intended for use to screen for or diagnose breast cancer. It is intended to be used as an adjunct to other prognostic factors currently used to predict disease-free and overall survival in stage II, node-positive breast cancer patients. In making decisions regarding adjuvant CAF treatment, all other available clinical information should also be taken into consideration, such as tumor size, number of involved lymph nodes, and steroid receptor status. No treatment decision for stage II, node-positive breast cancer patients should be based on HER-2/neu gene amplification status alone.

Specimen information

RPCI surgical pathology/cytology case number

Source of case

RPCI

Block number used A2

Specimen site

Breast

Female breast right

Specimen type

Complete excision (less total mastectomy)

Specimen fixative type

Formalin

Duration of fixation (hrs)

6 - 48 hrs

Comment:

Controls: The FISH study was performed with appropriately stained positive and negative controls.

Criteria	Yes	No
Diagnosis Discrepancy		✓
Primary Tumor Site Discrepancy		✓
HIPAA Discrepancy		✓
Prior Malignancy History		✓
Dual/Synchronous Primary Noted		✓
Case is (circle): QUALIFIED / DISQUALIFIED		
Reviewer Initials [illegible]	Date Reviewed: [illegible]	

LW 11/25/12

UUID:F3FAB505-03FF-4FAA-ABB6-96AD20F47871
TCGA-E2-A574-01A-PR Redacted

SPECIMEN(S): A. PAPILLOMA MEDIAL LEFT BREAST
B. EXCISION LEFT BREAST CANCER
C. SENTINEL LYMPH NODE #1 LEFT AXILLA
D. SENTINEL LYMPH NODE #2 LEFT AXILLA

CLINICAL HISTORY:
1) Cancer 2 cm in axillary tail left breast.
2) Medial left breast mass – Papilloma by core biopsy.

PRE-OPERATIVE DIAGNOSIS:
None Given.

INTRA-OPERATIVE DIAGNOSIS
B: Lumpectomy, left breast, excision; margins check: Mass is located 0.2 cm from the posterior margin.
Reported to Dr. by Dr.

ICD-0-3
carcinoma, infiltrating duct, NOS 8500/3
Site: breast, NOS C50.9
[illegible] 11/25/12

DIAGNOSIS:
A. BREAST, LEFT MEDIAL, EXCISION:
- INTRADUCTAL PAPILLOMA
- APOCRINE METAPLASIA AND COLUMNAR CELL CHANGE
- BIOPSY SITE CHANGES
- SURGICAL MARGINS NEGATIVE FOR TUMOR

B. BREAST, LEFT, EXCISION:
- INVASIVE DUCTAL CARCINOMA, 2.3 CM IN GREATEST DIMENSION, SBR GRADE 3
- SURGICAL MARGINS NEGATIVE FOR TUMOR
- BIOPSY SITE CHANGES

- SEE SYNOPTIC REPORT

C. LYMPH NODES, SENTINEL #1, LEFT AXILLA:
- TWO LYMPH NODES NEGATIVE FOR METASTATIC CARCINOMA (0/2)

D. LYMPH NODES SENTINEL #2, LEFT AXILLA:
- TWO LYMPH NODES NEGATIVE FOR METASTATIC CARCINOMA (0/2)

SYNOPTIC REPORT - BREAST

Specimens Involved
Specimens: B: EXCISION LEFT BREAST CANCER

Specimen Type: Excision
Needle Localization: Yes - For mass
Laterality: Left
Invasive Tumor: Present
Multifocality: No

WHO CLASSIFICATION

Invasive ductal carcinoma, NOS 8500/3
Tumor size: 2.3cm
Additional dimensions: 2.1cm x 2cm
Tumor Site: Not specified
Margins: Negative
Distance from closest margin: 0.3cm
deep
Tubular Score: 2
Nuclear Grade: 3
Mitotic Score: 3
Modified Scarff Bloom Richardson Grade: 3
Necrosis: Present
Vascular/Lymphatic Invasion: None identified
Lobular neoplasia: None
Lymph nodes: Sentinel lymph node
Lymph node status: Negative 0 / 4

DCIS not present

ER/PR/HER2 Results

ER: Negative
PR: Negative
HER2: Negative by FISH
Performed on Case:

Pathological staging (pTN): pT 2 N 0
Pathological staging is based on the AJCC Cancer Staging Manual, 7th Edition

GROSS DESCRIPTION:

A [illegible]PILLOMA MEDIAL LEFT BREAST

Received fresh/in formalin labeled with the patient's identification and designated "papilloma medial left breast" is an oriented (single anterior, double lateral, triple superior), previously inked, 14 g, 4.3 x 3.2 x 2.1 cm needle localized excision. Ink code: anterior-yellow, posterior-black, medial-green, lateral-red, superior-blue, inferior-orange. The specimen is serially sectioned from medial to lateral into 7 slices revealing firm, focally hemorrhagic breast parenchyma. No lesions or nodules are grossly appreciated. Entirely submitted as per the attached diagram:

A1: Perpendicular sections medial margin
A2-A3: Slice 2, bisected
A4-A5: Slice 3, bisected
A6-A7: Slice 4, bisected
A8-A9: Slice 5, bisected
A10-A11: Slice 6, bisected
A12-A13: Perpendicular sections lateral margin

B. EXCISION LEFT BREAST CANCER

Received fresh labeled with the patient's identification and designated "excision left breast cancer" is an oriented (triple - superior, double - lateral, single - anterior), 46 g, (Medial to lateral - 6.5 cm, superior to inferior - 5.4 cm, anterior to posterior - 3.5 cm) lumpectomy specimen. (The specimen is previously inked) Ink code: anterior-yellow, posterior-black, medial-green, lateral-red, superior-blue, inferior-orange. The specimen is serially sectioned from medial to lateral into 9 slices revealing a 2.3 x 2.1 x 2 cm, tan-white to tan-pink, firm to hard mass that is closest to the posterior margin at 0.2 cm, in slices 6 to 9. Tissue is procured.

Representatively submitted in 18 cassettes as per the attached diagram:

B1: slice 1 entire, perpendicular sections of the medial margin
B2: slice 2, representative anterior margin
B3: slice 3, representative posterior margin with the mass
B4-B5: slice 4, sections of the mass showing the closest posterior margin,
B6-B7: slice 5, mass with anterior and posterior margins
B8: slice 5, superior margin
B9-B10: slice 6, mass with anterior and posterior margins
B11: slice 6, inferior margin
B12-B13: slice 6, superior margin
B14-B15: slice 7, representative anterior and key and posterior margins
B16: slice 8, posterior margin
B17-B18: slice 9, lateral margin, perpendicular sections.

C. SENTINEL LYMPH NODE #1 LEFT AXILLA

Received in formalin with the patient's identification and designated "sentinel lymph node #1 left axilla" is a 3.2 x 1.5 x 1.2 cm fibrofatty tissue within which 2 lymph nodes, 2 x 1.3 x 0.7 cm and 2.5 x 1.2 x 1 cm are identified. The lymph nodes are serially sectioned and entirely submitted in 2 cassettes, C1-C2.

D. SENTINEL LYMPH NODE #2 LEFT AXILLA

Received in formalin with the patient's identification and designated "sentinel lymph node #2 left axilla" is a 3.5 x 2.5 x 0.8 cm fibrofatty tissue within which 2 lymph nodes, 1.2 x 1 x 0.5 cm and 0.5 x 0.4 x 0.3 cm are identified. The lymph nodes are serially sectioned and entirely submitted in 2 cassettes, d1-D2.

Criteria	Yes	No
Diagnosis Discrepancy		/
Primary Tumor Site Discrepancy		/
HIPAA Discrepancy		/
Prior Malignancy History		/
Dual/Synchronous Primary Noted		/
Case is (circle): QUALIFIED / DISQUALIFIED		
Reviewer Initials [illegible] Date Reviewed: [illegible]		

11/25/12

TSS ID·

TSS ID: _ _ _ **OC ID:**_ _ _ _ **Date of Procurement**

Gross Description:

Microscopic Description:

Diagnosis Details:

Comments:

Formatted Path Report:

BREAST TISSUE CHECKLIST

ICD-0-3
carcinoma, infiltrating duct, NOS 8500/3
Site: breast, NOS C50.9 4/8/11 [illegible]

Specimen type: Mastectomy

Specimen size: Not specified

Tumor site: Breast

Tumor size: 2 x 1.5 x 1.5 cm

Grossly evident lesion: Yes

Histologic type: Infiltrating ductal carcinoma

Histologic grade: Moderately differentiated

Tumor extent: Not specified

Lymph nodes: 1/12 positive for metastasis (Axillary 1/12)

Extracapsular invasion of the lymph nodes: Not specified

Margins: Not specified

Nottingham Histologic Score

Tubule formation: Not specified

Nuclear pleomorphism: Not specified

Mitotic count (25x): Not specified

Mitotic count (40x): Not specified

Total Nottingham Score: Score cannot be determined

Evidence of neo-adjuvant treatment: Not specified

Additional pathologic findings: Not specified

Comments: None

UUID:DD0ADC20-2059-435E-9EB4-351C87D7CBEB
TCGA-E9-A1R0-01A-PR Redacted

Criteria	Yes	No
Diagnosis Discrepancy		X
Primary Tumor Site Discrepancy		X
HIPAA Discrepancy		X
Prior Malignancy History		X
Dual/Synchronous Primary Noted		X
Case is (circle): QUALIFIED / DISQUALIFIED		
Reviewer Initials [illegible] Date Reviewed: 4/8/11		

ICD-O-3
carcinoma, infiltrating duct, NOS 8500/3
Site: breast, NOS C50.9 [illegible] 4/7/11

Procurement Date Laterality: Right, lower outer quadrant

Path Report: BREAST TISSUE CHECKLIST

Specimen type: Radical mastectomy

Specimen size: Not specified

Tumor site: Breast

Tumor size: 2.2 x 1.8 x 1.8 cm

Grossly evident lesion: Yes

Histologic type: Infiltrating ductal carcinoma

Histologic grade: Moderately differentiated

Tumor extent: Not specified

Lymph nodes: 0/2 positive for metastasis (Regional 0/2)

Extracapsular invasion of the lymph nodes: Not specified

Margins: Not specified

Nottingham Histologic Score

Tubule formation: Not specified

Nuclear pleomorphism: Not specified

Mitotic count (25x): Not specified

Mitotic count (40x): Not specified

Total Nottingham Score: Score cannot be determined

Evidence of neo-adjuvant treatment: Not specified

Additional pathologic findings: Not specified

Comments: None

UUID:BC3061B7-9C99-4270-84EE-B1306FA01AE2
TCGA-E9-A22A-01A-PR Redacted

Criteria	Yes	No
Diagnosis Discrepancy		X
Primary Tumor Site Discrepancy		X
HIPAA Discrepancy		X
Prior Malignancy History		X
Dual/Synchronous Primary Noted		X
Case is (circle) QUALIFIED / DISQUALIFIED		
Reviewer Initials [illegible] Date Reviewed: 4/7/11		

ICD-0-3
Carcinoma, infiltrating duct, NOS 8500/3
Site: breast, NOS C50.9 [illegible] 4/7/11

Procurement Date Laterality:Right, upper outer quadrant

Path Report:BREAST TISSUE CHECKLIST

Specimen type: Radical mastectomy

Specimen size: Not specified

Tumor site: Breast

Tumor size: 2.4 x 2.4 x 2.4 cm

Grossly evident lesion: Yes

Histologic type: Infiltrating ductal carcinoma

Histologic grade: Moderately differentiated

Tumor extent: Not specified

Lymph nodes: 0/10 positive for metastasis (Regional 0/10)

Extracapsular invasion of the lymph nodes: Not specified

Margins: Not specified

Nottingham Histologic Score

Tubule formation: Not specified

Nuclear pleomorphism: Not specified

Mitotic count (25x): Not specified

Mitotic count (40x): Not specified

Total Nottingham Score: Score cannot be determined

Evidence of neo-adjuvant treatment: Not specified

Additional pathologic findings: Not specified

Comments: None

UUID:ED60F792-A71B-4BAE-A536-5FF6D8C3DEAA
TCGA-E9-A22D-01A-PR Redacted

Criteria	Yes	No
Diagnosis Discrepancy		X
Primary Tumor Site Discrepancy		X
HIPAA Discrepancy		X
Prior Malignancy History		X
Dual/Synchronous Primary Noted		X
Case is (circle): QUALIFIED / DISQUALIFIED		
Reviewer Initials [illegible]	Date Reviewed: 4/7/11	

ICD-O-3
carcinoma, infiltrating duct, NOS 8500/3
Site: breast, NOS C50.9 4/7/11 [illegible]

Case ID	Subject ID	[redacted] Formatted Path Report	Laterality	Date of Procurment
		BREAST TISSUE CHECKLIST<br><br>Specimen type: Lumpectomy<br>Specimen size: Not specified<br>Tumor site: Breast<br>Tumor size: 2.1 x 1.8 x 1.4 cm<br>Grossly evident lesion: Yes<br>Histologic type: Infiltrating ductal carcinoma<br>Histologic grade: Moderately differentiated<br>Tumor extent: Not specified<br>Lymph nodes: 1/17 positive for metastasis (Sentinel 1/17)<br>Extracapsular invasion of the lymph nodes: No<br>Margins: Uninvolved<br><br>Nottingham Histologic Score<br>Tubule formation: Moderate 10% to 75% (score=2)<br>Nuclear pleomorphism: Marked variation in size, nucleoli, etc (score=3)<br>Mitotic count (25x): 10 to 20 mitoses per 10 HPF (score=2)<br>Mitotic count (40x): Not specified<br>Total Nottingham Score: Grade II (moderately differentiated): 6-7 points<br><br>Evidence of neo-adjuvant treatment: Not specified	Left, upper outer quadrant	

UUID:13CA22BB-5EA5-46C9-82D3-77867122D1B4
TCGA-E9-A227-01A-PR
Redacted

Criteria	Yes	No
Diagnosis Discrepancy		X
Primary Tumor Site Discrepancy		X
HIPAA Discrepancy		X
Prior Malignancy History		X
Dual/Synchronous Primary Noted		X
Case is (circle): QUALIFIED / DISQUALIFIED		
Reviewer Initials [illegible]	Date Reviewed: 4/7/11	

ICD-0-3
Carcinoma, infiltrating duct, NOS 8500/3
Site: breast, NOS C50.9 [illegible] 4/7/11

Procurement Date: Laterality:Right, lower inner quadrant

Path Report:BREAST TISSUE CHECKLIST

Specimen type: Mastectomy

Specimen size: Not specified

Tumor site: Breast

Tumor size: 2 x 1.5 x 2 cm

Grossly evident lesion: Yes

Histologic type: Infiltrating ductal carcinoma

Histologic grade: Moderately differentiated

Tumor extent: Not specified

Lymph nodes: Not specified

Extracapsular invasion of the lymph nodes: Not specified

Margins: Not specified

Nottingham Histologic Score

Tubule formation: Not specified

Nuclear pleomorphism: Not specified

Mitotic count (25x): Not specified

Mitotic count (40x): Not specified

Total Nottingham Score: Score cannot be determined

Evidence of neo-adjuvant treatment: Not specified

Additional pathologic findings: Not specified

Comments: None

UUID:15FA41AE-3A0F-4112-828B-EE8687A6F158
TCGA-E9-A229-01A-PR Redacted

Criteria	Yes	No
Diagnosis Discrepancy		X
Primary Tumor Site Discrepancy		X
HIPAA Discrepancy		X
Prior Malignancy History		X
Dual/Synchronous Primary Noted		X
Case is (circle): QUALIFIED / DISQUALIFIED		
Reviewer Initials [illegible] Date Reviewed: 4/7/11		

Surgical Pathology Report
* Revised *

Name: [redacted]
DOB: [redacted]
Gender: F
MRN: [redacted]
Location:
Physician:

Case #: [redacted]
Collected:
Received:
Reported:
Copy To:

Pathologic Interpretation:

ICD-0-3
Carcinoma, infiltrating ductal, NOS 8500/3
Site: breast, NOS C50.9 3/12/11

AMENDMENT,

A. SUPERIOR MEDIAL MARGIN, RULE OUT TUMOR:
- No malignancy seen.

B. SENTINEL NODE #1:
- **Microscopic foci of carcinoma are highlighted by Keratin (1/1).**

C. SENTINEL NODE #2:
- No malignancy seen in one lymph node (0/1).
- **Immunohistochemistry for keratin is negative for carcinoma.**

D. SENTINEL NODE #3:
- Metastatic carcinoma to one lymph node (1/1) with extracapsular extension.

E. RIGHT BREAST MASS:
- Invasive ductal carcinoma Nottingham grade 2 (3+2+1); 4.2 cm in greatest dimension.
- Specimen margins are negative for tumor closest margins are anterior and superior, 0.2 cm (See Tumor Summary).
- Lymphovascular invasion is identified.

F. ADDITIONAL AXILLARY FAT:
- No malignancy seen in one lymph node (0/1).

G. ADDITIONAL SUPERIOR MARGIN:
- Microscopic focus of invasive ductal carcinoma, 0.1 cm, adjacent to inked resection margin.
- **The focus of invasive carcinoma is present at the inked margin adjacent to previous resection.**
- **The "new" inked margin is free of tumor.**
- Lymphatic tumor emboli are identified.

H. RIGHT AXILLARY CONTENTS:
- No malignancy seen in nineteen lymph nodes (0/19).

I. ADDITIONAL AXILLARY CONTENTS LEVEL 2-3 LYMPH NODES:
- No malignancy seen in two lymph nodes (0/2).

UUID:6FEAD496-58BA-42DC-A3F2-2F380EEB1588
TCGA-EW-A1P6-01A-PR Redacted

Tumor Summary:

Specimen:
- Partial breast

Procedure:
- Excision without wire-guided localization.

Lymph Node Sampling:
- Sentinel lymph nodes
- Axillary dissection

Specimen Integrity:
- Multiple designated specimens

Specimen Size:
- Greatest dimension: 8.5 cm
* Additional dimension: 6 x 5 cm.

Laterality:
Right

Tumor Size:

- Greatest dimension of largest focus of invasion over 0.1 cm: 4.2 cm.
  - Additional dimensions: 3.8 x 2.8 cm.

Tumor Focality:
- Single focus of invasive carcinoma.

Macroscopic and Microscopic Extent of tumor:
- Skin: Skin is not present.
- Skeletal muscle: No skeletal muscle present

Lobular Carcinoma IN Situ (LCIS):
- Not identified

Histologic Type of Invasive Carcinoma:
- Invasive ductal carcinoma

Histologic Grade:
- Glandular (Acinar)/Tubular Differentiation:
  - Score 3: <10% of tumor area forming glandular/tubular structures
- Nuclear Pleomorphism:
  - Score 2: Cells larger than normal with open vesicular nuclei, visible nucleoli, and moderate variability in both size and Shape
- Mitotic Count:
  - Score 1
- Overall Grade:
  - Grade 2: scores of 6 or 7

Margins:
- Margins negative for invasive carcinoma.

Lymph-Vascular Invasion:
- Present

Lymph Nodes:
  - Number of sentinel lymph nodes examined: 3
  - Total number of lymph nodes examined (sentinel and Nonsentinel): 25
  - Number of lymph nodes with macrometastases (>0.2 cm): 2

Extranodal Extension:
- Present

Method of Evaluation of Sentinel Lymph Nodes:
- Hematoxylin and eosin (H&E), one level
- Immunohistochemistry

Pathologic Staging:
- Primary Tumor: pT2: Tumor >20 mm but ≤50 mm in greatest dimension
- Regional Lymph Nodes: pN1a: Metastases in 1 to 3 axillary lymph nodes, at least 1 metastasis greater than 2.0 mm
- Distant Metastasis: Not applicable

Ancillary Studies:

Estrogen Receptor:
  - Performed on another specimen: Results: Immunoreactive tumor cells present (≥1%)

Progesterone Receptor:
  - Performed on another specimen: Results: Immunoreactive tumor cells present (≥1%)

HER2/neu
  - Performed on another specimen: Results: Negative (Score 0)

Clinical History:
- Palpable mass

Pathologic Staging (pTNM): pT2, N1a, M-not applicable

PREVIOUSLY ISSUED DIAGNOSIS:

A. SUPERIOR MEDIAL MARGIN, RULE OUT TUMOR:
- No malignancy seen.

B. SENTINEL NODE #1:
- No malignancy seen in one lymph node (0/1).
- Immunohistochemistry for keratin to follow.

C. SENTINEL NODE #2:

- No malignancy seen in one lymph node (0/1).
- Immunohistochemistry for keratin to follow.

D. **SENTINEL NODE #3:**
- Metastatic carcinoma to one lymph node (1/1) with extracapsular extension.

E. **RIGHT BREAST MASS:**
- Invasive ductal carcinoma Nottingham grade 2 (3+2+1); 4.2 cm in greatest dimension.
- Specimen margins are negative for tumor closest margins are anterior and superior, 0.2 cm (See Tumor Summary).
- Lymphovascular invasion is identified.

F. **ADDITIONAL AXILLARY FAT:**
- No malignancy seen in one lymph node (0/1).

G. **ADDITIONAL SUPERIOR MARGIN:**
- Microscopic focus of invasive ductal carcinoma, 0.1 cm, adjacent to inked resection margin.
- Lymphatic tumor emboli are identified.

H. **RIGHT AXILLARY CONTENTS:**
- No malignancy seen in nineteen lymph nodes (0/19).

I. **ADDITIONAL AXILLARY CONTENTS LEVEL 2-3 LYMPH NODES:**
- No malignancy seen in two lymph nodes (0/2).

Tumor Summary:

Specimen:
- Partial breast

Procedure:
- Excision without wire-guided localization.

Lymph Node Sampling:
- Sentinel lymph nodes
- Axillary dissection

Specimen Integrity:
- Multiple designated specimens

Specimen Size:
- Greatest dimension: 8.5 cm
* Additional dimension: 6 x 5 cm.

Laterality:
- Right

Tumor Size:
- Greatest dimension of largest focus of invasion over 0.1 cm: 4.2 cm.
  - Additional dimensions: 3.8 x 2.8 cm.

Tumor Focality:
- Single focus of invasive carcinoma.

Macroscopic and Microscopic Extent of tumor:
- Skin: Skin is not present.
- Skeletal muscle: No skeletal muscle present

Lobular Carcinoma IN Situ (LCIS):
- Not identified

Histologic Type of Invasive Carcinoma:
- Invasive ductal carcinoma

Histologic Grade:
- Glandular (Acinar)/Tubular Differentiation:
  - Score 3: <10% of tumor area forming glandular/tubular structures
- Nuclear Pleomorphism:
  - Score 2: Cells larger than normal with open vesicular nuclei, visible nucleoli, and moderate variability in both size and Shape
- Mitotic Count:
  - Score 1
- Overall Grade:
  - Grade 2: scores of 6 or 7

Margins:

- Margins positive for invasive carcinoma.
    - Specify margin and Extent of involvement: Superior margin, focal

Lymph-Vascular Invasion:
- Present

Lymph Nodes:
- Number of sentinel lymph nodes examined: 3
- Total number of lymph nodes examined (sentinel and Nonsentinel): 25
- Number of lymph nodes with macrometastases (>0.2 cm): 1

Extranodal Extension:
- Present

Method of Evaluation of Sentinel Lymph Nodes:
- Hematoxylin and eosin (H&E), one level
- Immunohistochemistry

Pathologic Staging:
- Primary Tumor: pT2: Tumor >20 mm but ≤50 mm in greatest dimension
- Regional Lymph Nodes: pN1a: Metastases in 1 to 3 axillary lymph nodes, at least 1 metastasis greater than 2.0 mm
- Distant Metastasis: Not applicable

Ancillary Studies:

Estrogen Receptor:
- Performe[illegible] on another specimen — Results: Immunoreactive tumor cells present (≥1%)

Progesterone [illegible]tor:
- [illegible]ed on another specimen: — Results: Immunoreactive tumor cells present (≥1%)

HER[illegible]
[illegible]med on another specimen: — Results: Negative (Score 0)

Clinical History:
- Palpable mass

Pathologic Staging (pTNM): pT2, N1a, M-not applicable

NOTE: Some immunohistochem[illegible] [illegible]re analyte specific reagents (ASRs) validated by our laboratory. These ASRs are clinically useful indicators that do not require FDA approval. These clones are used: ID5-ER, PgR 636-PR, A485-H[illegible] [illegible] All immunohistochemical stains are used with formalin or molecular fixed, paraffin embedded tissue. Detection is by Envision Method. The results are read by a pathologist as positive or negative.

As the attending pathologist, I attest that I: (i) Examined the relevant preparation(s) for the specimen(s); and (ii) Rendered the diagnosis(es).

MD

Electronically Signed Out By

Amendments

Amended by

Reason: Diagnosis editing/clarification
New information provided to Dr. : by physician.

Previous Signout Date:

Intraoperative Consultation

A. Superior medial margin, rule out tumor, FS: No tumor seen.

B. Sentinel node #1, FS: Negative for carcinoma (touch prep and frozen)

C. Sentinel node #2, FS: Negative for carcinoma (touch prep and frozen)

D. Sentinel node #3, FS: Atypical cells on touch prep-carcinoma cannot be excluded.
FS: Metastatic carcinoma in lymph node.

, MD

MD

Clinical History:
female with right breast mass. Biopsy shows invasive ductal carcinoma

Operation Performed
Lumpectomy right breast with sentinel node

Pre Operative Diagnosis:
Breast cancer

Specimen(s) Received:
A: Superior medial margin, rule out tumor, FS
B: Sentinel node #1, FS
C: Sentinel node #2, FS
D: Sentinel node #3, FS
E: Right breast mass
F: Additional axillary fat
G: Additional superior margin
H: Right axillary contents
I: Additional axillary contents level 2-3 lymph nodes

Gross Description:

A. Received fresh are two yellow-tan tissue fragments, measuring up to 1.0 cm in length. In toto in one cassette for frozen.

B. Received fresh is a light brown lymph node 1.5 x 1.0 cm. In toto in one cassette for frozen.

C. Received fresh is a lymph node, measuring 2.0 x 1.5 cm. In toto in one cassette for frozen.

D. Received fresh is a lymph node, measuring 1.2 x 1.0 cm. In toto in one cassette for frozen.

E. Received in formalin is a yellow-tan fibroadipose tissue fragment, weighing 89 grams, measuring 8.5 x 6.0 x 5.0 cm. The specimen is oriented with a short superior, long lateral and double stitch deep margin. Inked as follows: Superior in blue, inferior in green, deep black, anterior yellow, lateral orange and medial red. Cross section through the specimen shows yellow-tan cut surface. There is an ovoid, markedly firm, tumoral mass, measuring 4.2 x 3.8 x 2.8 cm. The mass is within 2 mm from superior and anterior inferior margin. No other firm masses were grossly identified. Sections submitted as follows:
1&2 Composite resection of the largest axis of the mass from antero-inferior to superior margin
3-5 Additional section of the mass in relation with the antero-inferior margin
6&7 Section of the mass in relation with the deep resection margin
8 Medial margin
9 Lateral margin

F. Received in formalin are multiple yellow-tan adipose tissue fragments, measuring 6.0 x 4.0 x 1.0 cm in aggregate. No lymph nodes were grossly identified. Sections from the fat tissue in three cassettes.

G. Received in formalin is a yellow-tan adipose tissue fragment (additional superior margin), measuring 6.0 x 3.0 x 1.0 cm. Multiple sutures orient the specimen as a true margin. The true margin in green. The opposite margin in black. Cross section through the specimen shows a yellow-tan cut surface. No masses were grossly present. Submitted in toto in eight cassettes.

H. Received in formalin are multiple yellow-tan fibroadipose tissue fragments, measuring 10.0 x 7.0 x 3.0 cm in aggregates. Twenty lymph nodes are present, measuring up to 0.8 x 0.7 cm in greatest dimension. Sections as follows:
1 Two lymph nodes in toto
2-8 Three lymph nodes in toto per cassette

I. Received are multiple yellow-tan fibroadipose tissue fragments, measuring 5.0 x 3.0 x 1.0 cm. Four lymph nodes are identified, measuring up to 0.6 x 0.4 cm. Sections as follows:
1&2 Two lymph nodes in toto per cassette

Criteria	Yes	No
Diagnosis Discrepancy		/
Primary Tumor Site Discrepancy		/
HIPAA Discrepancy		/
Prior Malignancy History		/
Dual/Synchronous Primary Noted		/
Case is (circle): QUALIFIED / DISQUALIFIED		
Reviewer Initials [illegible] Date Reviewed: 3/12/11		

UUID:6EEF1F11-603E-4196-91AB-724BBEE20CE7
TCGA-EW-A6SB-01A-PR Redacted

Surgical Pathology Report

**Name:**	xxxx	**Case #:**	x
**DOB:**		**Collected:**	
**Gender:**	F	**Received:**	x
**MRN:**	x	**Reported:**	x
**Location:**	x		
**Physician:**	x		

ICD-O-3
Carcinoma, infiltrating duct NOS 8500/3
Site (L) Breast NOS C50.9
7/24/13

Pathologic Interpretation:

A. LEFT AXILLARY # 1

Negative for carcinoma, four lymph nodes examined (0/4).

Keratin immunostain is pending.

B. LEFT AXILLARY, , PALPABLE:

Negative for carcinoma, four lymph nodes examined (0/4).

Keratin immunostain is pending.

C. LEFT SEGMENTAL MASTECTOMY:

INVASIVE DUCTAL CARCINOMA, poorly differentiated, Nottingham grade 3 (3+3+3=9), 3.1 cm in greatest dimension.

Immunohistochemical studies performed on previous biopsy showed the following results:

Estrogen Receptor:	NEGATIVE (< 1%)
Progesterone Receptor:	NEGATIVE (< 1%)
Her2/neu	NEGATIVE (0)
Androgen Receptor:	NEGATIVE
EGFR:	POSITIVE
GCDFP:	NEGATIVE
HLA-DR:	Immunohistochemistry is pending

Skin is uninvolved by tumor.

Resection margins are uninvolved by invasive carcinoma; the tumor is at less than 1 mm from the deep resection margin.

Negative for lymphatic space invasion.

SURGICAL PATHOLOGY CANCER CASE SUMMARY

Procedure
Excision without wire-guided localization
Lymph Node
Sentinel lymph node(s)
Specimen Laterality
Left
Histologic Type of Invasive Carcinoma
Invasive ductal carcinoma (no special type or not otherwise specified)
Tumor Size: Size of Largest Invasive Carcinoma
Greatest dimension of largest focus of invasion >1 mm: 31 mm
Histologic Grade: Nottingham Histologic Score
Glandular (Acinar)/Tubular Differentiation
Score 3: <10% of tumor area forming glandular/tubular structures
Nuclear Pleomorphism
Score 3: Vesicular nuclei, often with prominent nucleoli, exhibiting marked variation in size and shape, occasionally with very large and bizarre forms
Mitotic Rate
Score 3 (≥8 mitoses per mm^2) (see Table 1)
Overall Grade
Grade 3: scores of 8 or 9
Tumor Focality
Single focus of invasive carcinoma.
Ductal Carcinoma In Situ (DCIS)
No DCIS is present
Lobular Carcinoma In Situ (LCIS)
Not identified
Margins
Invasive Carcinoma
Margins uninvolved by invasive carcinoma
Distance from closest margin: 1 mm
Specify margin: Deep
Lymph Nodes
Number of sentinel lymph nodes examined: 8
Total number of lymph nodes examined (sentinel and nonsentinel): 0
Number of lymph nodes without tumor cells identified: 8
Method of Evaluation of Sentinel Lymph Nodes
Hematoxylin and eosin (H&E), 1 level
Immunohistochemistry
Treatment Effect: Response to Presurgical (Neoadjuvant) Therapy
In the Breast
No known presurgical therapy
In the Lymph Nodes
No known presurgical therapy
Lymph-Vascular Invasion
Not identified
Dermal Lymph-Vascular Invasion
Not identified

Pathologic Staging (based on information available to the pathologist) (pTNM)

Primary Tumor (Invasive Carcinoma) (pT)
pT2: Tumor >20 mm but ≤50 mm in greatest dimension

Regional Lymph Nodes (pN)
pN0: No regional lymph node metastasis identified histologically

Distant Metastasis (pM)
Not applicable

Ancillary Studies

Performed on another specimen
Specify specimen (accession number): XXX

Estrogen Receptor (ER)
Results and interpretation:
Negative (<1% of tumor cells with nuclear positivity)

Progesterone Receptor (PgR)
Results and interpretation:
Negative (<1% of tumor cells with nuclear positivity)

HER2 *Immunoperoxidase Studies*
Results:
Negative (Score 0)

AJCC Classification (7th edition): pT2, pN0, pMn/a

Procedures/Addenda:

Addendum

Date Ordered:
Date Complete:
Date Reported:

Status:

Addendum Diagnosis

A. LEFT AXILLARY #1

Keratin immunohistochemistry is negative.

B. LEFT AXILLARY, PALPABLE:

Keratin immunohistochemistry is negative.

Final AJCC Classification (7th edition): pT2 (sn)N0(i -) Mn/a

Addendum Diagnosis

ADDENDUM C:

Immunohistochemistry for HLA-DR is positive in tumoral cells.

This neoplasm is consistent with an invasive ductal carcinoma, medullary type.

Intraoperative Consultation:

A. Left axillary #1, , FS: No malignancy seen

B. Left axillary , palpable, FS1-3: No malignancy seen.

X.

Clinical History:

Patient with palpable breast cancer, please evaluate margins and lymph nodes.

Operation Performed:
Left segmental mastectomy with sentinel lymph node biopsy, possible axillary node dissection

Pre Operative Diagnosis:
Carcinoma left breast

Specimen(s) Received/Processing Information:
Fee Codes:

**A: LEFT AXILLARY # 1** **FS** *Frozen section x 1, FS Perm x 1, Touch Prep Histology x 1, FSDeep 1 x 1, Cytokeratin Cocktail (KER) x 1, H&E, Initial x 1*	A:
**B: LEFT AXILLARY, , PALPABLE FS** *Frozen section x 1, FS Perm x 1, Touch Prep Histology x 1, Cytokeratin Cocktail (KER) x 1, Frozen section x 1, FS Perm x 1, Cytokeratin Cocktail (KER) x 1, Frozen section x 1, FS Perm x 1, Cytokeratin Cocktail (KER) x 1, H&E, Initial x 1, Cytokeratin Cock*	B:
**C: LEFT SEGMENTAL MASTECTOMY (1 SUTURE -SUPEIOR, 2 SUTURES - MEDIAL)** *H&E, Initial x 1, H&E, Initial x 1, H&E, Initial x 1, H&E, Initial x 1, H&E, Initial x 1, H&E, Initial x 1, H&E, Initial x 1, H&E, Initial x 1, H&E, Initial x 1, HLA-DR (IP) x 1, H&E, Initial x 1, H&E, Initial x 1, H&E, Initial x 1, H&E, Initial x 1, H&E,*	C:

Gross Description:

A.Received fresh labeled "Left axillary #1, . FS" is a fragment of adipose tissue that measures 5 x 4 x 2 cm. There are four palpable lymph nodes present. The lymph nodes are bisected and submitted for frozen section (cassette #1FS). Representative sections of the remaining adipose tissue are submitted in cassette #2.

B.Received fresh labeled "Left axillary , palpable, FS" is a fragment of adipose tissue that measures 4 x 4 x 2 cm. There are four palpable lymph nodes present. The lymph nodes are bisected and submitted for frozen section in cassettes #1-3FS. The rest of the specimen is submitted in cassettes #4-8.

C.Received in formalin labeled "Left segmental mastectomy (1 suture –superior; 2 sutures –medial)" is a lumpectomy specimen weighing 120 grams and measuring 9 x8 x 3.5 cm, with an unremarkable dark skin ellipse measuring 3.3 x 1.3 cm. The specimen is inked as follows: superior-blue, inferior-green, anterior-yellow, deep-black, medial-red, lateral-orange. Upon sectioning, there is a 3.1 x 3.0 x 3.0 cm well circumscribed, nodular tumor, white-tan, with an elastic consistency and central cavity. A metallic clip is identified in the central of the tumor. The tumor is grossly at less than 0.1 cm from the deep margin, 0.4 cm from the superior margin, 1.0 cm from the lateral margin, 2.0 cm from the medial margin, 2.5 cm from the anterior and skin margin, 4.0 cm from the inferior margin. The rest of the specimen has a cut surface, yellow-tan and homogeneous. Cassettes are submitted as follows:

Cassette #1	Tumor in relation with deep and superior margins
Cassette #2	Medial margin
Cassette #3	Lateral margin
Cassette #4	Anterior margin (and skin)
Cassette #5	Inferior margin
Cassette #6	Area of tumor with clip
Cassettes #7&8	Composite section of the tumor
Cassettes #9-12	Additional sections of the tumor
Cassettes #13-15	Representative sections of the rest of specimen

Criteria lw 7/9/13	Yes	No
Diagnosis Discrepancy		✓
Primary Tumor Site Discrepancy		✓
HIPAA Discrepancy		✓
Prior Malignancy History		✓
Dual/Synchronous Primary Noted		✓
Case is (circle): QUALIFIED / DISQUALIFIED		
Reviewer Initials [illegible] Date Reviewed: 7/9/13		

Criteria	Yes	No
Diagnosis Discrepancy		✓
Primary Tumor Site Discrepancy		✓
HIPAA Discrepancy		✓
Prior Malignancy History		✓
Dual/Synchronous Primary Noted		✓
Case is (circle): QUALIFIED / DISQUALIFIED		
Reviewer Initials [illegible] Date Reviewed: [illegible]		

Final Pathologic Diagnosis:
A. Lymph node, level III, excision:
No evidence of malignancy (3 lymph nodes).

B. Breast, right, radical mastectomy:
Tumor (histologic type): Invasive ductal carcinoma.
Final size of invasive tumor: 2.4 cm
Scarff-Bloom-Richardson score:
Tubular score: 3
Nuclear score: 3
Mitotic score: 2
Total score: 8
In-situ component: Not identified.
Type: NA
Percent: NA
Architectural pattern: NA
Nuclear grade: NA
Comedo necrosis: NA
Angiolymphatic invasion: Present.
Skin and nipple: Tumor present in the dermis of the skin and nipple.
Dermal lymphatics: Angiolymphatic invasion is present.
Microcalcifications: Not identified.
Margins (distance \T\ size of involved area): Tumor is >1 cm from all margins.
Other findings:
Despite gross impression, microscopically the tumor does not invade the muscle or bone.

ICD-0-3
Carcinoma, infiltrating duct, NOS 8500/3
Site breast, NOS C50.9
[illegible] 6/1/11

Lymph nodes (number positive/total number):
Ten lymph nodes negative for metastatic tumor (includes lymph nodes from part A).
Size of largest metastasis: NA
Number with extracapsular extension: NA

UUID:4AABCBD1-DF12-45E1-9307-21D6CB0894A9
TCGA-GI-A2C8-01A-PR Redacted

ER, PR: Positive, see case
HER2/neu: Negative, see case

pT4B, N0, MX

The examination of this case material and the preparation of this report were performed by the staff pathologist.

***Electronically Signed ***
, M.D.
, M.D.

Gross Description:
Received are two fixative filled containers labeled with the patient's name and medical record number.

Part A is additionally labeled "level 3 lymph nodes." The specimen consists of three fragments of yellow-tan fibroadipose tissue ranging from 1.1 to 2.2 cm in

greatest dimension. Six lymph node candidates are identified, ranging from 0.3 to 1.4 cm.

A1 four lymph nodes entirely submitted;
A2 two lymph nodes entirely submitted.

Part B is additionally labeled "right radical mastectomy."
Specimen type: Radical mastectomy.
Specimen dimensions: 21.2 x 20.5 x 4.1 cm with a 6.5 x 5.4 x 1.7 cm portion of rib and chest wall.
Skin: A 20.6 x 10.2 cm ellipse of skin including the nipple is present. No scar is identified, however, there is significant puckering of the skin inferior to the nipple.
Location of lesion: The location is central.
Estimated size of lesion: The lesion is approximately 4.8 x 2.9 x 2.9 cm. (for final size, see Microscopic Description)
Appearance of lesion: The tumor is firm and white.
Distance from closest margin: The tumor appears to abut the inked deep margin.
Other findings: The tumor appears to involve the included pectoralis muscle.
Blocks submitted:

B1 nipple;
B2 tumor to inked deep margin and chest wall muscle;
B3 tumor to skin and tumor greatest cross section;
B4-5 additional representative tumor;
B6 upper inner quadrant;
B7 lower inner quadrant;
B8 outer lower quadrant;
B9 outer upper quadrant;
B10 one axillary tail lymph node bisected, entirely submitted;
B11 three axillary tail lymph nodes entirely submitted;
B12 three axillary tail lymph nodes entirely submitted;
B13 chest wall bone closest to tumor after decalcification.

, M.D.

Microscopic Description:
The final diagnosis of each specimen incorporates the microscopic examination findings.

Final Pathologic Diagnosis:

A. Lymph node, level III, excision:
No evidence of malignancy (3 lymph nodes).

B. Breast, right, radical mastectomy:
Tumor (histologic type): Invasive ductal carcinoma.
Final size of invasive tumor: 2.4 cm
Scarff-Bloom-Richardson score:
Tubular score: 3
Nuclear score: 3
Mitotic score: 2
Total score: 8
In-situ component: Not identified.
Type: NA
Percent: NA
Architectural pattern: NA
Nuclear grade: NA
Comedo necrosis: NA
Angiolymphatic invasion: Present.
Skin and nipple: Tumor present in the dermis of the skin and nipple.
Dermal lymphatics: Angiolymphatic invasion is present.
Microcalcifications: Not identified.
Margins (distance \T\ size of involved area): Tumor is >1 cm from all margins.
Other findings:
Despite gross impression, microscopically the tumor does not invade the muscle or bone.

Lymph nodes (number positive/total number):
Ten lymph nodes negative for metastatic tumor (includes lymph nodes from part A).
Size of largest metastasis: NA
Number with extracapsular extension: NA

ER, PR: Positive, see case
HER2/neu: Negative, see case

pT4B, N0, MX

The examination of this case material and the preparation of this report were performed by the staff pathologist.

***Electronically Signed ***
, M.D.
, M.D.

Gross Description:
Received are two fixative filled containers labeled with the patient's name and medical record number.

Part A is additionally labeled "level 3 lymph nodes." The specimen consists of three fragments of yellow-tan fibroadipose tissue ranging from 1.1 to 2.2 cm in

greatest dimension. Six lymph node candidates are identified, ranging from 0.3 to 1.4 cm.
A1 four lymph nodes entirely submitted;
A2 two lymph nodes entirely submitted.

Part B is additionally labeled "right radical mastectomy."
Specimen type: Radical mastectomy.
Specimen dimensions: 21.2 x 20.5 x 4.1 cm with a 6.5 x 5.4 x 1.7 cm portion of rib and chest wall.
Skin: A 20.6 x 10.2 cm ellipse of skin including the nipple is present. No scar is identified, however, there is significant puckering of the skin inferior to the nipple.
Location of lesion: The location is central.
Estimated size of lesion: The lesion is approximately 4.8 x 2.9 x 2.9 cm. (for final size, see Microscopic Description)
Appearance of lesion: The tumor is firm and white.
Distance from closest margin: The tumor appears to abut the inked deep margin.
Other findings: The tumor appears to involve the included pectoralis muscle.
Blocks submitted:
B1 nipple;
B2 tumor to inked deep margin and chest wall muscle;
B3 tumor to skin and tumor greatest cross section;
B4-5 additional representative tumor;
B6 upper inner quadrant;
B7 lower inner quadrant;
B8 outer lower quadrant;
B9 outer upper quadrant;
B10 one axillary tail lymph node bisected, entirely submitted;
B11 three axillary tail lymph nodes entirely submitted;
B12 three axillary tail lymph nodes entirely submitted;
B13 chest wall bone closest to tumor after decalcification.

, M.D.

Microscopic Description:
The final diagnosis of each specimen incorporates the microscopic examination findings.

Taken:

ICD-O-3
Carcinoma, infiltrating duct, NOS 8500/3
Site: breast, NOS C50.9
[illegible] 5/23/11

Criteria [illegible] 5/23/11	Yes	No
Diagnosis Discrepancy		✓
Primary Tumor Site Discrepancy		✓
HIPAA Discrepancy		✓
Prior Malignancy History		✓
Dual/Synchronous Primary Noted		✓
Case is (circle): QUALIFIED / DISQUALIFIED		
Reviewer Initials [illegible]	Date Reviewed: [illegible]	

(A) SENTINEL LYMPH NODE #1, LEFT AXILLA, BIOPSY:
One lymph node, no tumor present (0/1).
Cytokeratin stain shows no evidence of metastatic carcinoma.

(B) SENTINEL LYMPH NODE #2, LEFT AXILLA, BIOPSY:
One lymph node, no tumor present (0/1).
Cytokeratin stain shows no evidence of metastatic carcinoma.

(C) SENTINEL LYMPH NODE #3, LEFT AXILLA, BIOPSY:
One lymph node, no tumor present (0/1).
Cytokeratin stain shows no evidence of metastatic carcinoma.

(D) LEFT BREAST, SEGMENTAL MASTECTOMY:
INVASIVE DUCTAL CARCINOMA OF BREAST, MODIFIED BLACK'S NUCLEAR GRADE 3 (POORLY DIFFERENTIATED) (SEE COMMENT).

INVASIVE CARCINOMA MEASURES 3.0 CM IN GREATEST DIMENSION GROSSLY.

No definitive lymphatic/vascular invasion identified.

FOCAL INTRADUCTAL CARCINOMA (DCIS), MODIFIED BLACK'S NUCLEAR GRADE 3 (HIGH GRADE), SOLID AND CRIBRIFORM TYPES WITH NECROSIS, COMPRISING APPROXIMATELY 10% OF TUMOR.

INVASIVE AND IN SITU CARCINOMA ARE 0.5 CM TO THE CLOSEST ANTERIOR MARGIN.

Remaining surgical margins are widely free of tumor.

(E) SENTINEL LYMPH NODE #4, LEFT AXILLA, BIOPSY:
Benign fibroadipose tissue, no lymph node or tumor present.

Entire report and diagnosis completed by

COMMENT

Immunohistochemical staining is performed on a representative formalin-fixed, paraffin-embedded section of INVASIVE CARCINOMA, left breast, block D6.

MARKER	RESULTS	% POSITIVE	SCORE
Estrogen Receptor	Negative	0%	N/A
Progesterone Receptor	Negative	0%	N/A
HER-2/neu overexpression	Negative		1+

UUID:5A315635-886B-459E-AF5D-079F66E859F0
TCGA-GM-A2DB-01A-PR Redacted

Due to the above HER-2/neu immunohistochemical staining result, gene copy level (HER-2/neu: CEP17 signal ratio) will be evaluated by FISH and a separate report will be issued.

FOOTNOTE

Estrogen receptor was assessed by immunohistochemistry using antibody 6F11 (

Progesterone receptor was assessed by immunohistochemistry using antibody PgR1294(

HER-2/neu was assessed by immunohistochemistry using antibody AB8 (

GROSS DESCRIPTION

(A) SENTINEL LYMPH NODE #1, LEFT AXILLA, IN VIVO 69, EX VIVO 55, BLUE - Two fragments of tissue including one lymph node measures 2.0 x 1.5 x 0.8 cm. Serially sectioned. Touch preparation performed.

SECTION CODE: A1, A2, one lymph node; A3, A4, second fragment of tissue.

*TP/DX: NO TUMOR PRESENT.

(B) SENTINEL LYMPH NODE #2, LEFT AXILLA, BLUE, IN VIVO 21, EX VIVO 39 - A single lymph node (2.5 x 1.8 x 1.0 cm).
Serially sectioned. Touch preparation performed. Entirely submitted.

SECTION CODE: B1-B3, one lymph node serially sectioned.

*TP/DX: NO TUMOR PRESENT.

(C) SENTINEL LYMPH NODE #3, LEFT AXILLA, BLUE, IN VIVO 15, EX VIVO 5 - A single lymph node (1.0 x 0.8 x 0.5 cm). Serially sectioned. Touch preparation performed.

SECTION CODE: C, one lymph node serially sectioned.

*TP/DX: NO TUMOR PRESENT.

(D) LEFT BREAST, SEGMENTAL MASTECTOMY, SHORT STITCH SUPERIOR, LONG STITCH LATERAL - The specimen consists of an oriented segmental mastectomy measuring 9.0 x 6.0 x 3.5 cm. Serially sectioned to reveal a 3.0 cm mass, which is 0.5 cm to the closest anterior margin. Remaining surgical margins are widely free of tumor. The specimen is x-rayed, and a surgical clip is identified within the tumor (slice #4). Suspicious areas are circled by the radiologist for sampling.

INK CODE: Superior - blue, anterior - yellow, inferior - green, medial and lateral - red, deep - black.

SECTION CODE: D1, perpendicular sections of medial margin; D2-D5, circled by radiologist in slice #3 with anterior margin; D6, D7, tumor/clipped area in slice #4 (mirror images); D8, anterior margin, slice #4; D9, inferior margin, slice #4; D10, deep margin, slice #4; D11, superior margin, slice #4; D12, D13, remaining tumor area in slice #4; D14-D16, circled by the radiologist in slice #5; D17, inferior and deep margin, slice #5; D18, remaining tumor area in slice #5; D19, D20, circled by radiologist in slice #6; D21, D22, circled by radiologist in slice #7; D23, perpendicular sections of lateral margin.

(E) SENTINEL LYMPH NODE #4, BLUE, LEFT AXILLA IN VIVO 20, EX VIVO 20 - An irregular fragment of fibroadipose tissue (3.0 x 2.0 x 0.6 cm). Serially sectioned and no lymph nodes grossly identified. The specimen is entirely submitted in E1-E3.

CLINICAL HISTORY

Newly diagnosed left breast cancer.

SNOMED CODES

T-04050, M-85003, M-85002, T-C4714, M-00110

"Some tests reported here may have been developed and performance characteristics determined b) These tests have not been specifically cleared or approved by the U.S. Food and Drug Administration."

Released by:

Start of ADDENDUM

ADDENDUM

Addendum completed by I

SPECIMEN SOURCE

Left breast

SUMMARY

Tissue section of the invasive carcinoma / D6 was evaluated for HER-2/neu gene amplification by interphase fluorescence in situ hybridization technique using the PathVysion HER-2/neu DNA Probe Kit (LSI HER-2/neu)/CEP17

Slide adequacy is satisfactory. Sixty tumor nuclei were counted and showed an average of 1.67 copies of LSI HER-2/neu gene per nucleus and an average of 1.50 copies of CEP17 per nucleus.

Negative and positive controls (established by) with this batch are appropriate.

Two representative images have been archived.

INTERPRETATION

The tumor cells demonstrated no amplification of the HER-2/neu gene copy levels (HER-2/neu: CEP17 signal ratio: 1.11)

The following guideline has been established for HER-2/neu testing:
Normal HER-2/neu levels: <1.80; Equivocal HER-2/neu levels: 1.80 – 2.20; Amplified HER-2/neu levels: >2.20
(Ref: Arch Pathol Lab Med. 2007; 131:18-43)

NOTES

The LSI HER-2/neu probe is specific for the HER-2/neu gene Locus (17q 11.2-q12) and the CEP 17 DNA probe is specific for the alpha satellite DNA sequence at the centromeric region of Chromosome 17 (17 p11.1 -q11.2).

This test has been cleared and approved for specific uses by the U.S. Food and Drug Administration. Its system is operating within the performance specifications stated in the product insert.

Released by:

------END OF REPORT------

ICD-0-3
carcinoma, infiltrating duct, NOS 8500/3
Site: breast, NOS C50.9
5/23/11

Criteria 5/23/11	Yes	No
Diagnosis Discrepancy		✓
Primary Tumor Site Discrepancy		✓
HIPAA Discrepancy		✓
Prior Malignancy History		✓
Dual/Synchronous Primary Noted		
Case is (circle): QUALIFIED / DISQUALIFIED		
Reviewer Initials MG Date Reviewed 5/19/11		

ACC # 26

A) ADDITIONAL MARGIN OF MOST MEDIAL TUMOR, EXCISION:
Benign breast tissue, no tumor present.

(B) LEFT BREAST, SEGMENTAL MASTECTOMY:
MULTIFOCAL INVASIVE DUCTAL CARCINOMA WITH ASSOCIATED LYMPHOCYTIC INFILTRATE, HIGH NUCLEAR GRADE, NOTTINGHAM HISTOLOGIC GRADE 3. (SEE COMMENT)
LARGEST FOCUS OF INVASIVE CARCINOMA MEASURES 1.2 CM IN GREATEST DIMENSION IN THE MEDIAL ASPECT OF THE SPECIMEN.
MULTIPLE SATELLITE FOCI OF INVASIVE CARCINOMA ARE LATERAL TO THE MAIN FOCUS AND MEASURE 5 MM (X 2), 4 MM, 3 MM AND 1 MM.
LYMPHOVASCULAR INVASION IDENTIFIED.
DUCTAL CARCINOMA IN SITU (DCIS), HIGH GRADE, PREDOMINANTLY CRIBRIFORM AND CLINGING TYPES WITH CANCERIZATION OF LOBULES AND ASSOCIATED LYMPHOCYTIC INFILTRATE.
DCIS EXTENDS IN AN APPARENT IRREGULAR DISTRIBUTION OVER A 4 CM AREA.
DCIS EXTENDS TO LESS THAN 1 MM FROM THE ANTERIOR MARGIN, TO 1 MM FROM THE INFERIOR MARGIN, AND TO 2 MM FROM THE SUPERIOR AND POSTERIOR MARGINS.
INVASIVE CARCINOMA EXTENDS TO 2 MM FROM THE ANTERIOR MARGIN. (SEE COMMENT)

(C) ADDITIONAL CENTRAL ANTERIOR INFERIOR MARGIN, EXCISION:
Benign breast tissue, no tumor present.

(D) LEFT AXILLARY CONTENTS, DISSECTION:
METASTATIC CARCINOMA IN ONE OF THIRTY LYMPH NODES (1/30); METASTASIS MEASURES 7 MM; NO EXTRANODAL EXTENSION IDENTIFIED.

(E) ADDITIONAL LEVEL I LEFT AXILLARY TISSUE, EXCISION:
Two lymph nodes, no tumor present (0/2).

Entire report and diagnosis completed by

COMMENT

Additional tissue was re-excised, and invasive carcinoma does not approach the final margins. However, I with involvement of lobules is 1 mm from the final inferior margin and 2 mm from the final superior and posterior margins.
Tumor marker studies will be performed and reported in an addendum.

UUID:CA6B733A-6030-422C-9DE9-B9718CBB2FDC
TCGA-GM-A2DF-01A-PR Redacted

GROSS DESCRIPTION

(A) ADDITIONAL MARGIN OF MOST MEDIAL TUMOR - Received is a piece of fibroadipose tissue (1.8 x 1.2 x 0.5 cm) with clips on the true margin. The true margin inked black. The specimen is serially sectioned and entirely submitted in A.
(B) LEFT SEGMENTAL MASTECTOMY - A segmental mastectomy specimen (8.0 x 6.5 x 5.5 cm) with a short stitch at the superior aspect, long stitch at the lateral aspect, and multiple clips on the anterior aspect. The specimen is serially sliced from medial to lateral into 11 slices. A well-circumscribed tan-pink tumor 1.4 x 1.1 x 1.0 cm, with an associated biopsy clip is identified in

slice #3, which is 0.7 cm from the closest anterior margin. Multiple smaller nodules are identified lateral to the main tumor. One of these is very close to the anterior margin in the central anterior inferior aspect of the specimen, and additional tissue is requested from this margin.

INK CODE: Anterior - yellow, posterior - black, superior - blue, inferior - green, medial and lateral - red.

SECTION CODE: B1, medial perpendicular margin; B2, tissue adjacent to tumor from slice #2 ; B3-B7, tumor and margins from slice #3 (B3 and B4, tumor with anterior margin in area of clip; B5, inferior margin; B6, posterior margin; B7, superior margin); B8, fibrous tissue adjacent to the tumor from slice #4; B9, one small nodule, 1.2 cm from superior margin and 1.8 from anterior margin of slice #4; B10, one small nodule 1.0 cm from anterior margin and 1.2 cm from superior margin of slice #5; B11, one small nodule, 1.5 cm from anterior margin, 2.0 cm from superior margin of slice #5; B12, B13, one small nodule, 0.1 cm from anterior margin and 1.2 cm from inferior margin of slices #6 and #7; B14, one small nodule 0.4 cm from posterior margin and 1.5 cm from inferior margin of slice #4; B15-B18, remainder of margins of slice #4, entirely submitted (B15, anterior margin; B16, superior margin; B17, posterior margin; B18, inferior margin); B19, representative section from slice #8; B20, lateral perpendicular margin.

(C) ADDITIONAL CENTRAL ANTERIOR INFERIOR MARGIN, LEFT BREAST – A portion of fibroadipose tissue (2.0 x 1.3 x 0.5 cm) with clips on the true margin. The true margin is inked black. The specimen is serially sectioned and entirely submitted in C1-C2.

(D) LEFT AXILLARY CONTENTS - Adipose tissue (11.0 x 7.5 x 3.0 cm). Multiple lymph nodes are identified ranging from 0.2 x 0.2 x 0.2 cm to 2.5 x 1.5 x 0.8 cm.

SECTION CODE: D1-D3, each containing six lymph nodes; D4, four lymph nodes; D5, four lymph nodes; D6, one lymph node, trisected; D7, one lymph node, serially sectioned: D8, one lymph node, serially sectioned; D9 and D10, one lymph node, serially sectioned.

(E) ADDITIONAL LEVEL I LEFT AXILLARY TISSUE - Two lymph nodes (0.2 x 0.2 x 0.2 cm and 0.8 x 0.4 x 0.4 cm), entirely submitted.

SECTION CODE: E1, one lymph node; E2, one lymph node, trisected.

CLINICAL HISTORY

Left breast cancer.

SNOMED CODES

T-04050, M-85003, M-85002 T-C4710, M-85006

"Some tests reported here may have been developed and performance characteristics determined by These tests have not been specifically cleared or approved by the U.S. Food and Drug Administration."

Released by:

Start of ADDENDUM #1

ADDENDUM

Addendum completed by

This report is issued to give immunohistochemistry results.

Immunohistochemical staining is performed on a representative formalin-fixed, paraffin-embedded section of INVASIVE DUCTAL CARCINOMA, left breast, block B3.

MARKER	RESULTS	% POSITIVE	SCORE
Estrogen Receptor	Negative	0 %	N/A
Progesterone Receptor	Negative	< 1 %	N/A
HER-2/neu overexpression	Negative		Focal 1+

Due to the above HER-2/neu immunohistochemical staining result, gene copy level (HER-2/neu: CEP17 signal ratio) will be evaluated by FISH and a separate report will be issued.

FOOTNOTE

Estrogen receptor was assessed by immunohistochemistry using antibody 6F11 (

Progesterone receptor was assessed by immunohistochemistry using antibody PgR1294(

HER-2/neu was assessed by immunohistochemistry using antibody AB8 (l

Released by:

Start of ADDENDUM #2

ADDENDUM #2

This modified report is being issued to report the results of HER-2/*neu* FISH

Addendum completed by

SPECIMEN SOURCE

Left breast

SUMMARY

Tissue section of the invasive carcinoma B3 was evaluated for HER-2/neu gene amplification by interphase fluorescence in situ hybridization technique using the HER-2/neu DNA Probe Kit (LSI HER-2/neu /CEP17

Slide adequacy is satisfactory. Sixty tumor nuclei were counted and showed an average of **1.57** copies of LSI HER-2/neu gene per nucleus and an average of **1.40** copies of CEP17 per nucleus.

Negative and positive controls (established by) with this batch are appropriate.

Two representative images have been archived.

INTERPRETATION
The tumor cells demonstrated **no amplification** of the HER-2/neu gene copy levels (HER-2/neu: CEP17 signal ratio: **1.12**)

The following guideline has been established for HER-2/neu testing:
Normal HER-2/neu levels: < 1.80; equivocal HER-2/neu levels: 1.80 – 2.20; amplified HER-2/neu levels: > 2.20
(Ref: Arch Patholo Lab Med. 2007; 131:18-43)

NOTES
The LSI HER-2/neu probe is specific for the HER-2/neu gene Locus (17q 11.2-q12) and the CEP 17 DNA probe is specific for the alpha satellite DNA sequence at the centromeric region of Chromosome 17 (17 p11.1 -q11.2).

This test has been cleared and approved for specific uses by the U.S. Food and Drug Administration. Its system is operating within the performance specifications stated in the product insert.

Released by:

------END OF REPORT------

UUID:E8601979-AE05-4E4D-BB39-6B9C5E67D050
TCGA-LD-A9QF-01A-PR Redacted

Surgical Pathology Report

[redacted]

DATE OBTAINED:
DATE RECEIVED:
DATE REPORTED:

LOCATION:
SUBMITTING MD:
CC:

DIAGNOSIS

1. **LEFT AXILLARY "SENTINEL" LYMPH NODES: NEGATIVE (0/3); NEGATIVE CUTDOWNS SECTIONS.**
2. **LEFT AXILLARY "PALPABLE" LYMPH NODES: NEGATIVE (0/4); NEGATIVE CUTDOWNS SECTIONS.**
3. **LEFT AXILLARY "SENTINEL" LYMPH NODE: NEGATIVE (0/1); NEGATIVE CUTDOWNS SECTIONS.**
4. **LEFT AXILLARY "SENTINEL" LYMPH NODES #3: NEGATIVE (0/6); NEGATIVE CUTDOWNS SECTIONS.**
5. **LEFT TOTAL MASTECTOMY: INVASIVE AND INTRADUCTAL MAMMARY DUCT CARCINOMA.**

**SIZE (INVASIVE):**	**1.6 cm (additional incidental < 1mm microinvasion)**		
**SIZE (DCIS COMPONENT):**	**~11 cm scattered foci (subareolar region to 11 cm from nipple @ 12-1 o'clock)**		
**LATERALITY:**	**Left**		
**TUMOR FOCALITY:**	**Unifocal gross reference mass; incidental microinvasive lesion**		
**LESIONAL SITE:**	**Subareolar (both reference mass and incidental microinvasion)**		
**HISTOLOGIC TYPE:**	**Invasive ductal carcinoma, apocrine type**		
**NUCLEAR GRADE:**	**III of III**		
**HISTOLOGIC GRADE:**(EEmSBR)	**III of III** (Tubules score 3 + NG score 3 + mitoses score 3)		
**IN-SITU COMPONENT:**	**DCIS (variable histology)**<br>comedo NG3 subareolar<br>cribriform non-apocrine with comedonecrosis NG2-3 UOQ<br>cribriform apocrine without comedonecrosis NG2 UOQ		
**LYMPH NODE SAMPLING:**	**Negative (0/14); see specimen #1 - #4**		
**AJCC CATEGORIES:**	**Stage I (assuming "cM0" status)**		
**pTNM:**	**pT1c**	**pN0**	
**cTNM:**	**cT1**	**cN0**	**cM0**
**INTEGRITY/ORIENTATION:**	**Intact specimen with designated margins**		
**MARGINS (invasive and DCIS):**	**Negative; 3 cm to nearest margins (anterior & posterior locations)**		
**LYMPHOVASCULAR INVASION:**	**Focal**		
**MICROCALCIFICATIONS:**	**Present (associated with comedo-DCIS)**		
**NIPPLE/SKIN:** (if applicable)	**Negative nipple; numerous pigmented seborrheic keratoses & simple lentigines**		
**SKELETAL MUSCLE**	**Not present**		
**OTHER:**	**Two core biopsy sites (1 o'clock subareolar; 12-1 o'clock UOQ); focal microcysts, mammary duct stasis/ectasia and focal usual duct hyperplasia**		

ICD-O-3
Carcinoma, infiltrating duct 8500/3
Site (L) Breast NOS C50.9
[illegible] 1/27/14

Electronically Signed Out

COMMENT

1-88329,88307 2-88333,88307 3-88333,88307 4-88307 5-88309

[redacted].

Clinical Diagnosis and History:

ear old female with left invasive CA/DCIS ? extensive DCIS 12-1:00
cT1,cNo,cMo clinical stage I

Tissue(s) Submitted:

1: LEFT AXILLARY SENTINEL LYMPH NODES #1
2: LEFT AXILLARY PALPABLE LYMPH NODES
3: LEFT AXILLARY SENTINEL LYMPH NODE #2
4: LEFT AXILLARY SENTINEL LYMPH NODE #3
5: LEFT TOTAL MASTECTOMY SUTURE IN AXILLARY TAIL

Gross Description:

Specimen #1 is received fresh for intraoperative consultation labeled left axillary sentinel lymph nodes, and consists of three lymph nodes with attached fat, ranging from 0.2 cm to 2.4 cm in greatest dimension. The lymph nodes are submitted in their entirety for permanent microscopy as follows:
1A: two lymph nodes
1B: one lymph node serially sectioned

Specimen #2 is received fresh for intraoperative consultation labeled left axillary palpable lymph nodes, and consists of four lymph nodes, ranging from 0.8 cm to 1.5 cm in greatest dimension. The lymph nodes are serially sectioned to reveal predominantly pink-tan cut surfaces, one of which displays a white focus. A smear preparation from the lymph node with the white focus is performed. The lymph nodes are submitted in their entirety for permanent microscopy as follows:
2A-D: one lymph node per cassette

Specimen#3 is received fresh for intraoperative consultation labeled left axillary lymph node, sentinel #2, and consists of one, 0.9 cm lymph node, which is serially sectioned and submitted in its entirety labeled 3A.

Specimen #4 is received in formalin labeled left axillary sentinel lymph node #3, and consists of six lymph nodes ranging from 0.2 cm to 0.7 cm in greatest dimension. The largest lymph node is bisected and the lymph nodes are submitted in their entirety as follows:
4A: five lymph nodes
4B: one lymph node, bisected

Specimen #5 is received fresh labeled left total mastectomy, suture in axillary tail, and consists of an 858 gm, 25 x 23 x 5 cm left total mastectomy specimen, with a suture marking the axillary tail. There is a 24 x 17 cm, ovoid to elliptical portion of brown skin on the anterior aspect, displaying a 2 x 2 x 1 cm everted nipple. The skin displays multiple brown-black, flat to raised, predominantly keratotic skin lesions, ranging from 0.1 cm to 1.0 cm and 0.6 to 0.2 cm. The largest lesion is located at 12 o'clock, 1.7 cm from the nearest 12 o'clock margins. One of the smaller lesions appears to be transected at the 5 o'clock margin. The specimen is inked as follows: superoanterior – blue, inferoanterior – green, posterior – black. The posterior margin is intact. The specimen is serially sectioned to reveal a 1.6 x 1.2 x 1.2 cm tan-white, ovoid, lobulated, indurated mass located retroareolar, 6 cm from the deep margin and 3 cm from the overlying skin. Sectioning of the mass reveals a clip. Anterior to the mass is some dense fibrosis, displaying possible comedonecrosis. Sectioning of the remaining fibrous tissue reveals a 1.2 x 1.0 x 0.6 cm rubbery, but stellate area of fibrosis at 1-2 o'clock, 11 cm from the nipple. Sectioning of this area reveals a clip. The clip is located 3 cm from the deep margin, and 6.5 cm from the overlying skin. No definitive gross tumor is associated with this clip. The remaining breast parenchyma consists of approximately 90% yellow, lobulated adipose tissue, and 10% scattered white, rubbery fibrous tissue. A small portion of tumor and normal are submitted for TCGA studies. Representative sections are submitted as follows:
5A: nipple
5B-5D: mass with clip, no margin
5E-5G: representative fibrous tissue anterior to mass, no margin
5H: representative deep margin to mass
5I-5K: stellate area of fibrosis with clip
5L: posterior margin and anterior skin to stellate area of fibrosis with clip
5M-5O: representative fibrous tissue at 12-1 o'clock
5P: representative upper inner quadrant
5Q: representative lower inner quadrant
5R: representative lower outer quadrant
5S: representative upper outer quadrant
5T: representative margin in second largest skin lesion
5U: representative medium five skin lesions
5V: representative smaller skin lesions
Time in formalin: 1 p.m, submitted same day

Intraoperative Consult Diagnosis

1A THREE NEGATIVE LYMPH NODES (BY GROSS).

2A FOUR LYMPH NODES IDENTIFIED; THREE NEGATIVE (BY GROSS); ONE NEGATIVE (BY GROSS AND SMEAR)

3A ONE NEGATIVE LYMPH NODE (BY GROSS AND SMEAR).

END OF REPORT

Criteria lw 12/20/13	Yes	No
Diagnosis Discrepancy		✓
Primary Tumor Site Discrepancy		✓
HIPAA Discrepancy		✓
Prior Malignancy History		✓
Dual/Synchronous Primary Noted		✓
Case is (circle): QUALIFIED / DISQUALIFIED		
Reviewer Initials [illegible] Date Reviewed: 12/2[illegible]2013		

UUID:4D4811FB-5BF1-4574-975B-1740602E43D8
TCGA-LL-A5YN-01A-PR
Redacted

RUN DATE:
RUN TIME:
RUN USER:

Inquiry

PATIENT: [redacted]
REG DR: [redacted]
ACCT #: [redacted]
AGE/SX [redacted]
DOB: [redacted]
STATUS:
LOC:
ROOM:
BED:
TLOC:
U # [redacted]
REG: [redacted]
DIS:

SPEC #:

COLL:

TIME IN FORMALIN: 4:43 hrs.

CLINICAL INFORMATION:
Pre-Op Diagnosis:
Remarks:
Specimen(s):
A. Right breast lumpectomy
B. Sentinel lymph node #1
C. Sentinel lymph node #2
D. Sentinel lymph node #3

ICD-O-3
Carcinoma, ductal infiltrating NOS 8500/3
Site (R) Breast NOS C50.9
4/2/13

MICROSCOPIC DIAGNOSIS

A. RIGHT BREAST LUMPECTOMY:
- INVASIVE DUCTAL CARCINOMA
- SEE COMMENT FOR DETAILS

B. SENTINEL LYMPH NODE #1:
- ONE BENIGN LYMPH NODE

C. SENTINEL LYMPH NODE #2:
- ONE BENIGN LYMPH NODE

D. SENTINEL LYMPH NODE #3:
- THREE BENIGN LYMPH NODES

COMMENT(S)

PROTOCOL FOR EXAMINATION OF SPECIMENS WITH INVASIVE CARCINOMA OF THE BREAST BASED ON AJCC/UICC TNM, 7TH EDITION
The following classification should be adjusted based on additional clinical information.

SPECIMEN:	Partial breast
PROCEDURE:	Excision without wire-guided localization
LYMPH NODE SAMPLING:	Sentinel lymph nodes
SPECIMEN INTEGRITY:	Single intact specimen
SPECIMEN SIZE:	Greatest dimensions: 10.5 x 9.5 x 4.5 cm
SPECIMEN LATERALITY:	Right
TUMOR SIZE:	Greatest dimension: 2.4 cm
TUMOR FOCALITY:	Single focus of invasive carcinoma
EXTENT OF TUMOR:	Skin: invasive carcinoma does not invade into dermis or epidermis
	Muscle: no skeletal muscle present
DUCTAL CARCINOMA IN SITU:	DCIS is present

** CONTINUED ON NEXT PAGE **

SPEC #: PATIENT: [redacted] [redacted] (Continued)

COMMENT(S) (Continued)

	Extensive intraductal component negative
LOBULAR CARCINOMA IN SITU:	Present
HISTOLOGIC TYPE:	Invasive ductal carcinoma
HISTOLOGIC GRADE:	Tubular differentiation score: 3
	Nuclear pleomorphism score: 3
	Mitotic count score: 2
	Overall grade: 3
MARGINS:	Margins uninvolved by invasive carcinoma
	Distance from closest margin: 1.3 cm, superior
	Margins uninvolved by ductal carcinoma
	Distance from closest margin: 1.3 cm, superior
LYMPH NODES:	Number of sentinel lymph nodes examined: 5
	Total number of lymph nodes examined: 5
	Number of lymph nodes with macrometastases: 0
	Number of lymph nodes with micrometastases: 0
	Number of lymph nodes with isolated tumor cells: 0
PATHOLOGIC STAGING:	Primary tumor: pT2
	Regional lymph nodes: pN0 (i-)
	Distant metastasis: not applicable

GROSS DESCRIPTION:
A. Received fresh for tissue banking and gross evaluation labeled with the patient's name and designated "right breast lumpectomy" is a 162 gram, 10.5 x 9.5 x 4.5 cm fibrofatty breast tissue biopsy. The biopsy has an overlying 8.5 x 2.0 cm black-brown skin ellipse. The ellipse is oriented as anterior. There is a short suture superior and a long suture lateral. The margins will be inked per orientation as labeled: blue superior, black inferior, yellow lateral, red medial and green deep or posterior. The biopsy is serially sectioned from lateral to medial to have a 2.4 x 1.8 x 1.5 cm tumor mass. The mass is centrally located within the specimen and is 1.3 cm from the nearest superior margin, is 1.5 cm from inferior, is at least 2.5 cm from deep as well as superficial and is more than 3 cm from medial or lateral. The tumor has adjacent minor hemorrhage and fat necrosis consistent with a previous biopsy. A section of tumor is sampled for tissue banking. There is diffuse dense white fibrous tissue surrounding the tumor mass. The white fibrous tissue has focal fibrocystic changes, and the white fibrous tissue makes up approximately 50% of the biopsy parenchyma. Representative sections are sampled from across the specimen to include tumor to the nearest margins as labeled:

A1	-	nearest perpendicular sections of lateral margin sampled
A2-A3	-	sections showing lateral dense white fibrous tissue with fibrocystic change
A4	-	section of tumor with adjacent petechial hemorrhage
A5	-	nearest deep margin
A6	-	nearest superficial skin margin
A7	-	tumor to nearest inferior margin
A8-A9	-	tumor to nearest superior margin
A10	-	tumor with adjacent fat necrosis and petechial biopsy hemorrhage
A11	-	near full cross section of tumor sampled
A12	-	perpendicular sections of medial margin sampled

** CONTINUED ON NEXT PAGE **

SPEC #: PATIENT: [redacted] [redacted] (Continued)

GROSS DESCRIPTION: (Continued)

B. Labeled "sentinel lymph node #1" is a nodular, fatty 2.4 x 2.0 x 1.8 cm lymph node. There is scant yellow adipose on the surface of the node. The node is serially sectioned perpendicular to the long axis to be entirely submitted per sentinel lymph node protocol in cassettes B1-B4.

C. Labeled "sentinel lymph node #2" is a nodular, fatty 2.0 x 1.7 x 1.4 cm lymph node. The fat is trimmed from the node, and the node is 1.2 x 0.7 x 0.6 cm. The node is sectioned perpendicular to the long axis to be entirely submitted per sentinel lymph node protocol in cassettes C1-C2.

D. Labeled "sentinel lymph node #3" is a 3.5 x 3.0 x 1.5 cm aggregate of yellow adipose. The adipose is sectioned to have three nodular lymph nodes. The nodes are 0.3 cm, 0.7 x 0.5 x 0.2 cm and 0.8 x 0.7 x 0.4 cm. The larger nodes are sectioned perpendicular to the long axis, and the nodes are entirely submitted for sentinel lymph node protocol separately from smallest to largest in cassettes D1-D3.

INTRAOPERATIVE CONSULTATION:
A. INTRAOPERATIVE CONSULTATION, RIGHT BREAST LUMPECTOMY:
- GROSS TUMOR PRESENT, ADEQUATE FOR TISSUE BANKING WITH NEOPLASTIC TISSUE PROVIDED TO TISSUE BANK COORDINATOR
- SURGICAL MARGINS NEGATIVE WITH CLOSEST MARGIN SUPERIOR MEASURING 1.5 CM
- RESULTS GIVEN TO DR.

PHOTO DOCUMENTATION

Image .

Signed ____(signature on file)____

** END OF REPORT **

Criteria lw 3/15/13	Yes	No
Diagnosis Discrepancy		✓
Primary Tumor Site Discrepancy		✓
HIPAA Discrepancy		✓
Prior Malignancy History		✓
Dual/Synchronous Primary Noted DCIS	✓	
Case is (circle): QUALIFIED / DISQUALIFIED		
Reviewer Initials KMT Date Reviewed: 3/6/13		

RUN DATE:
RUN TIME:
RUN USER [redacted]

PATIENT: [redacted] ACCT #: [redacted]
AGE/SX: [redacted] ROOM: [redacted]
REG DR: [redacted] DOB: [redacted] BED: [redacted]
STATUS: [redacted]

SPEC #: RECD: STATUS: [redacted]
COLL: TIME IN FORMALIN: hrs.
COLD ISCHEMA TIME: mins.

CLINICAL INFORMATION:
Pre-Op Diagnosis: Left breast cancer
Remarks:
Specimen(s): A. Left breast - green is 12 o'clock
B. Left axilla, level 1 and 2

MICROSCOPIC DIAGNOSIS

A. LEFT BREAST (TOTAL MASTECTOMY):
- INVASIVE DUCTAL CARCINOMA, TWO SEPARATE FOCI
- COMBINED HISTOLOGIC GRADE 3 OF 3
- LARGEST INVASIVE CARCINOMA MEASURES 2.8 CM IN GREATEST DIMENSION
- HIGH-GRADE DUCTAL CARCINOMA IN SITU PRESENT
- MARGINS UNINVOLVED BY INVASIVE CARCINOMA WITH CLOSEST MARGIN DEEP AT 4 MM
- MARGINS UNINVOLVED BY IN SITU CARCINOMA WITH CLOSEST MARGIN DEEP AT 1 MM
- SEE COMMENT FOR SYNOPTIC REPORT

B. LEFT AXILLARY LYMPH NODES, LEVELS 1 AND 2 (DISECTION):
- METASTATIC CARCINOMA IN ONE OF FOUR LYMPH NODES

ICD-O-3
Carcinoma, infiltrating duct 8500/3
Site (L) Breast NOS C50.9
(L) Breast, central portion C50.1
10/10/13

COMMENT(S)

CAP APPROVED SURGICAL PATHOLOGY CANCER CASE SUMMARY: INVASIVE CARCINOMA OF THE BREAST

PROCEDURE:	Total mastectomy
LYMPH NODE SAMPLING:	Axillary dissection, levels 1 and 2
SPECIMEN LATERALITY:	Left
HISTOLOGIC TYPE OF INVASIVE CARCINOMA:	Invasive ductal carcinoma
TUMOR SIZE:	Greatest dimension of largest focus of invasion: 2.8 cm
HISTOLOGIC GRADE:	NOTTINGHAM HISTOLOGIC SCORE:<br>Glandular/tubular differentiation: Score 3<br>Nuclear pleomorphism: Score 3<br>Mitotic rate: Score 3<br>Overall grade: Grade 3
TUMOR FOCALITY:	Multiple foci of invasive carcinoma<br>Number of foci: 2<br>Sizes of individual foci: 2.8 cm and 1.3 cm
DUCTAL CARCINOMA IN SITU:	DCIS is present

** CONTINUED ON NEXT PAGE **

SPEC #: [redacted] [redacted] (Continued)

COMMENT(S) (Continued)

Size of DCIS: Estimated size of DCIS at least 5 cm
Nuclear grade: Grade III
Necrosis: Present, central

MARGINS:
Invasive carcinoma: Margins uninvolved by invasive carcinoma
Distance from closest margin: 4 mm to deep
DCIS: Margins uninvolved by DCIS
Distance from closest margin: 1 mm to deep

LYMPH NODES:
Total number of lymph nodes examined: 4
Number of lymph nodes with metastasis: 1
Size of largest metastatic deposit: 7 mm
Extranodal extension: Not identified

PATHOLOGIC STAGING:
Primary tumor: pT2
Regional lymph nodes: Category: pN1a
Distance metastasis: Not applicable

ANCILLARY STUDIES:
PERFORMED ON
Estrogen receptor: Positive (100% of tumor cells with nuclear positivity)
Average intensity of tumor cell nuclei staining: strong
Progesterone receptor: Positive (2% of tumor cells with nuclear positivity)
Average intensity of tumor cell nuclei staining: weak
HER2: Immunoperoxidase studies: Positive (Score 3+)

GROSS DESCRIPTION:
Received fresh for tissue banking labeled with the patient's name and designated "left breast" is a 1,094 gram, 24.0 x 23.0 x 5.5 cm fibrofatty breast. The breast has an overlying 22.0 x 8.5 cm portion of tan-white skin. The skin has a central 6.5 x 5.5 cm areola and 1.5 cm raised nipple. There is a green suture on the skin designating 12 o'clock. An indurated nodule is present beneath the skin surface directly medial to the nipple and underlying the areola. The skin surface and areola have multiple plaque-like pigmented papules. The largest papule is superior and lateral at the edge of the areola. This papule is 1.6 x 0.8 cm. No additional scars or lesions are identified on the skin surface. The deep margin is ragged and fatty. The specimen is consistent with a simple mastectomy specimen.

The deep margin is inked blue, and the breast is sectioned to have a diffusely glistening fatty cut surface. A medial, 2.3 cm nodular tumor mass is present beneath the skin surface medial in the described area beneath the areola. The tumor has a focal multinodular appearance at the periphery, but appears to be one mass grossly. The mass is 0.7 cm from the skin surface, is 6.0 cm from superior, is 3.0 cm from inferior, is 5.0 cm from medial, and 18.5 cm from lateral. The tumor is at least 4.5 cm from the deep margin. The tumor has a central stellate, gritty indurated cut surface, and further sectioning shows the tumor to range up to 2.8 cm. A section of tumor is sampled for tissue banking. The remainder of the parenchyma is diffusely fatty with scant, delicate white fibrous tissue. No additional tumor-like masses are identified. The delicate white fibrous tissue makes up between 10-15% of the parenchyma. The tumor is most closely associated with the lower

** CONTINUED ON NEXT PAGE **

SPEC #: [redacted] [redacted] (Continued)

GROSS DESCRIPTION: (Continued)
inner quadrant. Further sectioning of the lower inner quadrant shows a deeper, separate, 1.3 x 0.7 x 0.5 cm possible tumor mass. This second described mass is 0.3 cm from the deep margin and is associated with the lower inner quadrant. This separate tumor is 5 cm from the first described tumor underlying the areola. The second mass is 4.5 cm from superior, 4.0 cm from inferior, 2.8 cm from medial, 20 cm from lateral. Representative sections are sampled as labeled:

A1 - nipple entirely submitted
A2 - en face section of areola
A3 - pigmented skin papule
A4 - deep margin associated with first described tumor mass
A5-7 - sections of first described tumor mass to include mass to skin
A8-10 - separate smaller circumscribed-appearing tumor mass at deep margin, entirely submitted
A11 - upper outer quadrant
A12 - lower outer quadrant
A13 - upper inner quadrant
A14 - lower inner quadrant (quadrant most closely associated with both tumor masses

B. Received in formalin, labeled with the patient's name and "left axilla level 1 and 2" is a 5.0 x 4.0 x 2.5 cm aggregate of yellow adipose. The adipose is trimmed to have four nodular, fatty lymph nodes. The nodes range from 1.2 cm to 2.5 x 1.9 x 1.1 cm. The fat is trimmed and the nodes are sectioned to be entirely submitted as labeled:

B1 - two lymph nodes with one lymph node bisected
B2 - one lymph node bisected
B3 - the largest lymph node bisected

PHOTO DOCUMENTATION

Signed _____(signature on file)_____ [redacted]

** END OF REPORT **

Criteria *9/23/13*	Yes	No
Diagnosis Discrepancy		✓
Primary Tumor Site Discrepancy		✓
HIPAA Discrepancy		✓
Prior Malignancy History		✓
Dual/Synchronous Primary Noted		✓
Case is (circle): QUALIFIED / DISQUALIFIED		
Reviewer Initials [illegible] Date Reviewed: *9/18/13*		

RUN DATE: [redacted] [redacted] PAGE 1
RUN TIME: [redacted]
RUN USER: [redacted]

PATIENT: [redacted] ACCT #: [redacted] LOC: [redacted] U #:
AGE/SX: [redacted] ROOM: [redacted] REG:
REG DR: [redacted] DOB: [redacted] BED: DIS:
STATUS: TLOC:

SPEC #: [redacted] RECD: [redacted] STATUS: PERFORMED AT [redacted]
COLL: TIME IN FORMALIN:

CLINICAL INFORMATION:
Pre-Op Diagnosis:
Remarks: Tag = axillary / tail specimen #3
Specimen(s): A. Sentinel node #1 left breast
B. Sentinel node #2
C. Left simple mastectomy

UUID:389044F0-9D90-4639-8C42-C1A6EA816442
TCGA-LL-A442-01A-PR Redacted

MICROSCOPIC DIAGNOSIS

A. ONE LYMPH NODE, SENTINEL NODE #1 LEFT BREAST, LYMPHADENECTOMY:
- NO TUMOR SEEN IN MULTIPLE STEP SECTIONS STAINED WITH ROUTINE AND PAN KERATIN IMMUNOCHEMICAL STAINS

B. THREE LYMPH NODES, SENTINEL NODE #2, LYMPHADENECTOMY:
- NO TUMOR SEEN IN MULTIPLE STEP SECTIONS STAINED WITH ROUTINE AND PAN KERATIN IMMUNOCHEMICAL STAINS

C. LEFT BREAST, TOTAL MASTECTOMY:
- TWO AREAS OF INFILTRATING DUCTAL CARCINOMA (IDENTICAL MORPHOLOGY)
- MODIFIED NOTTINGHAM HISTOLOGIC GRADE 2 OF 3: NUCLEAR SCORE 2 OF 3, TUBULAR FORMATION SCORE 3 OF 3, MITOTIC SCORE 1 OF 3 (TWO MITOTIC FIGURES PER SQUARE MILLIMETER)
- LARGER TUMOR MEASURES 2.7 CM (pT2)
- EXTENSIVE DUCTAL CARCINOMA IN SITU, LOW GRADE WITH SOLID PATTERN
- SURGICAL MARGINS FREE OF TUMOR WITH NEAREST MARGIN TO INVASIVE TUMOR 0.5 CM, THE DEEP MARGIN (R0)
- SEE COMMENT FOR SYNOPTIC REPORT

ICD-O-3
carcinoma, infiltrating ductal, NOS
8500/3
Site: breast, NOS C50.9
8/23/12 RD

COMMENT(S)

SURGICAL PATHOLOGY CANCER CASE SUMMARY - APPROVED BY COLLEGE OF AMERICAN PATHOLOGISTS

PROCEDURE:	Total mastectomy
LYMPH NODE SAMPLING:	Sentinel lymph nodes
SPECIMEN LATERALITY:	Left
HISTOLOGIC TYPE OF INVASIVE CARCINOMA:	Invasive ductal carcinoma
TUMOR SIZE:	Greatest dimension: 27 mm
HISTOLOGIC GRADE:	Glandular/tubular differentiation: score 3
	Nuclear pleomorphism: score 2
	Mitotic rate: score 1

** CONTINUED ON NEXT PAGE **

Criteria	Yes	No
Diagnosis Discrepancy		X
Primary Tumor Site Discrepancy		X
HIPAA Discrepancy		X
Prior Malignancy History	X	X
Dual/Synchronous Primary Noted		X
Case is (circle): QUALIFIED / DISQUALIFIED		
Reviewer Initials [illegible] Date Reviewed: 8/17/12		

8/19/12

COMMENT(S) (Continued)

Overall grade: grade 2
TUMOR FOCALITY: Multiple foci of invasive carcinoma
DUCTAL CARCINOMA IN SITU: DCIS is present, positive for EIC
MARGINS: Invasive carcinoma:
Margins uninvolved by invasive carcinoma
Distance from closest margin: 5 mm
DCIS:
Margins uninvolved by DCIS
LYMPH NODES: Number of sentinel lymph nodes examined: 4
Total number of lymph nodes examined: 4
Number of lymph nodes with macrometastases: 0
Number of lymph nodes with micrometastases: 0
Number of lymph nodes with isolated tumor cells: 0
PATHOLOGIC STAGING: Primary tumor: pT2
Regional lymph nodes:
Modifier: (sn)
Category: pN0 (i-)
Distant metastasis: not applicable
ANCILLARY STUDIES: Estrogen receptor:
Results: positive (100% of tumor cells with nuclear positivity)
Progesterone receptor:
Results: positive (100% of tumor cells with nuclear positivity)
Immunoperoxidase studies:
Results: equivocal (score 2+)
In situ hybridization (FISH):
Results: not amplified

GROSS DESCRIPTION:
A. Received in formalin labeled with the patient's name and "sentinel node #1, left breast" is a 2.0 x 1.3 x 0.9 cm nodular fatty tissue biopsy. The fat is trimmed, and there is a previously bisected, disrupted 1.5 x 0.8 x 0.6 cm lymph node present. The node is sectioned perpendicular to the long axis to be entirely submitted for sentinel lymph node protocol in cassette A1.

B. Received in formalin labeled with the patient's name and "sentinel node #2, left breast" is a 2.3 x 1.5 x 1.2 cm nodular fatty lymph node. The fat is trimmed, and there are three small lymph nodes present which range up to 0.8 cm. The nodes are sectioned to be entirely submitted per sentinel lymph node protocol as labeled:
B1 - two lymph nodes with one lymph node bisected
B2 - largest lymph node serially sectioned perpendicular to the long axis

C. Received fresh for tissue banking labeled with the patient's name and "left simple mastectomy" is a 481 gram, 17.0 x 16.0 x 4.5 cm fibrofatty breast. The breast is received with an overlying 9.5 x 3.0 cm black-brown skin ellipse. The ellipse has a central, circular 3.0 cm areola which extends to the skin margin and a 1.0 cm central nipple. The

** CONTINUED ON NEXT PAGE **

GROSS DESCRIPTION: (Continued)
breast is absent of an axillary tail and consistent with a simple mastectomy specimen. The deep fascial margin is smooth and will be inked blue. The breast is serially sectioned from medial to lateral to have an inferior, 2.7 x 2.5 x 2.0 cm circumscribed red-brown mass. The mass is more closely associated with the lower outer quadrant. This larger mass comes to within 0.5 cm of the nearest inked deep margin. This mass is inferior and comes to within 5.0 cm of the inferior margin, is 13 cm from the superior aspect, is at least 6.5 cm from lateral and 7.5 cm from medial. There is a clip identified and a separate tumor site which is centrally located between the upper and lower inner quadrant. This smaller tumor is 2.3 cm from the nearest deep margin and is 1.3 x 0.8 x 0.8 cm. The smaller tumor has a localization clip and is 11.0 cm from superior, is 6.5 cm from inferior, is approximately 4 cm from medial and at least 10 cm from lateral. The breast has diffuse dense white fibrous tissue with fibrocystic changes. The white fibrous tissue makes up between 60-75% of the parenchyma. Sections of tumor are sampled for tissue banking. The specimen is sampled as labeled:

C1 - sections of nipple
C2 - areola at base of nipple
C3-C5 - sections of large described brown septated mass sampled for tissue banking in lower outer quadrant with cassette C3 being section of mass nearest deep margin
C6 - deep margin overlying smaller mass with localization clip
C7,C8 - smaller tumor entirely submitted
C9 - upper outer quadrant
C10 - lower outer quadrant (quadrant closest to large tumor mass)
C11 - upper inner quadrant (quadrant associated with smaller tumor with localization clip)
C12 - lower inner quadrant sampled

INTRAOPERATIVE CONSULTATION:
IMMEDIATE GROSS EVALUATION C, LEFT BREAST:
- TUMOR PROCESSED FOR TUMOR BANKING

PHOTO DOCUMENTATION

Image
Image
Image

Signed ____(signature on file)____

** END OF REPORT **

UUID:3AD4AA3C-5F68-43D1-B578-07AB574D4DE3
TCGA-LL-A740-01A-PR Redacted

RUN DATE:
RUN TIME:
RUN USER:

[redacted]

PATIENT: [redacted] ACCT #: [redacted] LOC: [redacted]
AGE/SX: /F ROOM:
REG DR: [redacted] DOB: [redacted] BED:
STATUS:

SPEC #: [redacted] RECD: STATUS: [redacted]
COLL: TIME IN FORMALIN: hrs.
COLD ISCHEMA TIME: mins.

CLINICAL INFORMATION:
Pre-Op Diagnosis: Right breast mass
Remarks:
Specimen(s): A. Right breast needle localized lumpectomy for immediate gross evaluation
B. Sentinel lymph node

ICD-O-3
Carcinoma, infiltrating duct NOS 8500/3
Site (R) Breast NOS C50.9
8/11/13

MICROSCOPIC DIAGNOSIS

A. RIGHT BREAST (EXCISION WITH NEEDLE LOCALIZATION):
- INVASIVE DUCTAL CARCINOMA, 1.7 CM GREATEST DIMENSION
- MARGINS APPEAR UNINVOLVED
- SEE COMMENT FOR SYNOPTIC REPORT

B. SENTINEL LYMPH NODE, RIGHT AXILLA (BIOPSY):
- NO METASTASIS IN ONE LYMPH NODE

COMMENT(S)

SURGICAL PATHOLOGY CANCER CASE SUMMARY - CAP APPROVED

Procedure:	Excision with wire-guided localization
Lymph Node Sampling:	Sentinel lymph node(s)
Specimen Laterality:	Right
Histologic Type:	Invasive ductal carcinoma
Tumor Size:	Greatest dimension of largest focus of invasion >1 mm: 17 mm
Histologic Grade (Nottingham):	Glandular: Score 2<br>Nuclear Pleomorphism: Score 3<br>Mitotic Rate: Score 1<br>Overall Grade: Grade 2
Tumor Focality:	Single focus of invasive carcinoma
Ductal Carcinoma In Situ (DCIS):	DCIS is present, negative for extensive intraductal component (EIC)<br>Architectural Patterns: Comedo, Cribriform<br>Nuclear Grade: Grade III (high)<br>Necrosis: Present, central (expansive "comedo" necrosis)
Margins:	Invasive Carcinoma: Margins uninvolved by invasive

** CONTINUED ON NEXT PAGE **

SPEC #: [redacted] PATIENT: [redacted] [redacted] (Continued)

COMMENT(S) (Continued)

carcinoma
  Distance from closest margin: 2 mm
DCIS: Margins uninvolved by DCIS
  Distance from closest margin: 5 mm

Lymph Nodes:
Number of sentinel lymph nodes examined: 1
Total number of lymph nodes examined (sentinel and nonsentinel): 1
Number of lymph nodes with metastases: 0
Method of Evaluation of Sentinel Lymph Nodes:
H&E, multiple levels, immunohistochemistry

Lymph-Vascular Invasion: Not identified

Pathologic Staging:
Primary Tumor: pT1c
Regional Lymph Nodes:
  Modifier: (sn)
  Category: pN0(i-)
Distant Metastasis: Not applicable

Ancillary Studies:
Performed on
ER: Negative (<1% of tumor cells with nuclear positivity)
PR: Negative (<1% of tumor cells with nuclear positivity)
HER2 Immunoperoxidase Studies: Equivocal (Score 2+)
In Situ Hybridization for HER2 (FISH or CISH):
Not amplified (HER2 gene copy <4.0 or ratio <1.8)

GROSS DESCRIPTION:
A. This specimen is received in the fresh state from the operating room for immediate gross evaluation of the surgical margins and for specimen x-ray. Specimen x-ray is performed as requested demonstrating centrally a metallic clip, the radiographic target lesion. The specimen consists of a portion of breast tissue which measures 7.5 x 6 x 2 cm. A needle is situated in the specimen. The margins have not been differentially tagged. Blue ink is applied to the margins. Sections reveal a firm, tan tumor measuring 1.7 x 1.5 x 1.5 cm located 3 mm from the nearest surgical margin. A representative section of the tumor is removed by aseptic technique and submitted for tumor banking. The following sections are submitted:

1-5 – tumor sectioned and totally submitted, blue ink on margins
6-8 – representative breast and margins

B. This specimen in the fresh state is a soft, tan lymph node measuring 12 x 10 x 7 mm, sectioned and totally submitted as block B1.

** CONTINUED ON NEXT PAGE **

SPEC #: [redacted] PATIENT: [redacted] [redacted] (Continued)

MICROSCOPIC DESCRIPTION:
A. Sections of the breast tumor demonstrate invasive ductal carcinoma with focal apocrine features, combined histologic grade 2 of 3. DCIS is present exhibiting comedo and cribriform growth patterns with necrosis and a high nuclear grade. The DCIS does not appear extensive. Nearby breast tissue uninvolved by tumor demonstrates at least one radial sclerosing lesion and foci of ductal epithelial hyperplasia, focally atypical. The inked margins appear uninvolved. See the comment for a synoptic report.

INTRAOPERATIVE CONSULTATION:
A. IMMEDIATE GROSS EVALUATION RIGHT BREAST MASS:
- RADIOGRAPHIC TARGET LESION PRESENT BY SPECIMEN X-RAY
- SURGICAL MARGINS APPARENTLY FREE OF TUMOR, 2 MM FROM TUMOR

PHOTO DOCUMENTATION

Image
Image

Signed ____(signature on file)____ [redacted]

** END OF REPORT **

Criteria bw 8/1/13	Yes	No
Diagnosis Discrepancy		✓
Primary Tumor Site Discrepancy		✓
HIPAA Discrepancy		✓
Prior Malignancy History		✓
Dual/Synchronous Primary Noted DCIS	✓	
Case is (circle): QUALIFIED / DISQUALIFIED		
Reviewer Initials Kml Date Reviewed: 8/1/13		

ICD-O-3
Carcinoma, Infiltrating Duct NOS 8500/3
Site: (L) Breast, NOS C50.9
[illegible] 12/24/12

UUID:AD80BBA3-DA8E-4DFB-BDA1-FFDC131E9740
TCGA-OL-A5D6-01A-PR Redacted

FINAL PATHOLOGIC DIAGNOSIS
Left simple mastectomy and sentinal node biopsy:
A. Sentinal lymph node #1:
-One lymph node, no tumor (0/1).
B. Sentinel lymph node #2:
-One lymph node, no tumor (0/1).
C. Left breast (mastectomy):
-Intraductal apocrine ductal carcinoma with lobular cancerization and rare foci of
invasion (largest focus 3mm), associated with large area of recent hemorrhage (5 cm),
see below.
-Separate mass of intraductal and infiltrating ductal carcinoma (2.5 cm) inferior to hemorrhagic region.
-Small intraductal papilloma, sclerosing adenosis and radial scar lesion.
-Calcifications in vessel walls.
-Nipple, no tumor.

Breast Pathologic Parameters

1. Intraductal carcinoma:
A. Size: Gross measurement: 5 cm
B. Type: Comedo, solid, apocrine
C. Nuclear grade: High
(Modified Lagios Grading Scheme)
D. Associated features: Necrosis, Cancerization of lobules
2. Invasive carcinoma:
A. Size: Gross measurement: 1.0 x 1.0 x 0.6 cm
B. Composite histologic (modified SBR) grade: III
- Architecture: 3
- Nuclear grade: 3
- Mitotic count: 2
C. Associated ductal carcinoma in situ (DCIS):
-Within main mass (forming 50 % of tumor volume)
-Extending away from main mass
3. Excisional biopsy margins: Free of tumor. Margins greater than 2mm away.
4. Blood vessel and lymphatic invasion: Absent
5. Axillary lymph nodes: Negative for tumor (0/2)
6. Special studies
-No expression of ER in invasive tumor nuclei
-No expression of PR in invasive tumor nuclei
-Her2/neu antigen (FISH): Pending
7. pTNM: pT2,N0,MX

Clinical History:
This is a year-old female with history of a right papilloma. A screening mammogram in showed suspicious densities with calcifications in the left outer quadrant. By exam showed a 1.5 cm quasi suspicious mass at 4-5:00, 3 cm outside the areola margin on the left breast. , she had a FNA of the left breast showing carcinoma. On ı breast MRI on the left showed an outer quadrant 4.7 x 3.2 x 3.3 cm enhanced mass with three additional suspicious foci: one anterior and superior to the index mass, one adjacent to the nipple, and one suspicious focus at 12 o'clock. The patient undergoes a left breast sentinel node biopsy and simple mastectomy.

Specimens Received:
A: Sentinel Node #1
B: Sentinel Node #2
C: Left Breast Mastectomy with Sentinel Node Biopsies

Gross Description:
The specimens are received in three containers each labeled with the patient' s name and medical record number.

A. Container A is further designated 31. sentinel node #14. Received for frozen section diagnosis is a 1.8 x 1.2 x 0.5 cm rubbery portion of yellow, lobulated tissue with blue discoloration. It is read as 3one lymph node, negative for carcinoma4 by The specimen is entirely submitted as A1FS.

B. Container B is further designated 32. sentinel node #24. Received for frozen section diagnosis is a 1.5 x 0.8 x 0.8 cm portion of pink-tan rubbery tissue consistent with a lymph node. It is bisected and read as 3one lymph node, negative for carcinoma4 by The specimen is entirely submitted in cassette B1FS in a mesh bag.

C. Container C is further designated 33. left breast mastectomy with sentinel node biopsies4. Received fresh and placed in formalin is a 975 gm, 39 x 18 x 6 cm mastectomy specimen with an ellipse of white-tan skin with blue discoloration (27 x 11.2 cm), areola (4.7 x 4.5 cm), and nipple (1.2 x 1.0 x0.4 cm). The specimen is oriented as follows: short suture superior, long suture lateral. The deep margin is inked black and the specimen is serially sectioned into 13

slices from lateral (slice #1) to medial revealing a 5 x 5.5 x 4 cm ecchymotic area in the lower lateral quadrant located in slices #4-7. There is a well-circumscribed, firm, white mass measuring 2.5 x 1.7 x 1.5 cm in slice #5, 5.5 cm from the deep margin. It is located inferior to the ecchymotic region. At 2-3 o'clock adjacent to the firm mass is an ill-defined, gritty, indurated area primarily in slices #7 and #8. It extends superomedially with a span of 5.0 x 3.5 x 2.0 cm and is located 2.5 cm from the deep margin. Note: slices #7 and #8 contain the nipple and areola region.

Cassette SummaryC1: nipple
C2-C4: areola, serially sectioned
C5: section of the main mass in slice #5 with respect to closest distant from deep margin
C6-C7: one representative section immediately superior and anterior to the main lesion from slice #5
C8: section of hematoma from slice #5 anterior and medial to the main lesion
C9: section of hematoma from slice #6, directly medial to the main lesion
C10: section 2 cm superior, anterior, and medial to the main lesion from slice #7
C11: representative sections from slice #8, representing ill-defined, gritty, firm tissue
C12-C13: section from slice #7 also representing ill-defined, gritty, firm tissue
C14: another section from slice #7 immediately inferior to the section C12-C13
C15-C16: section from 12 o'clock on section #8
C17-C18: sections from slice #9
C19: another representative section from section #9
C20-C21: lateral upper quadrant from slices #3-4, respectively
C22-C23: lateral lower quadrant from slices #2 and #3, respectively
C24-C25: upper medial quadrant from slice #11 and #12, respectively C26-C27: medial lower quadrant from slices #11 and #12, respectively

Intraoperative Consult Diagnosis:
A1FS. Sentinel node #1: One lymph node, negative for carcinoma.
B1FS. Sentinel node #2: One lymph node, negative for carcinoma.

Criteria 12/20/12	Yes	No
Diagnosis Discrepancy		✓
Primary Tumor Site Discrepancy		✓
HIPAA Discrepancy		✓
Prior Malignancy History		✓
Dual/Synchronous Primary Noted		✓
Case is (circle): QUALIFIED / DISQUALIFIED		
Reviewer Initials [illegible] Date Reviewed 12/21/12		

UUID:A8980EE8-52DE-4DA5-84DD-F8E86EBF6A9B
TCGA-OL-A5D7-01A-PR Redacted

Sample #

ICD-O-3
Carcinoma, Infiltrating Duct NOS 8500/3
Site: (L) Breast, NOS C50.9
JW 12/24/12

Surgical Pathology Report
* Amended *

FINAL PATHOLOGIC DIAGNOSIS
Lumpectomy and sentinel lymph node biopsy, left breast and axilla:
A. Sentinel lymph node #1, left axilla:
- Single lymph node, no tumor (0/1).
B. Left breast lumpectomy:
- Invasive ductal carcinoma, SBR grade III, with focal necrosis and associated lymphocytic infiltrate. See parameters below.
- Ductal carcinoma in situ, solid type, high nuclear grade, associated with invasive carcinoma.
- Unremarkable uninvolved breast parenchyma.

Breast Pathologic Parameters

1. Invasive carcinoma:
A. Gross measurement: 2.8cm
B. Composite histologic (modified SBR) grade III
- Architecture: 3
- Nuclear grade: 3
- Mitotic count: 3
C. Associated ductal carcinoma in situ (DCIS): high nuclear grade, <5% of tumor volume,
associated with invasive carcinoma.
2. Excisional biopsy margins: Free of tumor
- DCIS >1 cm from nearest margin.
- Invasive carcinoma 7mm from posterior (closest) margin
3. Blood vessel and lymphatic invasion: Absent
4. Axillary lymph nodes: Negative for tumor
5. Special studies (performed on current material):
- No reactivity for ER (no staining).
- No reactivity for PR (no staining).
- Her2/neu (FISH) is pending.
6. pTNM: pT2, N0(sn), MX.

Clinical History:
year-old female with a left breast cancer undergoing left breast sentinel lymph node biopsy and partial mastectomy.

Specimens Received:
A: Left axillary sentinel lymph node #1
B: Left breast partial mastectomy

Gross Description:
Specimens received in two containers each labeled with the patient' s name and medical record number.

A. Part A is additionally designated 'left axillary sentinel lymph node #1.' Received fresh for frozen diagnosis is a 2.4 x 1.3 x 0.9 cm lymph node which is bisected. Half of the lymph node is frozen and read as no carcinoma per Dr. Chang. The remnant of frozen tissue is entirely submitted in cassettes A1FS. The remnant of non-frozen tissue is submitted in cassette A2.

Part B additionally designated '2 left breast partial mastectomy double short stitch superior, double long lateral'. Received fresh and placed in formalin is a 114 gm partial mastectomy specimen. The measurements are as follows: Superior to inferior 7.0 cm, medial to lateral 9.8 cm, and anterior to posterior 4 cm. There is skeletal muscle on the posterior aspect of the lumpectomy specimen measuring 3.8 x 1.5 cm. The specimen is inked as follows: Superior blue, inferior green, posterior red, anterior black, lateral purple and medial yellow. The specimen is serially sectioned from medial to lateral and to 11 slices. There is a 2.8 x 2.5 x 2.4 cm well-circumscribed firm tan-pink mass which demonstrates focal hemorrhage. This mass is 1.1 cm from the inferior margin, 0.7 cm from the posterior margin, 3.5 cm from the superior margin, 1.1 cm from the anterior margin, 2.2 cm from the lateral margin and greater than 5 cm from the medial margin.

B1-B2: medial margin.
B3-B7 lateral margin. Clearly sectioned and entirely submitted.
B8-B10 : representative sections of mass from slice 7 demonstrating relationship to red ink margin (posterior)
B11: representative site of section of mass in slice 8 showing relationship to green margin.
B12: representative section of slice 8 showing skeletal muscle on posterior aspect
B13-B15: representative sections of mass in slice 9 showing relationship to red, green, and black inked margins.
B16: representative section of slice 9 demonstratingskeletal muscle at closest approximation to mass.
B17: representative section of normal breast parenchyma, site unspecified

B18: normal breast parenchyma slice 3.
B19: normal breast parenchyma slice 7.

Intraoperative Consult Diagnosis:
A1FS. Left axillary sentinel lymph node #1: 'No carcinoma'

Criteria	Yes	No
Diagnosis Discrepancy		✓
Primary Tumor Site Discrepancy		✓
HIPAA Discrepancy		✓
Prior Malignancy History		✓
Dual/Synchronous Primary Noted		✓
Case is (circle): QUALIFIED / DISQUALIFIED		
Reviewer Initials: KMI Date Reviewed: 12/20/12		

lw 12/20/12

UUID:6A981CB0-F551-48B1-AB91-5D5585A7F282
TCGA-S3-AA10-01A-PR Redacted

Surgical Pathology Final Report
Temporary Copy

Case: [redacted]
Collected:
Ordered by:
Patient:
ID: [redacted]
Location:

Diagnosis

A. RIGHT BREAST, MASTECTOMY:
- **HIGH-GRADE INVASIVE DUCTAL CARCINOMA; SEE SYNOPTIC REPORT**
- **NEGATIVE MARGINS**
- **SKIN AND NIPPLE WITHOUT SIGNIFICANT PATHOLOGIC ABNORMALITY**
- **FOUR LYMPH NODES, NEGATIVE FOR TUMOR (0/4); SEE SPECIAL STAINS**

ICD-O-3
Carcinoma, infiltrating duct NOS 8500/3
Site (R) Breast NOS C50.9
(R) Breast, upper-outer quadrant C50.4
[illegible] 2/24/14

(Electronic signature)
Verified:

Synoptic Report

TUMOR SIZE: SIZE OF LARGEST INVASIVE CARCINOMA:
  Greatest dimension of largest focus of invasion over 0.1 cm: 2.5 cm
    Additional dimensions: 2.5 x 1.5 cm
TUMOR FOCALITY:
  Single focus of invasive carcinoma
MACROSCOPIC AND MICROSCOPIC EXTENT OF TUMOR:
  Skin: Invasive carcinoma does not invade into the dermis or epidermis
  Nipple: DCIS does not involve the nipple epidermis
  Skeletal Muscle: Skeletal muscle is present and is free of carcinoma
DUCTAL CARCINOMA IN SITU (DCIS):
  No DCIS is present
LOBULAR CARCINOMA IN SITU (LCIS):
  Not identified
HISTOLOGIC TYPE OF INVASIVE CARCINOMA:
  Invasive ductal carcinoma (no special type or not otherwise specified)
GLANDULAR (ACINAR)/TUBULAR DIFFERENTIATION:
  Score 3: <10% of tumor area forming glandular/tubular structures
NUCLEAR PLEOMORPHISM:
  Score 3: Vesicular nuclei, often with prominent nucleoli, exhibiting marked variation in size and shape, occasionally with very large and bizarre forms
MITOTIC COUNT:
  Score 3
    Number of mitoses per 10 high-power fields: 88
    Diameter of microscope field: 0.55 mm
OVERALL GRADE:
  Grade 3: scores of 8 or 9
MARGINS:
  Margins uninvolved by invasive carcinoma
    Distance from anterior margin: 4.0 mm
    Distance from posterior margin: 2.0 mm
TREATMENT EFFECT: RESPONSE TO PRESURGICAL THERAPY: IN THE BREAST:
  No known presurgical therapy
TREATMENT EFFECT: RESPONSE TO PRESURGICAL THERAPY: IN THE LYMPH NODES:
  No known presurgical therapy
LYMPH-VASCULAR INVASION:
  Not identified

Surgical Pathology Final Report
Temporary Copy
Case: [redacted] Patient: [redacted]
Collected: ID: [redacted]
Ordered by: Location:

DERMAL LYMPH-VASCULAR INVASION:
Not identified
LYMPH NODES:
Total number of lymph nodes examined (sentinel and nonsentinel): 4
Number of lymph nodes with macrometastases (>0.2 cm): 0
Number of lymph nodes with micrometastases (>0.2 mm to 0.2 cm and/or >200 cells): 0
METHOD OF EVALUATION OF SENTINAL LYMPH NODES:
Hematoxylin and eosin (H&E), one level
Immunohistochemistry
PRIMARY TUMOR (INVASIVE CARCINOMA (pT):
pT2: Tumor >20 mm but less than or equal to 50 mm in greatest dimension
REGIONAL LYMPH NODES (pN):
pN0: No regional lymph node metastasis identified histologically
DISTANT METASTASIS (M):
Not applicable
ADDITIONAL PATHOLOGIC FINDINGS:
Benign breast with calcifications
ESTROGEN RECEPTOR:
Performed on this specimen
No immunoreactive tumor cells present
PROGESTERONE RECEPTOR:
Performed on this specimen
No immunoreactive tumor cells present
HER2/NEU IMMUNOPEROXIDASE STUDIES:
Performed on this specimen
Negative (Score 0)
MICROCALCIFICATIONS:
Present in non-neoplastic tissue

Specimen Source

A RT Breast and Axillary Contents

Clinical Information

None
PRE-OP DIAGNOSIS: Right breast CA
POST-OP DIAGNOSIS: Same
TYPE OF PROCEDURE: Right modified mastectomy

Gross Description

The specimen is labeled "RIGHT BREAST AND AXILLARY CONTENTS" and is received unfixed (the specimen is in formalin for more than 6 hours and less than 48 hours). It consists of a 420 g right mastectomy with axillary contents weighing 420 g measuring 18 x 10 x 3.0 cm. The axillary content measures 6.0 x 5.5 x 3.0 cm. The nipple is unremarkable measures 1.3 x 1.3 cm in maximum dimensions. Solar is unremarkable. The left fascia is smooth and glistening. The anterior margin is inked red and the deep posterior margin is inked black. On sectioning, there is a well-circumscribed lobulated focally hemorrhagic pink-tan mass measuring 2.5 x 2.5 x 1.5 cm. The mass is close to the deep posterior margin in the outer quadrant close to the axillary contents. The mass is 0.7 cm from the anterior margin and approximately 12 cm from the nipple. On further sectioning the breast parenchyma is nodular, there are no other lesions grossly identified. There are four fatty lymph nodes

ranging from 0.5-2.0 cm in maximum dimensions. Representative sections are submitted.

Section Key:

A1 - A2 tumor markers with deep margin
A3 - tumor
A4 - nipple and skin
A5 - anterior margin close to mass
A6 - upper inner quadrant
A7 - lower inner quadrant
A8 - random sections from central breast
A9 - upper outer quadrant
A10 - lower outer quadrant
A11 - one lymph node bisected
A12 - one lymph node bisected
A13 - two lymph nodes

Time specimen was removed from the patient (procedure time):
Time specimen was placed in formalin:
Ischemic time: 1 hour 20 minutes

Dictated by:

Special Stains / Slides

IMMUNOHISTOCHEMICAL EVALUATION OF ESTROGEN RECEPTORS, PROGESTERONE RECEPTORS, AND HER-2NEU IN INVASIVE MAMMARY CARCINOMA .

ESTROGEN RECEPTORS: 0 %, NEGATIVE.
PROGESTERONE RECEPTORS: 0 %, NEGATIVE.
STAINING INTENSITY: _

HER-2NEU: SCORE 0, NEGATIVE.

Immunohistochemical studies were performed on formalin fixed paraffin embedded tissue (Block A2) using the following monoclonal antibodies: Estrogen receptor (Clone SP1), Progesterone receptor (Clone 1E2) and Her-2neu (Clone 4B5); control sections for HER-2Neu are provided within a kit (score 0 MCF-7, score 1+ T-47D, score 2+ MDA-MB-453, score 3+ BT-474). Detection system used: polymer. Primary antibodies, reagents and control sections for HER-2neu are all provided by

All controls show appropriate reactivity.

Reactivity of Estrogen and Progesterone receptors is determined based on the percentage of positively stained nuclei of tumor cells. Reference values (CAP accreditation program checklist 2010 and guidelines on webpage):
Positive: nuclear staining in 1% or greater than 1% of invasive carcinoma cells
Negative : nuclear staining in less than 1% of invasive carcinoma cells
Staining intensity: is reported as weak, moderate or strong.

Surgical Pathology Final Report
Temporary Copy

Case: [redacted] Patient: [redacted]
Collected: ID: [redacted]
Ordered by: Location:

<u>HER-2neu reactivity</u> is reported applying the CAP scoring guidelines (CAP accreditation program checklist 2010 and guidelines on webpage):
Score 0 = **Negative**: No immunoreactivity, or faint weak immunoreactivity in <10% of tumor cells but only a portion of the membrane is positive..
Score 1 = **Negative**: Faint weak immunoreactivity in 10% or >10% of tumor cells but only a portion of the membrane is positive.
Score 2+ = **Equivocal**: Weak to moderate complete membrane immunoreactivity in >10% of tumor cells or circumferential intense membrane staining in <30% of cells.
Score 3+ = **Positive**: More than 30% of the tumor cells must show circumferential intense and uniform membrane staining. A homogeneous (chicken wire) pattern should be present.

Equivocal results for HER-2neu (Score 2+) will be subsequently followed by a reflex dual-color ISH testing.

Additional immunohistochemical studies fpr AE1/AE3 were performed on formalin fixed, paraffin-embedded tissue (Blocks A11-13) with adequate positive and negative control sections. All stains were negative.

The performance characteristics of these antibodies were determined by the . They have not been cleared or approved by the U.S. Food and Drug Administration. The FDA has determined that such clearance or approval is not necessary. These tests are used for clinical purposes. They should not be regarded as investigational or for research. This laboratory is certified under the Clinical Laboratory Improvement Amendments of 1988 (CLIA-88) as qualified to perform high-complexity clinical laboratory testing.

Tissue Code

Criteria lw 12/11/13	Yes	No
Diagnosis Discrepancy		✓
Primary Tumor Site Discrepancy		✓
HIPAA Discrepancy		✓
Prior Malignancy History		✓
Dual/Synchronous Primary Noted		✓
Case is (circle): QUALIFIED (circled) / DISQUALIFIED		
Reviewer Initials BTH Date Reviewed: 12/11/2013		

UUID:FDB00212-9782-4ACE-AD59-019384297A2F
TCGA-WT-AB41-01A-PR Redacted

SURGICAL PATHOLOGY REPORT

NAME:		SURG PATH #:	
MR #:		SPECIMEN CLASS:	
BILLING #:		ALT ID #:	
LOCATION:		DATE OF PROCEDURE:	
AGE:	SEX: F	DATE RECEIVED:	
DOB:		TIME RECEIVED:	
PHYSICIAN:		DATE OF REPORT:	
COPY TO:		DATE OF PRINTING:	

ICD-O-3
Carcinoma, infiltrating duct NOS 8500/3
Site (L) Breast NOS C50.9
JLC 6/30/14

Material Received:
A: left axillary sentinel lymph node #1 blue
B: left axillary sentinel lymph node #2 blue
C: left breast total mastectomy
D: 8 o'clock additional medial short superior , long anterior, clip true margin
E: 8 o'clock additional inferior short anterior, long lateral clip true margin
F: 8 o'clock additional anterior short superior, long lateral, clip true margin
G: 8 o'clock additional posterior short superior, long lateral, clip true margin
H: additional skin left breast blue at 3:00

History:
year old female with a clinical history of breast cancer.

Final Diagnosis:

A. Lymph node, "left axillary sentinel lymph node #1 blue", biopsy:
Micrometastatic mammary carcinoma (0.23 mm) identified on permanent and pancytokeratin immunostained sections only. See comment.

B. Lymph node, "left axillary sentinel lymph node #2 blue", biopsy:
There is no evidence of malignancy (0/1).
Deeper sections and a pancytokeratin immunostain are negative in support of the above diagnosis.

C. Breast, "left breast total mastectomy", mastectomy:
Invasive ductal carcinoma, moderately differentiated, nuclear grade 2, two foci (measuring 1.2 cm and 1.8 cm in greatest dimension respectively). See comment.

D. Fibroadipose tissue, "8 o'clock additional medial short superior , long anterior, clip true margin", reresection:
No diagnostic abnormalities.
There is no evidence of malignancy.

E. Fibroadipose tissue, "8 o'clock additional inferior short anterior, long lateral clip true margin", reresection:
No diagnostic abnormalities.
There is no evidence of malignancy.

F. Fibroadipose tissue, "8 o'clock additional anterior short superior, long lateral, clip true margin", reresection:
No diagnostic abnormalities.
There is no evidence of malignancy.

G. Skeletal muscle and adipose tissue, "8 o'clock additional posterior short superior, long lateral, clip true margin", reresection:

No diagnostic abnormalities.
There is no evidence of malignancy.

H. Skin, "additional skin left breast blue at 3:00", reresection:
No diagnostic abnormalities.
There is no evidence of malignancy.

Comment:

INVASIVE CARCINOMA OF THE BREAST
Specimen Type: mastectomy
Laterality: left
Tumor Site: lower inner quadrant at 8:00 and mid outer quadrant at 3:00
Histologic Type: Invasive ductal carcinoma
Size of Invasive Component: 1.2 cm in greatest dimension (8:00) and 1.8 cm in greatest dimension (3:00)
Tumor Multicentricity: Present (2 foci as noted above)
Surgical Margins: Final margins are negative for carcinoma. In the mastectomy, the 8:00 focus is within 0.1 cm of the inferior, medial and anterior margins and within 0.2 cm of the deep margin; however the separately submitted additional medial, inferior, anterior and posterior margins (specimens D through G) are negative for carcinoma.
Histologic Grade (Nottingham Histologic Score): II/III
Tubule Formation: 3
Nuclear Grade: 2
Mitotic Count (40x objective): 1
Total Nottingham Score: 6/9
Ductal Carcinoma In-situ (DCIS): Present, focal, intermediate nuclear grade, without necrosis
Lobular Carcinoma In-situ (LCIS): Absent
Lymph-Vascular Invasion: Present, focal
Perineural Invasion: not identified
Tumor Necrosis: not identified
Nipple Involvement: not identified
Skin Involvement: not identified
Lymph Node Sampling:
Sentinel lymph node(s) only
Total number of involved nodes/total nodes found: 1 micrometastasis out of 2 nodes.
Size of largest metastasis: 0.23 mm
Extranodal extension: not identified
Fixation to one another:
Non-neoplastic Breast Tissue: not identified
Treatment Effect (Response to Presurgical Neoadjuvant Therapy):
In the Breast:
In the Lymph Nodes:
Prognostic markers: See for addendum on prior biopsy
Fixation Time between 6-48 hours: Yes
Pathologic Staging:
pT2N(sn) 1miM(not applicable)

Primary Tumor (Invasive Carcinoma) (pT)
pT1: Tumor ≤20 mm in greatest dimension
pT1c: Tumor >10 mm but ≤20 mm in greatest dimension

Regional Lymph Nodes (pN)
(sn): Only sentinel node(s) evaluated. If 6 or more sentinel nodes and/or nonsentinel nodes are removed, this modifier should not be used.
pN1mi: Micrometastases (greater than 0.2 mm and/or more than 200 cells, but none greater than 2.0 mm).

Distant Metastasis (M)
Not applicable

NAME: SURG PATH #:
MR #: ALT ID #:

The pathologic stage assigned here should be regarded as provisional, as it reflects only current pathologic data and does not incorporate full knowledge of the patient's clinical status and/or prior pathology.

The final diagnosis has been communicated via electronic mail to by

Attestation:
By this signature, I attest that I have personally formulated the final interpretation expressed in this report and that the above diagnosis is based upon my examination of the slides and/or other material indicated in this report.
Electronically Signed Out By
Interpreted by:

Gross Description:
A. Received fresh labeled with the patient's name and "left axillary sentinel lymph node #1 blue" is an irregular portion of red-tan fibroadipose tissue. The tissue is palpated to reveal one possible lymph node measuring 2.8 x 2.5 x 1.3 cm. The specimen is serially sectioned and submitted entirely in cassettes A1FS and A2FS for frozen and permanent diagnosis.

B. Received fresh labeled with the patient's name and "left axillary sentinel lymph node #2 blue" is an irregular portion of red-tan fibroadipose tissue. The tissue is palpated to reveal one possible lymph node measuring 2.5 x 1.8 x 1.0 cm. The possible lymph node is serially sectioned and submitted entirely in cassette B1FS for frozen and permanent diagnosis.

C. Received fresh labeled with the patient's name and "left breast total mastectomy short superior/long lateral" is a 650 gram simple mastectomy specimen measuring 20.5 x 18 x 3.5 cm. There is a short stitch marking superior and a long stitch marking lateral. The breast is serially sectioned to reveal a 1.2 x 1.2 x 0.8 cm tan-pink stellate mass that is 22.5 cm from the lateral margin, abutting the medial margin, 8.4 cm from the superior margin, abutting the inferior margin, 0.2 cm from the superficial margin, and 0.2 cm from the deep margin. The mass is located in the lower inner quadrant at approximately 8:00. The deep resection margin is inked black and the superficial resection margin is inked blue. The medial margin of the mass is inked orange and the inferior margin of the mass is inked green. A second tan-white stellate mass measuring 1.8 x 1.5 x 1.4 cm is located 6.5 cm from the lateral margin, 13.6 cm from the medial margin, 13.6 cm from the superficial margin, 8.3 cm from the inferior margin, 2.4 cm from the superficial margin/skin and 3.7 cm from the deep margin. The mass is located in the mid outer portion of the specimen at approximately 3:00. The remainder of the breast parenchyma is yellow-white and unremarkable. The specimen contains a tan-brown skin ellipse measuring 7.2 x 4.5 cm. The nipple measures 1.4 x 1.3 x 0.6 cm. There is no dimpling or skin retraction identified. The breast is placed in formalin at . The specimen is submitted as follows:

C1 Lower inner quadrant mass to medial margin.
C2-C4 Remainder of lower inner quadrant mass (to superficial, deep and inferior margin).
C5-C6 Second mass to deep margin (bisected).
C7-C8 Second mass to superficial margin/skin (bisected).
C9-C14 Remainder of second mass.
C15 Representative section from upper inner quadrant.
C16 Representative section from lower inner quadrant.
C17 Representative section from lower outer quadrant.
C18 Representative section from upper outer quadrant.
C19 Nipple (serially sectioned).
C20 Skin.

D. Received in formalin labeled with the patient's name and "8:00 additional medial, short superior, long lateral, clip true" is a 2.6 x 1.9 x 1.0 cm lumpectomy specimen, with a short suture designating superior, a long stitch designating anterior, and clips designating the true (medial) margin. The specimen is received as follows:
Anterior - orange
Posterior - black
True (medial) - green
Lateral - blue

The specimen is serially sectioned from superior to inferior to reveal a tan-yellow, glistening cut surface. No discrete lesions are identified. The specimen is submitted entirely from superior to inferior in cassettes D1 through D4. The breast was placed in formalin at

E. Received in formalin labeled with the patient's name and "8:00 additional inferior short anterior, long lateral, clip true margin" is a 3.0 x 2.3 x 1.1 cm lumpectomy specimen, with short suture designating anterior, long suture designating lateral, and clips marking the true margin. The specimen is inked as follows:
Anterior - orange
Posterior - black
Superior - blue
True (inferior) - green

The specimen is serially sectioned from medial to lateral to reveal a yellow-gold, glistening cut surface. No discrete lesions are identified. The specimen is submitted entirely from medial to lateral in cassette E1 through E4. The breast is placed in formalin at

F. Received in formalin labeled with the patient's name and "8:00 additional anterior short superior, long lateral, clip true margin" is a 3.2 x 1.7 x 0.3 cm lumpectomy specimen, with short sutures designating superior, a long stitch designating lateral, and clips designating the true (inferior) margin. The specimen is inked as follows:
True (anterior) - orange
Posterior - black
Medial - green
Lateral - blue

The specimen is serially sectioned from superior to inferior to reveal a yellow, glistening cut surface. No discrete lesions are identified. The specimen is submitted entirely from superior to inferior in cassettes F1 through F3. The breast is placed in formalin at

G. Received in formalin labeled with the patient's name and "8:00 additional posterior short superior, long lateral, clip at true margin" is a 2.1 x 1.9 x 0.6 cm lumpectomy specimen with short sutures designating superior, a long set of sutures designating lateral, and clips designating the true margin. The specimen is inked as follows:
Anterior - orange
True (posterior) - black
Medial - green
Lateral - blue

The specimen is serially sectioned from superior to inferior to reveal a tan-pink to tan-brown cut surface. No discrete lesions are grossly identified. The specimen is submitted entirely from superior to inferior in cassettes G1 through G3. The specimen is placed in formalin at

H. Received in formalin labeled with the patient's name and "additional skin left breast blue at 3:00" is a 5.0 x 5.0 cm C-shaped portion of tan-brown skin and subcutaneous tissue with an attached strip of brown skin measuring 13.9 x 0.3 x 0.5 cm. The skin on the specimen was inked blue in the OR and designates 3:00. The remainder of the specimen is inked as follows:
12 to 3:00 - orange
3 to 6:00 - purple
6 to 9:00 - blue
9 to 12:00 – green
Deep-black

The specimen is serially sectioned from 12 to 6:00, and no discrete lesions are identified. Also received within the container are two irregular portions of tan-brown skin and subcutaneous tissue measuring 1.4 x 0.6 x 0.6 cm and 8.6 x 0.3 x 0.5 cm. This tissue is serially sectioned and no discrete lesions are identified. The specimen is submitted as follows:

H1 -	Representative sections from the 12 to 3:00 margin
H2-H4 -	Representative sections from the 3 to 6:00 margin
H5 -	Representative sections from the 6 to 9:00 margin
H6 -	Representative sections from the 9 to 12:00 margin
H7-H8 -	Representative sections from the additional skin and underlying soft tissue

Intraoperative Consultation:
A1FS, A2FS, lymph node, "left axillary sentinel lymph node #1 blue":
Negative for carcinoma.

NAME:	SURG PATH #:
MR #:	ALT ID #:

B1FS, lymph node, "left axillary sentinel lymph node #2 blue":
Negative for carcinoma.

If immunohistochemical stains and/or in situ hybridization are cited in this report, the performance characteristics were determined by the in compliance with CLIA'88 regulations. Some of these tests rely on the use of "analyte specific reagents" and are subject to specific labeling requirements by the FDA. Known positive and negative control tissues demonstrate appropriate staining. This testing was developed by the It has not been cleared or approved by the FDA. The FDA has determined that such clearance or approval is not necessary.

Criteria lw 1/24/14	Yes	No
Diagnosis Discrepancy		✓
Primary Tumor Site Discrepancy		✓
HIPAA Discrepancy		✓
Prior Malignancy History		✓
Dual/Synchronous Primary Noted		✓
Case is (circle): QUALIFIED / DISQUALIFIED		
Reviewer Initials [illegible] Date Reviewed: 1/23/14		
