# Supplementary material for: Comparative Evaluation of Breast Ductal Carcinoma Grading: A Deep-Learning Model and General Pathologists’ Assessment Approach
Source: Diagnostics (Basel). 2023 Jul 10;13(14):2326. doi: 10.3390/diagnostics13142326 (PMC10377791; doi:10.3390/diagnostics13142326)
Supplement: Supplementary file 1 [file diagnostics-13-02326-s001.zip › S3 - NGS grading by the HA.pdf]

PATHOLOGIST 1

UUID	SLIDE ID	Tubule formation	Nuclear pleomorphism	Mitotic count	Total score	Grade
d2c7ad27-6090-421e-a12e-2cb66a5417e4	TCGA-E2-A1LK-01Z-00-DX1.5EBAA1F4-F1B4-4938-A51F-0246621BB0ED	3	3	2	8	3
b1e64f03-c419-421e-b801-2439bace5ad5	TCGA-AO-A0JB-01Z-00-DX1.250FE098-345B-4981-9236-0519E1C9058E	3	2	2	7	2
a7ccf35e-56a9-4bd9-904c-59b1b0cc33b4	TCGA-E2-A1L6-01Z-00-DX1.AFE87067-2BFD-42C2-9334-9DDE8AB61B49	1	2	1	4	1
d9ab7666-6cdd-4cb4-89fd-e7916e10986a	TCGA-A1-A0SN-01Z-00-DX1.5E9B85AE-AFB7-41DC-8A1B-BD6DA39B6540	2	3	2	7	2
469579d1-f578-4956-aab0-9e7728a88ff7	TCGA-A7-A13D-01Z-00-DX1.D206783C-FA6A-4B6A-B3AA-4132A2C9626B	3	3	2	8	3
454d3eb9-ff96-4cd4-9dec-17252819ed13	TCGA-B6-A0WW-01Z-00-DX1.64048633-1D1A-4074-8C50-7641159355DB	2	2	2	6	2
9b91d3c8-c250-4f74-9ff7-bb8d11f813a7	TCGA-D8-A1XL-01Z-00-DX1.FDF07020-8F40-4C00-9023-E5F40E0D8A7C	3	2	2	7	2
1f4d56a0-b948-4a65-8b3a-f66d30317e0b	TCGA-D8-A13Y-01Z-00-DX2.0043D2BB-B04D-4A44-AAB1-1CAAD97AC246	3	3	3	9	3
b658e65c-636f-43b9-8c52-a46ba2850044	TCGA-A2-A0SU-01Z-00-DX1.22420EE2-4FEB-42F3-9547-4739F0F73D50	3	2	3	8	3
f6f7b32b-33e4-462a-9f83-2a3f74a6302a	TCGA-E2-A108-01Z-00-DX1.B110ED43-08A4-476A-A658-1CA75F7C0DDE	2	2	2	6	2
55eb1c6a-2290-4458-afa2-f259d80e6395	TCGA-LL-A5YN-01Z-00-DX1.F221939B-3680-4B95-93A9-BE8599550E87	2	2	3	7	2
d29fb01b-dfb0-49fe-b42c-98dd32a306c1	TCGA-OL-A5D7-01Z-00-DX1.A4A45393-9AE1-4370-8B92-CB85CDB04934	3	2	3	8	3
40b5da84-5b98-4444-9745-2f1ebe5132e4	TCGA-WT-AB41-01Z-00-DX1.75BDFDF2-CD87-46D1-B32C-725741CB02BE	3	2	2	7	2
a18c60c5-3053-477a-b892-305bf4e5bfe7	TCGA-D8-A27R-01Z-00-DX1.F6E2FD1C-0666-4788-8D95-A76D15907270	3	3	2	8	3
fcb893aa-c7aa-438d-9d6d-c0adb397fa1e	TCGA-E2-A9RU-01Z-00-DX1.A06BE284-B9DC-4B45-A202-A9D027AEEDD9	3	3	2	8	3
68458986-1efd-4fce-9e65-ce5430ab125c	TCGA-3C-AALJ-01Z-00-DX1.777C0957-255A-42F0-9EEB-A3606BCF0C96	2	2	2	6	2
069a8f13-04e2-41c7-ab64-90a465c7c4bc	TCGA-AR-A0TQ-01Z-00-DX1.2BEA298C-6B3D-4133-ADBC-769E62CEFFA0	2	3	2	7	2
6f29dfa9-d166-4f07-9cdb-0daceb7341e9	TCGA-D8-A27R-01Z-00-DX2.31F47D8F-DFD7-42AE-BBBA-7DBBA12FA97D	3	3	2	8	3
0dcc8304-dfd1-4e79-b23f-30f1143c897c	TCGA-E2-A14N-01Z-00-DX1.15F5644F-CA9F-4688-B56E-BCC00CA4769B	2	3	3	8	3
63858a48-c99e-4080-97f0-419f662ef195	TCGA-D8-A1XD-01Z-00-DX1.E500D561-1F49-4F08-99AE-E8345F21B406	2	2	2	6	2
0279f555-9738-41e0-b89f-ded25829adc0	TCGA-E2-A1L9-01Z-00-DX1.F2CC1036-8EEE-4664-962E-541B8ACB10DE	2	2	2	6	2
7b3d2ef5-0b8b-4b3e-8210-c77f8302e437	TCGA-E2-A15C-01Z-00-DX1.26E13415-1D37-43C7-9EBB-4411BE7FCE10	2	2	2	6	2
bcd6977f-b269-48c4-b088-ddf8d6d95240	TCGA-GM-A2DF-01Z-00-DX1.CD0BE6D7-2DB3-4193-84CC-F9BE7BF18CC2	1	3	2	6	2
4fa9d2cb-379e-4183-b5c6-a8ba2a15ee57	TCGA-AR-A0U1-01Z-00-DX1.276433E7-E841-42D2-AF21-762F2FEA3B9B	1	3	3	7	2
33f7413f-6339-4389-98dd-eee280233684	TCGA-E2-A1LH-01Z-00-DX1.F85384B7-1EBF-4F57-A45B-4A668B68E535	1	3	2	6	2
9db0bfe8-9f66-4416-ab90-e0b1f91d2e01	TCGA-D8-A1XR-01Z-00-DX2.A103FB8B-4397-4DD4-8587-90A736407484	1	3	3	7	2
f554759b-e293-45f5-be85-34a561e36027	TCGA-AR-A0U2-01Z-00-DX1.03E2ADD9-F20F-44DA-93D1-D10BBD3844A5	3	3	3	9	3
75f48929-04a3-4970-8572-29b1068b2d85	TCGA-D8-A147-01Z-00-DX1.159094F2-BB78-4910-B7AE-3D7CAAB1DAD9	2	3	3	8	3
45b31b73-adeb-4793-837f-8a9f957cffad	TCGA-A2-A3XY-01Z-00-DX1.E57FC9BF-411E-4028-AC10-8BCA5D0C8472	3	3	2	8	3
e47bd352-9d96-4abd-b673-9d30038d218a	TCGA-B6-A0RI-01Z-00-DX1.E39951A4-AC6A-4B07-851B-F35CB86D79AA	2	2	2	6	2
54d20f52-a7b1-4814-ba36-082108f23b59	TCGA-E9-A22A-01Z-00-DX1.d986c9eb-2c54-4663-a54b-04c0756db6db	3	3	2	8	3
df1645c7-0db5-4262-8c8d-b8577f272914	TCGA-A2-A0YJ-01Z-00-DX1.8135C74E-DAA8-4C8E-AF14-A4B5B57695BE	3	3	2	8	3
26eb27eb-84c8-4fea-a51b-c3eb5c484b33	TCGA-E2-A1LI-01Z-00-DX1.503dd2fa-23ef-4b11-8aab-301e069eaa88	3	3	3	9	3
e5069ea5-1011-46cf-9a3c-925825eeb81e	TCGA-A2-A0T5-01Z-00-DX1.128C288B-B357-439B-A8D4-8E7DEBF73E4E	1	3	2	6	2
e15f67af-fb62-42c8-a451-83b035867106	TCGA-D8-A1JD-01Z-00-DX1.6D215B14-DD90-4635-8645-AF06EBD9BA3F	2	3	2	7	2
f273a858-1cfa-4983-ace4-b1e876d64473	TCGA-A1-A0SF-01Z-00-DX1.7F252D89-EA78-419F-A969-1B7313D77499	2	2	2	6	2
9b8dec44-e674-4dc7-a8a8-c962c907b64b	TCGA-D8-A1JC-01Z-00-DX2.854ABF5D-40F1-48AE-802F-97D75497F1FD	2	3	2	7	2
206ccd79-310d-40a4-852c-03930e685304	TCGA-EW-A6SB-01Z-00-DX1.D56E1922-01A9-4AEE-AB95-D69447DD13EE	3	3	3	9	3
8ffdde89-cb67-4707-a00c-b8d3089a7bf5	TCGA-AR-A256-01Z-00-DX1.950D4546-4BF4-4380-9877-51D86A93D755	3	3	2	8	3
d93a5cab-9a8a-42c8-973f-5868e748e8e7	TCGA-LD-A9QF-01Z-00-DX1.092108DF-1A60-459E-ACE6-5A71826A98D1	3	3	2	8	3
b411c802-4c8d-4b65-8eee-72a538f14f65	TCGA-C8-A12Q-01Z-00-DX1.CE74E5B7-FD30-4CBE-8716-ECCF2213AAC3	3	3	2	8	3
47ce0c0f-a5df-4a84-b980-b7f4ebbbeb9d	TCGA-B6-A0RO-01Z-00-DX1.3ADBFF05-92CE-41B5-BD49-CB3CE5B74CC9	1	2	1	4	1
bc6cd9e3-a33c-40da-bc82-cf85d2d03106	TCGA-A2-A25C-01Z-00-DX1.F8E6044A-435E-42D5-94FF-C0F572F1ED99	2	2	2	6	2
25aec062-60d1-446e-a1c6-0c79cc74a770	TCGA-E2-A154-01Z-00-DX1.01FC9B1A-8ECD-4467-9EDD-0B02E4AEEF72	3	3	2	8	3
1db81db6-77eb-4aee-9e20-5d9cad3d4917	TCGA-LL-A740-01Z-00-DX1.757D94A5-EF0F-4A0E-99A9-8809B66438DA	2	3	2	7	2
1bac9786-4d56-4f08-9792-ed131b398907	TCGA-D8-A1JH-01Z-00-DX1.4A4F2502-612C-421D-9F64-444BF2C85620	2	3	1	6	2
1bac9786-4d56-4f08-9792-ed131b398907	TCGA-E2-A573-01Z-00-DX1.D6633BC2-524D-4153-952F-6B1D8D067370	3	3	2	8	3
248c960a-1ed6-4393-9bdd-82dffe30786e	TCGA-BH-A0DX-01Z-00-DX1.45C27E71-9A0A-400E-93A9-5CE7780F3C5E	2	2	2	6	2
c796a50f-9129-4144-ba0b-e67f5d4fd5e8	TCGA-D8-A1JK-01Z-00-DX1.3190C919-A403-460D-9F6C-D2AB5FD3FD05	2	3	2	7	2
7a79416d-74da-48a6-8c44-df4f41b04e3a	TCGA-D8-A1XQ-01Z-00-DX1.1A17A5C7-F14B-4AD2-AD5F-D3400D86A366	3	3	3	9	3
672685f0-fe97-415f-b52b-e451ac87294f	TCGA-E2-A158-01Z-00-DX1.994C60FE-E651-4224-95E7-4669834F2338	1	2	1	4	1
c580a40b-fafe-4322-92b7-b065c8bd9f97	TCGA-E2-A10A-01Z-00-DX1.98B19EF1-0DAE-4DC6-8B0E-963CFABC6724	1	2	2	5	1
101c2a43-88d8-4cb8-b5bd-c8ec41e835b1	TCGA-EW-A1P6-01Z-00-DX1.A8024C26-6336-4858-88FD-5679795899BA	3	3	1	7	2
96f45c1d-a11a-428d-8160-f4a1b78d03b6	TCGA-D8-A1XY-01Z-00-DX2.33D96E5C-5291-4864-B282-8BACA2043586	2	3	2	7	2
ffd2e03b-702b-4a89-a3bc-25e3503e7a3c	TCGA-E9-A229-01Z-00-DX1.5B448B88-DA0C-44FF-87B3-20649A4A26FE	2	2	2	6	2
411e68e5-a78e-486d-aba6-fdef038f9082	TCGA-LL-A442-01Z-00-DX1.9275EDBD-1C89-4AF3-B02B-19F613A4E083	3	3	2	8	3
c0c80b78-109c-4f6c-8606-98cc0961c389	TCGA-BH-A0EI-01Z-00-DX1.929E126A-C9F8-4240-BF00-B6C4A57B7FF6	1	2	1	4	1
ce2b8842-4ddf-4415-9c7d-f211328c3d71	TCGA-A7-A0CE-01Z-00-DX2.5AD1DB65-10E7-4996-AB5E-13D7851EC5FA	3	3	2	8	3
ebef3a2a-227f-40f7-9a6d-5138fa43c872	TCGA-E9-A227-01Z-00-DX1.823062BF-3444-489B-AF91-AAD4ECAA1DC7	2	3	2	7	2
86867852-64fe-43e6-894d-b53b146c3518	TCGA-AR-A0TZ-01Z-00-DX1.2D58BE38-03F6-4310-8E06-F1A523FB0904	1	3	3	7	2
a8ec7eb3-4e63-4368-b45d-57c2cdfe1f45	TCGA-LL-A7T0-01Z-00-DX1.B03BBA63-ACF4-4BCA-9F2B-F631F0C6A25C	3	3	2	8	3
d661da33-ef97-4e13-a954-4e0eb6a16573	TCGA-C8-A132-01Z-00-DX1.6CCE1FE0-BB4B-4046-BAF0-43AA110B2EBE	3	3	2	8	3
bd74ee11-89ac-45f0-8257-a5f37bb14078	TCGA-E9-A1R0-01Z-00-DX1.187C58EA-132E-4B3C-BAD0-7F59101D5C4D	3	2	1	6	2
b9406762-f609-464e-a518-40277ce6bd7f	TCGA-BH-A0B0-01Z-00-DX1.316D35DB-7F13-4AE5-82A7-5716D2519669	2	2	2	6	2
b3f4c01e-4068-45bc-8a7f-e03683f12d42	TCGA-E2-A10E-01Z-00-DX1.C45030A9-CC1A-4BA7-8F62-872619C5AD5E	2	2	2	6	2
3631967b-07b8-4275-92fb-6f66abe465ec	TCGA-AQ-A0Y5-01Z-00-DX1.f68f5b49-30fa-4fb6-bec6-5da9f6809d02	3	2	2	7	2
b28b6590-f8f2-43fa-90f2-cf4b558797d9	TCGA-E2-A574-01Z-00-DX1.60341091-B118-4F20-9ADB-FB2886790B0E	2	3	3	8	3
43b25038-2f66-4bd4-b2af-69130271fd1d	TCGA-BH-A0DT-01Z-00-DX1.73AFCEBB-06B1-4870-ADA2-881511B1BE2D	1	2	1	4	1
86925470-1065-44f5-a4e5-a3c55a73c995	TCGA-OL-A5D6-01Z-00-DX1.6B11331B-4A0D-4E13-B054-A5C7A6FC3AAC	2	3	2	7	2
179185d2-5279-4e19-b9da-1f2296973156	TCGA-C8-A12Y-01Z-00-DX1.A15CB3E2-E145-4C75-8FEA-1DD503CD7C20	3	3	2	8	3
f6830586-1dc2-4cfd-8d99-b4b7c9161341	TCGA-D8-A1JE-01Z-00-DX2.CCF3DFEF-E851-425A-BCD0-0F7B377A00BC	1	2	3	6	2
66cfc895-af23-4992-9e27-ad3988b54af7	TCGA-BH-A1F6-01Z-00-DX1.E83F0DC0-EA2C-4641-81B0-8702B9C5D579	3	3	3	9	3
9f66d62b-cd1f-459a-9c4d-40b554faccca	TCGA-D8-A1XR-01Z-00-DX1.7F443346-C564-47B6-9736-6944230CAF46	2	3	2	7	2
81100322-018d-4957-bec7-c1833e288434	TCGA-D8-A1Y0-01Z-00-DX1.10F40197-4174-43CC-AAD3-8CB85154FB2D	2	2	2	6	2
f1b21fac-b36b-4fd4-b18f-b29337df4ce4	TCGA-E2-A14T-01Z-00-DX1.61B4C988-6D75-447B-A5F4-9DE92CEACC9F	2	3	1	6	2
edaed3cc-ec4f-4792-aab7-27078b0f30a3	TCGA-S3-AA10-01Z-00-DX1.C0468882-0DD8-4FC5-8C2F-E18BE8000F69	3	3	3	9	3
7d0b8ace-0a94-444a-b48e-6cdb4a7665ca	TCGA-A1-A0SM-01Z-00-DX1.AD503DBD-4D93-4476-B467-F091254FDF78	2	2	1	5	1
fb49bd4a-63e7-472d-9ff6-b297c46e43cf	TCGA-AR-A24Z-01Z-00-DX1.C88AFF18-A8E4-426A-BEA3-70566DE39C46	2	2	2	6	2
745f1703-c59d-4d43-b5ba-d908bf1498c3	TCGA-E2-A14R-01Z-00-DX1.DDE62ED5-1FC0-4B3B-A874-95C08B33AB20	3	3	3	9	3
ff1fe2c9-2f02-4a50-a0dc-1509a6b0d7a4	TCGA-D8-A1J8-01Z-00-DX1.EADAB43A-87C6-47FA-9120-25B69E23366D	2	3	3	8	3
c2c93798-a4df-47ff-a281-8960ae8c5c41	TCGA-C8-A12P-01Z-00-DX1.670B5DE8-07B0-4E4C-93FA-FA3DFFCCE50D	3	3	2	8	3
c713f828-2725-4901-a9fe-01d3192edc46	TCGA-GM-A2DB-01Z-00-DX1.9EE36AA6-2594-44C7-B05C-91A0AEC7E511	3	3	3	9	3
fb9961aa-e11f-4f75-bc5a-990e9302ae6e	TCGA-BH-A202-01Z-00-DX1.8CECDB74-5E6F-4CE8-B52C-A89E574F38FB	3	3	2	8	3
0a886f18-c44c-4b5e-b243-6df6e27f426a	TCGA-GI-A2C8-01Z-00-DX1.09BD8AC9-645A-4C8B-9B36-77D833BDBA09	3	3	2	8	3
d953668b-4a60-4e7c-a001-0d74e0ad6e93	TCGA-E2-A14Y-01Z-00-DX1.804A22A3-FD8D-4C8A-A766-48D28434DE22	3	3	2	8	3
73c4e299-8b32-4dc0-b83d-d1f45d78e1b4	TCGA-AC-A23E-01Z-00-DX1.F12A5A87-72CF-42F8-A6EC-8E7FAD80B1F7	2	2	1	5	1
8b5519e1-cbef-407d-b4b0-0a709cb9e924	TCGA-E2-A1LE-01Z-00-DX1.22856B2A-FBAA-4530-AEEC-E8F77BDA7F7F	2	3	1	6	2
8445cfc1-a20a-4b5b-861a-ae8412f4d497	TCGA-BH-A0BP-01Z-00-DX1.63A87C1D-87FA-494D-9836-74290B5DC30D	2	3	2	7	2
cce52fce-a092-4c66-af4e-a19cace6e154	TCGA-E2-A109-01Z-00-DX1.FCF5E9FC-F9FE-4F5F-96DD-5628E2609BEF	2	3	3	8	3
abf27f46-f39a-4f2d-8e7b-423e5da54419	TCGA-E2-A14X-01Z-00-DX1.24ADDA43-F127-4A6B-9AAD-2FAD982A853D	2	3	3	8	3
c9eda175-ef25-49f1-beb1-3a8cb99c1f37	TCGA-E2-A15J-01Z-00-DX1.BF7901D1-30B1-4A76-B0A5-E9B8B36EF4C9	1	2	1	4	1
044f28b0-564a-42d8-9df1-ba3fd4d6c014	TCGA-E9-A22D-01Z-00-DX1.b2867437-0add-4b7d-8002-fb09ed961942	2	3	2	7	2
2c83bc8f-ac3c-4749-921a-cdef6f872704	TCGA-BH-A0BT-01Z-00-DX1.9087B9E7-C0CD-4179-AF57-AD9255785169	2	2	2	6	2
96ce4bc0-f846-4a4a-b2a8-925ed5dd423e	TCGA-E2-A15K-01Z-00-DX1.9F424BE2-9BFE-4DFF-8CC9-10D2DADBBEA7	2	2	2	6	2
35ed3b43-99d6-4a9e-9b1b-1ded7e2ee7c7	TCGA-E2-A15O-01Z-00-DX1.4D0F3975-93EA-4DC2-AD0C-A76A24C3AE0C	2	3	2	7	2
6d4418e8-d621-4ace-9e1f-3fcc7d2c9546	TCGA-E2-A14Q-01Z-00-DX1.C19BA7FD-D986-4E3B-9A79-F3531A78F05D	2	3	2	7	2
63f10c46-8870-40dd-a531-6317306be707	TCGA-E2-A15D-01Z-00-DX1.AA5AF847-3635-4BAF-AAC3-BADB4A1B2CB1	2	3	2	7	2
86538d07-4a39-4c65-80fa-3e530660eb46	TCGA-AO-A12A-01Z-00-DX1.4E9609A7-9AAD-40A8-8344-8369DF998006	2	2	2	6	2
0ce5fcd8-3ace-4213-ae59-844c11a45f7b	TCGA-BH-A1EU-01Z-00-DX1.5A0956EF-0100-47FD-9026-1994CF22D0F1	2	3	1	6	2
c2e4fe6d-db89-42df-9594-8ef7a6d11490	TCGA-E2-A1IN-01Z-00-DX1.F63F004F-847D-41B0-BAEF-3189D4965838	2	3	2	7	2

UUID	SLIDE ID	Tubule formation	Nuclear pleomorphism	Mitotic count	Total score	Grade	PATHOLOGIST 2
d2c7ad27-6090-421e-a12e-2cb66a5417e4	**TCGA-E2-A1LK-01Z-00-DX1.5EBAA1F4-F1B4-4938-A51F-0246621BB0ED**	3	3	1	7	2	
b1e64f03-c419-421e-b801-2439bace5ad5	**TCGA-AO-A0JB-01Z-00-DX1.250FE098-345B-4981-9236-0519E1C9058E**	2	2	1	5	1	
a7ccf35e-56a9-4bd9-904c-59b1b0cc33b4	**TCGA-E2-A1L6-01Z-00-DX1.AFE87067-2BFD-42C2-9334-9DDE8AB61B49**	1	2	1	4	1	
d9ab7666-6cdd-4cb4-89fd-e7916e10986a	**TCGA-A1-A0SN-01Z-00-DX1.5E9B85AE-AFB7-41DC-8A1B-BD6DA39B6540**	2	2	1	5	1	
469579d1-f578-4956-aab0-9e7728a88ff7	**TCGA-A7-A13D-01Z-00-DX1.D206783C-FA6A-4B6A-B3AA-4132A2C9626B**	3	3	1	7	2	
454d3eb9-ff96-4cd4-9dec-17252819ed13	**TCGA-B6-A0WW-01Z-00-DX1.64048633-1D1A-4074-8C50-7641159355DB**	2	1	1	4	1	
9b91d3c8-c250-4f74-9ff7-bb8d11f813a7	**TCGA-D8-A1XL-01Z-00-DX1.FDF07020-8F40-4C00-9023-E5F40E0D8A7C**	3	2	2	7	2	
1f4d56a0-b948-4a65-8b3a-f66d30317e0b	**TCGA-D8-A13Y-01Z-00-DX2.0043D2BB-B04D-4A44-AAB1-1CAAD97AC246**	3	3	2	8	3	
b658e65c-636f-43b9-8c52-a46ba2850044	**TCGA-A2-A0SU-01Z-00-DX1.22420EE2-4FEB-42F3-9547-4739F0F73D50**	2	2	1	5	1	
f6f7b32b-33e4-462a-9f83-2a3f74a6302a	**TCGA-E2-A108-01Z-00-DX1.B110ED43-08A4-476A-A658-1CA75F7C0DDE**	1	1	1	3	1	
55eb1c6a-2290-4458-afa2-f259d80e6395	**TCGA-LL-A5YN-01Z-00-DX1.F221939B-3680-4B95-93A9-BE8599550E87**	2	2	1	5	1	
d29fb01b-dfb0-49fe-b42c-98dd32a306c1	**TCGA-OL-A5D7-01Z-00-DX1.A4A45393-9AE1-4370-8B92-CB85CDB04934**	3	3	3	9	3	
40b5da84-5b98-4444-9745-2f1ebe5132e4	**TCGA-WT-AB41-01Z-00-DX1.75BDFDF2-CD87-46D1-B32C-725741CB02BE**	2	1	1	4	1	
a18c60c5-3053-477a-b892-305bf4e5bfe7	**TCGA-D8-A27R-01Z-00-DX1.F6E2FD1C-0666-4788-8D95-A76D15907270**	2	3	2	7	2	
fcb893aa-c7aa-438d-9d6d-c0adb397fa1e	**TCGA-E2-A9RU-01Z-00-DX1.A06BE284-B9DC-4B45-A202-A9D027AEEDD9**	3	3	2	8	3	
68458986-1efd-4fce-9e65-ce5430ab125c	**TCGA-3C-AALJ-01Z-00-DX1.777C0957-255A-42F0-9EEB-A3606BCF0C96**	2	2	1	5	1	
069a8f13-04e2-41c7-ab64-90a465c7c4bc	**TCGA-AR-A0TQ-01Z-00-DX1.2BEA298C-6B3D-4133-ADBC-769E62CEFFA0**	2	3	1	6	2	
6f29dfa9-d166-4f07-9cdb-0daceb7341e9	**TCGA-D8-A27R-01Z-00-DX2.31F47D8F-DFD7-42AE-BBBA-7DBBA12FA97D**	2	2	1	5	1	
0dcc8304-dfd1-4e79-b23f-30f1143c897c	**TCGA-E2-A14N-01Z-00-DX1.15F5644F-CA9F-4688-B56E-BCC00CA4769B**	3	3	2	8	3	
63858a48-c99e-4080-97f0-419f662ef195	**TCGA-D8-A1XD-01Z-00-DX1.E500D561-1F49-4F08-99AE-E8345F21B406**	2	2	1	5	1	
0279f555-9738-41e0-b89f-ded25829adc0	**TCGA-E2-A1L9-01Z-00-DX1.F2CC1036-8EEE-4664-962E-541B8ACB10DE**	1	1	1	3	1	
7b3d2ef5-0b8b-4b3e-8210-c77f8302e437	**TCGA-E2-A15C-01Z-00-DX1.26E13415-1D37-43C7-9EBB-4411BE7FCE10**	2	2	1	5	1	
bcd6977f-b269-48c4-b088-ddf8d6d95240	**TCGA-GM-A2DF-01Z-00-DX1.CD0BE6D7-2DB3-4193-84CC-F9BE7BF18CC2**	1	2	1	4	1	
4fa9d2cb-379e-4183-b5c6-a8ba2a15ee57	**TCGA-AR-A0U1-01Z-00-DX1.276433E7-E841-42D2-AF21-762F2FEA3B9B**	2	3	1	6	2	
33f7413f-6339-4389-98dd-eee280233684	**TCGA-E2-A1LH-01Z-00-DX1.F85384B7-1EBF-4F57-A45B-4A668B68E535**	2	2	1	5	1	
9db0bfe8-9f66-4416-ab90-e0b1f91d2e01	**TCGA-D8-A1XR-01Z-00-DX2.A103FB8B-4397-4DD4-8587-90A736407484**	1	2	1	4	1	
f554759b-e293-45f5-be85-34a561e36027	**TCGA-AR-A0U2-01Z-00-DX1.03E2ADD9-F20F-44DA-93D1-D10BBD3844A5**	2	2	2	6	2	
75f48929-04a3-4970-8572-29b1068b2d85	**TCGA-D8-A147-01Z-00-DX1.159094F2-BB78-4910-B7AE-3D7CAAB1DAD9**	2	3	1	6	2	
45b31b73-adeb-4793-837f-8a9f957cffad	**TCGA-A2-A3XY-01Z-00-DX1.E57FC9BF-411E-4028-AC10-8BCA5D0C8472**	3	2	1	6	2	
e47bd352-9d96-4abd-b673-9d30038d218a	**TCGA-B6-A0RI-01Z-00-DX1.E39951A4-AC6A-4B07-851B-F35CB86D79AA**	1	1	1	3	1	
54d20f52-a7b1-4814-ba36-082108f23b59	**TCGA-E9-A22A-01Z-00-DX1.d986c9eb-2c54-4663-a54b-04c0756db6db**	2	2	1	5	1	
df1645c7-0db5-4262-8c8d-b8577f272914	**TCGA-A2-A0YJ-01Z-00-DX1.8135C74E-DAA8-4C8E-AF14-A4B5B57695BE**	3	3	2	8	3	
26eb27eb-84c8-4fea-a51b-c3eb5c484b33	**TCGA-E2-A1LI-01Z-00-DX1.503dd2fa-23ef-4b11-8aab-301e069eaa88**	3	3	2	8	3	
e5069ea5-1011-46cf-9a3c-925825eeb81e	**TCGA-A2-A0T5-01Z-00-DX1.128C288B-B357-439B-A8D4-8E7DEBF73E4E**	1	1	1	3	1	
e15f67af-fb62-42c8-a451-83b035867106	**TCGA-D8-A1JD-01Z-00-DX1.6D215B14-DD90-4635-8645-AF06EBD9BA3F**	1	2	1	4	1	
f273a858-1cfa-4983-ace4-b1e876d64473	**TCGA-A1-A0SF-01Z-00-DX1.7F252D89-EA78-419F-A969-1B7313D77499**	1	2	1	4	1	
9b8dec44-e674-4dc7-a8a8-c962c907b64b	**TCGA-D8-A1JC-01Z-00-DX2.854ABF5D-40F1-48AE-802F-97D75497F1FD**	2	1	1	4	1	
206ccd79-310d-40a4-852c-03930e685304	**TCGA-EW-A6SB-01Z-00-DX1.D56E1922-01A9-4AEE-AB95-D69447DD13EE**	3	3	2	8	3	
8ffdde89-cb67-4707-a00c-b8d3089a7bf5	**TCGA-AR-A256-01Z-00-DX1.950D4546-4BF4-4380-9877-51D86A93D755**	3	2	1	6	2	
d93a5cab-9a8a-42c8-973f-5868e748e8e7	**TCGA-LD-A9QF-01Z-00-DX1.092108DF-1A60-459E-ACE6-5A71826A98D1**	3	2	1	6	2	
b411c802-4c8d-4b65-8eee-72a538f14f65	**TCGA-C8-A12Q-01Z-00-DX1.CE74E5B7-FD30-4CBE-8716-ECCF2213AAC3**	2	3	1	6	2	
47ce0c0f-a5df-4a84-b980-b7f4ebbbeb9d	**TCGA-B6-A0RO-01Z-00-DX1.3ADBFF05-92CE-41B5-BD49-CB3CE5B74CC9**	1	1	1	3	1	
bc6cd9e3-a33c-40da-bc82-cf85d2d03106	**TCGA-A2-A25C-01Z-00-DX1.F8E6044A-435E-42D5-94FF-C0F572F1ED99**	1	1	1	3	1	
25aec062-60d1-446e-a1c6-0c79cc74a770	**TCGA-E2-A154-01Z-00-DX1.01FC9B1A-8ECD-4467-9EDD-0B02E4AEEF72**	3	1	1	5	1	
1db81db6-77eb-4aee-9e20-5d9cad3d4917	**TCGA-LL-A740-01Z-00-DX1.757D94A5-EF0F-4A0E-99A9-8809B66438DA**	2	2	1	5	1	
1bac9786-4d56-4f08-9792-ed131b398907	**TCGA-D8-A1JH-01Z-00-DX1.4A4F2502-612C-421D-9F64-444BF2C85620**	2	2	1	5	1	
1bac9786-4d56-4f08-9792-ed131b398907	**TCGA-E2-A573-01Z-00-DX1.D6633BC2-524D-4153-952F-6B1D8D067370**	3	2	1	6	2	
248c960a-1ed6-4393-9bdd-82dffe30786e	**TCGA-BH-A0DX-01Z-00-DX1.45C27E71-9A0A-400E-93A9-5CE7780F3C5E**	1	1	1	3	1	
c796a50f-9129-4144-ba0b-e67f5d4fd5e8	**TCGA-D8-A1JK-01Z-00-DX1.3190C919-A403-460D-9F6C-D2AB5FD3FD05**	3	3	1	7	2	
7a79416d-74da-48a6-8c44-df4f41b04e3a	**TCGA-D8-A1XQ-01Z-00-DX1.1A17A5C7-F14B-4AD2-AD5F-D3400D86A366**	3	3	1	7	2	
672685f0-fe97-415f-b52b-e451ac87294f	**TCGA-E2-A158-01Z-00-DX1.994C60FE-E651-4224-95E7-4669834F2338**	1	1	1	3	1	
c580a40b-fafe-4322-92b7-b065c8bd9f97	**TCGA-E2-A10A-01Z-00-DX1.98B19EF1-0DAE-4DC6-8B0E-963CFABC6724**	1	2	1	4	1	
101c2a43-88d8-4cb8-b5bd-c8ec41e835b1	**TCGA-EW-A1P6-01Z-00-DX1.A8024C26-6336-4858-88FD-5679795899BA**	2	2	1	5	1	

96f45c1d-a11a-428d-8160-f4a1b78d03b6	**TCGA-D8-A1XY-01Z-00-DX2.33D96E5C-5291-4864-B282-8BACA2043586**	2	2	1	5	1
ffd2e03b-702b-4a89-a3bc-25e3503e7a3c	**TCGA-E9-A229-01Z-00-DX1.5B448B88-DA0C-44FF-87B3-20649A4A26FE**	2	1	1	4	1
411e68e5-a78e-486d-aba6-fdef038f9082	**TCGA-LL-A442-01Z-00-DX1.9275EDBD-1C89-4AF3-B02B-19F613A4E083**	3	2	1	6	2
c0c80b78-109c-4f6c-8606-98cc0961c389	**TCGA-BH-A0EI-01Z-00-DX1.929E126A-C9F8-4240-BF00-B6C4A57B7FF6**	1	1	1	3	1
ce2b8842-4ddf-4415-9c7d-f211328c3d71	**TCGA-A7-A0CE-01Z-00-DX2.5AD1DB65-10E7-4996-AB5E-13D7851EC5FA**	3	3	2	8	3
ebef3a2a-227f-40f7-9a6d-5138fa43c872	**TCGA-E9-A227-01Z-00-DX1.823062BF-3444-489B-AF91-AAD4ECAA1DC7**	2	2	1	5	1
86867852-64fe-43e6-894d-b53b146c3518	**TCGA-AR-A0TZ-01Z-00-DX1.2D58BE38-03F6-4310-8E06-F1A523FB0904**	2	2	1	5	1
a8ec7eb3-4e63-4368-b45d-57c2cdfe1f45	**TCGA-LL-A7T0-01Z-00-DX1.B03BBA63-ACF4-4BCA-9F2B-F631F0C6A25C**	3	3	2	8	3
d661da33-ef97-4e13-a954-4e0eb6a16573	**TCGA-C8-A132-01Z-00-DX1.6CCE1FE0-BB4B-4046-BAF0-43AA110B2EBE**	3	3	1	7	2
bd74ee11-89ac-45f0-8257-a5f37bb14078	**TCGA-E9-A1R0-01Z-00-DX1.187C58EA-132E-4B3C-BAD0-7F59101D5C4D**	3	3	1	7	2
b9406762-f609-464e-a518-40277ce6bd7f	**TCGA-BH-A0B0-01Z-00-DX1.316D35DB-7F13-4AE5-82A7-5716D2519669**	2	2	1	5	1
b3f4c01e-4068-45bc-8a7f-e03683f12d42	**TCGA-E2-A10E-01Z-00-DX1.C45030A9-CC1A-4BA7-8F62-872619C5AD5E**	1	2	1	4	1
3631967b-07b8-4275-92fb-6f66abe465ec	**TCGA-AQ-A0Y5-01Z-00-DX1.f68f5b49-30fa-4fb6-bec6-5da9f6809d02**	3	2	1	6	2
b28b6590-f8f2-43fa-90f2-cf4b558797d9	**TCGA-E2-A574-01Z-00-DX1.60341091-B118-4F20-9ADB-FB2886790B0E**	2	2	2	6	2
43b25038-2f66-4bd4-b2af-69130271fd1d	**TCGA-BH-A0DT-01Z-00-DX1.73AFCEBB-06B1-4870-ADA2-881511B1BE2D**	1	1	1	3	1
86925470-1065-44f5-a4e5-a3c55a73c995	**TCGA-OL-A5D6-01Z-00-DX1.6B11331B-4A0D-4E13-B054-A5C7A6FC3AAC**	2	3	1	6	2
179185d2-5279-4e19-b9da-1f2296973156	**TCGA-C8-A12Y-01Z-00-DX1.A15CB3E2-E145-4C75-8FEA-1DD503CD7C20**	3	2	1	6	2
f6830586-1dc2-4cfd-8d99-b4b7c9161341	**TCGA-D8-A1JE-01Z-00-DX2.CCF3DFEF-E851-425A-BCD0-0F7B377A00BC**	2	1	1	4	1
66cfc895-af23-4992-9e27-ad3988b54af7	**TCGA-BH-A1F6-01Z-00-DX1.E83F0DC0-EA2C-4641-81B0-8702B9C5D579**	2	3	1	6	2
9f66d62b-cd1f-459a-9c4d-40b554faccca	**TCGA-D8-A1XR-01Z-00-DX1.7F443346-C564-47B6-9736-6944230CAF46**	1	2	1	4	1
81100322-018d-4957-bec7-c1833e288434	**TCGA-D8-A1Y0-01Z-00-DX1.10F40197-4174-43CC-AAD3-8CB85154FB2D**	2	2	1	5	1
f1b21fac-b36b-4fd4-b18f-b29337df4ce4	**TCGA-E2-A14T-01Z-00-DX1.61B4C988-6D75-447B-A5F4-9DE92CEACC9F**	2	2	1	5	1
edaed3cc-ec4f-4792-aab7-27078b0f30a3	**TCGA-S3-AA10-01Z-00-DX1.C0468882-0DD8-4FC5-8C2F-E18BE8000F69**	3	3	2	8	3
7d0b8ace-0a94-444a-b48e-6cdb4a7665ca	**TCGA-A1-A0SM-01Z-00-DX1.AD503DBD-4D93-4476-B467-F091254FDF78**	3	3	1	7	2
fb49bd4a-63e7-472d-9ff6-b297c46e43cf	**TCGA-AR-A24Z-01Z-00-DX1.C88AFF18-A8E4-426A-BEA3-70566DE39C46**	2	3	1	6	2
745f1703-c59d-4d43-b5ba-d908bf1498c3	**TCGA-E2-A14R-01Z-00-DX1.DDE62ED5-1FC0-4B3B-A874-95C08B33AB20**	3	3	2	8	3
ff1fe2c9-2f02-4a50-a0dc-1509a6b0d7a4	**TCGA-D8-A1J8-01Z-00-DX1.EADAB43A-87C6-47FA-9120-25B69E23366D**	2	3	1	6	2
c2c93798-a4df-47ff-a281-8960ae8c5c41	**TCGA-C8-A12P-01Z-00-DX1.670B5DE8-07B0-4E4C-93FA-FA3DFFCCE50D**	3	3	1	7	2
c713f828-2725-4901-a9fe-01d3192edc46	**TCGA-GM-A2DB-01Z-00-DX1.9EE36AA6-2594-44C7-B05C-91A0AEC7E511**	3	2	1	6	2
fb9961aa-e11f-4f75-bc5a-990e9302ae6e	**TCGA-BH-A202-01Z-00-DX1.8CECDB74-5E6F-4CE8-B52C-A89E574F38FB**	3	3	2	8	3
0a886f18-c44c-4b5e-b243-6df6e27f426a	**TCGA-GI-A2C8-01Z-00-DX1.09BD8AC9-645A-4C8B-9B36-77D833BDBA09**	2	3	1	6	2
d953668b-4a60-4e7c-a001-0d74e0ad6e93	**TCGA-E2-A14Y-01Z-00-DX1.804A22A3-FD8D-4C8A-A766-48D28434DE22**	3	3	2	8	3
73c4e299-8b32-4dc0-b83d-d1f45d78e1b4	**TCGA-AC-A23E-01Z-00-DX1.F12A5A87-72CF-42F8-A6EC-8E7FAD80B1F7**	1	1	1	3	1
8b5519e1-cbef-407d-b4b0-0a709cb9e924	**TCGA-E2-A1LE-01Z-00-DX1.22856B2A-FBAA-4530-AEEC-E8F77BDA7F7F**	2	3	1	6	2
8445cfc1-a20a-4b5b-861a-ae8412f4d497	**TCGA-BH-A0BP-01Z-00-DX1.63A87C1D-87FA-494D-9836-74290B5DC30D**	1	2	1	4	1
cce52fce-a092-4c66-af4e-a19cace6e154	**TCGA-E2-A109-01Z-00-DX1.FCF5E9FC-F9FE-4F5F-96DD-5628E2609BEF**	3	3	2	8	3
abf27f46-f39a-4f2d-8e7b-423e5da54419	**TCGA-E2-A14X-01Z-00-DX1.24ADDA43-F127-4A6B-9AAD-2FAD982A853D**	3	3	1	7	2
c9eda175-ef25-49f1-beb1-3a8cb99c1f37	**TCGA-E2-A15J-01Z-00-DX1.BF7901D1-30B1-4A76-B0A5-E9B8B36EF4C9**	2	3	1	6	2
044f28b0-564a-42d8-9df1-ba3fd4d6c014	**TCGA-E9-A22D-01Z-00-DX1.b2867437-0add-4b7d-8002-fb09ed961942**	2	3	1	6	2
2c83bc8f-ac3c-4749-921a-cdef6f872704	**TCGA-BH-A0BT-01Z-00-DX1.9087B9E7-C0CD-4179-AF57-AD9255785169**	1	1	1	3	1
96ce4bc0-f846-4a4a-b2a8-925ed5dd423e	**TCGA-E2-A15K-01Z-00-DX1.9F424BE2-9BFE-4DFF-8CC9-10D2DADBBEA7**	3	2	1	6	2
35ed3b43-99d6-4a9e-9b1b-1ded7e2ee7c7	**TCGA-E2-A15O-01Z-00-DX1.4D0F3975-93EA-4DC2-AD0C-A76A24C3AE0C**	2	3	1	6	2
6d4418e8-d621-4ace-9e1f-3fcc7d2c9546	**TCGA-E2-A14Q-01Z-00-DX1.C19BA7FD-D986-4E3B-9A79-F3531A78F05D**	2	3	1	6	2
63f10c46-8870-40dd-a531-6317306be707	**TCGA-E2-A15D-01Z-00-DX1.AA5AF847-3635-4BAF-AAC3-BADB4A1B2CB1**	1	2	1	4	1
86538d07-4a39-4c65-80fa-3e530660eb46	**TCGA-AO-A12A-01Z-00-DX1.4E9609A7-9AAD-40A8-8344-8369DF998006**	2	2	1	5	1
0ce5fcd8-3ace-4213-ae59-844c11a45f7b	**TCGA-BH-A1EU-01Z-00-DX1.5A0956EF-0100-47FD-9026-1994CF22D0F1**	2	2	1	5	1
c2e4fe6d-db89-42df-9594-8ef7a6d11490	**TCGA-E2-A1IN-01Z-00-DX1.F63F004F-847D-41B0-BAEF-3189D4965838**	2	2	1	5	1

PATHOLOGIST 3

UUID	SLIDE ID	Tubule formation	Nuclear pleomorphism	Mitotic count	Total score	Grade
d2c7ad27-6090-421e-a12e-2cb66a5417e4	**TCGA-E2-A1LK-01Z-00-DX1.5EBAA1F4-F1B4-4938-A51F-0246621BB0ED**	3	3	2	8	3
b1e64f03-c419-421e-b801-2439bace5ad5	**TCGA-AO-A0JB-01Z-00-DX1.250FE098-345B-4981-9236-0519E1C9058E**	3	3	2	8	3
a7ccf35e-56a9-4bd9-904c-59b1b0cc33b4	**TCGA-E2-A1L6-01Z-00-DX1.AFE87067-2BFD-42C2-9334-9DDE8AB61B49**	1	2	1	4	1
d9ab7666-6cdd-4cb4-89fd-e7916e10986a	**TCGA-A1-A0SN-01Z-00-DX1.5E9B85AE-AFB7-41DC-8A1B-BD6DA39B6540**	3	3	1	7	2
469579d1-f578-4956-aab0-9e7728a88ff7	**TCGA-A7-A13D-01Z-00-DX1.D206783C-FA6A-4B6A-B3AA-4132A2C9626B**	2	3	1	6	2
454d3eb9-ff96-4cd4-9dec-17252819ed13	**TCGA-B6-A0WW-01Z-00-DX1.64048633-1D1A-4074-8C50-7641159355DB**	2	2	1	5	1
9b91d3c8-c250-4f74-9ff7-bb8d11f813a7	**TCGA-D8-A1XL-01Z-00-DX1.FDF07020-8F40-4C00-9023-E5F40E0D8A7C**	2	3	1	6	2
1f4d56a0-b948-4a65-8b3a-f66d30317e0b	**TCGA-D8-A13Y-01Z-00-DX2.0043D2BB-B04D-4A44-AAB1-1CAAD97AC246**	3	3	3	9	3
b658e65c-636f-43b9-8c52-a46ba2850044	**TCGA-A2-A0SU-01Z-00-DX1.22420EE2-4FEB-42F3-9547-4739F0F73D50**	2	3	2	7	2
f6f7b32b-33e4-462a-9f83-2a3f74a6302a	**TCGA-E2-A108-01Z-00-DX1.B110ED43-08A4-476A-A658-1CA75F7C0DDE**	1	2	1	4	1
55eb1c6a-2290-4458-afa2-f259d80e6395	**TCGA-LL-A5YN-01Z-00-DX1.F221939B-3680-4B95-93A9-BE8599550E87**	1	3	3	7	2
d29fb01b-dfb0-49fe-b42c-98dd32a306c1	**TCGA-OL-A5D7-01Z-00-DX1.A4A45393-9AE1-4370-8B92-CB85CDB04934**	3	3	3	9	3
40b5da84-5b98-4444-9745-2f1ebe5132e4	**TCGA-WT-AB41-01Z-00-DX1.75BDFDF2-CD87-46D1-B32C-725741CB02BE**	2	2	2	6	2
a18c60c5-3053-477a-b892-305bf4e5bfe7	**TCGA-D8-A27R-01Z-00-DX1.F6E2FD1C-0666-4788-8D95-A76D15907270**	3	3	2	8	3
fcb893aa-c7aa-438d-9d6d-c0adb397fa1e	**TCGA-E2-A9RU-01Z-00-DX1.A06BE284-B9DC-4B45-A202-A9D027AEEDD9**	3	3	3	9	3
68458986-1efd-4fce-9e65-ce5430ab125c	**TCGA-3C-AALJ-01Z-00-DX1.777C0957-255A-42F0-9EEB-A3606BCF0C96**	2	3	2	7	2
069a8f13-04e2-41c7-ab64-90a465c7c4bc	**TCGA-AR-A0TQ-01Z-00-DX1.2BEA298C-6B3D-4133-ADBC-769E62CEFFA0**	2	3	3	8	3
6f29dfa9-d166-4f07-9cdb-0daceb7341e9	**TCGA-D8-A27R-01Z-00-DX2.31F47D8F-DFD7-42AE-BBBA-7DBBA12FA97D**	3	3	2	8	3
0dcc8304-dfd1-4e79-b23f-30f1143c897c	**TCGA-E2-A14N-01Z-00-DX1.15F5644F-CA9F-4688-B56E-BCC00CA4769B**	3	3	3	9	3
63858a48-c99e-4080-97f0-419f662ef195	**TCGA-D8-A1XD-01Z-00-DX1.E500D561-1F49-4F08-99AE-E8345F21B406**	2	2	1	5	1
0279f555-9738-41e0-b89f-ded25829adc0	**TCGA-E2-A1L9-01Z-00-DX1.F2CC1036-8EEE-4664-962E-541B8ACB10DE**	2	2	1	5	1
7b3d2ef5-0b8b-4b3e-8210-c77f8302e437	**TCGA-E2-A15C-01Z-00-DX1.26E13415-1D37-43C7-9EBB-4411BE7FCE10**	2	2	2	6	2
bcd6977f-b269-48c4-b088-ddf8d6d95240	**TCGA-GM-A2DF-01Z-00-DX1.CD0BE6D7-2DB3-4193-84CC-F9BE7BF18CC2**	2	2	1	5	1
4fa9d2cb-379e-4183-b5c6-a8ba2a15ee57	**TCGA-AR-A0U1-01Z-00-DX1.276433E7-E841-42D2-AF21-762F2FEA3B9B**	2	3	3	8	3
33f7413f-6339-4389-98dd-eee280233684	**TCGA-E2-A1LH-01Z-00-DX1.F85384B7-1EBF-4F57-A45B-4A668B68E535**	2	2	2	6	2
9db0bfe8-9f66-4416-ab90-e0b1f91d2e01	**TCGA-D8-A1XR-01Z-00-DX2.A103FB8B-4397-4DD4-8587-90A736407484**	2	3	2	7	2
f554759b-e293-45f5-be85-34a561e36027	**TCGA-AR-A0U2-01Z-00-DX1.03E2ADD9-F20F-44DA-93D1-D10BBD3844A5**	2	3	3	8	3
75f48929-04a3-4970-8572-29b1068b2d85	**TCGA-D8-A147-01Z-00-DX1.159094F2-BB78-4910-B7AE-3D7CAAB1DAD9**	2	2	2	6	2
45b31b73-adeb-4793-837f-8a9f957cffad	**TCGA-A2-A3XY-01Z-00-DX1.E57FC9BF-411E-4028-AC10-8BCA5D0C8472**	3	3	2	8	3
e47bd352-9d96-4abd-b673-9d30038d218a	**TCGA-B6-A0RI-01Z-00-DX1.E39951A4-AC6A-4B07-851B-F35CB86D79AA**	2	1	2	5	1
54d20f52-a7b1-4814-ba36-082108f23b59	**TCGA-E9-A22A-01Z-00-DX1.d986c9eb-2c54-4663-a54b-04c0756db6db**	2	3	1	6	2
df1645c7-0db5-4262-8c8d-b8577f272914	**TCGA-A2-A0YJ-01Z-00-DX1.8135C74E-DAA8-4C8E-AF14-A4B5B57695BE**	3	3	2	8	3
26eb27eb-84c8-4fea-a51b-c3eb5c484b33	**TCGA-E2-A1LI-01Z-00-DX1.503dd2fa-23ef-4b11-8aab-301e069eaa88**	3	3	2	8	3
e5069ea5-1011-46cf-9a3c-925825eeb81e	**TCGA-A2-A0T5-01Z-00-DX1.128C288B-B357-439B-A8D4-8E7DEBF73E4E**	1	2	1	4	1
e15f67af-fb62-42c8-a451-83b035867106	**TCGA-D8-A1JD-01Z-00-DX1.6D215B14-DD90-4635-8645-AF06EBD9BA3F**	2	2	1	5	1
f273a858-1cfa-4983-ace4-b1e876d64473	**TCGA-A1-A0SF-01Z-00-DX1.7F252D89-EA78-419F-A969-1B7313D77499**	2	1	1	4	1
9b8dec44-e674-4dc7-a8a8-c962c907b64b	**TCGA-D8-A1JC-01Z-00-DX2.854ABF5D-40F1-48AE-802F-97D75497F1FD**	2	3	2	7	2
206ccd79-310d-40a4-852c-03930e685304	**TCGA-EW-A6SB-01Z-00-DX1.D56E1922-01A9-4AEE-AB95-D69447DD13EE**	3	3	2	8	3
8ffdde89-cb67-4707-a00c-b8d3089a7bf5	**TCGA-AR-A256-01Z-00-DX1.950D4546-4BF4-4380-9877-51D86A93D755**	3	2	1	6	2
d93a5cab-9a8a-42c8-973f-5868e748e8e7	**TCGA-LD-A9QF-01Z-00-DX1.092108DF-1A60-459E-ACE6-5A71826A98D1**	3	3	1	7	2
b411c802-4c8d-4b65-8eee-72a538f14f65	**TCGA-C8-A12Q-01Z-00-DX1.CE74E5B7-FD30-4CBE-8716-ECCF2213AAC3**	2	3	1	6	2
47ce0c0f-a5df-4a84-b980-b7f4ebbbeb9d	**TCGA-B6-A0RO-01Z-00-DX1.3ADBFF05-92CE-41B5-BD49-CB3CE5B74CC9**	2	2	1	5	1
bc6cd9e3-a33c-40da-bc82-cf85d2d03106	**TCGA-A2-A25C-01Z-00-DX1.F8E6044A-435E-42D5-94FF-C0F572F1ED99**	3	3	1	7	2
25aec062-60d1-446e-a1c6-0c79cc74a770	**TCGA-E2-A154-01Z-00-DX1.01FC9B1A-8ECD-4467-9EDD-0B02E4AEEF72**	3	3	1	7	2
1db81db6-77eb-4aee-9e20-5d9cad3d4917	**TCGA-LL-A740-01Z-00-DX1.757D94A5-EF0F-4A0E-99A9-8809B66438DA**	2	3	2	7	2
1bac9786-4d56-4f08-9792-ed131b398907	**TCGA-D8-A1JH-01Z-00-DX1.4A4F2502-612C-421D-9F64-444BF2C85620**	2	3	1	6	2
1bac9786-4d56-4f08-9792-ed131b398907	**TCGA-E2-A573-01Z-00-DX1.D6633BC2-524D-4153-952F-6B1D8D067370**	3	3	2	8	3
248c960a-1ed6-4393-9bdd-82dffe30786e	**TCGA-BH-A0DX-01Z-00-DX1.45C27E71-9A0A-400E-93A9-5CE7780F3C5E**	2	2	1	5	1
c796a50f-9129-4144-ba0b-e67f5d4fd5e8	**TCGA-D8-A1JK-01Z-00-DX1.3190C919-A403-460D-9F6C-D2AB5FD3FD05**	2	3	1	6	2
7a79416d-74da-48a6-8c44-df4f41b04e3a	**TCGA-D8-A1XQ-01Z-00-DX1.1A17A5C7-F14B-4AD2-AD5F-D3400D86A366**	3	3	2	8	3
672685f0-fe97-415f-b52b-e451ac87294f	**TCGA-E2-A158-01Z-00-DX1.994C60FE-E651-4224-95E7-4669834F2338**	1	1	1	3	1

c580a40b-fafe-4322-92b7-b065c8bd9f97	**TCGA-E2-A10A-01Z-00-DX1.98B19EF1-0DAE-4DC6-8B0E-963CFABC6724**	2	2	2	6	2
101c2a43-88d8-4cb8-b5bd-c8ec41e835b1	**TCGA-EW-A1P6-01Z-00-DX1.A8024C26-6336-4858-88FD-5679795899BA**	3	3	1	7	2
96f45c1d-a11a-428d-8160-f4a1b78d03b6	**TCGA-D8-A1XY-01Z-00-DX2.33D96E5C-5291-4864-B282-8BACA2043586**	2	3	2	7	2
ffd2e03b-702b-4a89-a3bc-25e3503e7a3c	**TCGA-E9-A229-01Z-00-DX1.5B448B88-DA0C-44FF-87B3-20649A4A26FE**	2	2	1	5	1
411e68e5-a78e-486d-aba6-fdef038f9082	**TCGA-LL-A442-01Z-00-DX1.9275EDBD-1C89-4AF3-B02B-19F613A4E083**	3	3	1	7	2
c0c80b78-109c-4f6c-8606-98cc0961c389	**TCGA-BH-A0EI-01Z-00-DX1.929E126A-C9F8-4240-BF00-B6C4A57B7FF6**	1	2	1	4	1
ce2b8842-4ddf-4415-9c7d-f211328c3d71	**TCGA-A7-A0CE-01Z-00-DX2.5AD1DB65-10E7-4996-AB5E-13D7851EC5FA**	3	3	3	9	3
ebef3a2a-227f-40f7-9a6d-5138fa43c872	**TCGA-E9-A227-01Z-00-DX1.823062BF-3444-489B-AF91-AAD4ECAA1DC7**	2	3	1	6	2
86867852-64fe-43e6-894d-b53b146c3518	**TCGA-AR-A0TZ-01Z-00-DX1.2D58BE38-03F6-4310-8E06-F1A523FB0904**	3	3	2	8	3
a8ec7eb3-4e63-4368-b45d-57c2cdfe1f45	**TCGA-LL-A7T0-01Z-00-DX1.B03BBA63-ACF4-4BCA-9F2B-F631F0C6A25C**	3	3	3	9	3
d661da33-ef97-4e13-a954-4e0eb6a16573	**TCGA-C8-A132-01Z-00-DX1.6CCE1FE0-BB4B-4046-BAF0-43AA110B2EBE**	3	3	1	7	2
bd74ee11-89ac-45f0-8257-a5f37bb14078	**TCGA-E9-A1R0-01Z-00-DX1.187C58EA-132E-4B3C-BAD0-7F59101D5C4D**	3	3	1	7	2
b9406762-f609-464e-a518-40277ce6bd7f	**TCGA-BH-A0B0-01Z-00-DX1.316D35DB-7F13-4AE5-82A7-5716D2519669**	3	3	1	7	2
b3f4c01e-4068-45bc-8a7f-e03683f12d42	**TCGA-E2-A10E-01Z-00-DX1.C45030A9-CC1A-4BA7-8F62-872619C5AD5E**	2	2	2	6	2
3631967b-07b8-4275-92fb-6f66abe465ec	**TCGA-AQ-A0Y5-01Z-00-DX1.f68f5b49-30fa-4fb6-bec6-5da9f6809d02**	3	3	1	7	2
b28b6590-f8f2-43fa-90f2-cf4b558797d9	**TCGA-E2-A574-01Z-00-DX1.60341091-B118-4F20-9ADB-FB2886790B0E**	2	3	2	7	2
43b25038-2f66-4bd4-b2af-69130271fd1d	**TCGA-BH-A0DT-01Z-00-DX1.73AFCEBB-06B1-4870-ADA2-881511B1BE2D**	1	2	1	4	1
86925470-1065-44f5-a4e5-a3c55a73c995	**TCGA-OL-A5D6-01Z-00-DX1.6B11331B-4A0D-4E13-B054-A5C7A6FC3AAC**	3	3	1	7	2
179185d2-5279-4e19-b9da-1f2296973156	**TCGA-C8-A12Y-01Z-00-DX1.A15CB3E2-E145-4C75-8FEA-1DD503CD7C20**	3	3	2	8	3
f6830586-1dc2-4cfd-8d99-b4b7c9161341	**TCGA-D8-A1JE-01Z-00-DX2.CCF3DFEF-E851-425A-BCD0-0F7B377A00BC**	2	3	2	7	2
66cfc895-af23-4992-9e27-ad3988b54af7	**TCGA-BH-A1F6-01Z-00-DX1.E83F0DC0-EA2C-4641-81B0-8702B9C5D579**	2	3	2	7	2
9f66d62b-cd1f-459a-9c4d-40b554faccca	**TCGA-D8-A1XR-01Z-00-DX1.7F443346-C564-47B6-9736-6944230CAF46**	2	3	2	7	2
81100322-018d-4957-bec7-c1833e288434	**TCGA-D8-A1Y0-01Z-00-DX1.10F40197-4174-43CC-AAD3-8CB85154FB2D**	3	3	1	7	2
f1b21fac-b36b-4fd4-b18f-b29337df4ce4	**TCGA-E2-A14T-01Z-00-DX1.61B4C988-6D75-447B-A5F4-9DE92CEACC9F**	2	3	1	6	2
edaed3cc-ec4f-4792-aab7-27078b0f30a3	**TCGA-S3-AA10-01Z-00-DX1.C0468882-0DD8-4FC5-8C2F-E18BE8000F69**	3	3	3	9	3
7d0b8ace-0a94-444a-b48e-6cdb4a7665ca	**TCGA-A1-A0SM-01Z-00-DX1.AD503DBD-4D93-4476-B467-F091254FDF78**	2	3	1	6	2
fb49bd4a-63e7-472d-9ff6-b297c46e43cf	**TCGA-AR-A24Z-01Z-00-DX1.C88AFF18-A8E4-426A-BEA3-70566DE39C46**	2	2	1	5	1
745f1703-c59d-4d43-b5ba-d908bf1498c3	**TCGA-E2-A14R-01Z-00-DX1.DDE62ED5-1FC0-4B3B-A874-95C08B33AB20**	3	2	2	7	2
ff1fe2c9-2f02-4a50-a0dc-1509a6b0d7a4	**TCGA-D8-A1J8-01Z-00-DX1.EADAB43A-87C6-47FA-9120-25B69E23366D**	2	3	1	6	2
c2c93798-a4df-47ff-a281-8960ae8c5c41	**TCGA-C8-A12P-01Z-00-DX1.670B5DE8-07B0-4E4C-93FA-FA3DFFCCE50D**	3	3	1	7	2
c713f828-2725-4901-a9fe-01d3192edc46	**TCGA-GM-A2DB-01Z-00-DX1.9EE36AA6-2594-44C7-B05C-91A0AEC7E511**	2	2	2	6	2
fb9961aa-e11f-4f75-bc5a-990e9302ae6e	**TCGA-BH-A202-01Z-00-DX1.8CECDB74-5E6F-4CE8-B52C-A89E574F38FB**	3	3	2	8	3
0a886f18-c44c-4b5e-b243-6df6e27f426a	**TCGA-GI-A2C8-01Z-00-DX1.09BD8AC9-645A-4C8B-9B36-77D833BDBA09**	3	3	1	7	2
d953668b-4a60-4e7c-a001-0d74e0ad6e93	**TCGA-E2-A14Y-01Z-00-DX1.804A22A3-FD8D-4C8A-A766-48D28434DE22**	3	3	2	8	3
73c4e299-8b32-4dc0-b83d-d1f45d78e1b4	**TCGA-AC-A23E-01Z-00-DX1.F12A5A87-72CF-42F8-A6EC-8E7FAD80B1F7**	2	1	1	4	1
8b5519e1-cbef-407d-b4b0-0a709cb9e924	**TCGA-E2-A1LE-01Z-00-DX1.22856B2A-FBAA-4530-AEEC-E8F77BDA7F7F**	3	2	1	6	2
8445cfc1-a20a-4b5b-861a-ae8412f4d497	**TCGA-BH-A0BP-01Z-00-DX1.63A87C1D-87FA-494D-9836-74290B5DC30D**	2	2	1	5	1
cce52fce-a092-4c66-af4e-a19cace6e154	**TCGA-E2-A109-01Z-00-DX1.FCF5E9FC-F9FE-4F5F-96DD-5628E2609BEF**	3	2	2	7	2
abf27f46-f39a-4f2d-8e7b-423e5da54419	**TCGA-E2-A14X-01Z-00-DX1.24ADDA43-F127-4A6B-9AAD-2FAD982A853D**	3	3	2	8	3
c9eda175-ef25-49f1-beb1-3a8cb99c1f37	**TCGA-E2-A15J-01Z-00-DX1.BF7901D1-30B1-4A76-B0A5-E9B8B36EF4C9**	2	2	1	5	1
044f28b0-564a-42d8-9df1-ba3fd4d6c014	**TCGA-E9-A22D-01Z-00-DX1.b2867437-0add-4b7d-8002-fb09ed961942**	2	2	1	5	1
2c83bc8f-ac3c-4749-921a-cdef6f872704	**TCGA-BH-A0BT-01Z-00-DX1.9087B9E7-C0CD-4179-AF57-AD9255785169**	1	2	1	4	1
96ce4bc0-f846-4a4a-b2a8-925ed5dd423e	**TCGA-E2-A15K-01Z-00-DX1.9F424BE2-9BFE-4DFF-8CC9-10D2DADBBEA7**	3	2	1	6	2
35ed3b43-99d6-4a9e-9b1b-1ded7e2ee7c7	**TCGA-E2-A15O-01Z-00-DX1.4D0F3975-93EA-4DC2-AD0C-A76A24C3AE0C**	3	2	1	6	2
6d4418e8-d621-4ace-9e1f-3fcc7d2c9546	**TCGA-E2-A14Q-01Z-00-DX1.C19BA7FD-D986-4E3B-9A79-F3531A78F05D**	3	3	1	7	2
63f10c46-8870-40dd-a531-6317306be707	**TCGA-E2-A15D-01Z-00-DX1.AA5AF847-3635-4BAF-AAC3-BADB4A1B2CB1**	1	2	1	4	1
86538d07-4a39-4c65-80fa-3e530660eb46	**TCGA-AO-A12A-01Z-00-DX1.4E9609A7-9AAD-40A8-8344-8369DF998006**	2	2	1	5	1
0ce5fcd8-3ace-4213-ae59-844c11a45f7b	**TCGA-BH-A1EU-01Z-00-DX1.5A0956EF-0100-47FD-9026-1994CF22D0F1**	3	2	1	6	2
c2e4fe6d-db89-42df-9594-8ef7a6d11490	**TCGA-E2-A1IN-01Z-00-DX1.F63F004F-847D-41B0-BAEF-3189D4965838**	2	2	1	5	1

PATHOLOGIST 4

UUID	SLIDE ID	Tubule formation	Nuclear pleomorphism	Mitotic count	Total score	Grade
d2c7ad27-6090-421e-a12e-2cb66a5417e4	TCGA-E2-A1LK-01Z-00-DX1.5EBAA1F4-F1B4-4938-A51F-0246621BB0ED	3	3	2	8	3
b1e64f03-c419-421e-b801-2439bace5ad5	TCGA-AO-A0JB-01Z-00-DX1.250FE098-345B-4981-9236-0519E1C9058E	2	2	2	6	2
a7ccf35e-56a9-4bd9-904c-59b1b0cc33b4	TCGA-E2-A1L6-01Z-00-DX1.AFE87067-2BFD-42C2-9334-9DDE8AB61B49	1	1	1	3	1
d9ab7666-6cdd-4cb4-89fd-e7916e10986a	TCGA-A1-A0SN-01Z-00-DX1.5E9B85AE-AFB7-41DC-8A1B-BD6DA39B6540	2	2	1	5	1
469579d1-f578-4956-aab0-9e7728a88ff7	TCGA-A7-A13D-01Z-00-DX1.D206783C-FA6A-4B6A-B3AA-4132A2C9626B	2	3	2	7	2
454d3eb9-ff96-4cd4-9dec-17252819ed13	TCGA-B6-A0WW-01Z-00-DX1.64048633-1D1A-4074-8C50-7641159355DB	2	1	1	4	1
9b91d3c8-c250-4f74-9ff7-bb8d11f813a7	TCGA-D8-A1XL-01Z-00-DX1.FDF07020-8F40-4C00-9023-E5F40E0D8A7C	3	2	1	6	2
1f4d56a0-b948-4a65-8b3a-f66d30317e0b	TCGA-D8-A13Y-01Z-00-DX2.0043D2BB-B04D-4A44-AAB1-1CAAD97AC246	2	2	2	6	2
b658e65c-636f-43b9-8c52-a46ba2850044	TCGA-A2-A0SU-01Z-00-DX1.22420EE2-4FEB-42F3-9547-4739F0F73D50	2	2	2	6	2
f6f7b32b-33e4-462a-9f83-2a3f74a6302a	TCGA-E2-A108-01Z-00-DX1.B110ED43-08A4-476A-A658-1CA75F7C0DDE	1	1	1	3	1
55eb1c6a-2290-4458-afa2-f259d80e6395	TCGA-LL-A5YN-01Z-00-DX1.F221939B-3680-4B95-93A9-BE8599550E87	2	2	1	5	1
d29fb01b-dfb0-49fe-b42c-98dd32a306c1	TCGA-OL-A5D7-01Z-00-DX1.A4A45393-9AE1-4370-8B92-CB85CDB04934	3	3	3	9	3
40b5da84-5b98-4444-9745-2f1ebe5132e4	TCGA-WT-AB41-01Z-00-DX1.75BDFDF2-CD87-46D1-B32C-725741CB02BE	2	1	1	4	1
a18c60c5-3053-477a-b892-305bf4e5bfe7	TCGA-D8-A27R-01Z-00-DX1.F6E2FD1C-0666-4788-8D95-A76D15907270	2	3	2	7	2
fcb893aa-c7aa-438d-9d6d-c0adb397fa1e	TCGA-E2-A9RU-01Z-00-DX1.A06BE284-B9DC-4B45-A202-A9D027AEEDD9	2	3	2	7	2
68458986-1efd-4fce-9e65-ce5430ab125c	TCGA-3C-AALJ-01Z-00-DX1.777C0957-255A-42F0-9EEB-A3606BCF0C96	2	1	1	4	1
069a8f13-04e2-41c7-ab64-90a465c7c4bc	TCGA-AR-A0TQ-01Z-00-DX1.2BEA298C-6B3D-4133-ADBC-769E62CEFFA0	2	2	2	6	2
6f29dfa9-d166-4f07-9cdb-0daceb7341e9	TCGA-D8-A27R-01Z-00-DX2.31F47D8F-DFD7-42AE-BBBA-7DBBA12FA97D	2	3	2	7	2
0dcc8304-dfd1-4e79-b23f-30f1143c897c	TCGA-E2-A14N-01Z-00-DX1.15F5644F-CA9F-4688-B56E-BCC00CA4769B	3	3	2	8	3
63858a48-c99e-4080-97f0-419f662ef195	TCGA-D8-A1XD-01Z-00-DX1.E500D561-1F49-4F08-99AE-E8345F21B406	3	2	1	6	2
0279f555-9738-41e0-b89f-ded25829adc0	TCGA-E2-A1L9-01Z-00-DX1.F2CC1036-8EEE-4664-962E-541B8ACB10DE	2	2	1	5	1
7b3d2ef5-0b8b-4b3e-8210-c77f8302e437	TCGA-E2-A15C-01Z-00-DX1.26E13415-1D37-43C7-9EBB-4411BE7FCE10	1	2	1	4	1
bcd6977f-b269-48c4-b088-ddf8d6d95240	TCGA-GM-A2DF-01Z-00-DX1.CD0BE6D7-2DB3-4193-84CC-F9BE7BF18CC2	1	2	1	4	1
4fa9d2cb-379e-4183-b5c6-a8ba2a15ee57	TCGA-AR-A0U1-01Z-00-DX1.276433E7-E841-42D2-AF21-762F2FEA3B9B	2	2	2	6	2
33f7413f-6339-4389-98dd-eee280233684	TCGA-E2-A1LH-01Z-00-DX1.F85384B7-1EBF-4F57-A45B-4A668B68E535	2	2	1	5	1
9db0bfe8-9f66-4416-ab90-e0b1f91d2e01	TCGA-D8-A1XR-01Z-00-DX2.A103FB8B-4397-4DD4-8587-90A736407484	2	3	3	8	3
f554759b-e293-45f5-be85-34a561e36027	TCGA-AR-A0U2-01Z-00-DX1.03E2ADD9-F20F-44DA-93D1-D10BBD3844A5	2	2	2	6	2
75f48929-04a3-4970-8572-29b1068b2d85	TCGA-D8-A147-01Z-00-DX1.159094F2-BB78-4910-B7AE-3D7CAAB1DAD9	2	3	1	6	2
45b31b73-adeb-4793-837f-8a9f957cffad	TCGA-A2-A3XY-01Z-00-DX1.E57FC9BF-411E-4028-AC10-8BCA5D0C8472	2	3	2	7	2
e47bd352-9d96-4abd-b673-9d30038d218a	TCGA-B6-A0RI-01Z-00-DX1.E39951A4-AC6A-4B07-851B-F35CB86D79AA	2	1	1	4	1
54d20f52-a7b1-4814-ba36-082108f23b59	TCGA-E9-A22A-01Z-00-DX1.d986c9eb-2c54-4663-a54b-04c0756db6db	2	2	1	5	1
df1645c7-0db5-4262-8c8d-b8577f272914	TCGA-A2-A0YJ-01Z-00-DX1.8135C74E-DAA8-4C8E-AF14-A4B5B57695BE	3	3	2	8	3
26eb27eb-84c8-4fea-a51b-c3eb5c484b33	TCGA-E2-A1LI-01Z-00-DX1.503dd2fa-23ef-4b11-8aab-301e069eaa88	3	2	2	7	2
e5069ea5-1011-46cf-9a3c-925825eeb81e	TCGA-A2-A0T5-01Z-00-DX1.128C288B-B357-439B-A8D4-8E7DEBF73E4E	1	2	1	4	1
e15f67af-fb62-42c8-a451-83b035867106	TCGA-D8-A1JD-01Z-00-DX1.6D215B14-DD90-4635-8645-AF06EBD9BA3F	1	2	1	4	1
f273a858-1cfa-4983-ace4-b1e876d64473	TCGA-A1-A0SF-01Z-00-DX1.7F252D89-EA78-419F-A969-1B7313D77499	1	1	1	3	1
9b8dec44-e674-4dc7-a8a8-c962c907b64b	TCGA-D8-A1JC-01Z-00-DX2.854ABF5D-40F1-48AE-802F-97D75497F1FD	2	3	2	7	2
206ccd79-310d-40a4-852c-03930e685304	TCGA-EW-A6SB-01Z-00-DX1.D56E1922-01A9-4AEE-AB95-D69447DD13EE	3	2	3	8	3
8ffdde89-cb67-4707-a00c-b8d3089a7bf5	TCGA-AR-A256-01Z-00-DX1.950D4546-4BF4-4380-9877-51D86A93D755	2	2	2	6	2
d93a5cab-9a8a-42c8-973f-5868e748e8e7	TCGA-LD-A9QF-01Z-00-DX1.092108DF-1A60-459E-ACE6-5A71826A98D1	3	2	2	7	2
b411c802-4c8d-4b65-8eee-72a538f14f65	TCGA-C8-A12Q-01Z-00-DX1.CE74E5B7-FD30-4CBE-8716-ECCF2213AAC3	2	2	2	6	2
47ce0c0f-a5df-4a84-b980-b7f4ebbbeb9d	TCGA-B6-A0RO-01Z-00-DX1.3ADBFF05-92CE-41B5-BD49-CB3CE5B74CC9	1	1	1	3	1

bc6cd9e3-a33c-40da-bc82-cf85d2d03106	TCGA-A2-A25C-01Z-00-DX1.F8E6044A-435E-42D5-94FF-C0F572F1ED99	1	1	1	3	1
25aec062-60d1-446e-a1c6-0c79cc74a770	TCGA-E2-A154-01Z-00-DX1.01FC9B1A-8ECD-4467-9EDD-0B02E4AEEF72	2	2	2	6	2
1db81db6-77eb-4aee-9e20-5d9cad3d4917	TCGA-LL-A740-01Z-00-DX1.757D94A5-EF0F-4A0E-99A9-8809B66438DA	2	3	2	7	2
1bac9786-4d56-4f08-9792-ed131b398907	TCGA-D8-A1JH-01Z-00-DX1.4A4F2502-612C-421D-9F64-444BF2C85620	2	1	1	4	1
1bac9786-4d56-4f08-9792-ed131b398907	TCGA-E2-A573-01Z-00-DX1.D6633BC2-524D-4153-952F-6B1D8D067370	3	2	2	7	2
248c960a-1ed6-4393-9bdd-82dffe30786e	TCGA-BH-A0DX-01Z-00-DX1.45C27E71-9A0A-400E-93A9-5CE7780F3C5E	2	1	1	4	1
c796a50f-9129-4144-ba0b-e67f5d4fd5e8	TCGA-D8-A1JK-01Z-00-DX1.3190C919-A403-460D-9F6C-D2AB5FD3FD05	2	3	2	7	2
7a79416d-74da-48a6-8c44-df4f41b04e3a	TCGA-D8-A1XQ-01Z-00-DX1.1A17A5C7-F14B-4AD2-AD5F-D3400D86A366	3	3	2	8	3
672685f0-fe97-415f-b52b-e451ac87294f	TCGA-E2-A158-01Z-00-DX1.994C60FE-E651-4224-95E7-4669834F2338	1	1	1	3	1
c580a40b-fafe-4322-92b7-b065c8bd9f97	TCGA-E2-A10A-01Z-00-DX1.98B19EF1-0DAE-4DC6-8B0E-963CFABC6724	2	1	2	5	1
101c2a43-88d8-4cb8-b5bd-c8ec41e835b1	TCGA-EW-A1P6-01Z-00-DX1.A8024C26-6336-4858-88FD-5679795899BA	2	2	1	5	1
96f45c1d-a11a-428d-8160-f4a1b78d03b6	TCGA-D8-A1XY-01Z-00-DX2.33D96E5C-5291-4864-B282-8BACA2043586	2	3	2	7	2
ffd2e03b-702b-4a89-a3bc-25e3503e7a3c	TCGA-E9-A229-01Z-00-DX1.5B448B88-DA0C-44FF-87B3-20649A4A26FE	2	2	3	7	2
411e68e5-a78e-486d-aba6-fdef038f9082	TCGA-LL-A442-01Z-00-DX1.9275EDBD-1C89-4AF3-B02B-19F613A4E083	3	2	2	7	2
c0c80b78-109c-4f6c-8606-98cc0961c389	TCGA-BH-A0EI-01Z-00-DX1.929E126A-C9F8-4240-BF00-B6C4A57B7FF6	1	2	2	5	1
ce2b8842-4ddf-4415-9c7d-f211328c3d71	TCGA-A7-A0CE-01Z-00-DX2.5AD1DB65-10E7-4996-AB5E-13D7851EC5FA	3	3	2	8	3
ebef3a2a-227f-40f7-9a6d-5138fa43c872	TCGA-E9-A227-01Z-00-DX1.823062BF-3444-489B-AF91-AAD4ECAA1DC7	2	2	2	6	2
86867852-64fe-43e6-894d-b53b146c3518	TCGA-AR-A0TZ-01Z-00-DX1.2D58BE38-03F6-4310-8E06-F1A523FB0904	2	3	2	7	2
a8ec7eb3-4e63-4368-b45d-57c2cdfe1f45	TCGA-LL-A7T0-01Z-00-DX1.B03BBA63-ACF4-4BCA-9F2B-F631F0C6A25C	3	2	3	8	3
d661da33-ef97-4e13-a954-4e0eb6a16573	TCGA-C8-A132-01Z-00-DX1.6CCE1FE0-BB4B-4046-BAF0-43AA110B2EBE	2	3	2	7	2
bd74ee11-89ac-45f0-8257-a5f37bb14078	TCGA-E9-A1R0-01Z-00-DX1.187C58EA-132E-4B3C-BAD0-7F59101D5C4D	3	2	2	7	2
b9406762-f609-464e-a518-40277ce6bd7f	TCGA-BH-A0B0-01Z-00-DX1.316D35DB-7F13-4AE5-82A7-5716D2519669	2	3	2	7	2
b3f4c01e-4068-45bc-8a7f-e03683f12d42	TCGA-E2-A10E-01Z-00-DX1.C45030A9-CC1A-4BA7-8F62-872619C5AD5E	2	1	1	4	1
3631967b-07b8-4275-92fb-6f66abe465ec	TCGA-AQ-A0Y5-01Z-00-DX1.f68f5b49-30fa-4fb6-bec6-5da9f6809d02	2	2	2	6	2
b28b6590-f8f2-43fa-90f2-cf4b558797d9	TCGA-E2-A574-01Z-00-DX1.60341091-B118-4F20-9ADB-FB2886790B0E	2	2	3	7	2
43b25038-2f66-4bd4-b2af-69130271fd1d	TCGA-BH-A0DT-01Z-00-DX1.73AFCEBB-06B1-4870-ADA2-881511B1BE2D	1	1	1	3	1
86925470-1065-44f5-a4e5-a3c55a73c995	TCGA-OL-A5D6-01Z-00-DX1.6B11331B-4A0D-4E13-B054-A5C7A6FC3AAC	2	3	2	7	2
179185d2-5279-4e19-b9da-1f2296973156	TCGA-C8-A12Y-01Z-00-DX1.A15CB3E2-E145-4C75-8FEA-1DD503CD7C20	2	3	3	8	3
f6830586-1dc2-4cfd-8d99-b4b7c9161341	TCGA-D8-A1JE-01Z-00-DX2.CCF3DFEF-E851-425A-BCD0-0F7B377A00BC	2	2	3	7	2
66cfc895-af23-4992-9e27-ad3988b54af7	TCGA-BH-A1F6-01Z-00-DX1.E83F0DC0-EA2C-4641-81B0-8702B9C5D579	2	3	3	8	3
9f66d62b-cd1f-459a-9c4d-40b554faccca	TCGA-D8-A1XR-01Z-00-DX1.7F443346-C564-47B6-9736-6944230CAF46	2	2	2	6	2
81100322-018d-4957-bec7-c1833e288434	TCGA-D8-A1Y0-01Z-00-DX1.10F40197-4174-43CC-AAD3-8CB85154FB2D	2	3	2	7	2
f1b21fac-b36b-4fd4-b18f-b29337df4ce4	TCGA-E2-A14T-01Z-00-DX1.61B4C988-6D75-447B-A5F4-9DE92CEACC9F	1	2	1	4	1
edaed3cc-ec4f-4792-aab7-27078b0f30a3	TCGA-S3-AA10-01Z-00-DX1.C0468882-0DD8-4FC5-8C2F-E18BE8000F69	3	3	3	9	3
7d0b8ace-0a94-444a-b48e-6cdb4a7665ca	TCGA-A1-A0SM-01Z-00-DX1.AD503DBD-4D93-4476-B467-F091254FDF78	2	2	1	5	1
fb49bd4a-63e7-472d-9ff6-b297c46e43cf	TCGA-AR-A24Z-01Z-00-DX1.C88AFF18-A8E4-426A-BEA3-70566DE39C46	2	2	2	6	2
745f1703-c59d-4d43-b5ba-d908bf1498c3	TCGA-E2-A14R-01Z-00-DX1.DDE62ED5-1FC0-4B3B-A874-95C08B33AB20	3	2	3	8	3
ff1fe2c9-2f02-4a50-a0dc-1509a6b0d7a4	TCGA-D8-A1J8-01Z-00-DX1.EADAB43A-87C6-47FA-9120-25B69E23366D	2	2	1	5	1
c2c93798-a4df-47ff-a281-8960ae8c5c41	TCGA-C8-A12P-01Z-00-DX1.670B5DE8-07B0-4E4C-93FA-FA3DFFCCE50D	3	3	2	8	3
c713f828-2725-4901-a9fe-01d3192edc46	TCGA-GM-A2DB-01Z-00-DX1.9EE36AA6-2594-44C7-B05C-91A0AEC7E511	3	2	3	8	3
fb9961aa-e11f-4f75-bc5a-990e9302ae6e	TCGA-BH-A202-01Z-00-DX1.8CECDB74-5E6F-4CE8-B52C-A89E574F38FB	2	3	3	8	3
0a886f18-c44c-4b5e-b243-6df6e27f426a	TCGA-GI-A2C8-01Z-00-DX1.09BD8AC9-645A-4C8B-9B36-77D833BDBA09	2	3	2	7	2
d953668b-4a60-4e7c-a001-0d74e0ad6e93	TCGA-E2-A14Y-01Z-00-DX1.804A22A3-FD8D-4C8A-A766-48D28434DE22	3	3	2	8	3
73c4e299-8b32-4dc0-b83d-d1f45d78e1b4	TCGA-AC-A23E-01Z-00-DX1.F12A5A87-72CF-42F8-A6EC-8E7FAD80B1F7	2	1	1	4	1
8b5519e1-cbef-407d-b4b0-0a709cb9e924	TCGA-E2-A1LE-01Z-00-DX1.22856B2A-FBAA-4530-AEEC-E8F77BDA7F7F	3	1	1	5	1
8445cfc1-a20a-4b5b-861a-ae8412f4d497	TCGA-BH-A0BP-01Z-00-DX1.63A87C1D-87FA-494D-9836-74290B5DC30D	2	2	1	5	1
cce52fce-a092-4c66-af4e-a19cace6e154	TCGA-E2-A109-01Z-00-DX1.FCF5E9FC-F9FE-4F5F-96DD-5628E2609BEF	2	2	1	5	1
abf27f46-f39a-4f2d-8e7b-423e5da54419	TCGA-E2-A14X-01Z-00-DX1.24ADDA43-F127-4A6B-9AAD-2FAD982A853D	2	2	2	6	2
c9eda175-ef25-49f1-beb1-3a8cb99c1f37	TCGA-E2-A15J-01Z-00-DX1.BF7901D1-30B1-4A76-B0A5-E9B8B36EF4C9	1	2	1	4	1
044f28b0-564a-42d8-9df1-ba3fd4d6c014	TCGA-E9-A22D-01Z-00-DX1.b2867437-0add-4b7d-8002-fb09ed961942	2	2	3	7	2
2c83bc8f-ac3c-4749-921a-cdef6f872704	TCGA-BH-A0BT-01Z-00-DX1.9087B9E7-C0CD-4179-AF57-AD9255785169	2	1	1	4	1
96ce4bc0-f846-4a4a-b2a8-925ed5dd423e	TCGA-E2-A15K-01Z-00-DX1.9F424BE2-9BFE-4DFF-8CC9-10D2DADBBEA7	3	2	2	7	2
35ed3b43-99d6-4a9e-9b1b-1ded7e2ee7c7	TCGA-E2-A15O-01Z-00-DX1.4D0F3975-93EA-4DC2-AD0C-A76A24C3AE0C	3	2	2	7	2
6d4418e8-d621-4ace-9e1f-3fcc7d2c9546	TCGA-E2-A14Q-01Z-00-DX1.C19BA7FD-D986-4E3B-9A79-F3531A78F05D	2	1	1	4	1
63f10c46-8870-40dd-a531-6317306be707	TCGA-E2-A15D-01Z-00-DX1.AA5AF847-3635-4BAF-AAC3-BADB4A1B2CB1	2	1	1	4	1
86538d07-4a39-4c65-80fa-3e530660eb46	TCGA-AO-A12A-01Z-00-DX1.4E9609A7-9AAD-40A8-8344-8369DF998006	2	1	2	5	1

0ce5fcd8-3ace-4213-ae59-844c11a45f7b	**TCGA-BH-A1EU-01Z-00-DX1.5A0956EF-0100-47FD-9026-1994CF22D0F1**	2	1	1	4	1
c2e4fe6d-db89-42df-9594-8ef7a6d11490	**TCGA-E2-A1IN-01Z-00-DX1.F63F004F-847D-41B0-BAEF-3189D4965838**	2	1	1	4	1
