# Supplementary material for: Comparative Evaluation of Breast Ductal Carcinoma Grading: A Deep-Learning Model and General Pathologists’ Assessment Approach
Source: Diagnostics (Basel). 2023 Jul 10;13(14):2326. doi: 10.3390/diagnostics13142326 (PMC10377791; doi:10.3390/diagnostics13142326)

Example of generated "tissue" using Aiforia's image augmentations features

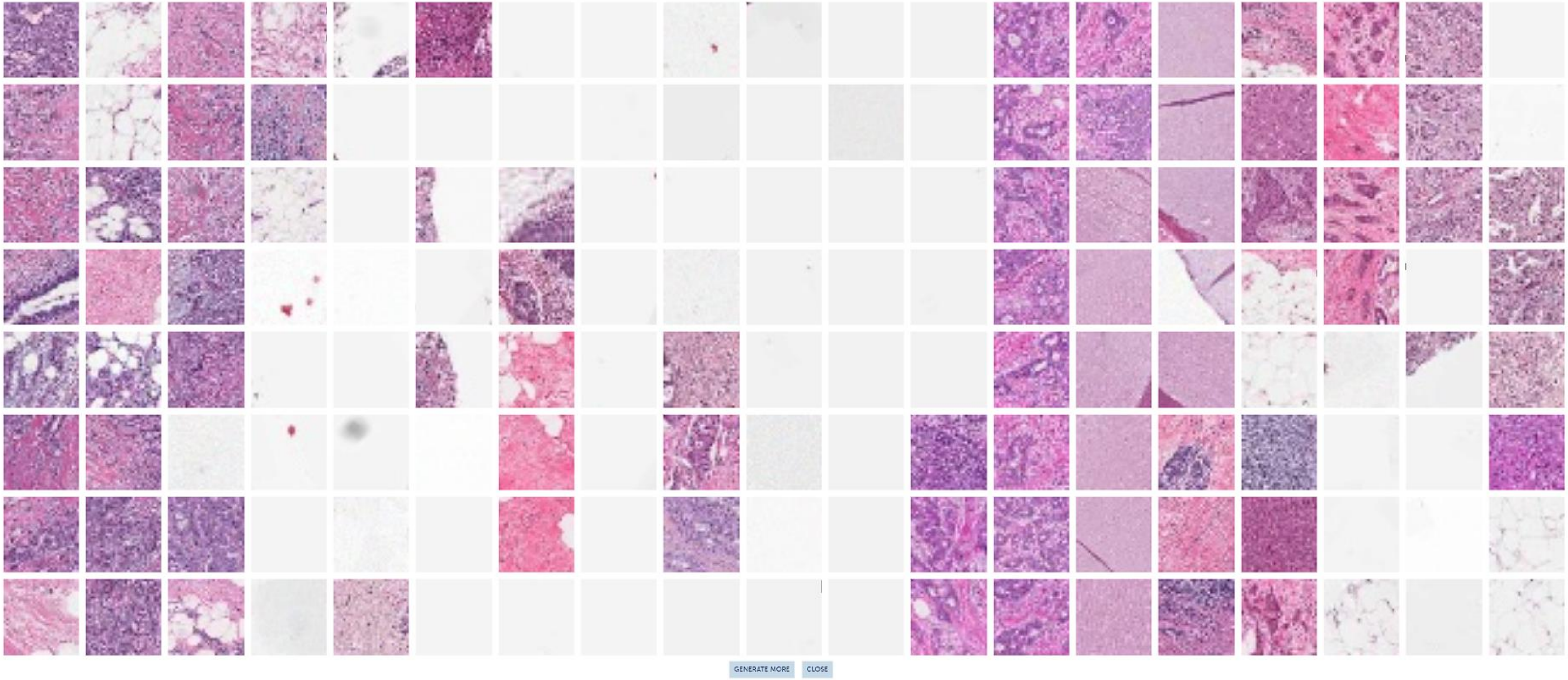

Example of generated “normal tissues vs. tumor tissue” using Aiforia's image augmentations features

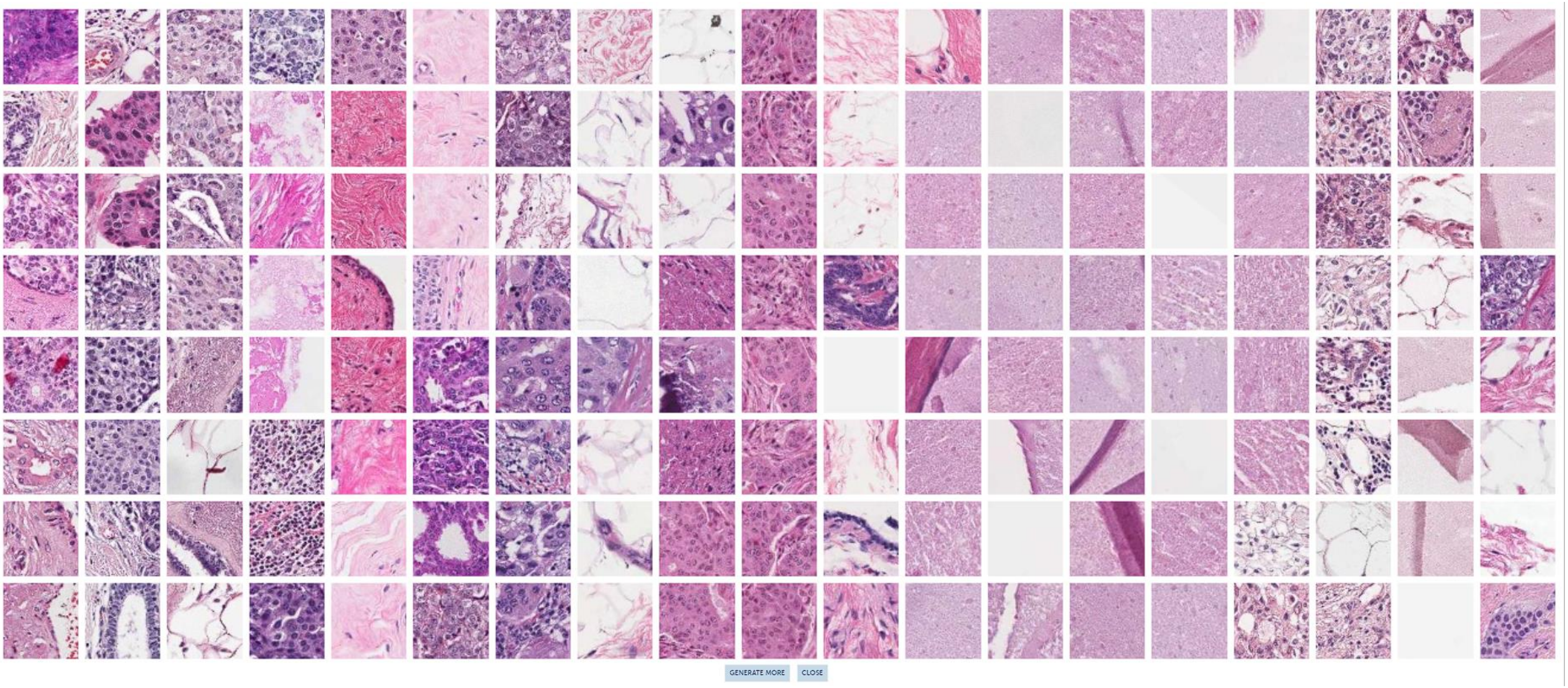

Example of generated “solid components and tubule formation” using Aiforia's image augmentations features

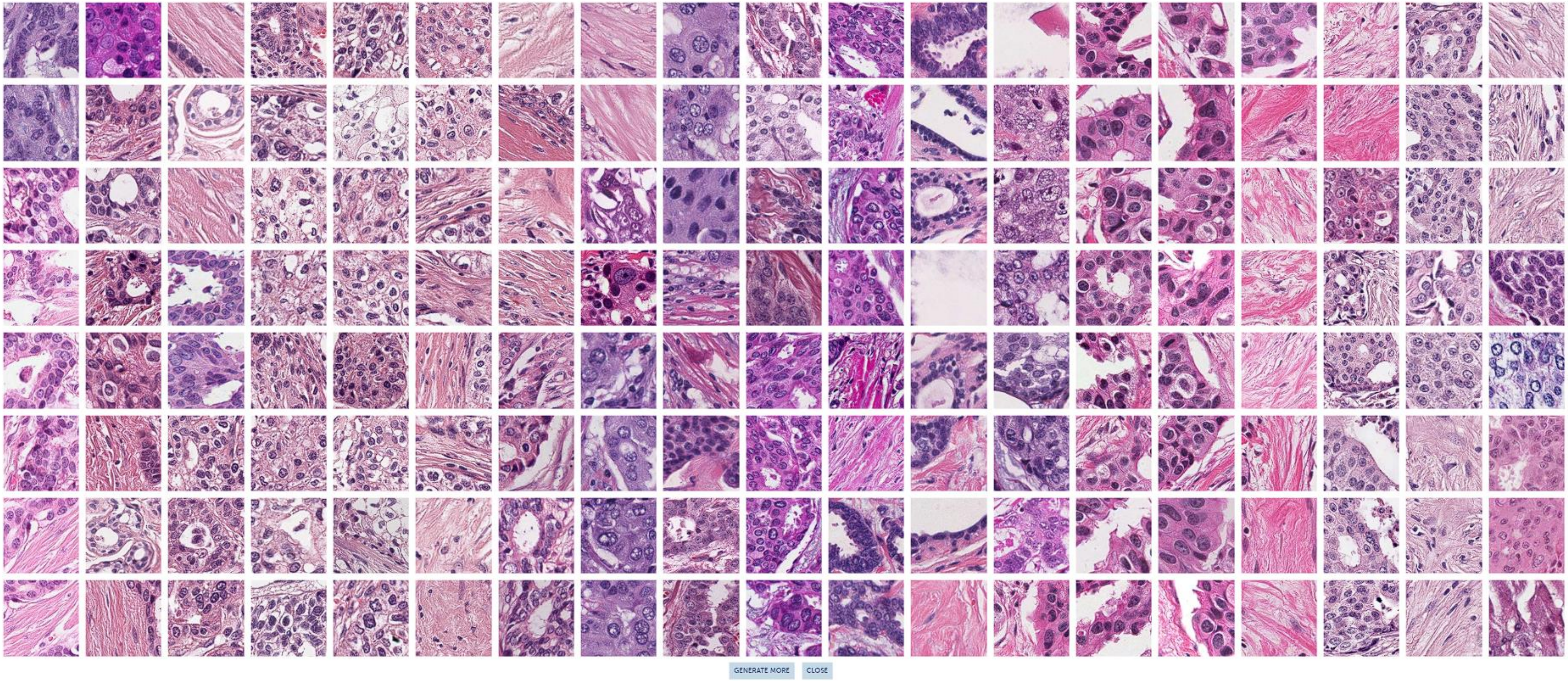

Example of generated “pleomorphism and mitosis” Aiforia's image augmentations features

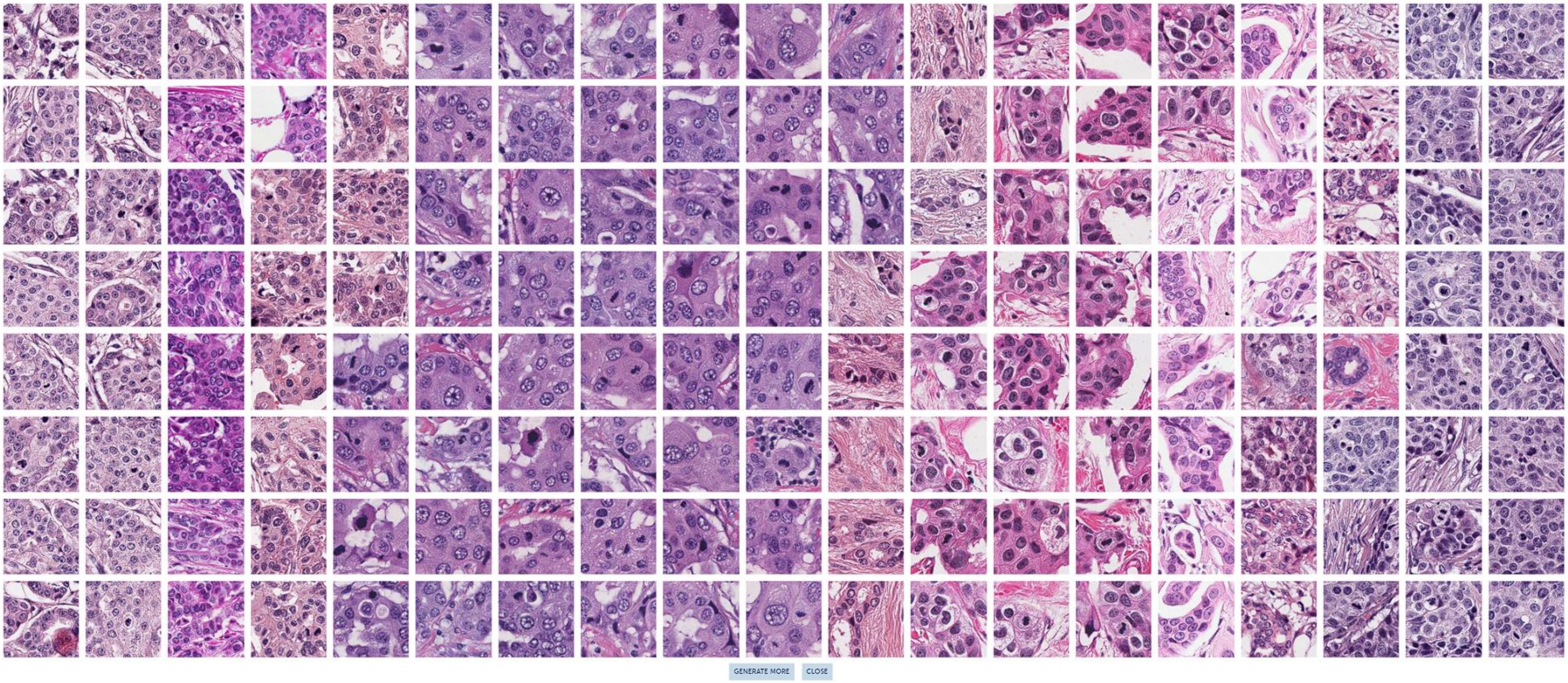

Supplement: Supplementary file 1 [file diagnostics-13-02326-s001.zip › S4 - image augmentation features.pdf]
